# Cardiometabolic traits mediating the effect of education on the risk of DKD and CKD: a Mendelian randomization study

**DOI:** 10.3389/fnut.2024.1400577

**Published:** 2024-08-13

**Authors:** Yukai Wang, Mengmeng Chen, Lin Wang, Yonggui Wu

**Affiliations:** ^1^Department of Nephropathy, The First Affiliated Hospital of Anhui Medical University, Hefei, Anhui, China; ^2^Center for Scientific Research of Anhui Medical University, Hefei, Anhui, China

**Keywords:** cardiometabolic traits, education, diabetic kidney disease, chronic kidney disease, mediation analyses, susceptibility genes, Mendelian randomization

## Abstract

**Background:**

Both diabetic kidney disease (DKD) and chronic kidney disease (CKD) are more prevalent among individuals with lower levels of education in observational studies. To quantify the mediation effect of recognized cardiometabolic traits, we obtain causal estimates between education and DKD as well as CKD.

**Materials and methods:**

We assessed the causal effect of education on DKD and CKD, separately estimated the causal effect of 26 cardiometabolic traits on DKD and CKD, and finally calculated the mediating effects and mediating proportions of each using two-step, two-sample multivariable Mendelian randomization (MVMR). Furthermore, the genetic association between exposure, mediators, and outcomes was investigated using linkage disequilibrium score (LDSC) regression analysis. Expression quantitative trait loci (eQTL) were retrieved from the Genotype-Tissue Expression Project (GTEx) v8 to serve as genetic instrumental variables. Transcriptome-wide association studies (TWAS), Bayesian colocalization analysis, and Summary-data-based Mendelian Randomization (SMR) analysis were performed to explore underlying susceptibility genes between education, mediators, and kidney diseases.

**Results:**

Higher education with a genetically predicted 1-SD (4.2 years) was linked to a 48.64% decreased risk of DKD and a 29.08% decreased risk of CKD. After extensive evaluation of 26 cardiometabolic traits, 7 and 6 causal mediators were identified as mediating the effects of education on DKD and CKD, respectively. The largest mediating factor between education and DKD was BMI, which was followed by WHR, T2D, fasting insulin, SBP, fasting glucose, and DBP. In contrast, candidate mediators in the education-to-CKD pathway included BMI, followed by cigarettes smoked per day, WHR, SBP, T2D, and DBP. MR analysis revealed that TP53INP1 was found to be a shared susceptibility gene for cardiometabolic traits and DKD, while L3MBTL3 was found to be a shared susceptibility gene for cardiometabolic traits and CKD.

**Conclusion:**

Our findings provide solid evidence that education has a causally protective effect on the development of DKD and CKD. We additionally reveal significant directions for intervention on cardiometabolic traits that mitigate the negative effects of educational inequities on the onset of DKD and CKD. Our work demonstrates a shared genetic basis between education, cardiometabolic traits, and kidney diseases. Future research aiming at lowering kidney risk may benefit from these findings.

## Introduction

The leading cause of chronic kidney disease (CKD) is diabetic kidney disease (DKD), which is brought on by metabolic and hemodynamic abnormalities driven on by long-term diabetes (1, 2). In addition to the high expenses for healthcare that place an enormous burden on social, financial, and health systems all over the world, CKD and diabetes are essential risk factors for cardiovascular disease and play a significant role in global morbidity and mortality (3, 4). Recent observational studies have demonstrated an adverse association between education level and CKD, with modifiable risk variables such as diabetes, smoking, BMI, WHR, and hypertension being highlighted as potential drivers of this correlation (5). Another study demonstrated that type 2 diabetes (T2D) patients with low educational attainment were more likely to experience DKD and other diabetic microvascular events (6). Improving these cardiometabolic traits could reduce the risk of renal disease since they are directly associated with the onset of DKD and CKD (7, 8). Due to the influence of confounding factors and reverse causality in traditional observational studies, the epidemiological evidence to date suggests that education and cardiometabolic traits are related to the risk of DKD and CKD, but it does not provide a good estimate of their causality. The ability of education to reduce the risk of DKD and CKD through cardiometabolic traits is therefore unknown, as is the extent to which cardiometabolic traits can account for the effect of education on DKD and CKD. Studying more concerning this topic will help with understanding the etiology of DKD and CKD as well as offer strategies for prevention.

Genome-wide association study (GWAS) and Mendelian randomization (MR) analyses allow us to explore the causal relationship and direction of education, cardiometabolic traits, DKD, and CKD. Mendelian randomization mimics the mechanism by which genes are randomly distributed from parents to offspring during gamete formation and conception, and it allows us to assess the causal association between exposure and outcome (9). Mendelian randomization, a natural randomized controlled experiment, evaluates the causal relationship between exposure and outcome using genetic variables related to the targeted phenotype. Additionally, MVMR can investigate the causal association between the outcome of interest and multiple exposures shared susceptibility to single nucleotide polymorphisms (SNPs) (10, 11). Mediation analysis is a method of decomposing the effects of an exposure on an outcome, which are categorized into direct effects, and indirect effects through mediating variables. These effects are decomposed through the use of MVMR to estimate the causal effect between exposure, mediator, and outcome (12). To evaluate the causal relationships between education, cardiometabolic traits, DKD, and CKD in this research, we utilized univariable Mendelian randomization (UVMR). In addition, we used multivariable MR to assess which cardiometabolic traits mediate the relationship between educational attainment and DKD and CKD, as well as what proportion of the mediation was accounted for by these factors. Understanding these mediating effects will make clinical practice guidance more accessible.

Nearly 90% of genetic variants contained by GWAS are found in the noncoding region of the genome, meaning that GWAS investigations alone are unable to identify the genes that cause disease. Expression quantitative trait loci (eQTL) are genomic loci that explain variation in gene-expression levels. Prioritizing putative causative genes from GWAS studies has been made possible by the merging of GWAS and eQTL research, since GWAS catalogs noncoding variants linked to disease while eQTL finds the relationship between variations and gene expression (13). TWAS aims to test whether the expression of a gene mediates the genotype effect on the phenotype development. Renal function susceptibility gene loci have been identified through the utilization of TWAS combined with Bayesian colocalization analysis and SMR analysis (13–15). Genetic evidence can be used to guide drug target development, which can dramatically increase the chance of drug approval (16). Even though their study produced significant results, more MR analysis is recommended to identify susceptibility genes related to education, cardiometabolic traits, and kidney diseases.

## Materials and methods

The article and its Supplementary material contain all of the supporting data that the authors declare to have.

### Data sources of exposures, mediators, and outcomes

Summary-level data based on GWAS carried out among participants with mainly European ancestry were used in this MR analysis to provide details about exposure, mediators, and outcomes (Table 1; Supplementary Table S5). Specific epidemiological evidence supporting the association of the 26 candidate mediators with DKD and CKD is presented in Supplementary Tables S6, S7. This study did not need ethical approval since its results were derived from summary-level data.

**Table 1:** Summary of the GWAS data used in the MR analyses.

Phenotype	Unit	No of participants (*n* Cases/*n* Controls)	Ancestry	Consortium/cohort	Author	Year of publication	PubMed ID
**Exposure**							
Education	SD (4.2 y)	766,345	European	SSGAC	Lee *et al*	2018	30038396
**Outcome**							
DKD	Event	312,650 (4,111/308539)	European	FinnGen	Kurki *et al*	2023	36653562
CKD^#^	Event	372,250 (9,073/363177)	European	FinnGen	Kurki *et al*	2023	36653562
CKD*	Event	480,698 (41,395/439303)	European	CKDGen Consortium	Wuttke *et al*	2019	31152163
**Candidate mediators**							
BMI	SD (4.7 kg/m^2^)	681,275	European	GIANT	Yengo *et al*	2018	30124842
BF%	SD (6.6%)	65,831	European	Meta	Lu *et al*	2016	26833246
WHR	SD (0.08)	118,003	European	GIANT	Shungin *et al*	2015	25673412
HC	SD (8.45 cm)	225,487	Mixed	GIANT	Shungin *et al*	2015	25673412
WC	SD (12.5 cm)	245,746	Mixed	GIANT	Shungin *et al*	2015	25673412
Cigarettes smoked per day	SD	249,752	European	GSCAN	Liu *et al*	2019	30643251
Pack years of smoking	SD (18.5y)	142,387	European	UK Biobank	Ben *et al*	2018	NA
Maternal smoking around birth	Event	289,727 (88,601/201126)	European	Neale Lab	Neale *et al*	2017	NA
Smoking initiation	Event	607,291 (311,629/321173)	European	GSCAN	Liu *et al*	2019	30643251
Age of smoking initiation	SD	341,427	European	GSCAN	Liu *et al*	2019	30643251
Alcoholic drinking	SD	335,394	European	GSCAN	Liu *et al*	2019	30643251
Coffee intake	SD (2.09 cups/day)	428,860	European	UK Biobank	Ben *et al*	2018	NA
SBP	SD	757,601	European	ICBP	Evangelou *et al*	2018	30224653
DBP	SD	757,601	European	ICBP	Evangelou *et al*	2018	30224653
TG	SD (41.8 mg/dL)	177,861	Mixed	GLGC	Willer *et al*	2013	24097068
TC	SD (90.8 mg/dL)	187,365	Mixed	GLGC	Willer *et al*	2013	24097068
HDL-C	SD (15.5 mg/dL)	187,167	Mixed	GLGC	Willer *et al*	2013	24097068
LDL-C	SD (38.7 mg/dL)	173,082	Mixed	GLGC	Willer *et al*	2013	24097068
Fasting glucose	SD (0.73 mmol/L)	133,010	European	MAGIC	Scott *et al*	2012	22885924
Fasting insulin	SD (0.79 pmol/L)	108,557	European	MAGIC	Scott *et al*	2012	22885924
MVPA	SD (2.084 MET-min/wk)	377,234	European	UK Biobank	Klimentidis *et al*	2018	29899525
VPA	Event	261,055 (98,060/162995)	European	UK Biobank	Klimentidis *et al*	2018	29899525
Sedentary behavior	SD	372,609	European	UK Biobank	Wang *et al*	2022	36071172
Household income	SD	397,751	European	UK Biobank	Hemani *et al*	2018	29846171
T1D	Event	24,840 (9,266/15574)	European	Meta	Forgetta *et al*	2020	32005708
T2D	Event	655,666 (61,714/1178)	European	Meta	Xue *et al*	2018	30054458

SSGAC indicates Social Science Genetic Association Consortium; GIANT, Genetic Investigation of Anthropometric Traits; GSCAN, GWAS & Sequencing Consortium of Alcohol and Nicotine use; GWAS, genome-wide association study; ICBP, International Consortium of Blood Pressure; GLGC, Global Lipids Genetics Consortium; MAGIC, Meta-Analyses of Glucose and Insulin-related traits Consortium; MET, metabolic equivalent; BMI, body mass index; BF%, body fat percentage; WHR, waist-to-hip ratio; HC, hip circumference; WC, waist circumference; SBP, systolic blood pressure; DBP, diastolic blood pressure; TG, triglyceride; TC, total cholesterol; HDL-C, high-density lipoprotein cholesterol; LDL-C, low-density lipoprotein cholesterol; MVPA, moderate to vigorous physical activity; VPA, vigorous physical activity; T1D, type 1 diabetes; T2D, type 2 diabetes; CKD^#^, this GWAS data was used for UVMR, MVMR, mediation analysis, and LDSC analysis; CKD*, this GWAS data was used for TWAS, SMR and colocalization analysis.

### Overall study design

**Figure 1:**
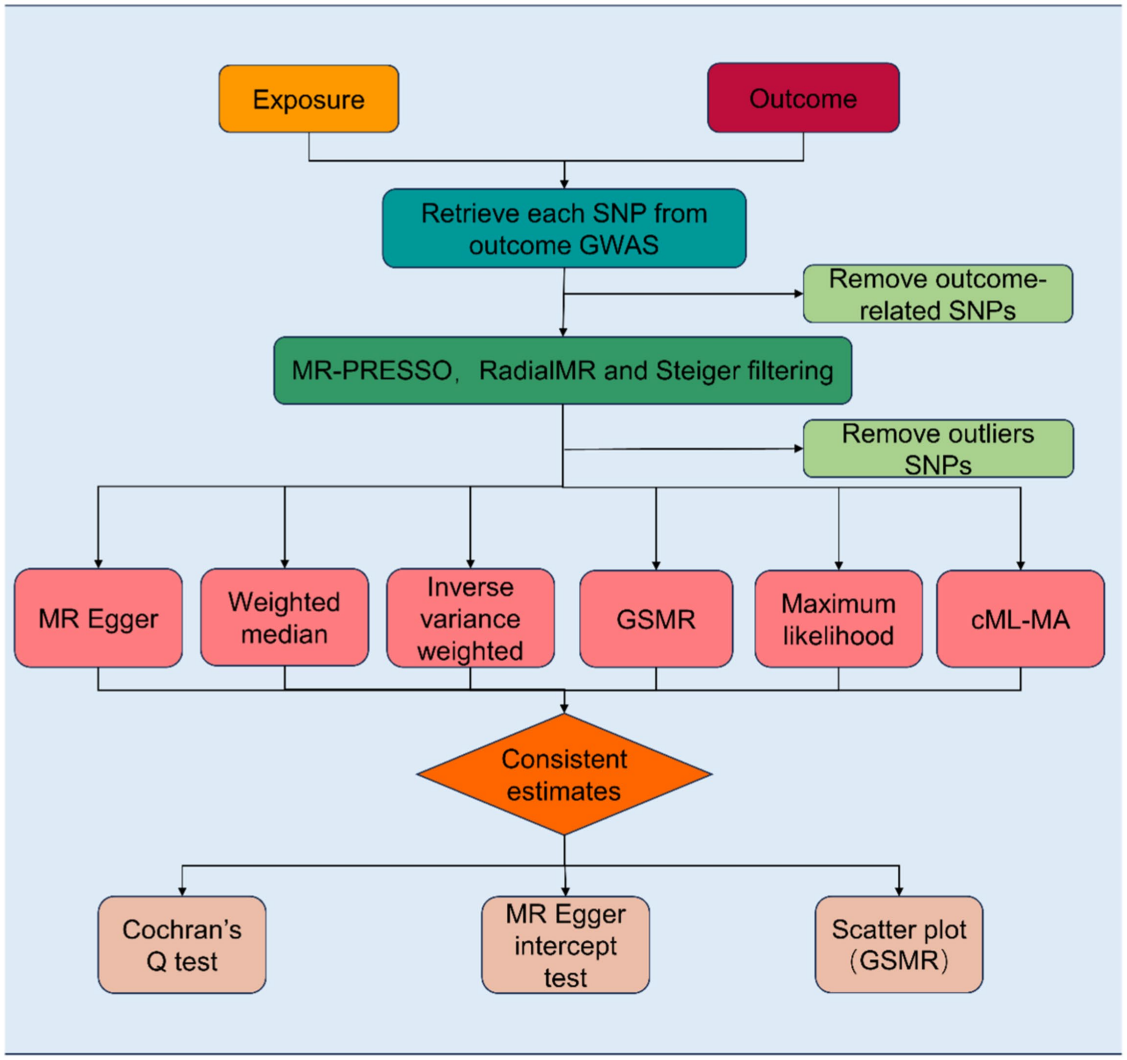
Flowchart of the Mendelian randomization study revealing the causal relationship between exposure and outcome. MR, Mendelian randomization; MR-PRESSO, Mendelian randomization pleiotropy residual sum and outlier; SNPs, single-nucleotide polymorphisms; GSMR, Generalized Summary-data-based Mendelian Randomization; cML-MA, Constrained Maximum Likelihood and Model Averaging.

**Figure 2:**
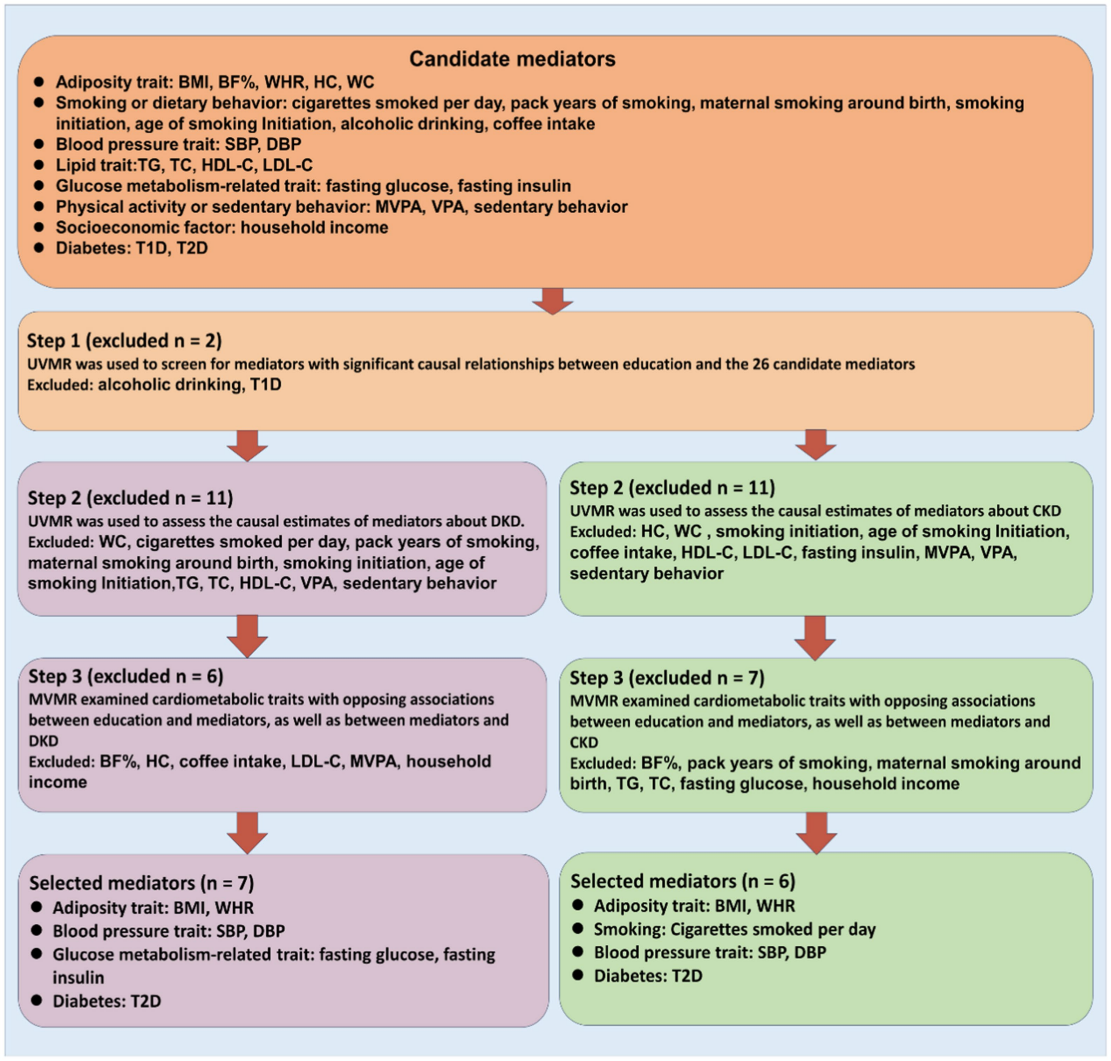
Overview of the study design. We first filter candidate mediators for the associations between education and DKD as well as CKD using stringent criteria, and then we estimate their mediation effects using two-step MR. BMI indicates body mass index; BF%, body fat percentage; WHR, waist-to-hip ratio; HC, hip circumference; WC, waist circumference; SBP, systolic blood pressure; DBP, diastolic blood pressure; TG, triglyceride; TC, total cholesterol; HDL-C, high-density lipoprotein cholesterol; LDL-C, low-density lipoprotein cholesterol; MVPA, moderate to vigorous physical activity; VPA, vigorous physical activity; T1D, type 1 diabetes; T2D, type 2 diabetes.

Based on two-sample MR analyses, we employed univariable and multivariable MR to evaluate the causal association between exposure, candidate mediators, and outcome. Three basic assumptions must be satisfied before SNPs can be used as genetic tools in MR analyses: first, the genetic variant must be associated with the exposure; second, it must not be associated with any confounding factors of the exposure-outcome association; and third, there must be no direct relationship between the genetic variant and the outcome. The study frame chart is presented in Figure 1. In order to determine if candidate mediators contribute to the exposure-outcome association, we used two-step MR methods (17). To analyze the estimation of the effects of exposure on outcomes through mediation, two-step MR approaches have been utilized extensively (18). Using LDSC regression analysis, the genetic correlation between exposure, mediators, and outcomes was investigated. The mediator screening procedure is described in Figure 2. Then, TWAS, colocalization analysis, and SMR analysis were performed to investigate underlying susceptibility genes between education, mediators, and kidney diseases. In addition, this study follows the STROBE-MR guidelines (19, 20).

### Statistical analysis

#### Primary analysis

To assess the causal relationships between education and the candidate mediators, DKD and CKD, as well as the associations involving the candidate mediators and these outcomes, we ran a two-sample UVMR. In order to calculate the direct effects between education and DKD and CKD as well as between mediators and DKD and CKD, MVMR was used to adjust for both education and mediators.

The causal effects of education on the candidate mediators, DKD and CKD, as well as the causal effects of the candidate mediators on DKD and CKD, were evaluated using UVMR. Instrumental variables for exposure acquisition were extracted from the outcome GWAS data. In addition, we orientated the effect variants in exposure and outcome to ensure the correct coordination of alleles (21). Inverse variance weighted (IVW) analysis served as the main statistical method in this study. Although IVW has effective statistical power, it is predicated on assumptions that all variables are valid instrumental variables and that effect estimates can be biased in the presence of directional pleiotropy (22). To aid in the evaluation of causal effects, we additionally utilized MR-Egger, weighted median, maximum likelihood, Constrained Maximum Likelihood and Model Averaging (cML-MA), and Generalized Summary-data-based Mendelian Randomization (GSMR). Although all SNPs have pleiotropic effects, MR-Egger can also offer unbiased estimates and sensitivity to abnormal values or less efficient (23). When up to half of the SNPs violate the assumptions about instrumental variables, the weighted median still produces accurate estimates (24). Maximum likelihood is a traditional method with low standard error, which estimates the probability distribution parameters by maximizing the likelihood function (25). ML and model averaging are combined in the cML-MA method, an MR method that is used to address both correlated and uncorrelated pleiotropic effects (25). Importantly, it does not rely on the InSIDE (Instrument Strength Independent of Direct Effect) assumption, setting it apart from other MR approaches. cML-MA has better type I error control. GSMR analysis extends the MR approach employing all the top related SNPs at a genome-wide significance level for exposure as IVs to evaluate causality. Moreover, in contrast to other methods, GSMR analysis takes into consideration sampling errors in the estimated effect sizes of the instruments on exposure as well as potential linkage disequilibrium between SNPs (26). However, at least 10 SNPs are required when using this method.

So as to calculate the direct effects between education and DKD and CKD as well as the direct effects between mediators and DKD and CKD, education and mediators were adjusted for using MVMR. MVMR was used to assess the stability of significant causal relationships by estimating the causal relationship between each exposure and a single outcome, generating causal estimates of direct effects, and adjusting for pleiotropy due to other exposures included in the MVMR analysis (12). To determine whether potential mediators generated a causal relationship between exposure and outcome, we used two-step MR methods (17). In the first step, instrumental variables from education were used to estimate the causal effects of education on cardiometabolic traits, DKD, and CKD. In the second step, instrumental variables from cardiometabolic traits were used to estimate the effects of these potential mediators on DKD and CKD, and MVMR was used to adjust for education, thus categorizing the causal effects of education on DKD and CKD into direct effects (the effects of education on DKD and CKD does not depend on the mediator) and indirect effects (the effect of education on DKD and CKD through the mediator). On this basis, the proportion of the mediating effect of the mediating factor was calculated using the product of coefficients method. We first estimated the causal effect of education on the mediator and then multiplied it by the mediator’s effect on DKD or CKD, which resulted in an indirect effect. Finally, we assessed the proportion of the mediating effect by dividing the indirect effect by the total effect, which in this case is the causal effect of education on DKD or CKD. Standard errors were generated using the delta method. A two-sided *p* < 0.05 significance level was used for all estimates. Two-sample univariate, multivariate MR analysis, and Bayesian colocalization analysis were performed using the TwoSampleMR package (version 0.5.6) (21), the MendelianRandomisation package (version 0.7.0) (27), the MRPRESSO package (version 1.0) (28), the RadialMR package (version 1.1), the MRcML package (version 0.0.0.900), the GSMR package (26) (version 1.0.9) and the coloc packages (version 5.1.0.1) in R (version 4.2.3).

### Sensitivity analyses

F-statistics were used to assess the strength of instrumental variables and the presence of bias for weak instrumental variables, conditional F-statistics were calculated in MVMR. The F statistic can reflect the strength of instrumental variables, F-statistics >10 is considered to suggest sufficient instrumental strength, while *F* < 10 indicates the risk of weak instrumental variables (29).

Furthermore, to make the results of Mendelian randomization more robust, we used multiple sensitivity tests to assess whether the MR assumptions were violated, such as the MR-Egger intercept test, Cochran’s Q test, MR pleiotropy residual sum and outlier (MR-PRESSO), RadialMR, and MR Steiger filtering. The MR-Egger intercept test can be used to detect the presence of directional pleiotropy, and the presence of a significant difference between the MR-Egger intercept and zero indicates the presence of directional pleiotropy (23). Cochran’s Q test is used to detect the presence of heterogeneity and a significant *p*-value indicates the presence of pleiotropy (30). MR-PRESSO identifies and removes potential pleiotropic outliers, but may have a high false positive rate (28). To improve the visualization of the IVW, we performed radial variants of the IVW instead of a scatter plot. Automatic detection of outliers was accomplished using RadialMR imaging (31). In addition, we used Steiger filtering to assess whether any individual SNP explained the differences in results better than exposure (32). After removing outliers identified by sensitivity analyses, MR analyses were re-performed.

### Linkage disequilibrium score regression analysis

Linkage disequilibrium score (LDSC) regression analysis is a reliable and effective tool for determining the shared genetic structure of complex human traits; it is mostly used to evaluate disease heritability and verify genetic correlation (33). We applied full GWAS summary data from exposure, mediators, and outcomes to examine genetic association in our study.

### Transcriptome-wide association studies

Examining overlapping genes could be useful in elucidating the mechanism of causality since many genetic variations impact complicated traits via regulating gene expression. To investigate the connection between genes and education, mediators, DKD as well as CKD in further detail, a TWAS analysis utilizing FUSION was performed. Gene-level expression quantitative trait loci (eQTL) for whole blood were obtained from the GTEx Consortium V8. The linear sum of Z-score weights for locus-specific independent SNPs was calculated by FUSION. Subsequently, the genetic effects of education, mediators, and DKD as well as CKD (GWAS Z scores for education, mediators, and DKD as well as CKD) were combined with mRNA expression weights. We calculated the expression weight (SNP-gene expression correlations) of TWAS using the FUSION platform, considering the reference transcriptome (34). We employ several prediction models in our study, including top1, blup, lasso, enet, and bslmm. To determine the weights for mRNA expression, choose the model that performs the best in terms of predictions. Then, the estimated gene expression level was used to study the susceptibility genes related to DKD and education as well as mediators or the susceptibility genes related to CKD and education as well as mediators. Genes classified as shared susceptibility genes have a false discovery rate (FDR) < 0. 5.

### Bayesian colocalization analysis

By eliminating the impact of linkage disequilibrium, Bayesian colocalization analysis was used to investigate whether shared susceptibility genes had a similar causal variation with DKD and CKD in the genome region (35). The following five hypotheses served as the foundation for the colocalization analysis: (i) H0: The genomic region contains no causal variant for exposure or outcome; (ii) H1: a single causal variant only significantly related to exposure; (iii) H2: a single causal variant significantly related to outcome; (iv) H3: the genomic region contains causal variables that are significantly related to exposure or outcome, but are driven by different causal variants; and (v) H4: The same causal variants drive both exposure and outcome. Using the default parameters, colocalization analysis of the identified causal genes was carried out (P1 = 1 × 10^−4^; P2 = 1 × 10^−4^; P12 = 1 × 10^−5^). PPH4 > 0.8 is regarded in this work as strong evidence of co-location (35, 36).

### Summary-data-based Mendelian randomization analysis

To confirm that shared susceptibility genes cause DKD and CKD, further SMR analysis was carried out (37). To determine whether shared susceptibility genes result from common genetic variation rather than genetic linkage, the Heterogeneity in Dependent Instruments (HEIDI) test was conducted if there were more than three SNPs. SMR analysis and HEIDI testing need to be performed using SMR software (version 1.3.1). In SMR analysis, *p* < 0.05 was the significant level. The causal relationship between exposure and outcome was not affected by linkage disequilibrium, as shown by a *p* > 0.05 in the HEIDI test.

## Results

### Effect of education on DKD and CKD

The causal effects of education on DKD and CKD were explored using two-sample UVMR analyses. The specific details concerning the genetic instruments we employ can be found in Supplementary Table S1. According to UVMR analysis, each 1-SD higher genetically predicted education was associated with 0.5136 times lower odds of DKD (OR 0.5136; 95% CI 0.4212, 0.6264) and 0.7092 times lower odds of CKD (OR 0.7092; 95% CI 0.6188, 0.8129) (Supplementary Table S8; Supplementary Figure S1). All sensitivity analyses in the MR results were robust, and there was no heterogeneity (Supplementary Table S9) and no pleiotropy (Supplementary Table S10) between all exposed genetic instrumental variables and DKD and CKD.

### Effect of education on each mediator

Using two-sample UVMR analyses, the causal effects of education on each mediator were evaluated. Supplementary Table S2 contains particular information about the genetic instruments that we use. Education with 26 candidate mediators did UVMR analyses, which showed that a total of 23 candidate mediators had statistically significant MR results. The MR results showed that each 1-SD longer years of schooling was associated with lower BMI (β −0.2942 SD; 95% CI −0.3187, −0.2698), lower BF% (β −0.1606 SD; 95% CI −0.2179, −0.1033), lower WHR (β −0.2157 SD; 95% CI −0.2670, −0.1643), higher HC (β 0.1086 SD; 95% CI 0.0634, 0.1539), lower cigarettes smoked per day (β −0.2799 SD; 95% CI −0.3314, −0.2285), lower pack years of smoking (β −0.3202 SD; 95% CI −0.3528, −0.2877), a decreased risk of maternal smoking around birth (OR 0.8879; 95% CI 0.8782, 0.8978), a decreased risk of smoking initiation (OR 0.6928; 95% CI 0.6681, 0.7185), higher age of smoking initiation (β 0.2777 SD; 95% CI 0.2528, 0.3027), higher coffee intake (β 0.0883 SD; 95% CI 0.0732, 0.1033), lower SBP (β −2.8239 SD; 95% CI −3.1674, −2.4803), lower DBP (β −1.1212 SD; 95% CI −1.3088, −0.9336), lower TG (β −0.1426 SD; 95% CI −0.1876, −0.0977), lower TC (β −0.0801 SD; 95% CI −0.1305, −0.0298), higher HDL-C (β 0.1109 SD; 95% CI 0.0634,0.1583), lower LDL-C (β −0.0938 SD; 95% CI −0.1448, −0.0429), lower fasting glucose (β −0.0917 SD; 95% CI −0.1457, −0.0378), lower fasting insulin (β −0.0717 SD; 95% CI −0.1227, −0.0206), lower MVPA (β −0.1059 SD; 95% CI −0.1264, −0.0854), higher VPA (β 0.0278 SD; 95% CI 0.0157, 0.0399), higher sedentary behavior (β 0.6082 SD; 95% CI 0.5647, 0.6517), an increased risk of household income (OR 1.8513; 95% CI 1.8053, 1.8985) and a decreased risk of T2D (OR 0.6982; 95% CI 0.6414, 0.7600). At least 4 sensitivity analyses confirmed these IVW estimates (Supplementary Table S11; Supplementary Figure S2). Genetic instrumental variables for education showed little heterogeneity and little pleiotropy with the mediator variables, except for sensitivity analyses between the instrumental variables for education and fasting insulin, which showed pleiotropy and was mainly driven by horizontal pleiotropy (Supplementary Tables S12, S13).

### Effect of each mediator on DKD without adjustment for education

Utilizing two-sample UVMR analyses, the causal effects of each mediator on DKD without controlling for education were assessed. Supplementary Table S3 contains particular information on the genetic instruments that we apply. Using the UVMR, the causal effect of 26 candidate mediators on DKD without adjusting for education was assessed and 14 mediators were finally selected for further MVMR analysis. Each 1-SD unit higher BMI (OR 2.4821; 95% CI 2.1981, 2.8027); BF% (OR 4.6427; 95% CI 2.5386, 8.4905); WHR (OR 3.3604; 95% CI 2.4326, 4.6419); coffee intake (OR 4.4362; 95% CI 2.5286, 7.7828); SBP (OR 1.0221; 95% CI 1.0146, 1.0297); DBP (OR 1.0164; 95% CI 1.0036, 1.0294); fasting glucose (OR 1.7873; 95% CI 1.2249, 2.6079); fasting insulin (OR 6.7742; 95% CI 2.5748, 17.8222); T1D (OR 1.1818; 95% CI 1.1304, 1.2355) and T2D (OR 1.7828; 95% CI 1.6565, 1.9187) were associated with an increased risk of DKD without adjustment for education (Supplementary Table S14). By contrast, each 1-SD unit higher HC (OR 0.7434; 95% CI 0.6090, 0.9076), LDL-C (OR 0.8767; 95% CI 0.7935, 0.9686), MVPA (OR 0.4109; 95% CI 0.1797, 0.9393) and household income (OR 0.5512; 95% CI 0.3709, 0.8193) were associated with a decreased risk of DKD without adjustment for education (Supplementary Table S14; Supplementary Figure S3). The heterogeneity of IVW was tested using the Cochran Q test, and neither the Q statistic nor the *p*-value was significant (*p* > 0.05), suggesting that there was no evidence of heterogeneity of the effects of candidate mediators on DKD (Supplementary Table S15). The p-value for the intercept was not significant when pleiotropy was tested using the MR-Egger intercept term, indicating that directional pleiotropy is not an issue for these results (Supplementary Table S16).

### Effect of each mediator on DKD with adjustment for education

After removing the mediators of the weak instrumental variables, we ended up with 7 mediators with robust results. The MVMR results showed that each 1-SD unit higher BMI (OR 2.3677; 95% CI 2.0582, 2.7237); SBP (OR 1.0230; 95% CI 1.0147, 1.0313); DBP (OR 1.0143; 95% CI 1.0003, 1.0284); and T2D (OR 1.7275; 95% CI 1.6035, 1.8612) were associated with an increased risk of DKD after adjusting for education (Table 2). However, each 1-SD unit with higher WHR (OR 0.6326; 95% CI 0.4994, 0.8014), fasting glucose (OR 0.4478; 95% CI 0.2270, 0.8836), and fasting insulin (OR 0.4850; 95% CI 0.2661, 0.8842) were associated with a decreased risk of DKD after adjusting for education (Table 2). Moreover, sensitivity analyses showed that no horizontal pleiotropy affected the results, indicating that our MVMR results were robust (Table 2; Supplementary Table S17).

**Table 2:** MVMR assessing the causal association between each mediator and DKD with adjustment for education.

Mediator	Method	β (95% CI)	OR (95% CI)	P_MVMR	Q_statistic	P_ Q_statistic	Egger_intercept	P_egger_intercept
BMI	MV-IVW	0.8619 (0.7218, 1.0020)	2.3677 (2.0582, 2.7237)	1.74E-33	1276.46	5.16E-09	−0.0015	2.91E-01
	MVMR-Egger	0.8451 (0.7022, 0.9880)	2.3283 (2.0183, 2.6859)	4.51E-31	1274.69	5.54E-09		
WHR	MV-IVW	−0.4579 (−0.6944, −0.2214)	0.6326 (0.4994, 0.8014)	1.48E-04	403.63	1.33E-02	0.0034	5.08E-01
	MVMR-Egger	−0.7216 (−1.5375, 0.0944)	0.4860 (0.2149, 1.0990)	8.30E-02	403.11	1.27E-02		
SBP	MV-IVW	0.0227 (0.0146, 0.0308)	1.0230 (1.0147, 1.0313)	3.91E-08	1147.07	5.88E-07	0.0029	5.16E-02
	MVMR-Egger	0.0230 (0.0149, 0.0311)	1.0233 (1.0150, 1.0316)	2.45E-08	1141.27	9.45E-07		
DBP	MV-IVW	0.0142 (0.0003, 0.0280)	1.0143 (1.0003, 1.0284)	4.55E-02	1197.71	2.02E-08	−0.0026	7.28E-02
	MVMR-Egger	0.0137 (−0.0002, 0.0276)	1.0138 (0.9998, 1.0280)	5.35E-02	1193.44	2.87E-08		
Fasting glucose	MV-IVW	−0.8033 (−1.4828, −0.1238)	0.4478 (0.2270, 0.8836)	2.05E-02	118.88	4.25E-04	−0.0063	3.84E-01
	MVMR-Egger	−0.3279 (−1.5965, 0.9406)	0.7204 (0.2026, 2.5615)	6.12E-01	117.63	4.23E-04		
Fasting insulin	MV-IVW	−0.7235 (−1.3239, −0.1231)	0.4850 (0.2661, 0.8842)	1.82E-02	73.26	7.22E-02	0.0045	6.02E-01
	MVMR-Egger	−1.0557 (−2.4414, 0.3300)	0.3479 (0.0870, 1.3910)	1.35E-01	72.90	6.41E-02		
T2D	MV-IVW	0.5467 (0.4722, 0.6212)	1.7275 (1.6035, 1.8612)	7.37E-47	503.19	1.55E-02	−0.0023	2.37E-01
	MVMR-Egger	0.5872 (0.4869, 0.6875)	1.7989 (1.6272, 1.9887)	1.76E-30	501.58	1.61E-02		

Effects of genetically predicted body mass index (BMI), waist-to-hip ratio (WHR), systolic blood pressure (SBP), diastolic blood pressure (DBP), fasting glucose, fasting insulin, and type 2 diabetes (T2D), respectively, on risk of diabetic kidney disease (DKD), after adjusting for education. Multivariable inverse variance weighted (MV-IVW) and multivariable Mendelian randomization Egger (MVMR-Egger) represent different Mendelian randomization models. OR indicates odds ratio; CI, confidence interval.

### Mediating effects of mediators in the association between education and DKD

**Figure 3:**
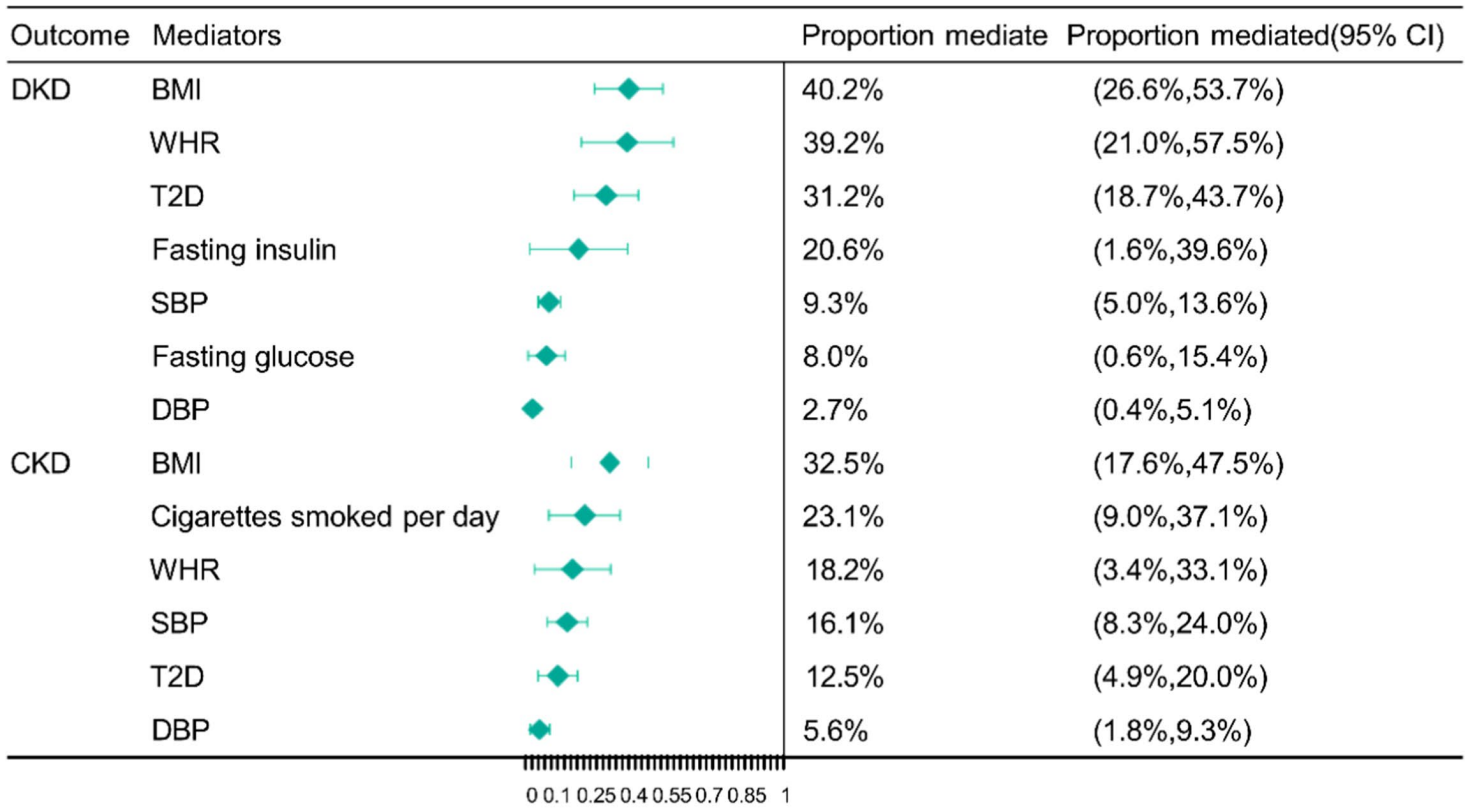
Mendelian randomization (MR) estimates of proportions mediated by mediators in the causal association between education and DKD as well as CKD. BMI, body mass index; WHR, waist-to-hip ratio; SBP, systolic blood pressure; DBP, diastolic blood pressure; T2D, type 2 diabetes; DKD, diabetic kidney disease; CKD, chronic kidney disease; CI, confidence interval.

Following the MVMR analyses mentioned above, we managed to eliminate the bias brought on by weak instrumental variables allowing us to obtain 7 mediators with robust results. These mediators consist of the adiposity trait (BMI and WHR), blood pressure trait (SBP and DBP), glucose metabolism-related trait (fasting glucose and fasting insulin), and diabetes (T2D), which are major factors in the causal pathway of education on DKD. The largest mediator of the causal effect from education to DKD was BMI (40.2%; 95% CI 26.6, 53.7%), followed by WHR (39.2%; 95% CI 21.0, 57.5%), T2D (31.2%; 95% CI 18.7, 43.7%), fasting insulin (20.6%; 95% CI 1.6, 39.6%), SBP (9.3%; 95% CI 5.0, 13.6%), fasting glucose (8.0%; 95% CI 0.6, 15.4%) and DBP (2.7%; 95% CI 0.4, 5.1%) (Figure 3).

### Effect of each mediator on CKD without adjustment for education

Two-sample UVMR analyses were used to explore the causal effects of each mediator on CKD without adjusting for education. Supplementary Table S4 provides comprehensive details regarding the genetic instruments we employ. The causal effect of the 26 candidate mediators on CKD without adjusting for education was assessed using UVMR, and 14 mediators were screened for subsequent MVMR analyses. Each 1-SD unit higher BMI (OR 1.4623; 95% CI 1.3460, 1.5887); BF% (OR 2.2205; 95% CI 1.2081, 4.0815); WHR (OR 1.3368; 95% CI 1.1003, 1.6241); cigarettes smoked per day (OR 1.3273; 95% CI 1.1767, 1.4970); pack years of smoking (OR 1.7019; 95% CI 1.2142, 2.3854); maternal smoking around birth (OR 7.5675; 95% CI 1.4536, 39.3967); SBP (OR 1.0198; 95% CI 1.0147, 1.0249); DBP (OR 1.0172; 95% CI 1.0082, 1.0263); TG (OR 1.0955; 95% CI 1.0014, 1.1985); fasting glucose (OR 1.4033; 95% CI 1.0934, 1.8012); T1D (OR 1.0536; 95% CI 1.0372, 1.0702); and T2D (OR 1.1267; 95% CI 1.0752, 1.1805) were associated with an increased risk of CKD without adjustment for education (Supplementary Table S18). By contrast, each 1-SD unit with higher TC (OR 0.8961; 95% CI 0.8321, 0.9651) and household income (OR 0.6017; 95% CI 0.4533, 0.7988) were associated with a decreased risk of CKD without adjustment for education (Supplementary Table S18; Supplementary Figure S4). In addition, both heterogeneity and pleiotropy were not detected by the sensitivity analyses, indicating that the MR results were reliable (Supplementary Tables S19, S20).

### Effect of each mediator on CKD with adjustment for education

After removing the mediators of the weak instrumental variables, we finally obtained 6 mediators with robust results, and the MVMR results showed that each 1-SD unit higher BMI (OR 1.4170; 95% CI 1.2906, 1.5559); SBP (OR 1.0207; 95% CI 1.0151, 1.0263); DBP (OR 1.0221; 95% CI 1.0123, 1.0320); and T2D (OR 1.1440; 95% CI 1.0911, 1.1995) were associated with an increased risk of CKD after adjusting for education (Table 3). However, each 1-SD unit with higher WHR (OR 0.7081; 95% CI 0.6043, 0.8298) and cigarettes smoked per day (OR 0.7233; 95% CI 0.6245, 0.8377) was associated with a decreased risk of CKD after adjusting for education (Table 3). Moreover, sensitivity analyses showed that no horizontal pleiotropy affected the results, indicating that our MVMR results were robust (Table 3; Supplementary Table S21).

**Table 3:** MVMR assessing the causal association between each mediator and CKD with adjustment for education.

Mediator	Method	β (95% CI)	OR (95% CI)	P_MVMR	Q_statistic	P_ Q_statistic	Egger_intercept	P_egger_intercept
BMI	MV-IVW	0.3486 (0.2551, 0.4420)	1.4170 (1.2906, 1.5559)	2.67E-13	1236.24	4.58E-07	−0.0006	5.16E-01
	MVMR-Egger	0.3343 (0.2393, 0.4292)	1.3969 (1.2704, 1.5361)	5.24E-12	1225.93	1.12E-06		
WHR	MV-IVW	−0.3451 (−0.5037, −0.1866)	0.7081 (0.6043, 0.8298)	1.99E-05	394.71	3.07E-02	0.0024	4.85E-01
	MVMR-Egger	−0.5315 (−1.0783, 0.0154)	0.5877 (0.3402, 1.0155)	5.68E-02	394.15	2.95E-02		
Cigarettes smoked per day	MV-IVW	−0.3239 (−0.4707, −0.1771)	0.7233 (0.6245, 0.8377)	1.53E-05	494.86	7.49E-03	0.0054	9.56E-02
	MVMR-Egger	−0.7209 (−1.2102, −0.2316)	0.4863 (0.2981, 0.7933)	3.88E-03	491.6	9.01E-03		
SBP	MV-IVW	0.0205 (0.0150, 0.0259)	1.0207 (1.0151, 1.0263)	1.90E-13	1138.49	2.39E-06	0.0018	7.50E-02
	MVMR-Egger	0.0206 (0.0151, 0.0260)	1.0208 (1.0153, 1.0264)	1.31E-13	1134.51	3.16E-06		
DBP	MV-IVW	0.0219 (0.0122, 0.0315)	1.0221 (1.0123, 1.0320)	8.58E-06	1262.16	1.75E-11	0.0002	8.66E-01
	MVMR-Egger	0.0215 (0.0119, 0.0312)	1.0218 (1.0120, 1.0317)	1.21E-05	1259.48	2.13E-11		
T2D	MV-IVW	0.1345 (0.0872, 0.1819)	1.1440 (1.0911, 1.1995)	2.54E-08	518.39	5.31E-03	−0.0007	6.02E-01
	MVMR-Egger	0.1450 (0.0834, 0.2067)	1.1561 (1.0869, 1.2297)	4.06E-06	518.06	4.97E-03		

Effects of genetically predicted body mass index (BMI), waist-to-hip ratio (WHR), Cigarettes smoked per day, systolic blood pressure (SBP), diastolic blood pressure (DBP) and type 2 diabetes (T2D), respectively, on risk of chronic kidney disease (CKD), after adjusting for education. Multivariable inverse variance weighted (MV-IVW) and multivariable Mendelian randomization Egger (MVMR-Egger) represent different Mendelian randomization models. OR indicates odds ratio; CI, confidence interval.

### Mediating effects of mediators in the association between education and CKD

We finally obtained 6 mediators with robust results, the adiposity trait (BMI and WHR), smoking (cigarettes smoked per day), blood pressure trait (SBP and DBP), and diabetes (T2D). These mediators are the most important factors in the association between education and CKD. The largest mediator from education to CKD was BMI (32.5%; 95% CI 17.6, 47.5%), followed by cigarettes smoked per day (23.1%; 95% CI 9.0, 37.1%), WHR (18.2%; 95% CI 3.4, 33.1%), and SBP (16.1%; 95% CI 8.3, 24.0%), T2D (12.5%; 95% CI 4.9, 20.0%) and DBP (5.6%; 95% CI 1.8, 9.3%) (Figure 3).

### Genetic correlation between exposure, mediators, and outcomes

LDSC regression analysis was employed to assess the genetic correlations between education, mediators, and DKD as well as CKD (Table 4). Negative genetic correlations were found between education and DKD (Rg = −0.2348, *p* = 8.23 × 10^−11^); CKD (Rg = −0.2655, *p* = 4.71 × 10^−8^); BMI (Rg = −0.2687, *p* = 4.19 × 10^−79^); WHR (Rg = −0.2687, *p* = 4.94 × 10^−22^); SBP (Rg = −0.1166, *p* = 1.30 × 10^−18^); DBP (Rg = −0.0827, *p* = 1.45 × 10^−9^); fasting glucose (Rg = −0.1029, *p* = 1.49 × 10^−2^); fasting insulin (Rg = −0.1344, *p* = 2.39 × 10^−3^); cigarettes smoked per day (Rg = −0.2928, *p* = 5.71 × 10^−42^); and T2D (Rg = −0.2575, *p* = 1.11 × 10^−36^). Positive genetic correlations were found between BMI (Rg = 0.4628, *p* = 1.91 × 10^−31^); WHR (Rg = 0.4130, *p* = 3.75 × 10^−9^); SBP (Rg = 0.2027, *p* = 5.15 × 10^−7^); DBP (Rg = 0.1442, *p* = 2.43 × 10^−4^); fasting glucose (Rg = 0.6635, *p* = 8.71 × 10^−7^); fasting insulin (Rg = 0.5784, *p* = 4.51 × 10^−4^); T2D (Rg = 0.7300, *p* = 2.80 × 10^−31^) and DKD. Significant positive genetic correlations were identified in the examination of the genetic association between mediators and CKD. Positive genetic correlations between mediators and CKD were discovered using LDSC regression analysis in BMI (Rg = 0.4006, *p* = 6.89 × 10^−16^), WHR (Rg = 0.4767, *p* = 1.43 × 10^−8^), SBP (Rg = 0.3370, *p* = 7.62 × 10^−9^), DBP (Rg = 0.2599, *p* = 4.25 × 10^−5^), and T2D (Rg = 0.5543, *p* = 1.27 × 10^−17^). Regretfully, no significant genetic correlation was found between cigarettes smoked per day (Rg = 0.1147, *p* = 9.08 × 10^−2^) and CKD.

**Table 4:** Genetic correlation estimates from LDSC regression between exposure, mediators, and outcomes.

Exposure	Outcome	Rg (SE)	*p*-value
Education	DKD	−0.2348 (0.0361)	8.23E-11
Education	CKD	−0.2655 (0.0486)	4.71E-08
Education	BMI	−0.2687 (0.0143)	4.19E-79
Education	WHR	−0.2687 (0.0278)	4.94E-22
Education	SBP	−0.1166 (0.0132)	1.30E-18
Education	DBP	−0.0827 (0.0137)	1.45E-09
Education	Fasting glucose	−0.1029 (0.0423)	1.49E-02
Education	Fasting insulin	−0.1344 (0.0443)	2.39E-03
Education	Cigarettes smoked per day	−0.2928 (0.0216)	5.71E-42
Education	T2D	−0.2575 (0.0204)	1.11E-36
BMI	DKD	0.4628 (0.0397)	1.91E-31
WHR	DKD	0.4130 (0.0701)	3.75E-09
SBP	DKD	0.2027 (0.0404)	5.15E-07
DBP	DKD	0.1442 (0.0393)	2.43E-04
Fasting glucose	DKD	0.6635 (0.1349)	8.71E-07
Fasting insulin	DKD	0.5784 (0.1649)	4.51E-04
T2D	DKD	0.7300 (0.0628)	2.80E-31
BMI	CKD	0.4006 (0.0496)	6.89E-16
WHR	CKD	0.4767 (0.0841)	1.43E-08
Cigarettes smoked per day	CKD	0.1147 (0.0678)	9.08E-02
SBP	CKD	0.3370 (0.0583)	7.62E-09
DBP	CKD	0.2599 (0.0635)	4.25E-05
T2D	CKD	0.5543 (0.0649)	1.27E-17

BMI, body mass index; WHR, waist-to-hip ratio; SBP, systolic blood pressure; DBP, diastolic blood pressure; T2D, type 2 diabetes; LDSC, linkage disequilibrium score; Rg, genetic correlation; SE, the standard error of Rg.

### Potential mediator interactions identified by UVMR analyses

To identify the key mediators and subsequently guide clinical decision-making, we employ UVMR analysis to investigate the intricate relationships between the mediators that have been screened. UVMR results showed that each 1-SD higher BMI was associated with higher WHR (β 0.3665 SD; 95% CI 0.3373, 0.3958), higher fasting glucose (β 0.0699 SD; 95% CI 0.0475, 0.0924), lower fasting insulin (β −0.0267 SD; 95% CI −0.0510, −0.0025), higher cigarettes smoked per day (β 0.3356 SD; 95% CI 0.2991, 0.3721), and a increased risk of T2D (OR 2.7189; 95% CI 2.5601, 2.8875); each 1-SD higher WHR was associated with higher fasting glucose (β 0.0869 SD; 95% CI 0.0551, 0.1186), higher fasting insulin (β 0.1331 SD; 95% CI 0.0493, 0.2170), higher SBP (β 1.4421 SD; 95% CI 0.0670, 2.8173), and a increased risk of T2D (OR 1.4388; 95% CI 1.0890, 1.9011); each 1-SD higher SBP was associated with higher cigarettes smoked per day (β 0.0026 SD; 95% CI 0.0006, 0.0047); each 1-SD higher DBP was associated with a increased risk of T2D (OR 1.0168; 95% CI 1.0096, 1.0241); each 1-SD higher fasting insulin was associated with higher fasting glucose (β 0.2934 SD; 95% CI 0.1878, 0.3989), lower BMI (β −0.2407 SD; 95% CI −0.4066, −0.0749), and a increased risk of T2D (OR 5.0216; 95% CI 1.8095, 13.9356); each 1-SD higher T2D was associated with higher fasting glucose (β 0.0680 SD; 95% CI 0.0506, 0.0853) and higher SBP (β 0.4566 SD; 95% CI 0.2516, 0.6617) (Table 5). Moreover, sensitivity analyses showed that no horizontal pleiotropy affected the results, indicating that our UVMR results were robust (Table 5).

**Table 5:** UVMR assessing the causal association among mediators.

Exposure	Outcome	Method	β (95% CI)	OR (95% CI)	P_ivw	Q_statistic	P_Q_statistic	Egger_intercept	P_egger_intercept
BMI	T2D	IVW	1.0002 (0.9400, 1.0604)	2.7189 (2.5601, 2.8875)	7.83E-233	1826.02	1.77E-74	0.0006	6.84E-01
BMI	WHR	IVW	0.3665 (0.3373, 0.3958)	1.4427 (1.4011, 1.4855)	2.54E-133	1295.71	6.00E-15	0.0002	8.22E-01
BMI	Fasting glucose	IVW	0.0699 (0.0475, 0.0924)	1.0724 (1.0486, 1.0968)	9.87E-10	459.93	1.77E-11	−0.0004	3.70E-01
BMI	Fasting insulin	IVW	−0.0267 (−0.0510, −0.0025)	0.9736 (0.9503, 0.9975)	3.07E-02	564.92	7.14E-22	0.0003	5.44E-01
BMI	Cigarettes smoked per day	IVW	0.3356 (0.2991, 0.3721)	1.3988 (1.3486, 1.4508)	1.85E-72	1655.38	3.55E-42	−0.0002	7.73E-01
WHR	T2D	IVW	0.3638 (0.0853, 0.6424)	1.4388 (1.0890, 1.9011)	1.05E-02	78.27	1.44E-10	0.0460	1.46E-01
WHR	Fasting glucose	IVW	0.0869 (0.0551, 0.1186)	1.0907 (1.0567, 1.1259)	7.95E-08	14.40	5.69E-01	−0.0038	1.64E-01
WHR	Fasting insulin	IVW	0.1331 (0.0493, 0.2170)	1.1424 (1.0505, 1.2424)	1.86E-03	77.20	9.31E-11	−0.0011	8.84E-01
WHR	SBP	IVW	1.4421 (0.0670, 2.8173)	4.2298 (1.0693, 16.7311)	3.98E-02	201.09	2.07E-32	0.0910	5.16E-01
SBP	Cigarettes smoked per day	IVW	0.0026 (0.0006, 0.0047)	1.0026 (1.0006, 1.0047)	1.02E-02	1172.78	1.40E-20	0.0008	3.10E-01
DBP	T2D	IVW	0.0167 (0.0096, 0.0238)	1.0168 (1.0096, 1.0241)	4.35E-06	1588.17	2.45E-90	0.0017	2.70E-01
Fasting insulin	T2D	IVW	1.6138 (0.5931, 2.6344)	5.0216 (1.8095, 13.9356)	1.94E-03	27.34	4.90E-05	−0.0353	5.16E-01
Fasting insulin	Fasting glucose	IVW	0.2934 (0.1878, 0.3989)	1.3409 (1.2066, 1.4901)	5.07E-08	16.81	1.14E-01	0.0015	8.10E-01
Fasting insulin	BMI	IVW	−0.2407 (−0.4066, −0.0749)	0.786 (0.6659, 0.9278)	4.44E-03	36.30	1.55E-05	−0.0089	2.17E-01
T2D	Fasting glucose	IVW	0.0680 (0.0506, 0.0853)	1.0703 (1.0519, 1.0891)	1.64E-14	194.67	2.30E-15	0.0016	4.09E-01
T2D	SBP	IVW	0.4566 (0.2516, 0.6617)	1.5788 (1.2860, 1.9381)	1.28E-05	552.97	8.01E-59	0.0072	6.73E-01

BMI, body mass index; WHR, waist-to-hip ratio; SBP, systolic blood pressure; DBP, diastolic blood pressure; T2D, type 2 diabetes.

### Identification of shared susceptibility genes for mediators and DKD as well as CKD

**Figure 4:**
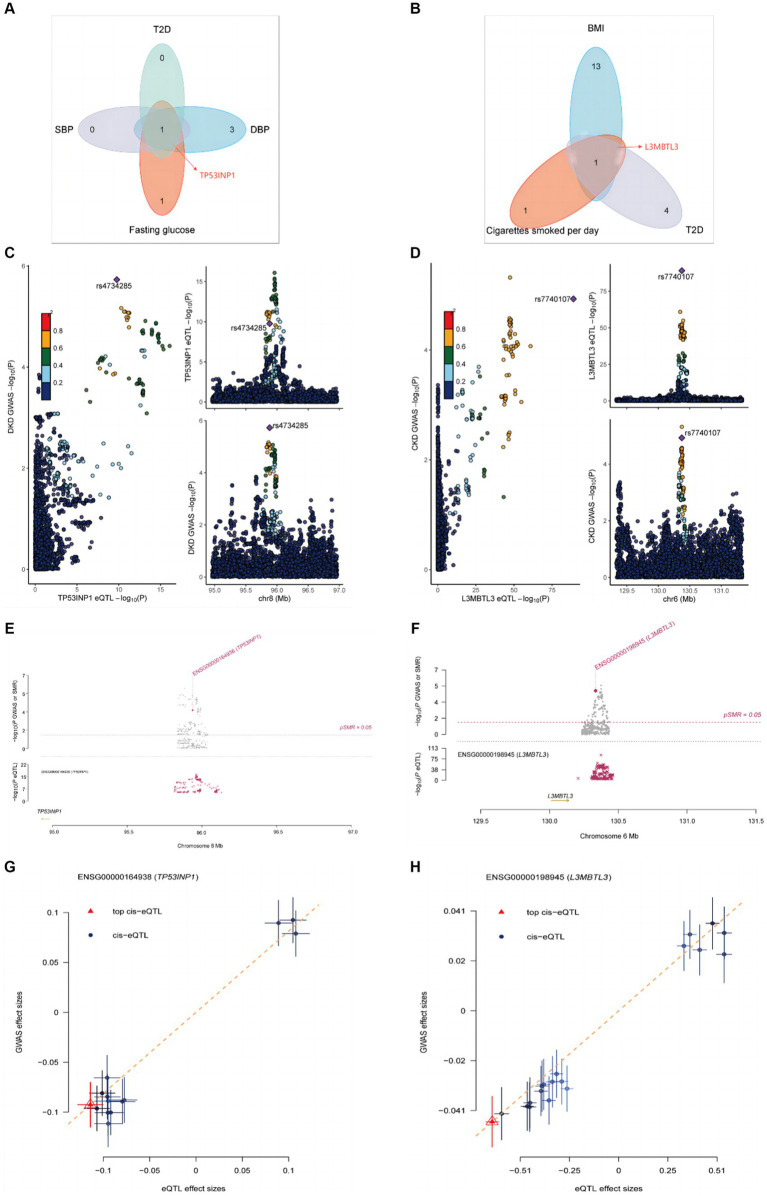
Colocalization and SMR analyses prioritized shared genes and DKD as well as CKD. **(A)** Hub gene transcripts analysis associated with mediators and DKD. **(B)** Hub gene transcripts analysis associated with mediators and CKD. **(C)** Colocalization analysis of TP53INP1 with DKD. **(D)** Colocalization analysis of L3MBTL3 with CKD. **(E)** Locus zoom plots showing the genetic effects between TP53INP1 and DKD. **(F)** Locus zoom plots showing the genetic effects between L3MBTL3 and CKD. **(G)** Three-step SMR indicating significant causal relationships between gene expressions and DKD onset (all three-step SMR *p* < 0.05, HEIDI test *p* > 0.05). **(H)** Three-step SMR indicating significant causal relationships between gene expressions and CKD onset (all three-step SMR *p* < 0.05, HEIDI test *p* > 0.05).

To explore the underlying molecular mechanisms, we used the TWAS approach to identify a series of genes that are commonly expressed in education, mediators, and kidney diseases (Supplementary Tables S22, S23). It is noteworthy that TP53INP1 is the common susceptibility gene for fasting glucose, T2D, SBP, DBP, and DKD (Supplementary Table S22; Figure 4A), while BMI, cigarettes smoked per day, T2D, and CKD are shared susceptibility genes for L3MBTL3 (Supplementary Table S22; Figure 4B). To find out if the GWAS variations in proteins are what cause the links between kidney diseases and proteins, we subsequently conducted colocalization analyses. The causal relationship between TP53INP1 and DKD was supported by the significant evidence of colocalization between TP53INP1 and DKD at rs4734285 (PPH4 = 0.8829) (Figure 4C). The causal relationship between L3MBTL3 and CKD was supported by the significant evidence of colocalization between L3MBTL3 and CKD at rs7740107 (PPH4 = 0.9715) (Figure 4D). To confirm the findings, we performed SMR analysis and the HEIDI test in later testing. Both the SMR analysis and the HEIDI test were passed by TP53INP1 (Psmr <0.05 and PHEIDI >0.05) and L3MBTL3 (Psmr <0.05 and PHEIDI >0.05) (Figures 4E–H).

## Discussion

The preventive effects of education on DKD and CKD are strongly supported by our MR findings. The incidence of developing DKD or CKD was found to be reduced by 48.64 and 29.08%, respectively, at an in-depth level of MR analysis for each additional 4.2 years of education. In order to explore the mechanisms by which education reduces the chance of getting DKD and CKD, we identified 26 cardiometabolic traits as potential mediators. Following the comprehensive screening, we identified 7 and 6 causal mediators, respectively, by which education generates a causal effect on DKD and CKD. Among the mediating effects between education and DKD, the largest mediator was BMI (40.2%), followed by its sub-WHR (39.2%), T2D (31.2%), fasting insulin (20.6%), SBP (9.3%), fasting glucose (8.0%) and DBP (2.7%), respectively. In contrast, potential mediators in the pathway from education to CKD, in descending order of mediator proportions, included BMI (32.5%), followed by cigarettes smoked per day (23.1%), WHR (18.2%), SBP (16.1%), T2D (12.5%) and DBP (5.6%). From our MR analysis, we revealed the protective effect of education on DKD as well as CKD. Common cardiometabolic traits (mainly adiposity trait, T2D, glucose metabolism-related trait, and blood pressure trait) were also explored to play an important role in the pathway from education to DKD. Moreover, similar findings are presented concerning the causal mediation from education to CKD. Interestingly, mediator interaction analysis revealed a strong causal relationship between BMI, WHR, and other cardiometabolic traits. This suggests that obesity is central to the estimation of clinical decision-making and should be prioritized when determining the direction of interventions. Moreover, it was shown that increased whole blood expression of TP53INP1 and L3MBTL3 was associated with an increased risk of kidney disease; TWAS, SMR, and colocalization analysis corroborated this finding.

In this study, UVMR found no causal association between genetically determined education and alcoholic drinking and T1D, suggesting that significant associations found in observational studies (38, 39) may be partially affected by confounders or reverse causality bias. On the one hand, WC, smoking trait, TG, TC, HDL-C, VPA, and sedentary behavior were excluded when UVMR was used to explore the causal effect of candidate mediators on DKD, a discrepancy that may be attributed to the limitations of traditional observational studies in identifying causal effects. It is worth noting that smoking may increase the risk of DKD through the metabolic effects of smoking as well as inflammation and endothelial dysfunction, but this relationship was more pronounced in patients with T1D (40). Although we used the most recent GWAS data from the FinnGen study database, we could not clearly distinguish whether DKD was from T1D or T2D and look forward to future sizable RCT (randomized controlled trial) studies to further explore this ambiguous association. In addition, an MR analysis study showed that genetically predicted lipid traits were associated with renal function in African populations (41), however, the data we used for the mediator analysis were from European populations, and the effect of lipid traits on renal disease may vary between different ethnic groups. On the other hand, when using UVMR to explore the causal effects of candidate mediators on CKD, HC, WC, smoking initiation, age of Smoking Initiation, coffee intake, HDL-C, LDL-C, fasting insulin, physical activity, or sedentary behavior were excluded due to non-significant *p*-values, and this discrepancy in results is since traditional observational studies have not done a good job of excluding confounders or the presence of reverse causality. Smoking initiation was defined as “ever” (current or former) and “never” are two phenotypes of regular smokers. Age of smoking initiation is a quasicontinuous variable that measures the age at which smokers begin to smoke regularly (42). It is striking that smoking initiation and age of smoking initiation were not associated with the onset of CKD, but cigarettes smoked per day significantly increased the risk of CKD, which may suggest that CKD is positively associated with the number of cigarettes smoked. In the mediation analyses between education and DKD, BF%, HC, and household income were excluded due to weak instrumental variable strengths or non-significance after adjusting for education because of their significant bidirectional causality with education, which was partially consistent with the mediation MR analyses of 1 MR study for the relationship of reverse causality between education and hypertension (43). When exploring education and CKD mediating risk factors, pack years of smoking and maternal smoking around birth were excluded due to bias from weak instrumental variables (29). After adjusting for education, BF%, TG, fasting glucose, and household income were no longer significant. The result was probably because, on the one hand, previous studies had found an inverse causal relationship between education and BF% and household income (43); on the other hand, TG and fasting glucose are significantly associated with the risk of developing CKD (44–46), but they do not act via the education-to-CKD pathway or the relationship between TG, fasting glucose and CKD is nonlinear (46).

Our findings in the mediating MR analysis of education for DKD showed that more than 20.6% of the total was mediated by BMI, WHR, T2D, and fasting insulin, with BMI accounting for as much as 40.2% of the mediating proportions. These results are consistent with previous observational epidemiological studies as well as MR analyses, in other words, obesity, T2D, and fasting insulin are closely associated with the risk of developing DKD or proteinuria (47–49). Regarding the mechanism of obesity on DKD, it is generally agreed that reactive oxygen and nitrogen species (ROS and RNS), redox processes, mitochondrial dysfunction, and adiponectin-5′-AMP activated protein kinase (AMPK) pathway are the core drivers between obesity and DKD (50, 51). Dihydroethidium fluorescence, a marker of *in vivo* superoxide generation, was surprisingly decreased in glomeruli and cortical tubules using both real-time measurements and confocal analysis, demonstrating lower superoxide production in the diabetic kidney compared to the control kidney (52). Electron paramagnetic resonance analysis of recently extracted renal cortex tissue revealed a comparable drop in superoxide when compared to the control kidney, in addition, the diabetic kidney’s mitochondria were observed to produce less hydrogen peroxide (50). Combining various methods led to the discovery that the kidney’s superoxide creation and the diabetic kidney’s mitochondria’s ROS production were decreased. Additionally, AMPK activation also significantly decreased glomerular transforming growth factor-beta (TGF-β), collagen, and fibronectin accumulation in several mouse models of diabetic kidney disease (53). The above results suggest that taking reasonable measures to lose weight, controlling fasting insulin, and even preventing the occurrence of T2D disease may produce a good reduction in the risk of DKD in some regions or populations with low education levels. Meanwhile, our results showed that SBP, fasting glucose, and DBP also played an important mediating role, and their mediating effects ranged from 2.7 to 9.3%, which is consistent with previous observational studies that blood pressure and fasting glucose increase the risk of DKD (49, 54). Vasoactive and AGE (Advanced glycation end-products) linkages connecting blood pressure, glucose, and DKD have been emphasized in recent research as contributors to renal impairment (55). Research employing an angiotensin type 2 (AT2) antagonist and studies in AT2 KO (knockout) mice revealed that suppressing AT2 in diabetes reduces macrovascular diseases (56). Additionally, prior research has indicated a connection between AT2 activation and apoptosis and antiproliferative and anti-inflammatory properties (57–59). A mineralocorticoid receptor (MR) blocker lowers the expression of monocyte chemoattractant protein 1 (MCP-1) upstream transcription factor NF-kB (nuclear factor-kappa B), renal macrophage infiltration, albuminuria, glomerulosclerosis, and MCP-1 in experimental models (60). The production of AGEs is accelerated by oxidative stress and persistent hyperglycemia (61, 62). In diabetes, advanced glycation modifies short-lived proteins and substantially alters long-lived proteins. Furthermore, the products of Kreb’s citric acid cycle and other glycolytic metabolites of glucose, such as glyoxal, are far more effective than glucose at initiating intracellular advanced glycation. The formation of Amadori products, the pentose phosphate pathway’s glyceraldehyde-3-phosphate, and the formation of the reactive carbonyl methylglyoxal—a dicarbonyl that was previously associated with diabetic complications—all result in complex biochemical reactions that give rise to AGE pathways, which are as diverse as their products (55).

After mediating effects for mediators of education and CKD were analyzed, we identified 6 mediators with significant results, of which BMI, cigarettes smoked per day, WHR, SBP, and T2D mediated greater than 12.5%, with BMI accounting for the largest mediator (32.5%) and DBP accounting for the smallest mediator (5.6%). The effects of BMI and WHR on CKD have been reported in observational studies. In univariate analysis, each 0.1 unit increase in WHR was associated with an 81% increase in CKD risk, while each 2 kg/m (2) increase in BMI was associated with an 11% increase in risk of developing incident CKD, but in multivariate analysis, each unit increase in WHR was still associated with an increased risk of CKD prevalence, and interestingly BMI in multivariate confounders such as potential age, sex, prior cardiovascular disease, diabetes, hypertension, and lipids were no longer significant after mutual correction (63). WHR reflects an indicator of the size of central adiposity, whereas BMI does not respond to the distributional characteristics of adiposity (64), which may be an important reason why the risk of CKD is mainly related to central obesity rather than peripheral obesity. The association of smoking, SBP, DBP, and T2D with CKD has been reported in a large number of observational studies (65–68), but evidence of causality is lacking. Cigarettes smoked per day accounted for 23.1% of the mediation in the education to CKD pathway, which may be because nicotine in cigarette smoke and reactive aldehydes by damaging endothelial cells, increasing reactive oxygen species production, and activating pro-fibrotic pathways (69, 70). The primary causes of hypertension and CKD are widely accepted to be the renin-angiotensin-aldosterone system (RAAS), endothelial dysfunction, and endoplasmic reticulum (ER) stress (71). Blood flow is diminished in peritubular capillaries located downstream of glomeruli that have sclerosed. Reduced effective (perceived) blood flow causes glomeruli in certain areas to hypersecrete renin, which raises the levels of angiotensin II in the blood (71). Due to its direct vasoconstrictor action, angiotensin II raises blood pressure and systemic vascular resistance. Endothelial dysfunction (including impaired nitrous oxide production), oxidative stress, and elevated endothelin levels are also implicated in the pathogenesis of hypertension in patients with CKD (72). Notably, obesity, blood pressure, glucose metabolism, and T2D are common disorders with significant public health implications, often occurring as co-morbidities and sharing common biological mechanisms including chronic inflammation, oxidative stress, and energy metabolism (73–75). The proportions of the mediators in our mediation analysis run at risk of overlapping since the mediators are inextricably linked.

Tumor protein p53 inducible nuclear protein 1 (TP53INP1) is involved in the regulation of N6-methyladenosine (M6A) methylation autophagy, apoptosis, and inflammation (76–78). M6A modification, apoptosis, and inflammation are key processes causing pathological damage in DKD (79, 80). Podocyte destruction is also significantly influenced by the intricate relationships among autophagy, apoptosis, and inflammatory regulation (51, 81). According to recent research, METTL3 induces apoptosis and inflammation by affecting TIMP2 mRNA m6A methylation (79). Additionally, FTO intronic transcript 1 (FTO-IT1) deletion elevated TP53INP1 mRNA m6A methylation, which consequently promoted apoptosis (76). The observed cell death following TP53INP1-LC3 interaction is dependent on both autophagy and caspase activity (77). Under oxidative stress conditions, P53 is regulated by TP53INP1, while P53 can positively regulate the expression of TP53INP1, so the two form a positive feedback loop (82). Through transcriptional activation, P53 can interact with the TP53INP1 gene’s promoter to boost the expression of TP53INP1 (82). One possible target for preventing oxidative stress-induced apoptosis is the TP53INP1-P53 positive feedback loop. In summary, we may conclude that TP53INP1 is essential for m6A methylation, autophagy, apoptosis, and oxidative stress. Given its predicted substantial effect on these critical pathways of podocyte damage in DKD, TP53INP1 may be provided as a possible target for therapy.

Nephron loss, inflammation, myofibroblast activation, and extracellular matrix (ECM) deposition are the hallmarks of CKD (83). The loss of the nephron, including the tubules, glomerulus, and endothelium, is caused by lipotoxicity and oxidative stress (83). Injured renal resident cells release proinflammatory cytokines and chemokines to recruit bone marrow-derived macrophages and other immune cells (84). Numerous profibrotic cytokines, including angiotensin II and TGF-β1, are secreted by injured renal resident cells and immune cells (83). TGF-β and Notch signaling facilitate myofibroblast activation and the production of ECM (85). In conclusion, Lipotoxicity, macrophages, and Notch signaling are critical to the pathogenic progression of CKD. Decreased peripheral fat depots, impaired adipogenesis, and ultimately elevated risk of kidney disease were linked to lethal (3) malignant brain tumor-like 3 (L3MBTL3) (86). Observational studies also revealed a significant association between fat depots and an increased chance of CKD (87, 88), resulting from a decreased ability for adipocyte differentiation triggered by L3MBTL3. The immune infiltration of macrophages and their polarization from M1 to M2 were notably related to L3MBTL3 (89). The recombining binding protein suppressor of hairless (RBPJ), a transcription factor, interacts with the intracellular domain of the Notch receptor and the coactivator mastermind to form an activation complex (90). In contrast to other RBPJ binding partners, L3MBTL3 exhibits a unique binding motif in its interaction with RBPJ to regulate the Notch pathway (90). In summary, L3MBTL3 contributes significantly to the critical pathway of CKD pathogenesis. However, further research is required to fully understand its specific function in CKD.

The large amount of GWAS data allowed us to explore causal associations between education, mediators, DKD, and CKD, and we set stringent criteria for the robustness of our results. First, the GWAS sources we chose for DKD and CKD were from the most recent FinnGen study, and there was little overlap between this data and the exposure and mediator’s GWAS data. Second, different assumptions were applied to different MR analysis methods, and the consistency of results from multiple MR analyses made our results more robust while selecting multiple sensitivity analyses such as MR-Egger intercept test, Cochran’s Q test, MR-PRESSO, RadialMR, and MR Steiger filtering. Finally, we set the criteria for mediator screening to exclude weak instrumental variables from interfering with the results, ensuring the credibility and plausibility of the model we construct to explain the mediation effect. In addition, we used LDSC to estimate whether the observed relationship is due to shared genetic background. TWAS, SMR, and colocalization analysis were used to find shared susceptibility genes among education, cardiometabolic traits, and kidney diseases. However, our findings have several important limitations to consider when interpreting the results. First, it is challenging to exclude the effect of numerous confounding factors from the relationship between CKD and education because CKD usually occurs later in life, several years after the completion of education. On the other hand, as educational attainment is a phenotype that can be genetically tracked and is a lifelong process, it has been suggested that MR analyses can show the causal relationship between educational attainment and complicated disorders (91, 92). Recent MR studies have shown that kidney damage causally influences the cortex structure (93), suggesting a strong link between kidney function and brain structure and education. Second, The GWAS data we used was from developed, high-income European populations, and the results of the MR analyses still need to be further validated in low-income country populations and other ethnic groups. Third, as our choice of candidate mediators was limited to cardiometabolic traits, this only partially explains the causal effect of education on DKD and CKD in clinical or public policy terms. Fourth, we used MR-PRESSO, RadialMR, and MR Steiger filtering to remove outliers and causally incorrect SNPs, but we could not exclude some mediators, such as poor areas and health policies, because these confounding factors do not have corresponding GWAS data and are non-heritable phenotypes. Fifth, sample overlap between GWAS studies may bias MR estimates toward observational association estimates (94). Sixth, GWAS research including higher sample sizes is necessary to reduce correlations across phenotypes because of the multicollinearity among cardiometabolic traits. Seventh, the function of susceptibility genes in kidney disease requires further research.

## Conclusion

Higher education has a protective effect on the risk of DKD and CKD, identifying the mediating effects of modifiable cardiometabolic traits on the causal relationship. The biggest mediator of the relationship between education and DKD was BMI (40.2%), which was then followed by its sub-WHR (39.2%), T2D (31.2%), fasting insulin (20.6%), SBP (9.3%), fasting glucose (8.0%), and DBP (2.7%). In descending order of mediator proportions, BMI (32.5%), cigarettes smoked per day (23.1%), WHR (18.2%), SBP (16.1%), T2D (12.5%), and DBP (5.6%) were potential mediators in the pathway from education to CKD. Furthermore, it was discovered that elevated whole blood levels of TP53INP1 and L3MBTL3 expression were related to a higher risk of kidney disease; TWAS, SMR, and colocalization analysis all supported this finding. Therefore, for individuals with limited access to educational resources, adopting strategies (including losing weight, controlling blood pressure, regulating fasting glucose as well as fasting insulin, quitting smoking, and targeting TP53INP1 and L3MBTL3) may prove effective in preventing DKD and CKD.

## Data availability statement

The original contributions presented in the study are included in the article/Supplementary material, further inquiries can be directed to the corresponding author.

## Author contributions

YuW: Conceptualization, Formal analysis, Methodology, Resources, Software, Visualization, Writing – original draft, Writing – review & editing. MC: Data curation, Formal analysis, Investigation, Methodology, Resources, Software, Validation, Writing – review & editing. LW: Data curation, Formal analysis, Methodology, Resources, Software, Writing – review & editing. YoW: Conceptualization, Project administration, Resources, Supervision, Writing – review & editing.

## References

[1] Duffield JS. Cellular and molecular mechanisms in kidney fibrosis. J Clin Invest. (2014) 124:2299–306. doi: 10.1172/jci72267 24892703 PMC4038570

[2] Forbes JM Cooper ME. Mechanisms of diabetic complications. Physiol Rev. (2013) 93:137–88. doi: 10.1152/physrev.00045.2011 23303908

[3] GBD Chronic Kidney Disease Collaboration. Global, regional, and national burden of chronic kidney disease, 1990–2017: a systematic analysis for the global burden of disease study 2017. Lancet. (2020) 395:709–33. doi: 10.1016/s0140-6736(20)30045-3, PMID: 32061315 PMC7049905

[4] Sun H Saeedi P Karuranga S Pinkepank M Ogurtsova K Duncan BB. IDF diabetes atlas: global, regional and country-level diabetes prevalence estimates for 2021 and projections for 2045. Diabetes Res Clin Pract. (2022) 183:109119. doi: 10.1016/j.diabres.2021.109119, PMID: 34879977 PMC11057359

[5] Thio CHL Vart P Kieneker LM Snieder H Gansevoort RT Bültmann U. Educational level and risk of chronic kidney disease: longitudinal data from the PREVEND study. Nephrol Dial Transplant. (2020) 35:1211–8. doi: 10.1093/ndt/gfy361, PMID: 30541108

[6] Blomster JI Zoungas S Woodward M Neal B Harrap S Poulter N. The impact of level of education on vascular events and mortality in patients with type 2 diabetes mellitus: results from the ADVANCE study. Diabetes Res Clin Pract. (2017) 127:212–7. doi: 10.1016/j.diabres.2017.03.015, PMID: 28395214

[7] Global Burden of Metabolic Risk Factors for Chronic Diseases Collaboration. Cardiovascular disease, chronic kidney disease, and diabetes mortality burden of cardiometabolic risk factors from 1980 to 2010: a comparative risk assessment. Lancet Diabetes Endocrinol. (2014) 2:634–47. doi: 10.1016/s2213-8587(14)70102-0 24842598 PMC4572741

[8] Halminen J Sattar N Rawshani A Eliasson B Eeg-Olofsson K Bhatt DL. Range of risk factor levels, risk control, and temporal trends for nephropathy and end-stage kidney disease in patients with type 1 and type 2 diabetes. Diabetes Care. (2022) 45:2326–35. doi: 10.2337/dc22-0926, PMID: 35984439

[9] Smith GD Ebrahim S. “Mendelian randomization”: can genetic epidemiology contribute to understanding environmental determinants of disease? Int J Epidemiol. (2003) 32:1–22. doi: 10.1093/ije/dyg070, PMID: 12689998

[10] Burgess S Thompson SG. Multivariable Mendelian randomization: the use of pleiotropic genetic variants to estimate causal effects. Am J Epidemiol. (2015) 181:251–60. doi: 10.1093/aje/kwu283 25632051 PMC4325677

[11] Sanderson E Davey Smith G Windmeijer F Bowden J. An examination of multivariable Mendelian randomization in the single-sample and two-sample summary data settings. Int J Epidemiol. (2019) 48:713–27. doi: 10.1093/ije/dyy262, PMID: 30535378 PMC6734942

[12] Sanderson E. Multivariable Mendelian randomization and mediation. Cold Spring Harb Perspect Med. (2021) 11:a038984. doi: 10.1101/cshperspect.a038984 32341063 PMC7849347

[13] Doke T Huang S Qiu C Liu H Guan Y Hu H. Transcriptome-wide association analysis identifies DACH1 as a kidney disease risk gene that contributes to fibrosis. J Clin Invest. (2021) 131:e141801. doi: 10.1172/JCI141801 33998598 PMC8121513

[14] Qiu C Huang S Park J Park YS Ko YA Seasock MJ. Renal compartment-specific genetic variation analyses identify new pathways in chronic kidney disease. Nat Med. (2018) 24:1721–31. doi: 10.1038/s41591-018-0194-4 30275566 PMC6301011

[15] Ko YA Yi H Qiu C Huang S Park J Ledo N. Genetic-variation-driven gene-expression changes highlight genes with important functions for kidney disease. Am J Hum Genet. (2017) 100:940–53. doi: 10.1016/j.ajhg.2017.05.004, PMID: 28575649 PMC5473735

[16] Zheng J Haberland V Baird D Walker V Haycock PC Hurle MR. Phenome-wide Mendelian randomization mapping the influence of the plasma proteome on complex diseases. Nat Genet. (2020) 52:1122–31. doi: 10.1038/s41588-020-0682-6, PMID: 32895551 PMC7610464

[17] Relton CL Davey SG. Two-step epigenetic Mendelian randomization: a strategy for establishing the causal role of epigenetic processes in pathways to disease. Int J Epidemiol. (2012) 41:161–76. doi: 10.1093/ije/dyr233, PMID: 22422451 PMC3304531

[18] Burgess S Daniel RM Butterworth AS Thompson SG. Network Mendelian randomization: using genetic variants as instrumental variables to investigate mediation in causal pathways. Int J Epidemiol. (2015) 44:484–95. doi: 10.1093/ije/dyu176, PMID: 25150977 PMC4469795

[19] Skrivankova VW Richmond RC Woolf BAR Yarmolinsky J Davies NM Swanson SA. Strengthening the reporting of observational studies in epidemiology using Mendelian randomization: the STROBE-MR statement. JAMA. (2021) 326:1614–21. doi: 10.1001/jama.2021.18236 34698778

[20] Skrivankova VW Richmond RC Woolf BAR Davies NM Swanson SA VanderWeele TJ. Strengthening the reporting of observational studies in epidemiology using mendelian randomisation (STROBE-MR): explanation and elaboration. BMJ. (2021) 375:n2233. doi: 10.1136/bmj.n2233, PMID: 34702754 PMC8546498

[21] Hemani G Zheng J Elsworth B Wade KH Haberland V Baird D. The MR-base platform supports systematic causal inference across the human phenome. eLife. (2018) 7:e34408. doi: 10.7554/eLife.34408, PMID: 29846171 PMC5976434

[22] Hemani G Bowden J Davey SG. Evaluating the potential role of pleiotropy in Mendelian randomization studies. Hum Mol Genet. (2018) 27:R195–r208. doi: 10.1093/hmg/ddy163, PMID: 29771313 PMC6061876

[23] Bowden J Davey Smith G Burgess S. Mendelian randomization with invalid instruments: effect estimation and bias detection through Egger regression. Int J Epidemiol. (2015) 44:512–25. doi: 10.1093/ije/dyv080 26050253 PMC4469799

[24] Bowden J Davey Smith G Haycock PC Burgess S. Consistent estimation in Mendelian randomization with some invalid instruments using a weighted median estimator. Genet Epidemiol. (2016) 40:304–14. doi: 10.1002/gepi.21965, PMID: 27061298 PMC4849733

[25] Xue H Shen X Pan W. Constrained maximum likelihood-based Mendelian randomization robust to both correlated and uncorrelated pleiotropic effects. Am J Hum Genet. (2021) 108:1251–69. doi: 10.1016/j.ajhg.2021.05.014, PMID: 34214446 PMC8322939

[26] Zhu Z Zheng Z Zhang F Wu Y Trzaskowski M Maier R. Causal associations between risk factors and common diseases inferred from GWAS summary data. Nat Commun. (2018) 9:224. doi: 10.1038/s41467-017-02317-2, PMID: 29335400 PMC5768719

[27] Yavorska OO Burgess S. MendelianRandomization: an R package for performing Mendelian randomization analyses using summarized data. Int J Epidemiol. (2017) 46:1734–9. doi: 10.1093/ije/dyx034, PMID: 28398548 PMC5510723

[28] Verbanck M Chen CY Neale B Do R. Detection of widespread horizontal pleiotropy in causal relationships inferred from Mendelian randomization between complex traits and diseases. Nat Genet. (2018) 50:693–8. doi: 10.1038/s41588-018-0099-7, PMID: 29686387 PMC6083837

[29] Burgess S Thompson SG. Avoiding bias from weak instruments in Mendelian randomization studies. Int J Epidemiol. (2011) 40:755–64. doi: 10.1093/ije/dyr036, PMID: 21414999

[30] Greco MF Minelli C Sheehan NA Thompson JR. Detecting pleiotropy in Mendelian randomisation studies with summary data and a continuous outcome. Stat Med. (2015) 34:2926–40. doi: 10.1002/sim.6522, PMID: 25950993

[31] Bowden J Spiller W Del Greco MF Sheehan N Thompson J Minelli C. Improving the visualization, interpretation and analysis of two-sample summary data Mendelian randomization via the radial plot and radial regression. Int J Epidemiol. (2018) 47:1264–78. doi: 10.1093/ije/dyy101, PMID: 29961852 PMC6124632

[32] Hemani G Tilling K Davey SG. Orienting the causal relationship between imprecisely measured traits using GWAS summary data. PLoS Genet. (2017) 13:e1007081. doi: 10.1371/journal.pgen.1007081, PMID: 29149188 PMC5711033

[33] Bulik-Sullivan BK Loh PR Finucane HK Ripke S Yang J Schizophrenia Working Group of the Psychiatric Genomics Consortium. LD score regression distinguishes confounding from polygenicity in genome-wide association studies. Nat Genet. (2015) 47:291–5. doi: 10.1038/ng.3211, PMID: 25642630 PMC4495769

[34] Zhang C Qin F Li X Du X Li T. Identification of novel proteins for lacunar stroke by integrating genome-wide association data and human brain proteomes. BMC Med. (2022) 20:211. doi: 10.1186/s12916-022-02408-y, PMID: 35733147 PMC9219149

[35] Huang Y Shan Y Zhang W Lee AM Li F Stranger BE. Deciphering genetic causes for sex differences in human health through drug metabolism and transporter genes. Nat Commun. (2023) 14:175. doi: 10.1038/s41467-023-35808-6, PMID: 36635277 PMC9837057

[36] Arvanitis M Tayeb K Strober BJ Battle A. Redefining tissue specificity of genetic regulation of gene expression in the presence of allelic heterogeneity. Am J Hum Genet. (2022) 109:223–39. doi: 10.1016/j.ajhg.2022.01.002, PMID: 35085493 PMC8874223

[37] Wu Y Zeng J Zhang F Zhu Z Qi T Zheng Z. Integrative analysis of omics summary data reveals putative mechanisms underlying complex traits. Nat Commun. (2018) 9:918. doi: 10.1038/s41467-018-03371-0, PMID: 29500431 PMC5834629

[38] Borschuk AP Everhart RS. Health disparities among youth with type 1 diabetes: a systematic review of the current literature. Fam Syst Health. (2015) 33:297–313. doi: 10.1037/fsh0000134, PMID: 25984737

[39] Shimotsu ST Jones-Webb RJ Lytle LA MacLehose RF Nelson TF Forster JL. The relationships among socioeconomic status, fruit and vegetable intake, and alcohol consumption. Am J Health Promot. (2012) 27:21–8. doi: 10.4278/ajhp.110311-QUAN-108 22950922

[40] Eliasson B. Cigarette smoking and diabetes. Prog Cardiovasc Dis. (2003) 45:405–13. doi: 10.1053/pcad.2003.00103 12704597

[41] Kintu C Soremekun O Kamiza AB Kalungi A Mayanja R Kalyesubula R. The causal effects of lipid traits on kidney function in Africans: bidirectional and multivariable Mendelian-randomization study. EBioMedicine. (2023) 90:104537. doi: 10.1016/j.ebiom.2023.104537, PMID: 37001235 PMC10070509

[42] Liu M Jiang Y Wedow R Li Y Brazel DM Chen F. Association studies of up to 1.2 million individuals yield new insights into the genetic etiology of tobacco and alcohol use. Nat Genet. (2019) 51:237–44. doi: 10.1038/s41588-018-0307-5, PMID: 30643251 PMC6358542

[43] Wang Y Ye C Kong L Zheng J Xu M Xu Y. Independent associations of education, intelligence, and cognition with hypertension and the mediating effects of Cardiometabolic risk factors: a Mendelian randomization study. Hypertension. (2023) 80:192–203. doi: 10.1161/hypertensionaha.122.20286 36353998 PMC9722390

[44] Chen J Muntner P Hamm LL Fonseca V Batuman V Whelton PK. Insulin resistance and risk of chronic kidney disease in nondiabetic US adults. J Am Soc Nephrol. (2003) 14:469–77. doi: 10.1097/01.asn.0000046029.53933.09, PMID: 12538749

[45] Lanktree MB Thériault S Walsh M Paré G. HDL cholesterol, LDL cholesterol, and triglycerides as risk factors for CKD: a Mendelian randomization study. Am J Kidney Dis. (2018) 71:166–72. doi: 10.1053/j.ajkd.2017.06.011, PMID: 28754456

[46] Zhu Q Chen Y Cai X Cai L Hong J Luo Q. The non-linear relationship between triglyceride-glucose index and risk of chronic kidney disease in hypertensive patients with abnormal glucose metabolism: a cohort study. Front Med (Lausanne). (2022) 9:1018083. doi: 10.3389/fmed.2022.1018083, PMID: 36203768 PMC9530361

[47] Geng T Zhu K Lu Q Wan Z Chen X Liu L. Healthy lifestyle behaviors, mediating biomarkers, and risk of microvascular complications among individuals with type 2 diabetes: a cohort study. PLoS Med. (2023) 20:e1004135. doi: 10.1371/journal.pmed.1004135, PMID: 36626356 PMC9831321

[48] Man REK Gan ATL Fenwick EK Gupta P Wong MYZ Wong TY. The relationship between generalized and abdominal obesity with diabetic kidney disease in type 2 diabetes: a multiethnic Asian study and Meta-analysis. Nutrients. (2018) 10:1685. doi: 10.3390/nu10111685 30400648 PMC6266073

[49] Schroijen MA de Mutsert R Dekker FW de Vries APJ de Koning EJP Rabelink TJ. The association of glucose metabolism and kidney function in middle-aged adults. Clin Kidney J. (2021) 14:2383–90. doi: 10.1093/ckj/sfab074, PMID: 34754434 PMC8572983

[50] Sharma K. Obesity and diabetic kidney disease: role of oxidant stress and redox balance. Antioxid Redox Signal. (2016) 25:208–16. doi: 10.1089/ars.2016.6696, PMID: 26983586 PMC4964755

[51] Liu XQ Jiang L Li YY Huang YB Hu XR Zhu W. Wogonin protects glomerular podocytes by targeting Bcl-2-mediated autophagy and apoptosis in diabetic kidney disease. Acta Pharmacol Sin. (2022) 43:96–110. doi: 10.1038/s41401-021-00721-5, PMID: 34253875 PMC8724322

[52] Dugan LL You YH Ali SS Diamond-Stanic M Miyamoto S DeCleves AE. AMPK dysregulation promotes diabetes-related reduction of superoxide and mitochondrial function. J Clin Invest. (2013) 123:4888–99. doi: 10.1172/jci66218, PMID: 24135141 PMC3809777

[53] Sharma K. Obesity, oxidative stress, and fibrosis in chronic kidney disease. Kidney Int Suppl (2011). (2014) 4:113–7. doi: 10.1038/kisup.2014.21, PMID: 25401040 PMC4220515

[54] De Cosmo S Viazzi F Piscitelli P Giorda C Ceriello A Genovese S. Blood pressure status and the incidence of diabetic kidney disease in patients with hypertension and type 2 diabetes. J Hypertens. (2016) 34:2090–8. doi: 10.1097/hjh.0000000000001045, PMID: 27457667

[55] Patel DM Bose M Cooper ME. Glucose and blood pressure-dependent pathways-the progression of diabetic kidney disease. Int J Mol Sci. (2020) 21:2218. doi: 10.3390/ijms21062218 32210089 PMC7139394

[56] Koïtka A Cao Z Koh P Watson AMD Sourris KC Loufrani L. Angiotensin II subtype 2 receptor blockade and deficiency attenuate the development of atherosclerosis in an apolipoprotein E-deficient mouse model of diabetes. Diabetologia. (2010) 53:584–92. doi: 10.1007/s00125-009-1619-x, PMID: 19957160

[57] Brassard P Amiri F Thibault G Schiffrin EL. Role of angiotensin type-1 and angiotensin type-2 receptors in the expression of vascular integrins in angiotensin II-infused rats. Hypertension. (2006) 47:122–7. doi: 10.1161/01.Hyp.0000196272.79321.11, PMID: 16330679

[58] Hu C Dandapat A Chen J Liu Y Hermonat PL Carey RM. Over-expression of angiotensin II type 2 receptor (agtr2) reduces atherogenesis and modulates LOX-1, endothelial nitric oxide synthase and heme-oxygenase-1 expression. Atherosclerosis. (2008) 199:288–94. doi: 10.1016/j.atherosclerosis.2007.11.006 18096165

[59] Savoia C Ebrahimian T He Y Gratton JP Schiffrin EL Touyz RM. Angiotensin II/AT2 receptor-induced vasodilation in stroke-prone spontaneously hypertensive rats involves nitric oxide and cGMP-dependent protein kinase. J Hypertens. (2006) 24:2417–22. doi: 10.1097/01.hjh.0000251902.85675.7e, PMID: 17082724

[60] Han SY Kim CH Kim HS Jee YH Song HK Lee MH. Spironolactone prevents diabetic nephropathy through an anti-inflammatory mechanism in type 2 diabetic rats. J Am Soc Nephrol. (2006) 17:1362–72. doi: 10.1681/asn.2005111196, PMID: 16571782

[61] Fu MX Wells-Knecht KJ Blackledge JA Lyons TJ Thorpe SR Baynes JW. Glycation, glycoxidation, and cross-linking of collagen by glucose. Kinetics, mechanisms, and inhibition of late stages of the Maillard reaction. Diabetes. (1994) 43:676–83. doi: 10.2337/diab.43.5.676, PMID: 8168645

[62] Vlassara H Uribarri J Cai W Goodman S Pyzik R Post J. Effects of sevelamer on HbA1c, inflammation, and advanced glycation end products in diabetic kidney disease. Clin J Am Soc Nephrol. (2012) 7:934–42. doi: 10.2215/cjn.12891211, PMID: 22461535 PMC3362316

[63] Elsayed EF Sarnak MJ Tighiouart H Griffith JL Kurth T Salem DN. Waist-to-hip ratio, body mass index, and subsequent kidney disease and death. Am J Kidney Dis. (2008) 52:29–38. doi: 10.1053/j.ajkd.2008.02.363, PMID: 18511168 PMC4052757

[64] Klein S Allison DB Heymsfield SB Kelley DE Leibel RL Nonas C. Waist circumference and cardiometabolic risk: a consensus statement from shaping America's health: Association for Weight Management and Obesity Prevention; NAASO, the Obesity Society; the American Society for Nutrition; and the American Diabetes Association. Am J Clin Nutr. (2007) 85:1197–202. doi: 10.1093/ajcn/85.5.1197 17490953

[65] Agarwal R. Blood pressure components and the risk for end-stage renal disease and death in chronic kidney disease. Clin J Am Soc Nephrol. (2009) 4:830–7. doi: 10.2215/cjn.06201208 19339424 PMC2666439

[66] Lou QL Ouyang XJ Gu LB Mo YZ Ma R Nan J. Chronic kidney disease and associated cardiovascular risk factors in chinese with type 2 diabetes. Diabetes Metab J. (2012) 36:433–42. doi: 10.4093/dmj.2012.36.6.433, PMID: 23275937 PMC3530714

[67] Orth SR Hallan SI. Smoking: a risk factor for progression of chronic kidney disease and for cardiovascular morbidity and mortality in renal patients--absence of evidence or evidence of absence? Clin J Am Soc Nephrol. (2008) 3:226–36. doi: 10.2215/cjn.03740907 18003763

[68] Schaeffner ES Kurth T Bowman TS Gelber RP Gaziano JM. Blood pressure measures and risk of chronic kidney disease in men. Nephrol Dial Transplant. (2008) 23:1246–51. doi: 10.1093/ndt/gfm757 17984108

[69] Jain G Jaimes EA. Nicotine signaling and progression of chronic kidney disease in smokers. Biochem Pharmacol. (2013) 86:1215–23. doi: 10.1016/j.bcp.2013.07.014, PMID: 23892062 PMC3838879

[70] Mercado C Jaimes EA. Cigarette smoking as a risk factor for atherosclerosis and renal disease: novel pathogenic insights. Curr Hypertens Rep. (2007) 9:66–72. doi: 10.1007/s11906-007-0012-8, PMID: 17362674

[71] Ku E Lee BJ Wei J Weir MR. Hypertension in CKD: Core curriculum 2019. Am J Kidney Dis. (2019) 74:120–31. doi: 10.1053/j.ajkd.2018.12.044, PMID: 30898362

[72] Lin L Tan W Pan X Tian E Wu Z Yang J. Metabolic syndrome-related kidney injury: a review and update. Front Endocrinol (Lausanne). (2022) 13:904001. doi: 10.3389/fendo.2022.904001, PMID: 35813613 PMC9261267

[73] Antuna-Puente B Feve B Fellahi S Bastard JP. Adipokines: the missing link between insulin resistance and obesity. Diabetes Metab. (2008) 34:2–11. doi: 10.1016/j.diabet.2007.09.004, PMID: 18093861

[74] Kirichenko TV Markina YV Bogatyreva AI Tolstik TV Varaeva YR Starodubova AV. The role of Adipokines in inflammatory mechanisms of obesity. Int J Mol Sci. (2022) 23:14982. doi: 10.3390/ijms232314982 36499312 PMC9740598

[75] Rohm TV Meier DT Olefsky JM Donath MY. Inflammation in obesity, diabetes, and related disorders. Immunity. (2022) 55:31–55. doi: 10.1016/j.immuni.2021.12.013, PMID: 35021057 PMC8773457

[76] Zhang J Wei J Sun R Sheng H Yin K Pan Y. A lncRNA from the FTO locus acts as a suppressor of the m(6)a writer complex and p53 tumor suppression signaling. Mol Cell. (2023) 83:2692–2708.e7. doi: 10.1016/j.molcel.2023.06.024, PMID: 37478845 PMC10427207

[77] Seillier M Peuget S Gayet O Gauthier C N'Guessan P Monte M. TP53INP1, a tumor suppressor, interacts with LC3 and ATG8-family proteins through the LC3-interacting region (LIR) and promotes autophagy-dependent cell death. Cell Death Differ. (2012) 19:1525–35. doi: 10.1038/cdd.2012.30 22421968 PMC3422476

[78] Li XQ Yu Q Tan WF Zhang ZL Ma H. MicroRNA-125b mimic inhibits ischemia reperfusion-induced neuroinflammation and aberrant p53 apoptotic signalling activation through targeting TP53INP1. Brain Behav Immun. (2018) 74:154–65. doi: 10.1016/j.bbi.2018.09.002, PMID: 30193876

[79] Jiang L Liu X Hu X Gao L Zeng H Wang X. METTL3-mediated m(6)a modification of TIMP2 mRNA promotes podocyte injury in diabetic nephropathy. Mol Ther. (2022) 30:1721–40. doi: 10.1016/j.ymthe.2022.01.002, PMID: 34995800 PMC9077313

[80] Wang JN Wang F Ke J Li Z Xu CH Yang Q. Inhibition of METTL3 attenuates renal injury and inflammation by alleviating TAB3 m6A modifications via IGF2BP2-dependent mechanisms. Sci Transl Med. (2022) 14:eabk2709. doi: 10.1126/scitranslmed.abk2709, PMID: 35417191

[81] Liu X Jiang L Zeng H Gao L Guo S Chen C. Circ-0000953 deficiency exacerbates podocyte injury and autophagy disorder by targeting Mir665-3p-Atg4b in diabetic nephropathy. Autophagy. (2024) 20:1072–97. doi: 10.1080/15548627.2023.2286128, PMID: 38050963 PMC11135827

[82] Li F Zhang F Wang T Xie Z Luo H Dong W. A self-amplifying loop of TP53INP1 and P53 drives oxidative stress-induced apoptosis of bone marrow mesenchymal stem cells. Apoptosis. (2024) 29:882–97. doi: 10.1007/s10495-023-01934-1, PMID: 38491252 PMC11055765

[83] Yuan Q Tang B Zhang C. Signaling pathways of chronic kidney diseases, implications for therapeutics. Signal Transduct Target Ther. (2022) 7:182. doi: 10.1038/s41392-022-01036-5, PMID: 35680856 PMC9184651

[84] Meng X Jin J Lan HY. Driving role of macrophages in transition from acute kidney injury to chronic kidney disease. Chin Med J. (2022) 135:757–66. doi: 10.1097/cm9.0000000000002100, PMID: 35671177 PMC9276339

[85] Fan J Shen W Lee SR Mathai AE Zhang R Xu G. Targeting the notch and TGF-β signaling pathways to prevent retinal fibrosis in vitro and in vivo. Theranostics. (2020) 10:7956–73. doi: 10.7150/thno.45192, PMID: 32724452 PMC7381727

[86] Lotta LA Gulati P Day FR Payne F Ongen H van de Bunt M. Integrative genomic analysis implicates limited peripheral adipose storage capacity in the pathogenesis of human insulin resistance. Nat Genet. (2017) 49:17–26. doi: 10.1038/ng.3714, PMID: 27841877 PMC5774584

[87] Han E Lee YH Lee BW Kang ES Lee IK Cha BS. Anatomic fat depots and cardiovascular risk: a focus on the leg fat using nationwide surveys (KNHANES 2008–2011). Cardiovasc Diabetol. (2017) 16:54. doi: 10.1186/s12933-017-0536-4 28441953 PMC5405479

[88] Foster MC Hwang SJ Porter SA Massaro JM Hoffmann U Fox CS. Fatty kidney, hypertension, and chronic kidney disease: the Framingham heart study. Hypertension. (2011) 58:784–90. doi: 10.1161/hypertensionaha.111.175315, PMID: 21931075 PMC3204377

[89] Gan L Yang C Zhao L Wang S Ye Y Gao Z. L3MBTL3 is a potential prognostic biomarker and correlates with immune infiltrations in gastric cancer. Cancers (Basel). (2023) 16:128. doi: 10.3390/cancers16010128 38201555 PMC10778146

[90] Hall D Giaimo BD Park SS Hemmer W Friedrich T Ferrante F. The structure, binding and function of a notch transcription complex involving RBPJ and the epigenetic reader protein L3MBTL3. Nucleic Acids Res. (2022) 50:13083–99. doi: 10.1093/nar/gkac1137, PMID: 36477367 PMC9825171

[91] Carter AR Gill D Davies NM Taylor AE Tillmann T Vaucher J. Understanding the consequences of education inequality on cardiovascular disease: mendelian randomisation study. BMJ. (2019) 365:l1855. doi: 10.1136/bmj.l1855, PMID: 31122926 PMC6529852

[92] Seyedsalehi A Warrier V Bethlehem RAI Perry BI Burgess S Murray GK. Educational attainment, structural brain reserve and Alzheimer's disease: a Mendelian randomization analysis. Brain. (2023) 146:2059–74. doi: 10.1093/brain/awac392, PMID: 36310536 PMC10151197

[93] Chen X Kong J Pan J Huang K Zhou W Diao X. Kidney damage causally affects the brain cortical structure: a Mendelian randomization study. EBioMedicine. (2021) 72:103592. doi: 10.1016/j.ebiom.2021.103592, PMID: 34619639 PMC8498227

[94] Burgess S Davies NM Thompson SG. Bias due to participant overlap in two-sample Mendelian randomization. Genet Epidemiol. (2016) 40:597–608. doi: 10.1002/gepi.21998 27625185 PMC5082560

