# Supplementary material for: Cardiometabolic traits mediating the effect of education on the risk of DKD and CKD: a Mendelian randomization study
Source: Front Nutr. 2024 Aug 13;11:1400577. doi: 10.3389/fnut.2024.1400577 (PMC11347428; doi:10.3389/fnut.2024.1400577)
Supplement: Supplementary file 1 [file Presentation_1.zip › Supplemental material/Supplemental material.docx]

**Cardiometabolic Traits Mediating the Effect of Education on the Risk of DKD and CKD: A Mendelian Randomization Study**

Yukai Wang1, Mengmeng Chen1, Lin Wang1, Yonggui Wu1,2

**Author Affiliations:**

1Department of Nephropathy, The First Affiliated Hospital of Anhui Medical University, Hefei, Anhui 230022, P.R. China.

2Center for Scientific Research of Anhui Medical University, Hefei, Anhui 230022, P.R. China.

**Address for Correspondence:**

Yonggui Wu, MD, PhD. Center for Scientific Research of Anhui Medical University, Department of Nephropathy, The First Affiliated Hospital of Anhui Medical University, Jixi road 218, Hefei, Anhui, China. E-mail: wuyonggui@medmail.com.cn. Phone number: +86 0551 6292 2111. ORCID ID: 0000-0001-6434-4759.

**CONTENTS**

[**Table S5. Data Sources of Exposures, Mediators, and Outcomes 6**](#_Toc171270505)

[**Table S6. Epidemiological evidence for the relationship between the 26 candidate mediators and DKD 8**](#_Toc171270506)

[**Table S7. Epidemiological evidence for the relationship between the 26 candidate mediators and CKD 11**](#_Toc171270507)

[**Table S8. UVMR estimating the associations of education with DKD and CKD 14**](#_Toc171270508)

[**Table S9. MR heterogeneity test of the associations of education with DKD and CKD 14**](#_Toc171270509)

[**Table S10. MR directional pleiotropy test (MR Egger) of the associations of education with DKD and CKD 15**](#_Toc171270510)

[**Table S11. UVMR assessing the causal association between education and each candidate mediator 15**](#_Toc171270511)

[**Table S12. MR heterogeneity test of the association of education with each candidate mediator 25**](#_Toc171270512)

[**Table S13. MR directional pleiotropy test (MR Egger) of the association of education with each candidate mediator 27**](#_Toc171270513)

[**Table S14. UVMR estimating the associations of candidate mediators with DKD 29**](#_Toc171270514)

[**Table S15. MR heterogeneity test of the associations of candidate mediators with DKD 36**](#_Toc171270515)

[**Table S16. MR directional pleiotropy test (MR Egger) of the association candidate mediators with DKD 38**](#_Toc171270516)

[**Table S17. MVMR assessing the causal association between each mediator and DKD with adjustment for education 40**](#_Toc171270517)

[**Table S18. UVMR estimating the associations of candidate mediators with CKD 42**](#_Toc171270518)

[**Table S19. MR heterogeneity test of the associations of candidate mediators with CKD 49**](#_Toc171270519)

[**Table S20. MR directional pleiotropy test (MR Egger) of the association candidate mediators with CKD 51**](#_Toc171270520)

[**Table S21. MVMR assessing the causal association between each mediator and CKD with adjustment for education 53**](#_Toc171270521)

[**Table S22. Identification of shared susceptibility genes using TWAS 55**](#_Toc171270522)

[**Table S23. TWAS identified shared susceptibility genes associated with education, cardiometabolic traits, and kidney diseases 56**](#_Toc171270523)

[**Figure S1. Scatter plot of SNPs associated with Education and the risk of DKD as well as CKD 304**](#_Toc171270524)

[**Figure S2. Scatter plot of SNPs associated with Education and each candidate mediator 305**](#_Toc171270525)

[**Figure S3. Scatter plot of SNPs associated with candidate mediators and DKD 307**](#_Toc171270526)

[**Figure S4. Scatter plot of SNPs associated with candidate mediators and CKD 309**](#_Toc171270527)

**References**

1. Lee JJ, Wedow R, Okbay A, et al. Gene discovery and polygenic prediction from a genome-wide association study of educational attainment in 1.1 million individuals. *Nature genetics*. Jul 23 2018;50(8):1112-1121. doi:10.1038/s41588-018-0147-3

2. Hartwig FP, Davies NM, Hemani G, Davey Smith G. Two-sample Mendelian randomization: avoiding the downsides of a powerful, widely applicable but potentially fallible technique. *International journal of epidemiology*. Dec 1 2016;45(6):1717-1726. doi:10.1093/ije/dyx028

3. Lu J, Liu X, Jiang S, et al. Body Mass Index and Risk of Diabetic Nephropathy: A Mendelian Randomization Study. *The Journal of clinical endocrinology and metabolism*. May 17 2022;107(6):1599-1608. doi:10.1210/clinem/dgac057

4. Elsayed EF, Sarnak MJ, Tighiouart H, et al. Waist-to-hip ratio, body mass index, and subsequent kidney disease and death. *American journal of kidney diseases : the official journal of the National Kidney Foundation*. Jul 2008;52(1):29-38. doi:10.1053/j.ajkd.2008.02.363

5. Pengrattanachot N, Thongnak L, Lungkaphin A. The impact of prebiotic fructooligosaccharides on gut dysbiosis and inflammation in obesity and diabetes related kidney disease. *Food & function*. Jun 6 2022;13(11):5925-5945. doi:10.1039/d1fo04428a

6. Oh SW, Ahn SY, Jianwei X, et al. Relationship between changes in body fat and a decline of renal function in the elderly. *PloS one*. 2014;9(1):e84052. doi:10.1371/journal.pone.0084052

7. Wan H, Wang Y, Xiang Q, et al. Associations between abdominal obesity indices and diabetic complications: Chinese visceral adiposity index and neck circumference. *Cardiovascular diabetology*. Jul 31 2020;19(1):118. doi:10.1186/s12933-020-01095-4

8. Zhang HS, An S, Ahn C, Park SK, Park B. Obesity measures at baseline, their trajectories over time, and the incidence of chronic kidney disease: A 14 year cohort study among Korean adults. *Nutrition, metabolism, and cardiovascular diseases : NMCD*. Mar 10 2021;31(3):782-792. doi:10.1016/j.numecd.2020.10.021

9. Wang Y, Pang X, Gu C, et al. Different associations of anthropometric indices with diabetic retinopathy and diabetic kidney disease in chinese patients with type 2 diabetes mellitus. *Acta diabetologica*. Sep 2023;60(9):1187-1198. doi:10.1007/s00592-023-02111-1

10. Eliasson B. Cigarette smoking and diabetes. *Progress in cardiovascular diseases*. Mar-Apr 2003;45(5):405-13. doi:10.1053/pcad.2003.00103

11. Orth SR, Hallan SI. Smoking: a risk factor for progression of chronic kidney disease and for cardiovascular morbidity and mortality in renal patients--absence of evidence or evidence of absence? *Clinical journal of the American Society of Nephrology : CJASN*. Jan 2008;3(1):226-36. doi:10.2215/cjn.03740907

12. Cheungpasitporn W, Thongprayoon C, Kittanamongkolchai W, et al. High alcohol consumption and the risk of renal damage: a systematic review and meta-analysis. *QJM : monthly journal of the Association of Physicians*. Jul 2015;108(7):539-48. doi:10.1093/qjmed/hcu247

13. Roy S, Schweiker-Kahn O, Jafry B, et al. Risk Factors and Comorbidities Associated with Diabetic Kidney Disease. *Journal of primary care & community health*. Jan-Dec 2021;12:21501327211048556. doi:10.1177/21501327211048556

14. Hu EA, Selvin E, Grams ME, Steffen LM, Coresh J, Rebholz CM. Coffee Consumption and Incident Kidney Disease: Results From the Atherosclerosis Risk in Communities (ARIC) Study. *American journal of kidney diseases : the official journal of the National Kidney Foundation*. Aug 2018;72(2):214-222. doi:10.1053/j.ajkd.2018.01.030

15. Fang J, Song K, Zhang D, et al. Coffee intake and risk of diabetic nephropathy: a Mendelian randomization study. *Frontiers in endocrinology*. 2023;14:1169933. doi:10.3389/fendo.2023.1169933

16. Agarwal R. Blood pressure components and the risk for end-stage renal disease and death in chronic kidney disease. *Clinical journal of the American Society of Nephrology : CJASN*. Apr 2009;4(4):830-7. doi:10.2215/cjn.06201208

17. Zhu W, Xu L, Chen X, Lee YJ, Zhang Z, Lou Q. Effects of different blood pressures and their long-term variability on the development of diabetic kidney disease in patients with type 2 diabetes mellitus. *Clinical and experimental hypertension (New York, NY : 1993)*. Jul 4 2022;44(5):464-469. doi:10.1080/10641963.2022.2071917

18. Schaeffner ES, Kurth T, Bowman TS, Gelber RP, Gaziano JM. Blood pressure measures and risk of chronic kidney disease in men. *Nephrology, dialysis, transplantation : official publication of the European Dialysis and Transplant Association - European Renal Association*. Apr 2008;23(4):1246-51. doi:10.1093/ndt/gfm757

19. De Cosmo S, Viazzi F, Piscitelli P, et al. Blood pressure status and the incidence of diabetic kidney disease in patients with hypertension and type 2 diabetes. *Journal of hypertension*. Oct 2016;34(10):2090-8. doi:10.1097/hjh.0000000000001045

20. Sacks FM, Hermans MP, Fioretto P, et al. Association between plasma triglycerides and high-density lipoprotein cholesterol and microvascular kidney disease and retinopathy in type 2 diabetes mellitus: a global case-control study in 13 countries. *Circulation*. Mar 4 2014;129(9):999-1008. doi:10.1161/circulationaha.113.002529

21. Lanktree MB, Thériault S, Walsh M, Paré G. HDL Cholesterol, LDL Cholesterol, and Triglycerides as Risk Factors for CKD: A Mendelian Randomization Study. *American journal of kidney diseases : the official journal of the National Kidney Foundation*. Feb 2018;71(2):166-172. doi:10.1053/j.ajkd.2017.06.011

22. Kintu C, Soremekun O, Kamiza AB, et al. The causal effects of lipid traits on kidney function in Africans: bidirectional and multivariable Mendelian-randomization study. *EBioMedicine*. Apr 2023;90:104537. doi:10.1016/j.ebiom.2023.104537

23. Kim MK, Han K, Koh ES, et al. Variability in Total Cholesterol Is Associated With the Risk of End-Stage Renal Disease: A Nationwide Population-Based Study. *Arteriosclerosis, thrombosis, and vascular biology*. Oct 2017;37(10):1963-1970. doi:10.1161/atvbaha.117.309803

24. Schroijen MA, de Mutsert R, Dekker FW, et al. The association of glucose metabolism and kidney function in middle-aged adults. *Clinical kidney journal*. Nov 2021;14(11):2383-2390. doi:10.1093/ckj/sfab074

25. Chen J, Muntner P, Hamm LL, et al. Insulin resistance and risk of chronic kidney disease in nondiabetic US adults. *Journal of the American Society of Nephrology : JASN*. Feb 2003;14(2):469-77. doi:10.1097/01.asn.0000046029.53933.09

26. Cai Z, Yang Y, Zhang J. Effects of physical activity on the progression of diabetic nephropathy: a meta-analysis. *Bioscience reports*. Jan 29 2021;41(1)doi:10.1042/bsr20203624

27. Seidu S, Abdool M, Almaqhawi A, et al. Physical activity and risk of chronic kidney disease: systematic review and meta-analysis of 12 cohort studies involving 1,281,727 participants. *European journal of epidemiology*. Mar 2023;38(3):267-280. doi:10.1007/s10654-022-00961-7

28. Morton RL, Schlackow I, Gray A, et al. Impact of CKD on Household Income. *Kidney international reports*. May 2018;3(3):610-618. doi:10.1016/j.ekir.2017.12.008

29. Lee YH. Socioeconomic differences among community-dwelling diabetic adults screened for diabetic retinopathy and nephropathy: The 2015 Korean Community Health Survey. *PloS one*. 2018;13(1):e0191496. doi:10.1371/journal.pone.0191496

30. Zelmanovitz T, Gerchman F, Balthazar AP, Thomazelli FC, Matos JD, Canani LH. Diabetic nephropathy. *Diabetology & metabolic syndrome*. Sep 21 2009;1(1):10. doi:10.1186/1758-5996-1-10

31. Lee YB, Han K, Kim B, et al. Risk of end-stage renal disease from chronic kidney disease defined by decreased glomerular filtration rate in type 1 diabetes: A comparison with type 2 diabetes and the effect of metabolic syndrome. *Diabetes/metabolism research and reviews*. Nov 2019;35(8):e3197. doi:10.1002/dmrr.3197

32. Lou QL, Ouyang XJ, Gu LB, et al. Chronic kidney disease and associated cardiovascular risk factors in chinese with type 2 diabetes. *Diabetes & metabolism journal*. Dec 2012;36(6):433-42. doi:10.4093/dmj.2012.36.6.433

Table S5. Data Sources of Exposures, Mediators, and Outcomes

**Exposure**

The International Standard Classification of Education (ISCED) was used to quantify educational attainment, which was then converted to US years of education and standardized so that each unit corresponds to 4.2 years of education1. Instrumental variables for education were obtained from the publicly available GWAS study of 1.1 million individuals, with pooled data including 766 345 European-descent individuals (excluding participants in 23andMe cohorts)1. Variants clumping with linkage disequilibrium r2 < 0.01 and distance threshold >10 000 kb, reaching genome-wide significance (P < 5 × 10-8), were extracted. The reference panel for linkage disequilibrium was the European sample from the 1000 Genomes Project. Proxy SNPs were chosen if there were no exposed relevant SNPs in the outcome dataset. Genetic instruments were looked for in proxy SNPs with high linkage disequilibrium (r2 > 0.8) at https://ldlink.nci.nih.gov. By combining exposure and outcome data, we harmonized genetic variations. The consistency of genetic variants across all publicly accessible datasets must be ensured, and allele mismatches must be avoided because they may bias estimates of causal effects2. SNPs that are ambiguous or palindromic were excluded from the analysis. 481 significant SNPs were selected in total as educational attainment instrumental variables.

**Mediators**

A literature review was performed to screen a total of 26 mediators related to cardiometabolic risk factors, which may be involved in the route of the causal effect of education on DKD and CKD 19-48. Data for instrumental variables were obtained from GWAS, including adiposity traits (body mass index [BMI]3,4, body fat percentage [BF%]5,6, waist-to-hip ratio [WHR]4,7, hip circumference [HC]7,8, and waist circumference [WC]8,9, smoking or dietary behavior (cigarettes smoked per day10,11, pack years of smoking10,11, maternal smoking around birth10,11, smoking initiation10,11, age of smoking Initiation10,11, alcoholic drinking12,13, and coffee intake14,15), blood pressure trait (systolic blood pressure [SBP]16,17, and diastolic blood pressure [DBP]18,19), lipids trait (triglyceride [TG]20,21, total cholesterol [TC]22,23, high-density lipoprotein cholesterol [HDL-C]20,21, and low-density lipoprotein cholesterol [LDL-C]21,22), glucose metabolism-related traits (fasting glucose24,25 and fasting insulin24,25), physical activity and sedentary behavior26,27 (moderate to vigorous physical activity [MVPA], vigorous physical activity [VPA], and sedentary behavior), socioeconomic factor (household income28,29), and diabetes (type 1 diabetes [T1D]30,31 and type 2 diabetes [T2D]30,32).

We used the following criteria to identify mediators with a more accurate causal relationship between education and DKD and CKD: first, the UVMR was used to screen for mediators with significant causal relationships between education and the 26 candidate mediators; second, the UVMR was used to assess the causal estimates of the 26 candidate mediators about DKD and CKD; statistically significant mediators were then included in further analyses; and third, the MVMR examined cardiometabolic traits with opposing associations between education and mediators, as well as between mediators and DKD and CKD, so as to recognize final mediators that play a role in the pathway of education to DKD or education to CKD.

Based on the two-sample MR framework, the UVMR and MVMR were utilized to screen potential mediators between education and DKD or CKD. The previously listed exposure-to-outcome screening criteria were used to select instrumental variables. To assess their mediating effects in the causal relationship between education and DKD or CKD, the mediation analyses included 7 and 6 mediators in the pathway from education to DKD or CKD, respectively.

**Outcomes**

The FinnGen study, which has little overlap with exposure or mediator GWAS to guarantee the lowest type 1 error rate, is a singular study that combines genome information with digital healthcare data. It is also an unprecedented global research project that represents one of the largest studies on this topic. The ICD-10 (code: N08.3*) criterion for glomerular disorders in patients with diabetes mellitus was used to define the R9 release of genome-wide analysis on DKD, which incorporated summary statistics from the FinnGen biobank and included 312 650 European individuals (4 111 cases and 308 539 controls). The R9 version of the FinnGen consortium's GWAS on CKD, which contains 363 177 European ancestry controls and 9 073 patients, was used. Cases of CKD in FinnGen were defined by hospital discharge and cause of death code N18 of International Classification of Disease-10 (ICD-10) and 585 of ICD-9. All GWAS underwent thorough quality control, informed consent from participants, and ethical approval from the relevant institutional review boards.

Table S6. Epidemiological evidence for the relationship between the 26 candidate mediators and DKD

| **Candidate mediators** | **Epidemiological evidence** |
| --- | --- |
| **Adiposity trait** | |
| BMI | One SD increase in BMI was causally associated with higher DKD risk (OR 3.76, 95% CI 1.88,7.53, P < 0.001)3. |
| BF% | Obesity is a risk factor for the development of several diseases including diabetic nephropathy. Recent studies have reported that gut dysbiosis aggravates the progression of obesity and diabetes by increasing the production of uremic toxins in conjunction with gut barrier dysfunction which then leads to increased passage of lipopolysaccharides (LPS) into the blood circulatory system eventually causing systemic inflammation5. |
| WHR | In men, a one SD increase in WHR (OR 1.18; 95% CI 1.03, 1.35) was significantly associated with a greater prevalence of DKD (P<0.05). In women, a one SD increase in WHR (OR 1.23; 95% CI 1.08,1.40) was significantly associated with a greater prevalence of DKD (P<0.05)7. |
| HC | In the restricted cubic spline models, HC showed J-shaped associations with DKD. Compared to the lowest ffth, the odds ratios (OR) based on the highest ffth of HC for DKD was 1.585(1.300–1.937) in multivariable models9. |
| WC | In men, a one SD increase in WC (OR 1.31; 95% CI 1.06, 1.60) was significantly associated with a greater prevalence of DKD (P<0.05). In women, a one SD increase in WC (OR 1.40; 95% CI 1.14, 1.72) was significantly associated with a greater prevalence of DKD (P<0.05)7. |
| **Smoking or dietary behavior** | |
| Cigarettes smoked per day | Recently, it was also shown that smoking promotes the progression of renal disease in type 2 diabetic subjects. After the 13-year follow-up period, the 2 groups (smokers and nonsmokers) had similar clinical and laboratory data, but the progression of nephropathy, as well as the development of atherosclerotic disease, was increased in the smokers. Tobacco use levels were associated independently with the impairment in renal function in a multiple regression analysis, emphasizing the possible roles of smoking in these susceptible individuals10. |
| Pack years of smoking |
| Maternal smoking around birth |
| Smoking initiation |
| Age of smoking initiation |
| Alcoholic drinking | We found decreased odds of DKD for those who consumed alcohol moderately (OR 0.612, 95% CI 0.377,0.994; P<0.05)13. |
| Coffee intake | Our current study demonstrated positive associations of genetically predicted coffee intake with diabetic nephropathy (OR=1.939; P = 0.045)15. |
| **Blood pressure trait** | |
| SBP | Adjusted for age, sex, and diabetes duration, patients in the Q4 group (SBP-Mean ≥ 130, SBP-SD ≥ 11.06) had the highest DKD prevalence (HR = 1.976, p < .001), while the Q1 group (SBP-Mean < 130, SBP-SD < 11.06) had the lowest17. |
| DBP | The observed relationship between each 5 mmHg DBP reduction (below 85 mmHg) and the incidence of DKD was statistically significant (OR 0.89 95%CI 0.85,0.93, P < 0.001)19. |
| **Lipid trait** | |
| TG | For Diabetic Kidney Disease, the odds ratio increased by 1.23 (1.16,1.31) with triglycerides and decreased by 0.86 (0.82,0.91) with high-density lipoprotein cholesterol20. |
| TC | We found a significant causal association between genetically predicted low-density lipoprotein (LDL) cholesterol and eGFR in African ancestry individuals β = 1.1(95% CI 0.411,1.788]; p = 0.002). Similarly, total cholesterol (TC) showed a significant causal effect on eGFR β = 1.619 (95% CI [0.412-2.826]; p = 0.009)22. |
| LDL-C |
| HDL-C | For Diabetic Kidney Disease, the odds ratio decreased by 0.86(0.82,0.91) with high-density lipoprotein cholesterol20. |
| **Glucose metabolism-related trait** | |
| Fasting glucose | Higher levels of fasting glucose and Fasting insulin were associated with increased odds ratios of micro-albuminuria [fasting glucose:1.21(1.04, 1.42), Fasting insulin:1.01(1.00, 1.03)]24. |
| Fasting insulin |
| **Physical activity or sedentary behavior** | |
| MVPA | The subgroup analysis revealed that the risk of DKD decreased in the physical activity group compared with the control group (OR: 0.67, 95% CI: 0.51,0.89)26. |
| VPA |
| Sedentary behavior |
| **Socioeconomic factor** | |
| Household income | After full adjustment, those who had no formal versus a college or higher education had an OR for DKD screening of 0.68(95% CI, 0.60,0.78)29. |
| **Diabetes** | |
| T1D | Nephropathy is pathologically characterized in individuals with T1D by the thickening of glomerular and tubular basal membranes, with progressive mesangial expansion (diffuse or nodular) leading to progressive reduction of glomerular filtration surface. Concurrent interstitial morphological alterations and hyalinization of afferent and efferent glomerular arterioles also occur. Podocyte abnormalities also appear to be involved in the glomerulosclerosis process. In patients with T2D, renal lesions are heterogeneous and more complex than in individuals with T1D30. |
| T2D |

Abbreviations: BMI, body mass index; BF%, body fat percentage; WHR, waist-to-hip ratio; HC, hip circumference; WC, waist circumference; SBP, systolic blood pressure; DBP, diastolic blood pressure; TG, triglyceride; TC, total cholesterol; HDL-C, high-density lipoprotein cholesterol; LDL-C, low-density lipoprotein cholesterol; MVPA, moderate to vigorous physical activity; VPA, vigorous physical activity; T1D, type 1 diabetes; T2D, type 2 diabetes; DKD, diabetic kidney disease; CKD, chronic kidney disease; BP, blood pressure; eGFR, estimated glomerular filtration rate; ESRD, end stage renal disease; OR, odds ratio; CI, confidence interval; HR, hazard ratio; RR, relative risk; SD, standard deviation.

Table S7. Epidemiological evidence for the relationship between the 26 candidate mediators and CKD

| **Candidate mediators** | **Epidemiological evidence** |
| --- | --- |
| **Adiposity trait** | |
| BMI | Individual patient data was pooled from the Atherosclerosis Risk in Communities Study and the Cardiovascular Health Study. In univariate analysis of creatinine-based Outcomes, each 2-kg/m2 increase in BMI was associated with an 11% increase in the risk of developing incident CKD (OR 1.11; 95% CI 1.06,1.15)4. |
| BF% | We divided participants into two halves according to baseline eGFR and analyzed the prevalence of renal outcome in combination with tertiles of changes of BF%. In participants with lower eGFR, the incidence of rapid renal progression was significantly increased according to the increase in body fat percent (P = 0.010). However, rapid progression was not significantly increased in participants with higher eGFR (P = 0.435)6. |
| WHR | In univariate analysis of creatinine-based outcomes, each 0.1-unit increase in WHR was associated with an 81% increase in the risk of developing CKD (OR 1.81; 95% CI 1.54,2.12)4. |
| HC | Utilizing data from 2001 to 2014 for 9,796 Korean adults without CKD at baseline, the association of baseline obesity measures with incident CKD was evaluated using logistic regression. HC increased the odds of incident CKD (OR 1.25, 95% CI 1.11,1.41)8. |
| WC | Utilizing data from 2001 to 2014 for 9,796 Korean adults without CKD at baseline, the association of baseline obesity measures with incident CKD was evaluated using logistic regression. WC increased the odds of incident CKD (OR 1.22, 95% CI 1.07,1.38)8. |
| **Smoking or dietary behavior** | |
| Cigarettes smoked per day | After adjustment for potential confounding factors, current smokers who smoked < 20 or > 20 cigarettes per day, respectively, had an elevated risk for high normal urine albumin concentration (RR 1.33 and 1.98, respectively). Current and former smokers had a marked risk for macroalbuminuria (adjusted RR 3.26 and 2.69, respectively), indicating nonreversible kidney damage related to smoking. Smokers had a slightly but significantly higher creatinine clearance than nonsmokers, at least in men, leading to the hypothesis of smoking-induced hyperfiltration11. |
| Pack years of smoking |
| Maternal smoking around birth |
| Smoking initiation |
| Age of smoking initiation |
| Alcoholic drinking | Twenty studies with 292 431 patients were included in our analysis to assess the associations between high alcoholic drinking and the progression of kidney damage. The pooled RRs of CKD, proteinuria, and ESRD in patients with high alcohol consumption were 0.83 (95% CI: 0.71,0.98), 0.85 (95% CI: 0.62,1.17) and 1.00 (95% CI: 0.55,1.82), respectively12. |
| Coffee intake | Compared with never-drinkers, each category of coffee intake was associated with a 10% to 16% lower risk for CKD (<1 cup per day: 10% lower risk [HR, 0.90; 95% CI, 0.82,0.99]; <2 cups per day: 10% lower risk [HR, 0.90; 95% CI, 0.82,0.99]; <3 cups per day: 13% lower risk [HR: 0.87, 95% CI: 0.77,0.97]; and ≥3 cups per day: 16% lower risk [HR, 0.84; 95% CI, 0.75,0.94]; P for trend < 0.001). Each additional cup of coffee consumed per day was associated with a 3% lower risk for incident CKD (HR, 0.97; 95% CI, 0.95,0.99). Compared with participants who never consumed coffee, participants who consumed any amount of coffee had an 11% lower risk for CKD (HR, 0.89; 95% CI, 0.82,0.96; P for trend < 0.001)14. |
| **Blood pressure trait** | |
| SBP | A single-center, prospective cohort study was conducted of 218 veterans with CKD (22% black, 4% women, mean age 68 years, clinic BP 154.1±25.1/85.2±13.9 mmHg, 48% with diabetes). During follow-up of up to 7 years, Compared with those with controlled SBP (<130 mmHg), patients with moderate control (130 to 149 mmHg) had an HR of 3.87 and those with poor control HR of 9.09 for ESRD16. |
| DBP | We prospectively followed 8093 male participants in the Physicians’ Health Study, without a known history of kidney disease at baseline, who provided BP values on the baseline and 24-month questionnaires, and for whom we had creatinine measures after 14 years of follow-up. After 14 years of follow-up, 1039 men (12.8%) had CKD. An increase of 10 mmHg had a corresponding multivariable-adjusted OR (95% CI) of 1.11 (1.03,1.19) for SBP and 1.05 (0.93,1.17) for DBP18. |
| **Lipid trait** | |
| TG | In multivariable analysis, a 17-mg/dl higher HDL-C concentration was associated with an 0.8% higher eGFR (95% CI,0.4%-1.3%; P = 0.004) and lower risk for eGFR < 60 mL/min/1.73 m2(OR, 0.85; 95% CI,0.77,0.93; P < 0.001), while Egger analysis showed no evidence of pleiotropy. There was no evidence for a causal relationship between LDL-C concentration and any kidney disease measure. Genetically higher TG concentrations appeared associated with higher eGFRs, but this finding was driven by a single pleiotropic variant in the glucokinase regulator gene (GCKR)21. |
| HDL-C |
| LDL-C |
| TC | Using nationally representative data from the Korean National Health Insurance System, 8 493 277 subjects who were free of ESRD and who underwent ≥3 health examinations from 2005 to 2010 were followed to the end of 2015. There were 11,247 cases of ESRD during a median follow-up of 6.1 years. There was a graded association between a higher TC variability and incident ESRD. In the multivariable-adjusted model, the HR and 95% CI comparing the highest versus lowest quartiles of the coefficient of variation of TC were 2.66 (95% CI, 2.52,2.82)23. |
| **Glucose metabolism-related trait** | |
| Fasting glucose | The result shows the proportion of participants with CKD by quartile of fasting glucose and fasting insulin among 6453 participants without diabetes. There was a significant positive relationship between higher Fasting glucose, Fasting insulin, and higher prevalence of CKD25. |
| Fasting insulin |
| **Physical activity or sedentary behavior** | |
| MVPA | In a pooled analysis of 12 studies, the multivariable-adjusted RR (95% CI) of CKD comparing the most physically active versus the least physically active groups was 0.91 (0.85,0.97)27. |
| VPA |
| Sedentary behavior |
| **Socioeconomic factor** | |
| Household income | Compared with participants with stage 3 CKD at baseline, the odds of falling into poverty were 51% higher for those with stage 4 (OR 1.51; 95% CI 1.09,2.10), 66% higher for those with stage 5 (OR 1.66; 95% CI 1.11,2.47), and 78% higher for those on dialysis at baseline (OR 1.78, 95% CI 1.22,2.60)28. |
| **Diabetes** | |
| T1D | In all models adjusted for potential confounders, the risk for incident ESRD increased in the T1D group compared with the T2D and nondiabetes groups. The HR (95% CI) for ESRD in the T1D group was 9.267 (8.378,10.251) compared with nondiabetes groups in fully adjusted model 431. |
| T2D | After adjusting for age and gender, the duration of T2D was independently associated with CKD with an OR (95% CI) of 1.03 (1.00,1.06, P=0.040)32. |

Abbreviations: BMI, body mass index; BF%, body fat percentage; WHR, waist-to-hip ratio; HC, hip circumference; WC, waist circumference; SBP, systolic blood pressure; DBP, diastolic blood pressure; TG, triglyceride; TC, total cholesterol; HDL-C, high-density lipoprotein cholesterol; LDL-C, low-density lipoprotein cholesterol; MVPA, moderate to vigorous physical activity; VPA, vigorous physical activity; T1D, type 1 diabetes; T2D, type 2 diabetes; DKD, diabetic kidney disease; CKD, chronic kidney disease; BP, blood pressure; eGFR, estimated glomerular filtration rate; ESRD, end stage renal disease; OR, odds ratio; CI, confidence interval; HR, hazard ratio; RR, relative risk; SD, standard deviation.

Table S8. UVMR estimating the associations of education with DKD and CKD

| **Exposure** | **Outcome** | **Method** | **No. of SNPs** | **β (95% CI)** | **OR (95% CI)** | **P value** |
| --- | --- | --- | --- | --- | --- | --- |
| **Education** | DKD | MR Egger | 384 | -0.8016(-1.5778,-0.0254) | 0.4486(0.2064,0.9749) | 4.36E-02 |
| **Education** | DKD | Weighted median | 384 | -0.5747(-0.8652,-0.2842) | 0.5629(0.4210,0.7526) | 1.05E-04 |
| **Education** | DKD | **Inverse variance weighted** | **384** | **-0.6662(-0.8646,-0.4678)** | **0.5136(0.4212,0.6264)** | **4.66E-11** |
| **Education** | DKD | Maximum likelihood | 384 | -0.6698(-0.8697,-0.4698) | 0.5118(0.4191,0.6251) | 5.21E-11 |
| **Education** | DKD | cML-MA | 409 | -0.6516(-0.9335,-0.3696) | 0.5212(0.3932,0.6910) | 5.92E-06 |
| **Education** | DKD | GSMR | 383 | -0.7038(-0.9128,-0.4948) | 0.4947(0.4014,0.6097) | 4.12E-11 |
| **Education** | CKD | MR Egger | 383 | -0.5677(-1.1313,-0.0041) | 0.5668(0.3226,0.9959) | 4.91E-02 |
| **Education** | CKD | Weighted median | 383 | -0.3581(-0.5636,-0.1527) | 0.6990(0.5692,0.8584) | 6.34E-04 |
| **Education** | CKD | **Inverse variance weighted** | **383** | **-0.3436(-0.4800,-0.2072)** | **0.7092(0.6188,0.8129)** | **7.96E-07** |
| **Education** | CKD | Maximum likelihood | 383 | -0.3470(-0.4845,-0.2095) | 0.7068(0.6160,0.8110） | 7.58E-07 |
| **Education** | CKD | cML-MA | 383 | -0.3390(-0.5138,-0.1643) | 0.7125(0.5982,0.8485) | 1.44E-04 |
| **Education** | CKD | GSMR | 382 | -0.3620(-0.5057,-0.2183) | 0.6963(0.6031,0.8039) | 7.90E-07 |

Abbreviations: DKD, diabetic kidney disease; CKD, chronic kidney disease; SNP, single nucleotide polymorphism; OR, odds ratio;

CI, confidence interval; GSMR, Generalized Summary-data-based Mendelian Randomization; cML-MA, Constrained Maximum Likelihood and Model Averaging; MR, Mendelian randomization; UVMR, univariable Mendelian randomization.

Table S9. MR heterogeneity test of the associations of education with DKD and CKD

| **Exposure** | **Outcome** | **Method** | **Q statistic** | **Q df** | **Q p-value** |
| --- | --- | --- | --- | --- | --- |
| Education | DKD | MR Egger | 313.46 | 382 | 9.96E-01 |
| Education | DKD | IVW | 313.58 | 383 | 9.96E-01 |
| Education | CKD | MR Egger | 318.46 | 381 | 9.91E-01 |
| Education | CKD | IVW | 319.11 | 382 | 9.92E-01 |

Abbreviations: DKD, diabetic kidney disease; CKD, chronic kidney disease; df, degree of freedom; MR, Mendelian randomization.

Table S10. MR directional pleiotropy test (MR Egger) of the associations of education with DKD and CKD

| **Exposure** | **Outcome** | **Egger intercept** | **SE** | **P-value** |
| --- | --- | --- | --- | --- |
| Education | DKD | 0.0019 | 0.0052 | 7.24E-01 |
| Education | CKD | 0.0030 | 0.0037 | 4.22E-01 |

Abbreviations: DKD, diabetic kidney disease; CKD, chronic kidney disease; SE, standard error; MR, Mendelian randomization.

Table S11. UVMR assessing the causal association between education and each candidate mediator

| **Exposure** | **Candidate mediators** | **Method** | **No. of SNPs** | **β (95% CI)** | **OR (95% CI)** | **P value** |
| --- | --- | --- | --- | --- | --- | --- |
| Education | BMI | MR Egger | 183 | -0.3861(-0.5110,-0.2611) | 0.6797(0.5999,0.7702) | 7.84E-09 |
| Education | BMI | Weighted median | 183 | -0.2857(-0.3198,-0.2515) | 0.7515(0.7263,0.7776) | 2.08E-60 |
| **Education** | **BMI** | **Inverse variance weighted** | **183** | **-0.2942(-0.3187,-0.2698)** | **0.7451(0.7271,0.7635)** | **5.28E-123** |
| Education | BMI | Maximum likelihood | 183 | -0.2926(-0.3172,-0.2680) | 0.7463(0.7282,0.7649) | 3.25E-120 |
| Education | BMI | cML-MA | 183 | -0.3020(-0.3290,-0.2749) | 0.7394(0.7196,0.7596) | 3.14E-106 |
| Education | BMI | GSMR | 183 | -0.3086(-0.3332,-0.2841) | 0.7344(0.7166,0.7527) | 1.02E-133 |
| Education | BF% | MR Egger | 289 | -0.2954(-0.5450,-0.0457) | 0.7443(0.5798,0.9553) | 2.11E-02 |
| Education | BF% | Weighted median | 289 | -0.1927(-0.2765,-0.1089) | 0.8247(0.7584,0.8968) | 6.60E-06 |
| **Education** | **BF%** | **Inverse variance weighted** | **289** | **-0.1606(-0.2179,-0.1033)** | **0.8517(0.8042,0.9019)** | **3.96E-08** |
| Education | BF% | Maximum likelihood | 289 | -0.1604(-0.2181,-0.1028) | 0.8518(0.8040,0.9023) | 5.00E-08 |
| Education | BF% | cML-MA | 289 | -0.1644(-0.2268,-0.1021) | 0.8484(0.7971,0.9029) | 2.31E-07 |
| Education | BF% | GSMR | 288 | -0.1716(-0.2321,-0.1111) | 0.8423(0.7929,0.8949) | 2.72E-08 |
| Education | WHR | MR Egger | 303 | -0.1892(-0.4129,0.0345) | 0.8276(0.6617,1.0351) | 9.83E-02 |
| Education | WHR | Weighted median | 303 | -0.1896(-0.2685,-0.1106) | 0.8273(0.7645,0.8953) | 2.52E-06 |
| **Education** | **WHR** | **Inverse variance weighted** | **303** | **-0.2157(-0.2670,-0.1643)** | **0.8060(0.7657,0.8485)** | **1.79E-16** |
| Education | WHR | Maximum likelihood | 303 | -0.2152(-0.2670,-0.1635) | 0.8063(0.7657,0.8491) | 3.39E-16 |
| Education | WHR | cML-MA | 303 | -0.2206(-0.2789,-0.1624) | 0.8020(0.7566,0.8501) | 1.12E-13 |
| Education | WHR | GSMR | 303 | -0.2262(-0.2802,-0.1721) | 0.7976(0.7556,0.8419) | 2.43E-16 |
| Education | HC | MR Egger | 285 | 0.1311(0.2023,0.3344) | 1.1401(0.9304,1.3971) | 2.07E-01 |
| Education | HC | Weighted median | 285 | 0.1109(0.0433,0.1786) | 1.1173(1.0442,1.1955) | 1.31E-03 |
| **Education** | **HC** | **Inverse variance weighted** | **285** | **0.1086(0.0634,0.1539)** | **1.1148(1.0654,1.1663)** | **2.52E-06** |
| Education | HC | Maximum likelihood | 285 | 0.1091(0.0635,0.1548) | 1.1153(1.0656,1.1674) | 2.74E-06 |
| Education | HC | cML-MA | 285 | 0.1095(0.0584,0.1607) | 1.1158(1.0602,1.1743) | 2.68E-05 |
| Education | HC | GSMR | 285 | 0.1141(0.0664,0.1618) | 1.1209(1.0687,1.1756) | 2.73E-06 |
| Education | WC | MR Egger | 287 | 0.1297(-0.0563,0.3156) | 1.1384(0.9453,1.3711) | 1.73E-01 |
| Education | WC | Weighted median | 287 | -0.0244(-0.0904,0.0417) | 0.9759(0.9135,1.0426) | 4.70E-01 |
| **Education** | **WC** | **Inverse variance weighted** | **287** | **-0.0263(-0.0688,0.0163)** | **0.9741(0.9335,1.0164)** | **2.26E-01** |
| Education | WC | Maximum likelihood | 287 | -0.0262(-0.0691,0.0167) | 0.9741(0.9332,1.0169) | 2.31E-01 |
| Education | WC | cML-MA | 287 | -0.0279(-0.0771,0.0213) | 0.9725(0.9258,1.0215) | 2.67E-01 |
| Education | WC | GSMR | 287 | -0.0274(-0.0722,0.0174) | 0.9730(0.9304,1.0176) | 2.31E-01 |
| Education | Cigarettes smoked per day | MR Egger | 341 | -0.3204(-0.5261,-0.1146) | 0.7259(0.5909,0.8917) | 2.45E-03 |
| Education | Cigarettes smoked per day | Weighted median | 341 | -0.2494(-0.3256,-0.1732) | 0.7792(0.7221,0.8409) | 1.40E-10 |
| **Education** | **Cigarettes smoked per day** | **Inverse variance weighted** | **341** | **-0.2799(-0.3314,-0.2285)** | **0.7558(0.7179,0.7957)** | **1.48E-26** |
| Education | Cigarettes smoked per day | Maximum likelihood | 341 | -0.2800(-0.3319,-0.2281) | 0.7557(0.7175,0.7960) | 3.85E-26 |
| Education | Cigarettes smoked per day | cML-MA | 341 | -0.2840(-0.3482,-0.2197) | 0.7528(0.7059,0.8028) | 4.71E-18 |
| Education | Cigarettes smoked per day | GSMR | 340 | -0.2962(-0.3506,-0.2418) | 0.7436(0.7042,0.7852) | 1.34E-26 |
| Education | Pack years of smoking | MR Egger | 336 | -0.3397(-0.4667,-0.2126) | 0.7120(0.6271,0.8085) | 2.86E-07 |
| Education | Pack years of smoking | Weighted median | 336 | -0.3218(-0.3729,-0.2708) | 0.7248(0.6887,0.7628) | 4.80E-35 |
| **Education** | **Pack years of smoking** | **Inverse variance weighted** | **336** | **-0.3202(-0.3528,-0.2877)** | **0.7260(0.7027,0.7500)** | **7.33E-83** |
| Education | Pack years of smoking | Maximum likelihood | 336 | -0.3200(-0.3529,-0.2870) | 0.7262(0.7026,0.7505) | 1.03E-80 |
| Education | Pack years of smoking | cML-MA | 336 | -0.3268(-0.3644,-0.2892) | 0.7212(0.6946,0.7489) | 4.51E-65 |
| Education | Pack years of smoking | GSMR | 335 | -0.3384(-0.3731,-0.3036) | 0.7129(0.6886,0.7381) | 3.26E-81 |
| Education | Maternal smoking around birth | MR Egger | 350 | -0.1020(-0.1462,-0.0578) | 0.9030(0.8640,0.9439) | 8.50E-06 |
| Education | Maternal smoking around birth | Weighted median | 350 | -0.1110(-0.1277,-0.0943) | 0.8949(0.8801,0.9100) | 7.91E-39 |
| **Education** | **Maternal smoking around birth** | **Inverse variance weighted** | **350** | **-0.1189(-0.1299,-0.1078)** | **0.8879(0.8782,0.8978)** | **2.38E-98** |
| Education | Maternal smoking around birth | Maximum likelihood | 350 | -0.1182(-0.1295,-0.1070) | 0.8885(0.8786,0.8985) | 9.76E-95 |
| Education | Maternal smoking around birth | cML-MA | 350 | -0.1207(-0.1330,-0.1083) | 0.8863(0.8754,0.8974) | 2.87E-81 |
| Education | Maternal smoking around birth | GSMR | 349 | -0.1253(-0.1371,-0.1135) | 0.8822(0.8719,0.8927) | 2.85E-96 |
| Education | Smoking initiation | MR Egger | 294 | -0.3125(-0.4603,-0.1648) | 0.7316(0.6311,0.8480) | 4.42E-05 |
| Education | Smoking initiation | Weighted median | 294 | -0.3355(-0.3912,-0.2799) | 0.7149(0.6763,0.7558) | 2.89E-32 |
| **Education** | **Smoking initiation** | **Inverse variance weighted** | **294** | **-0.3670(-0.4033,-0.3306)** | **0.6928(0.6681,0.7185)** | **6.17E-87** |
| Education | Smoking initiation | Maximum likelihood | 294 | -0.3668(-0.4038,-0.3298) | 0.6929(0.6678,0.7191) | 3.58E-84 |
| Education | Smoking initiation | cML-MA | 294 | -0.3788(-0.4214,-0.3361) | 0.6847(0.6561,0.7146) | 9.14E-68 |
| Education | Smoking initiation | GSMR | 294 | -0.3854(-0.4243,-0.3465) | 0.6802(0.6542,0.7071) | 4.54E-84 |
| Education | Age of smoking initiation | MR Egger | 360 | 0.3442(0.2460,0.4424) | 1.4109(1.2790,1.5564) | 2.83E-11 |
| Education | Age of smoking initiation | Weighted median | 360 | 0.2741(0.2363,0.3120) | 1.3154(1.2666,1.3661) | 8.01E-46 |
| **Education** | **Age of smoking initiation** | **Inverse variance weighted** | **360** | **0.2777(0.2528,0.3027)** | **1.3201(1.2876,1.3535)** | **1.33E-105** |
| Education | Age of smoking initiation | Maximum likelihood | 360 | 0.2788(0.2535,0.3041) | 1.3216(1.2885,1.3554) | 2.46E-103 |
| Education | Age of smoking initiation | cML-MA | 360 | 0.2837(0.2548,0.3126) | 1.3280(1.2902,1.3670) | 2.53E-82 |
| Education | Age of smoking initiation | GSMR | 359 | 0.2932(0.2666,0.3199) | 1.3407(1.3055,1.3770) | 4.23E-103 |
| Education | Alcoholic drinking | MR Egger | 325 | -0.0054(-0.0822,0.0713) | 0.9946(0.9211,1.0740) | 8.90E-01 |
| Education | Alcoholic drinking | Weighted median | 325 | -0.0074(-0.0352,0.0205) | 0.9927(0.9654,1.0207) | 6.05E-01 |
| **Education** | **Alcoholic drinking** | **Inverse variance weighted** | **325** | **-0.0031(-0.0217,0.0155)** | **0.9969(0.9786,1.0156)** | **7.46E-01** |
| Education | Alcoholic drinking | Maximum likelihood | 325 | -0.0031(-0.0219,0.0157) | 0.9969(0.9784,1.0158) | 7.49E-01 |
| Education | Alcoholic drinking | cML-MA | 325 | -0.0033(-0.0258,0.0193) | 0.9967(0.9745,1.0195) | 7.77E-01 |
| Education | Alcoholic drinking | GSMR | 324 | -0.0032(-0.0227,0.0164) | 0.9968(0.9775,1.0165) | 7.51E-01 |
| Education | Coffee intake | MR Egger | 342 | 0.0456(-0.0151,0.1062) | 1.0466(0.9850,1.1121) | 1.42E-01 |
| Education | Coffee intake | Weighted median | 342 | 0.0895(0.0670,0.1120) | 1.0936(1.0693,1.1185) | 5.84E-15 |
| **Education** | **Coffee intake** | **Inverse variance weighted** | **342** | **0.0883(0.0732,0.1033)** | **1.0923(1.0760,1.1088)** | **1.49E-30** |
| Education | Coffee intake | Maximum likelihood | 342 | 0.0891(0.0739,0.1043) | 1.0932(1.0767,1.1100) | 1.45E-30 |
| Education | Coffee intake | cML-MA | 342 | 0.0906(0.0737,0.1076) | 1.0949(1.0765,1.1136) | 9.90E-26 |
| Education | Coffee intake | GSMR | 342 | 0.0929(0.0769,0.1088) | 1.0973(1.0799,1.1149) | 3.67E-30 |
| Education | SBP | MR Egger | 270 | -2.6938(-4.3483,-1.0393) | 0.0676(0.0129,0.3537) | 1.59E-03 |
| Education | SBP | Weighted median | 270 | -2.7636(-3.2679,-2.2593) | 0.0631(0.0381,0.1044) | 6.57E-27 |
| **Education** | **SBP** | **Inverse variance weighted** | **270** | **-2.8239(-3.1674,-2.4803)** | **0.0594(0.0421,0.0837)** | **2.13E-58** |
| Education | SBP | Maximum likelihood | 270 | -2.7991(-3.1461,-2.4522) | 0.0609(0.0430,0.0861) | 2.55E-56 |
| Education | SBP | cML-MA | 270 | -2.9319(-3.4404,-2.4234) | 0.0533(0.0321,0.0886) | 1.29E-29 |
| Education | SBP | GSMR | 270 | -2.9707(-3.3166,-2.6248) | 0.0513(0.0363,0.0725) | 1.41E-63 |
| Education | DBP | MR Egger | 283 | -0.5122(-1.3402,0.3157) | 0.5991(0.2618,1.3712) | 2.26E-01 |
| Education | DBP | Weighted median | 283 | -1.0005(-1.2775,-0.7236) | 0.3677(0.2787,0.4850) | 1.44E-12 |
| **Education** | **DBP** | **Inverse variance weighted** | **283** | **-1.1212(-1.3088,-0.9336)** | **0.3259(0.2701,0.3931)** | **1.09E-31** |
| Education | DBP | Maximum likelihood | 283 | -1.1224(-1.3117,-0.9331) | 0.3255(0.2693,0.3933) | 3.23E-31 |
| Education | DBP | cML-MA | 283 | -1.1416(-1.3888,-0.8943) | 0.3193(0.2494,0.4089) | 1.42E-19 |
| Education | DBP | GSMR | 283 | -1.1770(-1.3687,-0.9852) | 0.3082(0.2544,0.3734) | 2.44E-33 |
| Education | TG | MR Egger | 308 | -0.2163(-0.4159,-0.0167) | 0.8055(0.6597,0.9834) | 3.45E-02 |
| Education | TG | Weighted median | 308 | -0.1313(-0.1982,-0.0643) | 0.8770(0.8202,0.9377) | 1.22E-04 |
| **Education** | **TG** | **Inverse variance weighted** | **308** | **-0.1426(-0.1876,-0.0977)** | **0.8671(0.8289,0.9069)** | **5.01E-10** |
| Education | TG | Maximum likelihood | 308 | -0.1428(-0.1881,-0.0974) | 0.8670(0.8285,0.9072) | 6.67E-10 |
| Education | TG | cML-MA | 308 | -0.1458(-0.1943,-0.0973） | 0.8643(0.8234,0.9072) | 3.73E-09 |
| Education | TG | GSMR | 308 | -0.1510(-0.1984,-0.1036) | 0.8598(0.8201,0.9016) | 4.21E-10 |
| Education | TC | MR Egger | 299 | -0.1691(-0.3952,0.0570) | 0.8444(0.6735,1.0587) | 1.44E-01 |
| Education | TC | Weighted median | 299 | -0.0658(-0.1417,0.0100) | 0.9363(0.8679,1.0101) | 8.90E-02 |
| **Education** | **TC** | **Inverse variance weighted** | **299** | **-0.0801(-0.1305,-0.0298)** | **0.9230(0.8776,0.9707)** | **1.82E-03** |
| Education | TC | Maximum likelihood | 299 | -0.0800(-0.1308,-0.0292) | 0.9231(0.8774,0.9712) | 2.01E-03 |
| Education | TC | cML-MA | 299 | -0.0820(-0.1382,-0.0258) | 0.9213(0.8709,0.9746) | 4.27E-03 |
| Education | TC | GSMR | 298 | -0.0824(-0.1356,-0.0293) | 0.9209(0.8732,0.9711) | 2.36E-03 |
| Education | HDL-C | MR Egger | 292 | 0.0327(-0.1733,0.2386) | 1.0332(0.8409,1.2695) | 7.56E-01 |
| Education | HDL-C | Weighted median | 292 | 0.0901(0.0187,0.1615) | 1.0943(1.0188,1.1753) | 1.34E-02 |
| **Education** | **HDL-C** | **Inverse variance weighted** | **292** | **0.1109(0.0634,0.1583)** | **1.1172(1.0655,1.1715)** | **4.60E-06** |
| Education | HDL-C | Maximum likelihood | 292 | 0.1114(0.0636,0.1592) | 1.1178(1.0656,1.1726) | 5.01E-06 |
| Education | HDL-C | cML-MA | 292 | 0.1113(0.0541,0.1684) | 1.1177(1.0556,1.1834) | 1.35E-04 |
| Education | HDL-C | GSMR | 292 | 0.1179(0.0679,0.1678) | 1.1251(1.0703,1.1827) | 3.74E-06 |
| Education | LDL-C | MR Egger | 307 | -0.0874(-0.3149,0.1401) | 0.9163(0.7298,1.1504) | 4.52E-01 |
| Education | LDL-C | Weighted median | 307 | -0.0710(-0.1485,0.0065) | 0.9315(0.8620,1.0065) | 7.25E-02 |
| **Education** | **LDL-C** | **Inverse variance weighted** | **307** | **-0.0938(-0.1448,-0.0429)** | **0.9104(0.8652,0.9580)** | **3.05E-04** |
| Education | LDL-C | Maximum likelihood | 307 | -0.0937(-0.1451,-0.0424) | 0.9105(0.8650,0.9585) | 3.44E-04 |
| Education | LDL-C | cML-MA | 307 | -0.0934(-0.1507,-0.0360) | 0.9109(0.8601,0.9646) | 1.42E-03 |
| Education | LDL-C | GSMR | 306 | -0.0976(-0.1513,-0.0439) | 0.9070(0.8596,0.9570) | 3.68E-04 |
| Education | Fasting glucose | MR Egger | 44 | 0.0315(-0.1812,0.2442) | 1.0320(0.8343,1.2766) | 7.73E-01 |
| Education | Fasting glucose | Weighted median | 44 | -0.0634(-0.1428,0.0161) | 0.9386(0.8669,1.0162) | 1.18E-01 |
| **Education** | **Fasting glucose** | **Inverse variance weighted** | **44** | **-0.0917(-0.1457,-0.0378)** | **0.9123(0.8644,0.9629)** | **8.66E-04** |
| Education | Fasting glucose | Maximum likelihood | 44 | -0.0920(-0.1465,-0.0376) | 0.9121(0.8637,0.9631) | 9.26E-04 |
| Education | Fasting glucose | cML-MA | 44 | -0.0916(-0.1507,-0.0325) | 0.9124(0.8601,0.9680) | 2.38E-03 |
| Education | Fasting glucose | GSMR | 43 | -0.0970(-0.1540,-0.0400) | 0.9075(0.8573,0.9608) | 8.48E-04 |
| Education | Fasting insulin | MR Egger | 42 | -0.2588(-0.4234,-0.0942) | 0.7720(0.6548,0.9101) | 3.72E-03 |
| Education | Fasting insulin | Weighted median | 42 | -0.1112(-0.1878,-0.0346) | 0.8948(0.8287,0.9660) | 4.46E-03 |
| **Education** | **Fasting insulin** | **Inverse variance weighted** | **42** | **-0.0717(-0.1227,-0.0206)** | **0.9308(0.8845,0.9796)** | **5.91E-03** |
| Education | Fasting insulin | Maximum likelihood | 42 | -0.0726(-0.1241,-0.0211) | 0.9300(0.8833,0.9791) | 5.71E-03 |
| Education | Fasting insulin | cML-MA | 42 | -0.0740(-0.1291,-0.0190) | 0.9287(0.8789,0.9812) | 8.39E-03 |
| Education | Fasting insulin | GSMR | 41 | -0.0811(-0.1350,-0.0273) | 0.9221(0.8737,0.9731) | 3.14E-03 |
| Education | MVPA | MR Egger | 349 | -0.0723(-0.1562,0.0117) | 0.9303(0.8554,1.0118) | 9.26E-02 |
| Education | MVPA | Weighted median | 349 | -0.1064(-0.1364,-0.0764) | 0.8991(0.8725,0.9264) | 3.54E-12 |
| **Education** | **MVPA** | **Inverse variance weighted** | **349** | **-0.1059(-0.1264,-0.0854)** | **0.8995(0.8813,0.9181)** | **3.76E-24** |
| Education | MVPA | Maximum likelihood | 349 | -0.1064(-0.1271,-0.0857) | 0.8991(0.8807,0.9178) | 6.29E-24 |
| Education | MVPA | cML-MA | 349 | -0.1084(-0.1317,-0.0851) | 0.8973(0.8766,0.9184) | 7.27E-20 |
| Education | MVPA | GSMR | 349 | -0.1112(-0.1329,-0.0896) | 0.8947(0.8756,0.9143) | 5.96E-24 |
| Education | VPA | MR Egger | 352 | 0.0041(-0.0443,0.0526) | 1.0041(0.9566,1.0540) | 8.68E-01 |
| Education | VPA | Weighted median | 352 | 0.0355(0.0178,0.0532) | 1.0361(1.0179,1.0547) | 8.74E-05 |
| **Education** | **VPA** | **Inverse variance weighted** | **352** | **0.0278(0.0157,0.0399)** | **1.0281(1.0158,1.0407)** | **6.99E-06** |
| Education | VPA | Maximum likelihood | 352 | 0.0282(0.0159,0.0404) | 1.0286(1.0161,1.0412) | 6.18E-06 |
| Education | VPA | cML-MA | 352 | 0.0284(0.0152,0.0415) | 1.0288(1.0153,1.0424) | 2.39E-05 |
| Education | VPA | GSMR | 352 | 0.0288(0.0161,0.0416) | 1.0293(1.0162,1.0425) | 9.28E-06 |
| Education | Sedentary behavior | MR Egger | 363 | 0.6628(0.4707,0.8549) | 1.9402(1.6012,2.3510) | 5.44E-11 |
| Education | Sedentary behavior | Weighted median | 363 | 0.5667(0.5029,0.6306) | 1.7625(1.6534,1.8787) | 9.04E-68 |
| **Education** | **Sedentary behavior** | **Inverse variance weighted** | **363** | **0.6082(0.5647,0.6517)** | **1.8372(1.7590,1.9188)** | **2.05E-165** |
| Education | Sedentary behavior | Maximum likelihood | 363 | 0.6102(0.5656,0.6548) | 1.8408(1.7605,1.9247) | 1.86E-158 |
| Education | Sedentary behavior | cML-MA | 363 | 0.6207(0.5681,0.6733) | 1.8603(1.7649,1.9607) | 2.45E-118 |
| Education | Sedentary behavior | GSMR | 362 | 0.6404(0.5933,0.6874) | 1.8972(1.8100,1.9886) | 8.93E-157 |
| Education | Household income | MR Egger | 337 | 0.6834(0.5729,0.7939) | 1.9806(1.7734,2.2120) | 2.78E-28 |
| Education | Household income | Weighted median | 337 | 0.5941(0.5538,0.6345) | 1.8115(1.7398,1.8861) | 5.83E-183 |
| **Education** | **Household income** | **Inverse variance weighted** | **337** | **0.6159(0.5907,0.6411)** | **1.8513(1.8053,1.8985)** | **0.00E+00** |
| Education | Household income | Maximum likelihood | 337 | 0.6136(0.5868,0.6404) | 1.8471(1.7983,1.8973) | 0.00E+00 |
| Education | Household income | cML-MA | 337 | 0.6292(0.5993,0.6591) | 1.8762(1.8209,1.9331) | 0.00E+00 |
| Education | Household income | GSMR | 337 | 0.6489(0.6203,0.6775) | 1.9134(1.8595,1.9689) | 0.00E+00 |
| Education | T1D | MR Egger | 290 | -0.2665(-1.2201,0.6872) | 0.7661(0.2952,1.9882) | 5.84E-01 |
| Education | T1D | Weighted median | 290 | -0.1732(-0.4980,0.1516) | 0.8410(0.6077,1.1637) | 2.96E-01 |
| **Education** | **T1D** | **Inverse variance weighted** | **290** | **-0.1384(-0.3744,0.0975)** | **0.8707(0.6877,1.1024)** | **2.50E-01** |
| Education | T1D | Maximum likelihood | 290 | -0.1399(-0.3767,0.0970) | 0.8695(0.6861,1.1019) | 2.47E-01 |
| Education | T1D | cML-MA | 290 | -0.1454(-0.4213,0.1306) | 0.8647(0.6562,1.1395) | 3.02E-01 |
| Education | T1D | GSMR | 289 | -0.1395(-0.3883,0.1094) | 0.8698(0.6782,1.1156) | 2.72E-01 |
| Education | T2D | MR Egger | 241 | -0.5229(-0.8802,-0.1655) | 0.5928(0.4147,0.8474) | 4.50E-03 |
| Education | T2D | Weighted median | 241 | -0.3740(-0.4996,-0.2485) | 0.6880(0.6068,0.7800) | 5.27E-09 |
| **Education** | **T2D** | **Inverse variance weighted** | **241** | **-0.3593(-0.4442,-0.2745)** | **0.6982(0.6414,0.7600)** | **1.04E-16** |
| Education | T2D | Maximum likelihood | 241 | -0.3601(-0.4456,-0.2746) | 0.6976(0.6405,0.7599) | 1.53E-16 |
| Education | T2D | cML-MA | 241 | -0.3696(-0.4633,-0.2760) | 0.6910(0.6292,0.7588) | 1.00E-14 |
| Education | T2D | GSMR | 241 | -0.3784(-0.4679,-0.2889) | 0.6850(0.6263,0.7491) | 1.16E-16 |

Abbreviations: BMI, body mass index; BF%, body fat percentage; WHR, waist-to-hip ratio; HC, hip circumference; WC, waist circumference; SBP, systolic blood pressure; DBP, diastolic blood pressure; TG, triglyceride; TC, total cholesterol; HDL-C, high-density lipoprotein cholesterol; LDL-C, low-density lipoprotein cholesterol; MVPA, moderate to vigorous physical activity; VPA, vigorous physical activity; T1D, type 1 diabetes; T2D, type 2 diabetes; SNP, single nucleotide polymorphism; OR, odds ratio; CI, confidence interval; GSMR, Generalized Summary-data-based Mendelian Randomization; cML-MA, Constrained Maximum Likelihood and Model Averaging; MR, Mendelian randomization; UVMR, univariable Mendelian randomization.

Table S12. MR heterogeneity test of the association of education with each candidate mediator

| **Exposure** | **Candidate mediators** | **Method** | **Q statistic** | **Q df** | **Q p-value** |
| --- | --- | --- | --- | --- | --- |
| Education | BMI | MR Egger | 213.41 | 181 | 4.99E-02 |
| Education | BMI | IVW | 215.95 | 182 | 4.33E-02 |
| Education | BF% | MR Egger | 207.75 | 287 | 1.00E+00 |
| Education | BF% | IVW | 208.94 | 288 | 1.00E+00 |
| Education | WHR | MR Egger | 263.86 | 301 | 9.40E-01 |
| Education | WHR | IVW | 263.91 | 302 | 9.44E-01 |
| Education | HC | MR Egger | 248.20 | 283 | 9.33E-01 |
| Education | HC | IVW | 248.25 | 284 | 9.38E-01 |
| Education | WC | MR Egger | 236.70 | 285 | 9.83E-01 |
| Education | WC | IVW | 239.55 | 286 | 9.79E-01 |
| Education | Cigarettes smoked per day | MR Egger | 316.57 | 339 | 8.04E-01 |
| Education | Cigarettes smoked per day | IVW | 316.73 | 340 | 8.13E-01 |
| Education | Pack years of smoking | MR Egger | 280.41 | 334 | 9.85E-01 |
| Education | Pack years of smoking | IVW | 280.50 | 335 | 9.86E-01 |
| Education | Maternal smoking around birth | MR Egger | 293.48 | 348 | 9.85E-01 |
| Education | Maternal smoking around birth | IVW | 294.07 | 349 | 9.85E-01 |
| Education | Smoking initiation | MR Egger | 288.98 | 292 | 5.39E-01 |
| Education | Smoking initiation | IVW | 289.54 | 293 | 5.46E-01 |
| Education | Age of smoking initiation | MR Egger | 314.59 | 358 | 9.52E-01 |
| Education | Age of smoking initiation | IVW | 316.48 | 359 | 9.48E-01 |
| Education | Alcoholic drinking | MR Egger | 308.32 | 323 | 7.12E-01 |
| Education | Alcoholic drinking | IVW | 308.33 | 324 | 7.26E-01 |
| Education | Coffee intake | MR Egger | 312.67 | 340 | 8.54E-01 |
| Education | Coffee intake | IVW | 314.69 | 341 | 8.43E-01 |
| Education | SBP | MR Egger | 302.38 | 268 | 7.30E-02 |
| Education | SBP | IVW | 302.40 | 269 | 7.88E-02 |
| Education | DBP | MR Egger | 302.26 | 281 | 1.83E-01 |
| Education | DBP | IVW | 304.62 | 282 | 1.70E-01 |
| Education | TG | MR Egger | 257.26 | 306 | 9.80E-01 |
| Education | TG | IVW | 257.82 | 307 | 9.81E-01 |
| Education | TC | MR Egger | 242.54 | 297 | 9.91E-01 |
| Education | TC | IVW | 243.17 | 298 | 9.91E-01 |
| Education | HDL-C | MR Egger | 271.86 | 290 | 7.71E-01 |
| Education | HDL-C | IVW | 272.44 | 291 | 7.76E-01 |
| Education | LDL-C | MR Egger | 247.10 | 305 | 9.94E-01 |
| Education | LDL-C | IVW | 247.10 | 306 | 9.94E-01 |
| Education | Fasting glucose | MR Egger | 38.74 | 42 | 6.15E-01 |
| Education | Fasting glucose | IVW | 40.12 | 43 | 5.97E-01 |
| Education | Fasting insulin | MR Egger | 33.50 | 40 | 7.56E-01 |
| Education | Fasting insulin | IVW | 38.99 | 41 | 5.60E-01 |
| Education | MVPA | MR Egger | 334.88 | 347 | 6.70E-01 |
| Education | MVPA | IVW | 335.53 | 348 | 6.75E-01 |
| Education | VPA | MR Egger | 301.50 | 350 | 9.71E-01 |
| Education | VPA | IVW | 302.48 | 351 | 9.71E-01 |
| Education | Sedentary behavior | MR Egger | 304.88 | 361 | 9.85E-01 |
| Education | Sedentary behavior | IVW | 305.21 | 362 | 9.86E-01 |
| Education | Household income | MR Egger | 315.47 | 335 | 7.71E-01 |
| Education | Household income | IVW | 316.98 | 336 | 7.65E-01 |
| Education | T1D | MR Egger | 111.87 | 288 | 1.00E+00 |
| Education | T1D | IVW | 111.94 | 289 | 1.00E+00 |
| Education | T2D | MR Egger | 148.53 | 239 | 1.00E+00 |
| Education | T2D | IVW | 149.38 | 240 | 1.00E+00 |

Abbreviations: BMI, body mass index; BF%, body fat percentage; WHR, waist-to-hip ratio; HC, hip circumference; WC, waist circumference; SBP, systolic blood pressure; DBP, diastolic blood pressure; TG, triglyceride; TC, total cholesterol; HDL-C, high-density lipoprotein cholesterol; LDL-C, low-density lipoprotein cholesterol; MVPA, moderate to vigorous physical activity; VPA, vigorous physical activity; T1D, type 1 diabetes; T2D, type 2 diabetes; IVW, Inverse variance weighted, df, degree of freedom; MR, Mendelian randomization.

Table S13. MR directional pleiotropy test (MR Egger) of the association of education with each candidate mediator

| **Exposure** | **Candidate mediators** | **Egger intercept** | **SE** | **P-value** |
| --- | --- | --- | --- | --- |
| Education | BMI | 0.0012 | 0.0008 | 1.44E-01 |
| Education | BF% | 0.0018 | 0.0016 | 2.78E-01 |
| Education | WHR | -0.0004 | 0.0015 | 8.12E-01 |
| Education | HC | -0.0003 | 0.0013 | 8.24E-01 |
| Education | WC | -0.0021 | 0.0012 | 9.24E-02 |
| Education | Cigarettes smoked per day | 0.0005 | 0.0014 | 6.91E-01 |
| Education | Pack years of smoking | 0.0003 | 0.0009 | 7.57E-01 |
| Education | Maternal smoking around birth | -0.0002 | 0.0003 | 4.40E-01 |
| Education | Smoking initiation | -0.0007 | 0.0010 | 4.57E-01 |
| Education | Age of smoking initiation | -0.0009 | 0.0007 | 1.71E-01 |
| Education | Alcoholic drinking | 0.0000 | 0.0005 | 9.51E-01 |
| Education | Coffee intake | 0.0006 | 0.0004 | 1.55E-01 |
| Education | SBP | -0.0017 | 0.0108 | 8.75E-01 |
| Education | DBP | -0.0081 | 0.0054 | 1.40E-01 |
| Education | TG | 0.0010 | 0.0013 | 4.58E-01 |
| Education | TC | 0.0012 | 0.0015 | 4.30E-01 |
| Education | HDL-C | 0.0010 | 0.0014 | 4.45E-01 |
| Education | LDL-C | -0.0001 | 0.0015 | 9.55E-01 |
| Education | Fasting glucose | -0.0016 | 0.0014 | 2.47E-01 |
| Education | Fasting insulin | 0.0026 | 0.0011 | 2.42E-02 |
| Education | MVPA | -0.0005 | 0.0006 | 4.18E-01 |
| Education | VPA | 0.0003 | 0.0003 | 3.24E-01 |
| Education | Sedentary behavior | -0.0007 | 0.0012 | 5.68E-01 |
| Education | Household income | -0.0009 | 0.0007 | 2.20E-01 |
| Education | T1D | 0.0017 | 0.0064 | 7.86E-01 |
| Education | T2D | 0.0022 | 0.0024 | 3.57E-01 |

Abbreviations: BMI, body mass index; BF%, body fat percentage; WHR, waist-to-hip ratio; HC, hip circumference; WC, waist circumference; SBP, systolic blood pressure; DBP, diastolic blood pressure; TG, triglyceride; TC, total cholesterol; HDL-C, high-density lipoprotein cholesterol; LDL-C, low-density lipoprotein cholesterol; MVPA, moderate to vigorous physical activity; VPA, vigorous physical activity; T1D, type 1 diabetes; T2D, type 2 diabetes; SE, standard error; MR, Mendelian randomization.

Table S14. UVMR estimating the associations of candidate mediators with DKD

| **Candidate mediators** | **Outcome** | **Method** | **No. of SNPs** | **β (95% CI)** | **OR (95% CI)** | **P value** |
| --- | --- | --- | --- | --- | --- | --- |
| **BMI** | **DKD** | MR Egger | 763 | 1.1475(0.8048,1.4901) | 3.1502(2.2363,4.4375) | 9.69E-11 |
|  | **DKD** | Weighted median | 763 | 1.0213(0.8125,1.2300) | 2.7767(2.2535,3.4213) | 9.04E-22 |
|  | **DKD** | **Inverse variance weighted** | **763** | **0.9091(0.7876,1.0306)** | **2.4821(2.1981,2.8027)** | **1.04E-48** |
|  | **DKD** | Maximum likelihood | 763 | 0.9101(0.7878,1.0324) | 2.4846(2.1986,2.8077) | 3.27E-48 |
|  | **DKD** | cML-MA | 763 | 0.9315(0.7697,1.0933) | 2.5382(2.1591,2.9840) | 1.58E-29 |
|  | **DKD** | GSMR | 753 | 0.9698(0.8399,1.0996) | 2.6373(2.3161,3.0030) | 1.64E-48 |
| **BF%** | **DKD** | MR Egger | 6 | 1.6269(-1.7428,4.9967) | 5.0882(0.1750,147.9193) | 3.98E-01 |
|  | **DKD** | Weighted median | 6 | 1.4461(0.6584,2.2338) | 4.2463(1.9316,9.3350) | 3.21E-04 |
|  | **DKD** | **Inverse variance weighted** | **6** | **1.5353(0.9316,2.1390)** | **4.6427(2.5386,8.4905)** | **6.20E-07** |
|  | **DKD** | Maximum likelihood | 6 | 1.5668(0.9134,2.2202) | 4.7912(2.4927,9.2091) | 2.60E-06 |
|  | **DKD** | cML-MA | 6 | 1.5917(0.7816,2.4018) | 4.9123(2.1851,11.0434) | 1.18E-04 |
|  | **DKD** | GSMR | NA | NA | NA | NA |
| **WHR** | **DKD** | MR Egger | 18 | 0.6940(-0.9033,2.2914) | 2.0017(0.4052,9.8884) | 4.07E-01 |
|  | **DKD** | Weighted median | 18 | 1.0861(0.6402,1.5321) | 2.9627(1.8968,4.6278) | 1.81E-06 |
|  | **DKD** | **Inverse variance weighted** | **18** | **1.2121(0.8890,1.5351)** | **3.3604(2.4326,4.6419)** | **1.94E-13** |
|  | **DKD** | Maximum likelihood | 18 | 1.2260(0.8932,1.5588) | 3.4075(2.4429,4.7530) | 5.18E-13 |
|  | **DKD** | cML-MA | 18 | 1.2202(0.8447,1.5957) | 3.3879(2.3274,4.9316) | 1.89E-10 |
|  | **DKD** | GSMR | 18 | 1.3011(0.9443,1.6580) | 3.6734(2.5710,5.2485) | 8.88E-13 |
| **HC** | **DKD** | MR Egger | 76 | -0.1652(-0.9749,0.6445) | 0.8477(0.3772,1.9050) | 6.90E-01 |
|  | **DKD** | Weighted median | 76 | -0.2888(-0.5671,-0.0106) | 0.7491(0.5672,0.9895) | 4.19E-02 |
|  | **DKD** | **Inverse variance weighted** | **76** | **-0.2965(-0.4960,-0.0970)** | **0.7434(0.6090,0.9076)** | **3.58E-03** |
|  | **DKD** | Maximum likelihood | 76 | -0.2999(-0.5010,-0.0988) | 0.7409(0.6059,0.9059) | 3.47E-03 |
|  | **DKD** | cML-MA | 76 | -0.3049(-0.5478,-0.0620) | 0.7372(0.5782,0.9398) | 1.39E-02 |
|  | **DKD** | GSMR | 76 | -0.3648(-0.6017,-0.1278) | 0.6944(0.5479,0.8800) | 2.55E-03 |
| **WC** | **DKD** | MR Egger | 58 | -0.5753(-1.6869,0.5362) | 0.5625(0.1851,1.7095) | 3.15E-01 |
|  | **DKD** | Weighted median | 58 | -0.0856(-0.4447,0.2734) | 0.9179(0.6410,1.3145) | 6.40E-01 |
|  | **DKD** | **Inverse variance weighted** | **58** | **-0.0323(-0.2891,0.2246)** | **0.9682(0.7489,1.2518)** | **8.05E-01** |
|  | **DKD** | Maximum likelihood | 58 | -0.0323(-0.2919,0.2273) | 0.9682(0.7469,1.2552) | 8.07E-01 |
|  | **DKD** | cML-MA | 58 | -0.0323(-0.3534,0.2888) | 0.9682(0.7023,1.3348) | 8.44E-01 |
|  | **DKD** | GSMR | 56 | 0.0077(-0.2941,0.3095) | 1.0077(0.7452,1.3627) | 9.60E-01 |
| **Cigarettes smoked per day** | **DKD** | MR Egger | 25 | -0.1154(-0.3976,0.1668) | 0.8910(0.6719,1.1816) | 4.31E-01 |
|  | **DKD** | Weighted median | 25 | 0.0470(-0.1805,0.2745) | 1.0481(0.8349,1.3159) | 6.85E-01 |
|  | **DKD** | **Inverse variance weighted** | **25** | **0.0704(-0.0907,0.2315)** | **1.0729(0.9133,1.2605)** | **3.92E-01** |
|  | **DKD** | Maximum likelihood | 25 | 0.0710(-0.0909,0.2329) | 1.0736(0.9131,1.2623) | 3.90E-01 |
|  | **DKD** | cML-MA | 25 | 0.0801(-0.1397,0.2999) | 1.0834(0.8696,1.3498) | 4.75E-01 |
|  | **DKD** | GSMR | 25 | 0.1293(-0.1929,0.4515) | 1.1380(0.8245,1.5706) | 4.32E-01 |
| **Pack years of smoking** | **DKD** | MR Egger | 10 | -0.0633(-1.1194,0.9928) | 0.9387(0.3265,2.6987) | 9.09E-01 |
|  | **DKD** | Weighted median | 10 | 0.0936(-0.4773,0.6646) | 1.0982(0.6204,1.9437) | 7.48E-01 |
|  | **DKD** | **Inverse variance weighted** | **10** | **0.1361(-0.3299,0.6021)** | **1.1458(0.7190,1.8259)** | **5.67E-01** |
|  | **DKD** | Maximum likelihood | 10 | 0.1375(-0.3307,0.6057) | 1.1474(0.7184,1.8325) | 5.65E-01 |
|  | **DKD** | cML-MA | 10 | 0.1214(-0.4128,0.6556) | 1.1291(0.6618,1.9263) | 6.56E-01 |
|  | **DKD** | GSMR | 10 | 0.1258(-0.3083,0.5599) | 1.1341(0.7347,1.7505) | 5.70E-01 |
| **Maternal smoking around birth** | **DKD** | MR Egger | 5 | 2.4967(-13.9115,18.9049) | 12.1423(9.09E-07,1.62E+08) | 7.85E-01 |
|  | **DKD** | Weighted median | 5 | -0.8607(-4.2113,2.4899) | 0.4229(0.0148,12.0600) | 6.15E-01 |
|  | **DKD** | **Inverse variance weighted** | **5** | **-0.1705(-2.8849,2.5439)** | **0.8432(0.0559,12.7291)** | **9.02E-01** |
|  | **DKD** | Maximum likelihood | 5 | -0.1751(-2.9257,2.5755) | 0.8394(0.0536,13.1375) | 9.01E-01 |
|  | **DKD** | cML-MA | 5 | -0.1005(-3.1949,2.9940) | 0.9044(0.0410,19.9662) | 9.49E-01 |
|  | **DKD** | GSMR | NA | NA | NA | NA |
| **Smoking initiation** | **DKD** | MR Egger | 84 | -0.2575(-1.2755,0.7606) | 0.7730(0.2793,2.1395) | 6.21E-01 |
|  | **DKD** | Weighted median | 84 | 0.2032(-0.1175,0.5239) | 1.2254(0.8892,1.6886) | 2.14E-01 |
|  | **DKD** | **Inverse variance weighted** | **84** | **0.2048(-0.0052,0.4148)** | **1.2273(0.9948,1.5141)** | **5.60E-02** |
|  | **DKD** | Maximum likelihood | 84 | 0.2086(-0.0039,0.4211) | 1.2320(0.9961,1.5236) | 5.43E-02 |
|  | **DKD** | cML-MA | 84 | 0.2217(-0.0775,0.5210) | 1.2482(0.9254,1.6837) | 1.46E-01 |
|  | **DKD** | GSMR | 84 | 0.4046(-0.0153,0.8245) | 1.4987(0.9849,2.2807) | 5.89E-02 |
| **Age of smoking initiation** | **DKD** | MR Egger | 5 | 0.1572(-2.8699,3.1844) | 1.1703(0.0567,24.1522) | 9.25E-01 |
|  | **DKD** | Weighted median | 5 | 0.2027(-1.1799,1.5854) | 1.2247(0.3073,4.8810) | 7.74E-01 |
|  | **DKD** | **Inverse variance weighted** | **5** | **0.4087(-0.7451,1.5626)** | **1.5049(0.4747,4.7710)** | **4.87E-01** |
|  | **DKD** | Maximum likelihood | 5 | 0.4154(-0.7483,1.5792) | 1.5150(0.4732,4.8510) | 4.84E-01 |
|  | **DKD** | cML-MA | 5 | 0.4276(-0.8099,1.6651) | 1.5335(0.4449,5.2860) | 4.98E-01 |
|  | **DKD** | GSMR | NA | NA | NA | NA |
| **Alcoholic drinking** | **DKD** | MR Egger | 32 | 0.2668(-1.0052,1.5387) | 1.3057(0.3660,4.6586) | 6.84E-01 |
|  | **DKD** | Weighted median | 32 | 0.2841(-0.5677,1.1359) | 1.3286(0.5668,3.1141) | 5.13E-01 |
|  | **DKD** | **Inverse variance weighted** | **32** | **-0.0817(-0.6565,0.4931)** | **0.9215(0.5187,1.6373)** | **7.81E-01** |
|  | **DKD** | Maximum likelihood | 32 | -0.0829(-0.6625,0.4967) | 0.9204(0.5156,1.6432) | 7.79E-01 |
|  | **DKD** | cML-MA | 32 | -0.0901(-0.8440,0.6638) | 0.9138(0.4300,1.9422) | 8.15E-01 |
|  | **DKD** | GSMR | 32 | -0.1142(-0.7003,0.4718) | 0.8920(0.4964,1.6029) | 7.02E-01 |
| **Coffee intake** | **DKD** | MR Egger | 32 | 1.0881(0.0160,2.1601) | 2.9685(1.0161,8.6724) | 5.59E-02 |
|  | **DKD** | Weighted median | 32 | 1.3845(0.5490,2.2199) | 3.9927(1.7315,9.2068) | 1.16E-03 |
|  | **DKD** | **Inverse variance weighted** | **32** | **1.4898(0.9277,2.0519)** | **4.4362(2.5286,7.7828)** | **2.05E-07** |
|  | **DKD** | Maximum likelihood | 32 | 1.5165(0.9505,2.0826) | 4.5564(2.5870,8.0249) | 1.51E-07 |
|  | **DKD** | cML-MA | 32 | 1.5383(0.7788,2.2978) | 4.6567(2.1788,9.9527) | 7.20E-05 |
|  | **DKD** | GSMR | 32 | 1.1167(0.6931,1.5403) | 3.0547(1.9999,4.6660) | 2.38E-07 |
| **SBP** | **DKD** | MR Egger | 604 | 0.0025(-0.0168,0.0217) | 1.0025(0.9834,1.0219) | 8.01E-01 |
|  | **DKD** | Weighted median | 604 | 0.0182(0.0065,0.0298) | 1.0183(1.0066,1.0302) | 2.19E-03 |
|  | **DKD** | **Inverse variance weighted** | **604** | **0.0219(0.0145,0.0292)** | **1.0221(1.0146,1.0297)** | **5.48E-09** |
|  | **DKD** | Maximum likelihood | 604 | 0.0219(0.0145,0.0293) | 1.0221(1.0146,1.0297) | 6.90E-09 |
|  | **DKD** | cML-MA | 604 | 0.0227(0.0125,0.0329) | 1.0230(1.0126,1.0335) | 1.27E-05 |
|  | **DKD** | GSMR | 604 | 0.4090(0.2718,0.5462) | 1.5054(1.3124,1.7268) | 5.11E-09 |
| **DBP** | **DKD** | MR Egger | 612 | 0.0171(-0.0154,0.0495) | 1.0172(0.9847,1.0508) | 3.04E-01 |
|  | **DKD** | Weighted median | 612 | 0.0171(-0.0034,0.0375) | 1.0172(0.9966,1.0382) | 1.02E-01 |
|  | **DKD** | **Inverse variance weighted** | **612** | **0.0163(0.0036,0.0290)** | **1.0164(1.0036,1.0294)** | **1.20E-02** |
|  | **DKD** | Maximum likelihood | 612 | 0.0164(0.0036,0.0292) | 1.0165(1.0036,1.0296) | 1.20E-02 |
|  | **DKD** | cML-MA | 612 | 0.0162(-0.0010,0.0334) | 1.0163(0.9990,1.0339) | 6.46E-02 |
|  | **DKD** | GSMR | 610 | 0.1785(0.0412,0.3158) | 1.1954(1.0420,1.3713) | 1.08E-02 |
| **TG** | **DKD** | MR Egger | 60 | -0.0252(-0.2425,0.1921) | 0.9751(0.7846,1.2118) | 8.21E-01 |
|  | **DKD** | Weighted median | 60 | 0.0279(-0.1926,0.2485) | 1.0283(0.8248,1.2821) | 8.04E-01 |
|  | **DKD** | **Inverse variance weighted** | **60** | **0.0524(-0.0852,0.1900)** | **1.0538(0.9183,1.2093)** | **4.56E-01** |
|  | **DKD** | Maximum likelihood | 60 | 0.0528(-0.0853,0.1909) | 1.0542(0.9182,1.2103) | 4.54E-01 |
|  | **DKD** | cML-MA | 60 | 0.0551(-0.1317,0.2419) | 1.0566(0.8766,1.2736) | 5.63E-01 |
|  | **DKD** | GSMR | 60 | 0.0495(-0.0959,0.1949) | 1.0507(0.9085,1.2152) | 5.05E-01 |
| **TC** | **DKD** | MR Egger | 94 | -0.1518(-0.3255,0.0218) | 0.8591(0.7222,1.0221) | 9.00E-02 |
|  | **DKD** | Weighted median | 94 | -0.1732(-0.3435,-0.0030) | 0.8409(0.7093,0.9970) | 4.61E-02 |
|  | **DKD** | **Inverse variance weighted** | **94** | **-0.0893(-0.1949,0.0163)** | **0.9146(0.8229,1.0165)** | **9.76E-02** |
|  | **DKD** | Maximum likelihood | 94 | -0.0893(-0.1953,0.0167) | 0.9146(0.8226,1.0168) | 9.87E-02 |
|  | **DKD** | cML-MA | 94 | -0.1141(-0.2569,0.0286) | 0.8921(0.7734,1.0290) | 1.17E-01 |
|  | **DKD** | GSMR | 92 | -0.0951(-0.2183,0.0280) | 0.9093(0.8039,1.0284) | 1.30E-01 |
| **HDL-C** | **DKD** | MR Egger | 99 | 0.1671(-0.0414,0.3755) | 1.1818(0.9595,1.4558) | 1.19E-01 |
|  | **DKD** | Weighted median | 99 | -0.0181(-0.1938,0.1575) | 0.9820(0.8238,1.1706) | 8.40E-01 |
|  | **DKD** | **Inverse variance weighted** | **99** | **-0.0815(-0.1966,0.0337)** | **0.9218(0.8215,1.0342)** | **1.65E-01** |
|  | **DKD** | Maximum likelihood | 99 | -0.0819(-0.1975,0.0337) | 0.9214(0.8208,1.0343) | 1.65E-01 |
|  | **DKD** | cML-MA | 99 | -0.0895(-0.2507,0.0717) | 0.9144(0.7783,1.0743) | 2.77E-01 |
|  | **DKD** | GSMR | 99 | -0.0889(-0.2060,0.0281) | 0.9149(0.8139,1.0285) | 1.36E-01 |
| **LDL-C** | **DKD** | MR Egger | 77 | -0.1234(-0.2738,0.0269) | 0.8839(0.7605,1.0273) | 1.12E-01 |
|  | **DKD** | Weighted median | 77 | -0.1698(-0.3168,-0.0227) | 0.8439(0.7285,0.9775) | 2.36E-02 |
|  | **DKD** | **Inverse variance weighted** | **77** | **-0.1316(-0.2313,-0.0319)** | **0.8767(0.7935,0.9686)** | **9.70E-03** |
|  | **DKD** | Maximum likelihood | 77 | -0.1309(-0.2309,-0.0309) | 0.8773(0.7938,0.9696) | 1.03E-02 |
|  | **DKD** | cML-MA | 77 | -0.1416(-0.2620,-0.0212) | 0.8679(0.7695,0.9790) | 2.11E-02 |
|  | **DKD** | GSMR | 76 | -0.1475(-0.2608,-0.0342) | 0.8629(0.7705,0.9664) | 1.07E-02 |
| **Fasting glucose** | **DKD** | MR Egger | 25 | 0.1644(-0.6038,0.9327) | 1.1787(0.5467,2.5413) | 6.79E-01 |
|  | **DKD** | Weighted median | 25 | 0.3077(-0.2428,0.8581) | 1.3603(0.7845,2.3587) | 2.73E-01 |
|  | **DKD** | **Inverse variance weighted** | **25** | **0.5807(0.2029,0.9585)** | **1.7873(1.2249,2.6079)** | **2.59E-03** |
|  | **DKD** | Maximum likelihood | 25 | 0.5936(0.2141,0.9730) | 1.8104(1.2388,2.6459) | 2.17E-03 |
|  | **DKD** | cML-MA | 25 | 0.5775(0.0843,1.0707) | 1.7816(1.0880,2.9174) | 2.17E-02 |
|  | **DKD** | GSMR | 25 | 0.3455(0.1214,0.5695) | 1.4126(1.1290,1.7675) | 2.52E-03 |
| **Fasting insulin** | **DKD** | MR Egger | 13 | 0.5423(-4.5307,5.6154) | 1.7200(0.0108,274.6120) | 8.38E-01 |
|  | **DKD** | Weighted median | 13 | 2.4039(1.1641,3.6436) | 11.0658(3.2031,38.2293) | 1.44E-04 |
|  | **DKD** | **Inverse variance weighted** | **13** | **1.9131(0.9458,2.8804)** | **6.7742(2.5748,17.8222)** | **1.06E-04** |
|  | **DKD** | Maximum likelihood | 13 | 1.9842(0.9950,2.9735) | 7.2734(2.7046,19.5599) | 8.45E-05 |
|  | **DKD** | cML-MA | 13 | 2.0747(0.9098,3.2396) | 7.9622(2.4839,25.5233) | 4.82E-04 |
|  | **DKD** | GSMR | 13 | 0.9835(0.5557,1.4114) | 2.6739(1.7431,4.1016) | 6.62E-06 |
| **MVPA** | **DKD** | MR Egger | 17 | -3.7716(-9.3496,1.8065) | 0.0230(0.0001,6.0891) | 2.05E-01 |
|  | **DKD** | Weighted median | 17 | -0.6297(-1.7919,0.5325) | 0.5328(0.1666,1.7032) | 2.88E-01 |
|  | **DKD** | **Inverse variance weighted** | **17** | **-0.8894(-1.7162,-0.0626)** | **0.4109(0.1797,0.9393)** | **3.50E-02** |
|  | **DKD** | Maximum likelihood | 17 | -0.8953(-1.7338,-0.0567) | 0.4085(0.1766,0.9449) | 3.64E-02 |
|  | **DKD** | cML-MA | 17 | -0.9085(-1.9332,0.1162) | 0.4031(0.1447,1.1232) | 8.22E-02 |
|  | **DKD** | GSMR | 17 | -0.8547(-1.6509,-0.0586) | 0.4254(0.1919,0.9431) | 3.54E-02 |
| **VPA** | **DKD** | MR Egger | 6 | 11.1687(-8.0434,30.3808) | 70875.1005(3.21E-04,1.56E+13) | 3.18E-01 |
|  | **DKD** | Weighted median | 6 | 0.5999(-2.1972,3.3969) | 1.8219(0.1111,29.8727) | 6.74E-01 |
|  | **DKD** | **Inverse variance weighted** | **6** | **-0.2071(-2.6821,2.2678)** | **0.8129(0.0684,9.6584)** | **8.70E-01** |
|  | **DKD** | Maximum likelihood | 6 | -0.2129(-2.7253,2.2996) | 0.8083(0.0655,9.9701) | 8.68E-01 |
|  | **DKD** | cML-MA | 6 | -0.0482(-2.9738,2.8775) | 0.9530(0.0511,17.7693) | 9.74E-01 |
|  | **DKD** | GSMR | NA | NA | NA | NA |
| **Sedentary behavior** | **DKD** | MR Egger | 7 | -0.4118(-2.2617,1.4381) | 0.6625(0.1042,4.2128) | 6.81E-01 |
|  | **DKD** | Weighted median | 7 | -0.0612(-0.7069,0.5845) | 0.9406(0.4932,1.7942) | 8.53E-01 |
|  | **DKD** | **Inverse variance weighted** | **7** | **-0.0616(-0.5844,0.4613)** | **0.9403(0.5574,1.5861)** | **8.17E-01** |
|  | **DKD** | Maximum likelihood | 7 | -0.0619(-0.5861,0.4623) | 0.9400(0.5565,1.5878) | 8.17E-01 |
|  | **DKD** | cML-MA | 7 | -0.0684(-0.6318,0.4950) | 0.9339(0.5316,1.6404) | 8.12E-01 |
|  | **DKD** | GSMR | NA | NA | NA | NA |
| **Household income** | **DKD** | MR Egger | 47 | -0.2650(-2.3267,1.7967) | 0.7672(0.0976,6.0295) | 8.02E-01 |
|  | **DKD** | Weighted median | 47 | -0.4767(-1.0376,0.0841) | 0.6208(0.3543,1.0878) | 9.57E-02 |
|  | **DKD** | **Inverse variance weighted** | **47** | **-0.5956(-0.9919,-0.1993)** | **0.5512(0.3709,0.8193)** | **3.22E-03** |
|  | **DKD** | Maximum likelihood | 47 | -0.5790(-0.9791,-0.1789) | 0.5604(0.3756,0.8362) | 4.56E-03 |
|  | **DKD** | cML-MA | 47 | -0.6244(-1.1240,-0.1247) | 0.5356(0.3250,0.8827) | 1.43E-02 |
|  | **DKD** | GSMR | 47 | -0.7035(-1.1731,-0.2340) | 0.4948(0.3094,0.7914) | 3.32E-03 |
| **T1D** | **DKD** | MR Egger | 28 | 0.1913(0.1153,0.2673) | 1.2108(1.1223,1.3064) | 3.97E-05 |
|  | **DKD** | Weighted median | 28 | 0.1965(0.1295,0.2635) | 1.2171(1.1383,1.3014) | 8.89E-09 |
|  | **DKD** | **Inverse variance weighted** | **28** | **0.1670(0.1226,0.2115)** | **1.1818(1.1304,1.2355)** | **1.75E-13** |
|  | **DKD** | Maximum likelihood | 28 | 0.1679(0.1227,0.2131) | 1.1828(1.1305,1.2376) | 3.47E-13 |
|  | **DKD** | cML-MA | 28 | 0.1737(0.1157,0.2317) | 1.1897(1.1226,1.2608) | 4.44E-09 |
|  | **DKD** | GSMR | 28 | 0.4567(0.3264,0.5870) | 1.5789(1.3860,1.7986) | 6.42E-12 |
| **T2D** | **DKD** | MR Egger | 110 | 0.5546(0.3370,0.7723) | 1.7413(1.4008,2.1647) | 2.27E-06 |
|  | **DKD** | Weighted median | 110 | 0.6120(0.4958,0.7282) | 1.8441(1.6418,2.0714) | 5.73E-25 |
|  | **DKD** | **Inverse variance weighted** | **110** | **0.5782(0.5047,0.6517)** | **1.7828(1.6565,1.9187)** | **1.16E-53** |
|  | **DKD** | Maximum likelihood | 110 | 0.5839(0.5091,0.6587) | 1.7930(1.6638,1.9324) | 8.31E-53 |
|  | **DKD** | cML-MA | 110 | 0.5982(0.4920,0.7044) | 1.8188(1.6356,2.0226) | 2.44E-28 |
|  | **DKD** | GSMR | 109 | 0.7574(0.6597,0.8551) | 2.1327(1.9343,2.3516) | 3.64E-52 |

Abbreviations: BMI, body mass index; BF%, body fat percentage; WHR, waist-to-hip ratio; HC, hip circumference; WC, waist circumference; SBP, systolic blood pressure; DBP, diastolic blood pressure; TG, triglyceride; TC, total cholesterol; HDL-C, high-density lipoprotein cholesterol; LDL-C, low-density lipoprotein cholesterol; MVPA, moderate to vigorous physical activity; VPA, vigorous physical activity; T1D, type 1 diabetes; T2D, type 2 diabetes; DKD, diabetic kidney disease; SNP, single nucleotide polymorphism; OR, odds ratio; CI, confidence interval; GSMR, Generalized Summary-data-based Mendelian Randomization; cML-MA, Constrained Maximum Likelihood and Model Averaging; MR, Mendelian randomization; UVMR, univariable Mendelian randomization.

Table S15. MR heterogeneity test of the associations of candidate mediators with DKD

| **Candidate mediators** | **Outcome** | **Method** | **Q statistic** | **Q df** | **Q p-value** |
| --- | --- | --- | --- | --- | --- |
| BMI | DKD | MR Egger | 675.83 | 761 | 9.88E-01 |
| BMI | DKD | IVW | 677.96 | 762 | 9.87E-01 |
| BF% | DKD | MR Egger | 4.46 | 4 | 3.48E-01 |
| BF% | DKD | IVW | 4.46 | 5 | 4.85E-01 |
| WHR | DKD | MR Egger | 12.05 | 16 | 7.41E-01 |
| WHR | DKD | IVW | 12.47 | 17 | 7.71E-01 |
| HC | DKD | MR Egger | 58.98 | 74 | 8.99E-01 |
| HC | DKD | IVW | 59.09 | 75 | 9.11E-01 |
| WC | DKD | MR Egger | 52.90 | 56 | 5.93E-01 |
| WC | DKD | IVW | 53.87 | 57 | 5.93E-01 |
| Cigarettes smoked per day | DKD | MR Egger | 21.50 | 23 | 5.51E-01 |
| Cigarettes smoked per day | DKD | IVW | 23.97 | 24 | 4.63E-01 |
| Pack years of smoking | DKD | MR Egger | 7.44 | 8 | 4.90E-01 |
| Pack years of smoking | DKD | IVW | 7.61 | 9 | 5.74E-01 |
| Maternal smoking around birth | DKD | MR Egger | 4.84 | 3 | 1.84E-01 |
| Maternal smoking around birth | DKD | IVW | 5.01 | 4 | 2.87E-01 |
| Smoking initiation | DKD | MR Egger | 77.86 | 82 | 6.09E-01 |
| Smoking initiation | DKD | IVW | 78.68 | 83 | 6.14E-01 |
| Age of smoking initiation | DKD | MR Egger | 2.66 | 3 | 4.48E-01 |
| Age of smoking initiation | DKD | IVW | 2.69 | 4 | 6.11E-01 |
| Alcoholic drinking | DKD | MR Egger | 24.97 | 30 | 7.26E-01 |
| Alcoholic drinking | DKD | IVW | 25.34 | 31 | 7.52E-01 |
| Coffee intake | DKD | MR Egger | 30.50 | 30 | 4.41E-01 |
| Coffee intake | DKD | IVW | 31.25 | 31 | 4.53E-01 |
| SBP | DKD | MR Egger | 520.31 | 602 | 9.93E-01 |
| SBP | DKD | IVW | 524.89 | 603 | 9.90E-01 |
| DBP | DKD | MR Egger | 522.55 | 610 | 9.96E-01 |
| DBP | DKD | IVW | 522.55 | 611 | 9.96E-01 |
| TG | DKD | MR Egger | 52.91 | 58 | 6.64E-01 |
| TG | DKD | IVW | 53.73 | 59 | 6.69E-01 |
| TC | DKD | MR Egger | 87.28 | 92 | 6.20E-01 |
| TC | DKD | IVW | 88.07 | 93 | 6.25E-01 |
| HDL-C | DKD | MR Egger | 99.82 | 97 | 4.02E-01 |
| HDL-C | DKD | IVW | 107.69 | 98 | 2.37E-01 |
| LDL-C | DKD | MR Egger | 66.79 | 75 | 7.39E-01 |
| LDL-C | DKD | IVW | 66.81 | 76 | 7.65E-01 |
| Fasting glucose | DKD | MR Egger | 23.01 | 23 | 4.60E-01 |
| Fasting glucose | DKD | IVW | 24.49 | 24 | 4.34E-01 |
| Fasting insulin | DKD | MR Egger | 15.35 | 11 | 1.67E-01 |
| Fasting insulin | DKD | IVW | 15.75 | 12 | 2.03E-01 |
| MVPA | DKD | MR Egger | 12.41 | 15 | 6.48E-01 |
| MVPA | DKD | IVW | 13.46 | 16 | 6.39E-01 |
| VPA | DKD | MR Egger | 5.41 | 4 | 2.48E-01 |
| VPA | DKD | IVW | 7.26 | 5 | 2.02E-01 |
| Sedentary behavior | DKD | MR Egger | 1.50 | 5 | 9.13E-01 |
| Sedentary behavior | DKD | IVW | 1.65 | 6 | 9.49E-01 |
| Household income | DKD | MR Egger | 30.52 | 45 | 9.51E-01 |
| Household income | DKD | IVW | 30.63 | 46 | 9.60E-01 |
| T1D | DKD | MR Egger | 24.10 | 26 | 5.70E-01 |
| T1D | DKD | IVW | 24.70 | 27 | 5.91E-01 |
| T2D | DKD | MR Egger | 91.69 | 108 | 8.70E-01 |
| T2D | DKD | IVW | 91.74 | 109 | 8.83E-01 |

Abbreviations: BMI, body mass index; BF%, body fat percentage; WHR, waist-to-hip ratio; HC, hip circumference; WC, waist circumference; SBP, systolic blood pressure; DBP, diastolic blood pressure; TG, triglyceride; TC, total cholesterol; HDL-C, high-density lipoprotein cholesterol; LDL-C, low-density lipoprotein cholesterol; MVPA, moderate to vigorous physical activity; VPA, vigorous physical activity; T1D, type 1 diabetes; T2D, type 2 diabetes; DKD, diabetic kidney disease; IVW, Inverse variance weighted; df, degree of freedom; MR, Mendelian randomization.

Table S16. MR directional pleiotropy test (MR Egger) of the association candidate mediators with DKD

| **Candidate mediators** | **Outcome** | **Egger intercept** | **SE** | **P-value** |
| --- | --- | --- | --- | --- |
| BMI | DKD | -0.0038 | 0.0026 | 1.45E-01 |
| BF% | DKD | -0.0033 | 0.0607 | 9.59E-01 |
| WHR | DKD | 0.0187 | 0.0289 | 5.26E-01 |
| HC | DKD | -0.0039 | 0.0119 | 7.44E-01 |
| WC | DKD | 0.0141 | 0.0143 | 3.29E-01 |
| Cigarettes smoked per day | DKD | 0.0150 | 0.0095 | 1.30E-01 |
| Pack years of smoking | DKD | 0.0076 | 0.0184 | 6.91E-01 |
| Maternal smoking around birth | DKD | -0.0253 | 0.0780 | 7.67E-01 |
| Smoking initiation | DKD | 0.0122 | 0.0134 | 3.66E-01 |
| Age of smoking initiation | DKD | 0.0061 | 0.0344 | 8.71E-01 |
| Alcoholic drinking | DKD | -0.0064 | 0.0107 | 5.52E-01 |
| Coffee intake | DKD | 0.0079 | 0.0091 | 3.95E-01 |
| SBP | DKD | 0.0060 | 0.0028 | 3.26E-02 |
| DBP | DKD | -0.0001 | 0.0028 | 9.60E-01 |
| TG | DKD | 0.0051 | 0.0057 | 3.69E-01 |
| TC | DKD | 0.0041 | 0.0046 | 3.76E-01 |
| HDL-C | DKD | -0.0142 | 0.0051 | 6.82E-03 |
| LDL-C | DKD | -0.0007 | 0.0049 | 8.88E-01 |
| Fasting glucose | DKD | 0.0138 | 0.0113 | 2.36E-01 |
| Fasting insulin | DKD | 0.0227 | 0.0420 | 6.00E-01 |
| MVPA | DKD | 0.0416 | 0.0406 | 3.22E-01 |
| VPA | DKD | -0.1094 | 0.0935 | 3.07E-01 |
| Sedentary behavior | DKD | 0.0120 | 0.0311 | 7.15E-01 |
| Household income | DKD | -0.0064 | 0.0201 | 7.50E-01 |
| T1D | DKD | -0.0081 | 0.0105 | 4.47E-01 |
| T2D | DKD | 0.0016 | 0.0073 | 8.22E-01 |

Abbreviations: BMI, body mass index; BF%, body fat percentage; WHR, waist-to-hip ratio; HC, hip circumference; WC, waist circumference; SBP, systolic blood pressure; DBP, diastolic blood pressure; TG, triglyceride; TC, total cholesterol; HDL-C, high-density lipoprotein cholesterol; LDL-C, low-density lipoprotein cholesterol; MVPA, moderate to vigorous physical activity; VPA, vigorous physical activity; T1D, type 1 diabetes; T2D, type 2 diabetes; DKD, diabetic kidney disease; SE, standard error; MR, Mendelian randomization.

Table S17. MVMR assessing the causal association between each mediator and DKD with adjustment for education

| **Mediators** | **method** | **Variable** | **β** | **SE** | **P** | **MVMR Instrument validity test** | | | **MVMR Heterogeneity test** | | **MVMR directional pleiotropy**  **test** | | | **NO. of SNPs** |
| --- | --- | --- | --- | --- | --- | --- | --- | --- | --- | --- | --- | --- | --- | --- |
|  |  |  |  |  |  | **F_stastistics** | **Q statistic** | **P value** | **Q statistic** | **P value** | **Egger Intercept** | **SE** | **P value** |  |
| **BMI** | MV-IVW | Education | -0.4573 | 0.1268 | 3.09E-04 | 34.08 | 1269.61 | 9.75E-09 | 1276.46 | 5.16E-09 | -0.0015 | 0.0014 | 2.91E-01 | 1001 |
|  |  | BMI | 0.8619 | 0.0715 | 1.74E-33 |  |  |  |  |  |  |  |  |  |
|  | MVMR-Egger | Education | -0.3123 | 0.1878 | 9.63E-02 |  |  |  | 1274.69 | 5.54E-09 |  |  |  |  |
|  |  | BMI | 0.8451 | 0.0729 | 4.51E-31 |  |  |  |  |  |  |  |  |  |
| **WHR** | MV-IVW | WHR | -0.4579 | 0.1207 | 1.48E-04 | 27.77 | 390.72 | 3.54E-02 | 403.63 | 1.33E-02 | 0.0034 | 0.0052 | 5.08E-01 | 345 |
|  |  | Education | 0.7817 | 0.1396 | 2.14E-08 |  |  |  |  |  |  |  |  |  |
|  | MVMR-Egger | WHR | -0.7216 | 0.4163 | 8.30E-02 |  |  |  | 403.11 | 1.27E-02 |  |  |  |  |
|  |  | Education | 0.7739 | 0.1402 | 3.41E-08 |  |  |  |  |  |  |  |  |  |
| **SBP** | MV-IVW | Education | -0.4730 | 0.1138 | 3.24E-05 | 41.74 | 1144.55 | 6.79E-07 | 1147.07 | 5.88E-07 | 0.0029 | 0.0015 | 5.16E-02 | 925 |
|  |  | SBP | 0.0227 | 0.0041 | 3.91E-08 |  |  |  |  |  |  |  |  |  |
|  | MVMR-Egger | Education | -0.7309 | 0.1747 | 2.86E-05 |  |  |  | 1141.27 | 9.45E-07 |  |  |  |  |
|  |  | SBP | 0.0230 | 0.0041 | 2.45E-08 |  |  |  |  |  |  |  |  |  |
| **DBP** | MV-IVW | Education | -0.5490 | 0.1169 | 2.64E-06 | 43.48 | 1195.49 | 2.29E-08 | 1197.71 | 2.02E-08 | -0.0026 | 0.0015 | 7.28E-02 | 942 |
|  |  | DBP | 0.0142 | 0.0071 | 4.55E-02 |  |  |  |  |  |  |  |  |  |
|  | MVMR-Egger | Education | -0.3100 | 0.1771 | 8.00E-02 |  |  |  | 1193.44 | 2.87E-08 |  |  |  |  |
|  |  | DBP | 0.0137 | 0.0071 | 5.35E-02 |  |  |  |  |  |  |  |  |  |
| **Fasting glucose** | MV-IVW | Fasting glucose | -0.8033 | 0.3467 | 2.05E-02 | 32.82 | 117.95 | 3.95E-04 | 118.88 | 4.25E-04 | -0.0063 | 0.0072 | 3.84E-01 | 74 |
|  |  | Education | 0.6874 | 0.2349 | 3.42E-03 |  |  |  |  |  |  |  |  |  |
|  | MVMR-Egger | Fasting glucose | -0.3279 | 0.6472 | 6.12E-01 |  |  |  | 117.63 | 4.23E-04 |  |  |  |  |
|  |  | Education | 0.6607 | 0.2372 | 5.35E-03 |  |  |  |  |  |  |  |  |  |
| **Fasting insulin** | MV-IVW | Fasting insulin | -0.7235 | 0.3063 | 1.82E-02 | 39.70 | 70.99 | 8.56E-02 | 73.26 | 7.22E-02 | 0.0045 | 0.0086 | 6.02E-01 | 59 |
|  |  | Education | 1.8869 | 0.4989 | 1.55E-04 |  |  |  |  |  |  |  |  |  |
|  | MVMR-Egger | Fasting insulin | -1.0557 | 0.7070 | 1.35E-01 |  |  |  | 72.90 | 6.41E-02 |  |  |  |  |
|  |  | Education | 1.8957 | 0.5024 | 1.61E-04 |  |  |  |  |  |  |  |  |  |
| **T2D** | MV-IVW | T2D | 0.5467 | 0.0380 | 7.37E-47 | 16.71 | 486.02 | 4.89E-02 | 503.19 | 1.55E-02 | -0.0023 | 0.0019 | 2.37E-01 | 439 |
|  |  | Education | -0.2797 | 0.1166 | 1.65E-02 |  |  |  |  |  |  |  |  |  |
|  | MVMR-Egger | T2D | 0.5872 | 0.0512 | 1.76E-30 |  |  |  | 501.58 | 1.61E-02 |  |  |  |  |
|  |  | Education | -0.3340 | 0.1253 | 7.69E-03 |  |  |  |  |  |  |  |  |  |

Abbreviations: BMI, body mass index; WHR, waist-to-hip ratio; SBP, systolic blood pressure; DBP, diastolic blood pressure; T2D, type 2 diabetes; DKD, diabetic kidney disease; SE, standard error; SNP, single nucleotide polymorphism; MV-IVW, multivariable inverse variance weighted; MVMR, multivariable Mendelian randomization.

Table S18. UVMR estimating the associations of candidate mediators with CKD

| **Candidate mediators** | **Outcome** | **Method** | **No. of SNPs** | **β (95% CI)** | **OR (95% CI)** | **P value** |
| --- | --- | --- | --- | --- | --- | --- |
| **BMI** | **CKD** | MR Egger | 763 | 0.2887(0.0517,0.5257) | 1.3347(1.0530,1.6916) | 1.72E-02 |
|  | **CKD** | Weighted median | 763 | 0.3469(0.1975,0.4962) | 1.4146(1.2184,1.6425) | 5.31E-06 |
|  | **CKD** | **Inverse variance weighted** | **763** | **0.3800(0.2971,0.4629)** | **1.4623(1.3460,1.5887)** | **2.56E-19** |
|  | **CKD** | Maximum likelihood | 763 | 0.3807(0.2974,0.4641) | 1.4633(1.3463,1.5905) | 3.49E-19 |
|  | **CKD** | cML-MA | 763 | 0.3827(0.2778,0.4877) | 1.4663(1.3202,1.6286) | 8.93E-13 |
|  | **CKD** | GSMR | 752 | 0.4002(0.3116,0.4888) | 1.4922(1.3657,1.6304) | 8.39E-19 |
| **BF%** | **CKD** | MR Egger | 6 | -1.0303(-6.2247,4.1642) | 0.3569(0.0020,64.3387) | 7.17E-01 |
|  | **CKD** | Weighted median | 6 | 0.8199(0.1858,1.4541) | 2.2703(1.2042,4.2804) | 1.13E-02 |
|  | **CKD** | **Inverse variance weighted** | **6** | **0.7977(0.1890,1.4065)** | **2.2205(1.2081,4.0815)** | **1.02E-02** |
|  | **CKD** | Maximum likelihood | 6 | 0.8288(0.2099,1.4477) | 2.2905(1.2336,4.2531) | 8.67E-03 |
|  | **CKD** | cML-MA | 6 | 0.8710(0.1692,1.5729) | 2.3893(1.1843,4.8205) | 1.50E-02 |
|  | **CKD** | GSMR | NA | NA | NA | NA |
| **WHR** | **CKD** | MR Egger | 22 | 0.2579(-0.7529,1.2686) | 1.2942(0.4710,3.5559) | 6.22E-01 |
|  | **CKD** | Weighted median | 22 | 0.2361(-0.0391,0.5112) | 1.2663(0.9617,1.6673) | 9.26E-02 |
|  | **CKD** | **Inverse variance weighted** | **22** | **0.2903(0.0956,0.4849)** | **1.3368(1.1003,1.6241)** | **3.48E-03** |
|  | **CKD** | Maximum likelihood | 22 | 0.2993(0.1032,0.4953) | 1.3489(1.1087,1.6410) | 2.77E-03 |
|  | **CKD** | cML-MA | 22 | 0.2897(0.0477,0.5317) | 1.3360(1.0488,1.7019) | 1.90E-02 |
|  | **CKD** | GSMR | 22 | 0.3112(0.1014,0.5210) | 1.3651(1.1068,1.6837) | 3.64E-03 |
| **HC** | **CKD** | MR Egger | 77 | 0.4275(-0.1485,1.0036) | 1.5335(0.8620,2.7281) | 1.50E-01 |
|  | **CKD** | Weighted median | 77 | 0.0481(-0.1396,0.2358) | 1.0492(0.8697,1.2659) | 6.16E-01 |
|  | **CKD** | **Inverse variance weighted** | **77** | **0.0089(-0.1266,0.1443)** | **1.0089(0.8811,1.1553)** | **8.98E-01** |
|  | **CKD** | Maximum likelihood | 77 | 0.0090(-0.1274,0.1454) | 1.0091(0.8804,1.1565) | 8.97E-01 |
|  | **CKD** | cML-MA | 77 | 0.0099(-0.1524,0.1723) | 1.0100(0.8586,1.1880) | 9.05E-01 |
|  | **CKD** | GSMR | 76 | -0.0021(-0.1614,0.1573) | 0.9979(0.8509,1.1703) | 9.80E-01 |
| **WC** | **CKD** | MR Egger | 57 | 0.3895(-0.3333,1.1123) | 1.4762(0.7166,3.0414) | 2.95E-01 |
|  | **CKD** | Weighted median | 57 | -0.0449(-0.2872,0.1973) | 0.9561(0.7504,1.2181) | 7.16E-01 |
|  | **CKD** | **Inverse variance weighted** | **57** | **-0.0440(-0.2168,0.1287)** | **0.9569(0.8051,1.1373)** | **6.17E-01** |
|  | **CKD** | Maximum likelihood | 57 | -0.0446(-0.2187,0.1294) | 0.9563(0.8036,1.1382) | 6.15E-01 |
|  | **CKD** | cML-MA | 57 | -0.0514(-0.2485,0.1457) | 0.9499(0.7799,1.1568) | 6.09E-01 |
|  | **CKD** | GSMR | 55 | -0.0557(-0.2591,0.1476) | 0.9458(0.7718,1.1591) | 5.91E-01 |
| **Cigarettes smoked per day** | **CKD** | MR Egger | 23 | 0.2492(0.0462,0.4522) | 1.2830(1.0473,1.5718) | 2.54E-02 |
|  | **CKD** | Weighted median | 23 | 0.2680(0.1063,0.4297) | 1.3073(1.1121,1.5367) | 1.16E-03 |
|  | **CKD** | **Inverse variance weighted** | **23** | **0.2831(0.1627,0.4035)** | **1.3273(1.1767,1.4970)** | **4.03E-06** |
|  | **CKD** | Maximum likelihood | 23 | 0.2883(0.1671,0.4095) | 1.3342(1.1819,1.5060) | 3.11E-06 |
|  | **CKD** | cML-MA | 23 | 0.2798(0.1417,0.4180) | 1.3229(1.1522,1.5190) | 7.20E-05 |
|  | **CKD** | GSMR | 23 | 0.5671(0.3250,0.8093) | 1.7632(1.3840,2.2464) | 4.43E-06 |
| **Pack years of smoking** | **CKD** | MR Egger | 8 | 0.4082(-0.3370,1.1535) | 1.5042(0.7139,3.1692) | 3.24E-01 |
|  | **CKD** | Weighted median | 8 | 0.5294(0.1176,0.9413) | 1.6980(1.1248,2.5632) | 1.17E-02 |
|  | **CKD** | **Inverse variance weighted** | **8** | **0.5318(0.1941,0.8694)** | **1.7019(1.2142,2.3854)** | **2.02E-03** |
|  | **CKD** | Maximum likelihood | 8 | 0.5342(0.1935,0.8748) | 1.7060(1.2135,2.3984) | 2.11E-03 |
|  | **CKD** | cML-MA | 8 | 0.5402(0.1623,0.9180) | 1.7163(1.1762,2.5043) | 5.08E-03 |
|  | **CKD** | GSMR | NA | NA | NA | NA |
| **Maternal smoking around birth** | **CKD** | MR Egger | 5 | 2.2353(-6.5531,11.0238) | 9.3497(0.0014,61316.6823) | 6.52E-01 |
|  | **CKD** | Weighted median | 5 | 2.3770(0.3393,4.4147) | 10.7722(1.4039,82.6540) | 2.22E-02 |
|  | **CKD** | **Inverse variance weighted** | **5** | **2.0239(0.3741,3.6737)** | **7.5675(1.4536,39.3967)** | **1.62E-02** |
|  | **CKD** | Maximum likelihood | 5 | 2.0390(0.3589,3.7190) | 7.6826(1.4318,41.2225) | 1.74E-02 |
|  | **CKD** | cML-MA | 5 | 2.0832(0.3675,3.7989) | 8.0302(1.4442,44.6519) | 1.73E-02 |
|  | **CKD** | GSMR | NA | NA | NA | NA |
| **Smoking initiation** | **CKD** | MR Egger | 80 | 0.1606(-0.5726,0.8939) | 1.1743(0.5641,2.4446) | 6.69E-01 |
|  | **CKD** | Weighted median | 80 | 0.0431(-0.1623,0.2486) | 1.0441(0.8502,1.2822) | 6.81E-01 |
|  | **CKD** | **Inverse variance weighted** | **80** | **0.0533(-0.0939,0.2006)** | **1.0548(0.9103,1.2221)** | **4.78E-01** |
|  | **CKD** | Maximum likelihood | 80 | 0.0545(-0.0942,0.2031) | 1.0560(0.9101,1.2252) | 4.73E-01 |
|  | **CKD** | cML-MA | 80 | 0.0438(-0.1409,0.2285) | 1.0448(0.8685,1.2567) | 6.42E-01 |
|  | **CKD** | GSMR | 80 | 0.1093(-0.1848,0.4035) | 1.1155(0.8312,1.4971) | 4.66E-01 |
| **Age of smoking initiation** | **CKD** | MR Egger | 5 | 0.9066(-1.9411,3.7543) | 2.4759(0.1436,42.7040) | 5.77E-01 |
|  | **CKD** | Weighted median | 5 | -0.6998(-1.7246,0.3249) | 0.4967(0.1783,1.3838) | 1.81E-01 |
|  | **CKD** | **Inverse variance weighted** | **5** | **-0.2379(-1.2838,0.8080)** | **0.7883(0.2770,2.2435)** | **6.56E-01** |
|  | **CKD** | Maximum likelihood | 5 | -0.2478(-1.3152,0.8196) | 0.7805(0.2684,2.2696) | 6.49E-01 |
|  | **CKD** | cML-MA | 5 | -0.3863(-1.4589,0.6862) | 0.6796(0.2325,1.9862) | 4.80E-01 |
|  | **CKD** | GSMR | NA | NA | NA | NA |
| **Alcoholic drinking** | **CKD** | MR Egger | 32 | 0.1676(-0.6994,1.0347) | 1.1825(0.4969,2.8142) | 7.07E-01 |
|  | **CKD** | Weighted median | 32 | 0.2065(-0.3192,0.7322) | 1.2293(0.7267,2.0796) | 4.41E-01 |
|  | **CKD** | **Inverse variance weighted** | **32** | **-0.0086(-0.3864,0.3693)** | **0.9915(0.6795,1.4467)** | **9.65E-01** |
|  | **CKD** | Maximum likelihood | 32 | -0.0086(-0.3885,0.3714) | 0.9915(0.6780,1.4498) | 9.65E-01 |
|  | **CKD** | cML-MA | 32 | 0.0204(-0.4198,0.4607) | 1.0207(0.6572,1.5851) | 9.27E-01 |
|  | **CKD** | GSMR | 32 | -0.0181(-0.4048,0.3687） | 0.9821(0.6671,1.4458） | 9.27E-01 |
| **Coffee intake** | **CKD** | MR Egger | 36 | -0.6126(-1.3217,0.0965) | 0.5419(0.2667,1.1013) | 9.95E-02 |
|  | **CKD** | Weighted median | 36 | -0.2974(-0.8021,0.2072) | 0.7427(0.4484,1.2302) | 2.48E-01 |
|  | **CKD** | **Inverse variance weighted** | **36** | **-0.2243(-0.5902,0.1417)** | **0.7991(0.5542,1.1522)** | **2.30E-01** |
|  | **CKD** | Maximum likelihood | 36 | -0.2267(-0.5945,0.1411) | 0.7971(0.5518,1.1515) | 2.27E-01 |
|  | **CKD** | cML-MA | 36 | -0.2524(-0.6808,0.1760) | 0.7769(0.5062,1.1924) | 2.48E-01 |
|  | **CKD** | GSMR | 36 | -0.1680(-0.4430,0.1070) | 0.8453(0.6421,1.1129) | 2.31E-01 |
| **SBP** | **CKD** | MR Egger | 614 | 0.0298(0.0164,0.0433) | 1.0303(1.0165,1.0443) | 1.64E-05 |
|  | **CKD** | Weighted median | 614 | 0.0198(0.0121,0.0275) | 1.0200(1.0121,1.0279) | 5.27E-07 |
|  | **CKD** | **Inverse variance weighted** | **614** | **0.0196(0.0146,0.0246)** | **1.0198(1.0147,1.0249)** | **1.88E-14** |
|  | **CKD** | Maximum likelihood | 614 | 0.0196(0.0146,0.0247) | 1.0198(1.0147,1.0250) | 2.55E-14 |
|  | **CKD** | cML-MA | 614 | 0.0197(0.0130,0.0265) | 1.0199(1.0130,1.0269) | 1.18E-08 |
|  | **CKD** | GSMR | 614 | 0.3655(0.2718,0.4592) | 1.4412(1.3124,1.5827) | 2.03E-14 |
| **DBP** | **CKD** | MR Egger | 610 | 0.0172(-0.0067,0.0411) | 1.0174(0.9933,1.0420) | 1.59E-01 |
|  | **CKD** | Weighted median | 610 | 0.0181(0.0045,0.0317) | 1.0183(1.0045,1.0322) | 9.13E-03 |
|  | **CKD** | **Inverse variance weighted** | **610** | **0.0171(0.0082,0.0259)** | **1.0172(1.0082,1.0263)** | **1.65E-04** |
|  | **CKD** | Maximum likelihood | 610 | 0.0171(0.0082,0.0260) | 1.0173(1.0082,1.0264) | 1.75E-04 |
|  | **CKD** | cML-MA | 610 | 0.0178(0.0064,0.0292) | 1.0179(1.0064,1.0296) | 2.27E-03 |
|  | **CKD** | GSMR | 607 | 0.2025(0.1062,0.2988) | 1.2245(1.1121,1.3482) | 3.73E-05 |
| **TG** | **CKD** | MR Egger | 60 | 0.1146(-0.0259,0.2550) | 1.1214(0.9744,1.2905) | 1.15E-01 |
|  | **CKD** | Weighted median | 60 | 0.0920(-0.0355,0.2196) | 1.0964(0.9651,1.2455) | 1.57E-01 |
|  | **CKD** | **Inverse variance weighted** | **60** | **0.0913(0.0014,0.1811)** | **1.0955(1.0014,1.1985)** | **4.66E-02** |
|  | **CKD** | Maximum likelihood | 60 | 0.0919(0.0017,0.1820) | 1.0962(1.0017,1.1996) | 4.58E-02 |
|  | **CKD** | cML-MA | 60 | 0.0915(-0.0157,0.1987) | 1.0958(0.9845,1.2198) | 9.42E-02 |
|  | **CKD** | GSMR | 60 | 0.0951(0.0010,0.1892) | 1.0998(1.0010,1.2082) | 4.75E-02 |
| **TC** | **CKD** | MR Egger | 98 | -0.1274(-0.2682,0.0134) | 0.8804(0.7648,1.0135) | 7.94E-02 |
|  | **CKD** | Weighted median | 98 | -0.1231(-0.2384,-0.0078) | 0.8841(0.7879,0.9922) | 3.63E-02 |
|  | **CKD** | **Inverse variance weighted** | **98** | **-0.1097(-0.1839,-0.0355)** | **0.8961(0.8321,0.9651)** | **3.75E-03** |
|  | **CKD** | Maximum likelihood | 98 | -0.1105(-0.1849,-0.0360) | 0.8954(0.8312,0.9646) | 3.64E-03 |
|  | **CKD** | cML-MA | 98 | -0.1087(-0.2029,-0.0145) | 0.8970(0.8163,0.9856) | 2.37E-02 |
|  | **CKD** | GSMR | 96 | -0.1230(-0.2067,-0.0393) | 0.8843(0.8132,0.9615) | 3.98E-03 |
| **HDL-C** | **CKD** | MR Egger | 99 | -0.0375(-0.1989,0.1239) | 0.9632(0.8196,1.1319) | 6.50E-01 |
|  | **CKD** | Weighted median | 99 | -0.0352(-0.1587,0.0883) | 0.9655(0.8533,1.0924) | 5.77E-01 |
|  | **CKD** | **Inverse variance weighted** | **99** | **-0.0793(-0.1603,0.0017)** | **0.9238(0.8519,1.0017)** | **5.51E-02** |
|  | **CKD** | Maximum likelihood | 99 | -0.0793(-0.1605,0.0020) | 0.9238(0.8517,1.0020) | 5.60E-02 |
|  | **CKD** | cML-MA | 99 | -0.0756(-0.1709,0.0198) | 0.9272(0.8429,1.0200) | 1.20E-01 |
|  | **CKD** | GSMR | 99 | -0.0810(-0.1668,0.0049) | 0.9222(0.8464,1.0049) | 6.45E-02 |
| **LDL-C** | **CKD** | MR Egger | 85 | -0.0394(-0.1500,0.0711) | 0.9613(0.8607,1.0737) | 4.87E-01 |
|  | **CKD** | Weighted median | 85 | -0.0797(-0.1894,0.0300) | 0.9234(0.8274,1.0305) | 1.54E-01 |
|  | **CKD** | **Inverse variance weighted** | **85** | **-0.0661(-0.1355,0.0032)** | **0.9360(0.8733,1.0032)** | **6.16E-02** |
|  | **CKD** | Maximum likelihood | 85 | -0.0658(-0.1353,0.0038) | 0.9363(0.8734,1.0038) | 6.38E-02 |
|  | **CKD** | cML-MA | 85 | -0.0645(-0.1493,0.0204) | 0.9376(0.8613,1.0206) | 1.36E-01 |
|  | **CKD** | GSMR | 84 | -0.0743(-0.1522,0.0037) | 0.9284(0.8588,1.0037） | 6.18E-02 |
| **Fasting glucose** | **CKD** | MR Egger | 27 | 0.4086(-0.1199,0.9371) | 1.5047(0.8870,2.5527) | 1.42E-01 |
|  | **CKD** | Weighted median | 27 | 0.3094(-0.0642,0.6830) | 1.3626(0.9378,1.9798) | 1.05E-01 |
|  | **CKD** | **Inverse variance weighted** | **27** | **0.3389(0.0893,0.5885)** | **1.4033(1.0934,1.8012)** | **7.79E-03** |
|  | **CKD** | Maximum likelihood | 27 | 0.3407(0.0904,0.5910) | 1.4059(1.0946,1.8057) | 7.63E-03 |
|  | **CKD** | cML-MA | 27 | 0.3511(0.0748,0.6275) | 1.4207(1.0777,1.8729) | 1.27E-02 |
|  | **CKD** | GSMR | 27 | 0.1921(0.0402,0.3440) | 1.2118(1.0411,1.4105) | 1.32E-02 |
| **Fasting insulin** | **CKD** | MR Egger | 13 | 0.5295(-2.2987,3.3577) | 1.6981(0.1004,28.7224) | 7.21E-01 |
|  | **CKD** | Weighted median | 13 | 0.2047(-0.5622,0.9717) | 1.2272(0.5699,2.6424) | 6.01E-01 |
|  | **CKD** | **Inverse variance weighted** | **13** | **0.2206(-0.3316,0.7728)** | **1.2468(0.7178,2.1657)** | **4.34E-01** |
|  | **CKD** | Maximum likelihood | 13 | 0.2264(-0.3310,0.7837) | 1.2540(0.7182,2.1895) | 4.26E-01 |
|  | **CKD** | cML-MA | 13 | 0.2463(-0.4084,0.9010) | 1.2793(0.6647,2.4621) | 4.61E-01 |
|  | **CKD** | GSMR | 13 | 0.1193(-0.1500,0.3886) | 1.1267(0.8607,1.4749) | 3.85E-01 |
| **MVPA** | **CKD** | MR Egger | 17 | -2.1584(-5.9543,1.6376) | 0.1155(0.0026,5.1426) | 2.83E-01 |
|  | **CKD** | Weighted median | 17 | 0.1271(-0.6417,0.8960) | 1.1355(0.5264,2.4497) | 7.46E-01 |
|  | **CKD** | **Inverse variance weighted** | **17** | **0.0966(-0.4651,0.6582)** | **1.1014(0.6281,1.9314)** | **7.36E-01** |
|  | **CKD** | Maximum likelihood | 17 | 0.0999(-0.4676,0.6674) | 1.1051(0.6265,1.9493) | 7.30E-01 |
|  | **CKD** | cML-MA | 17 | 0.0802(-0.5759,0.7363) | 1.0836(0.5622,2.0883) | 8.11E-01 |
|  | **CKD** | GSMR | 17 | 0.0918(-0.4480,0.6316) | 1.0961(0.6389,1.8806) | 7.39E-01 |
| **VPA** | **CKD** | MR Egger | 6 | 4.5482(-6.6853,15.7817) | 94.4625(0.0012,7143534.7620) | 4.72E-01 |
|  | **CKD** | Weighted median | 6 | 1.0283(-0.7364,2.7930) | 2.7963(0.4788,16.3292) | 2.53E-01 |
|  | **CKD** | **Inverse variance weighted** | **6** | **0.7484(-0.6473,2.1442)** | **2.1137(0.5235,8.5349)** | **2.93E-01** |
|  | **CKD** | Maximum likelihood | 6 | 0.7622(-0.6455,2.1699) | 2.1430(0.5244,8.7575) | 2.89E-01 |
|  | **CKD** | cML-MA | 6 | 0.8363(-0.8255,2.4981) | 2.3078(0.4380,12.1598) | 3.24E-01 |
|  | **CKD** | GSMR | NA | NA | NA | NA |
| **Sedentary behavior** | **CKD** | MR Egger | 7 | -0.3368(-1.5952,0.9217) | 0.7141(0.2029,2.5135) | 6.22E-01 |
|  | **CKD** | Weighted median | 7 | -0.0496(-0.4922,0.3930) | 0.9516(0.6113,1.4814) | 8.26E-01 |
|  | **CKD** | **Inverse variance weighted** | **7** | **0.0142(-0.3414,0.3698)** | **1.0143(0.7108,1.4474)** | **9.38E-01** |
|  | **CKD** | Maximum likelihood | 7 | 0.0144(-0.3439,0.3728) | 1.0146(0.7090,1.4518) | 9.37E-01 |
|  | **CKD** | cML-MA | 7 | 0.0067(-0.3882,0.4016) | 1.0067(0.6783,1.4942) | 9.74E-01 |
|  | **CKD** | GSMR | NA | NA | NA | NA |
| **Household income** | **CKD** | MR Egger | 43 | -0.4087(-1.8497,1.0323) | 0.6645(0.1573,2.8076) | 5.81E-01 |
|  | **CKD** | Weighted median | 43 | -0.3351(-0.7435,0.0732) | 0.7152(0.4754,1.0760) | 1.08E-01 |
|  | **CKD** | **Inverse variance weighted** | **43** | **-0.5080(-0.7913,-0.2247)** | **0.6017(0.4533,0.7988)** | **4.40E-04** |
|  | **CKD** | Maximum likelihood | 43 | -0.5156(-0.8022,-0.2291) | 0.5971(0.4484,0.7952) | 4.20E-04 |
|  | **CKD** | cML-MA | 43 | -0.4733(-0.8330,-0.1136) | 0.6229(0.4347,0.8926) | 9.90E-03 |
|  | **CKD** | GSMR | 43 | -0.6011(-0.9363,-0.2659) | 0.5482(0.3921,0.7665) | 4.41E-04 |
| **T1D** | **CKD** | MR Egger | 38 | 0.0615(0.0387,0.0842) | 1.0634(1.0395,1.0879) | 6.02E-06 |
|  | **CKD** | Weighted median | 38 | 0.0501(0.0268,0.0733) | 1.0513(1.0272,1.0761) | 2.47E-05 |
|  | **CKD** | **Inverse variance weighted** | **38** | **0.0522(0.0365,0.0678)** | **1.0536(1.0372,1.0702)** | **6.21E-11** |
|  | **CKD** | Maximum likelihood | 38 | 0.0525(0.0367,0.0683) | 1.0539(1.0374,1.0706) | 7.11E-11 |
|  | **CKD** | cML-MA | 38 | 0.0521(0.0311,0.0731) | 1.0535(1.0316,1.0759) | 1.17E-06 |
|  | **CKD** | GSMR | 38 | 0.1704(0.1148,0.2260) | 1.1858(1.1217,1.2535) | 1.87E-09 |
| **T2D** | **CKD** | MR Egger | 111 | 0.1980(0.0889,0.3071) | 1.2190(1.0930,1.3594) | 5.54E-04 |
|  | **CKD** | Weighted median | 111 | 0.1924(0.1155,0.2694) | 1.2122(1.1225,1.3091) | 9.39E-07 |
|  | **CKD** | **Inverse variance weighted** | **111** | **0.1193(0.0725,0.1660)** | **1.1267(1.0752,1.1805)** | **5.62E-07** |
|  | **CKD** | Maximum likelihood | 111 | 0.1206(0.0736,0.1676) | 1.1282(1.0764,1.1824) | 4.91E-07 |
|  | **CKD** | cML-MA | 111 | 0.1243(0.0642,0.1843) | 1.1323(1.0664,1.2023) | 4.94E-05 |
|  | **CKD** | GSMR | 110 | 0.1574(0.0969,0.2180) | 1.1705(1.1017,1.2436) | 3.48E-07 |

Abbreviations: BMI, body mass index; BF%, body fat percentage; WHR, waist-to-hip ratio; HC, hip circumference; WC, waist circumference; SBP, systolic blood pressure; DBP, diastolic blood pressure; TG, triglyceride; TC, total cholesterol; HDL-C, high-density lipoprotein cholesterol; LDL-C, low-density lipoprotein cholesterol; MVPA, moderate to vigorous physical activity; VPA, vigorous physical activity; T1D, type 1 diabetes; T2D, type 2 diabetes; CKD, chronic kidney disease; SNP, single nucleotide polymorphism; OR, odds ratio; CI, confidence interval; GSMR, Generalized Summary-data-based Mendelian Randomization; cML-MA, Constrained Maximum Likelihood and Model Averaging; MR, Mendelian randomization; UVMR, univariable Mendelian randomization.

Table S19. MR heterogeneity test of the associations of candidate mediators with CKD

| **Candidate mediators** | **Outcome** | **Method** | **Q statistic** | **Q df** | **Q p-value** |
| --- | --- | --- | --- | --- | --- |
| BMI | CKD | MR Egger | 602.44 | 761 | 1.00E+00 |
| BMI | CKD | IVW | 603.09 | 762 | 1.00E+00 |
| BF% | CKD | MR Egger | 8.33 | 4 | 8.03E-02 |
| BF% | CKD | IVW | 9.33 | 5 | 9.65E-02 |
| WHR | CKD | MR Egger | 17.50 | 20 | 6.21E-01 |
| WHR | CKD | IVW | 17.50 | 21 | 6.80E-01 |
| HC | CKD | MR Egger | 51.05 | 75 | 9.85E-01 |
| HC | CKD | IVW | 53.20 | 76 | 9.78E-01 |
| WC | CKD | MR Egger | 39.39 | 55 | 9.44E-01 |
| WC | CKD | IVW | 40.86 | 56 | 9.36E-01 |
| Cigarettes smoked per day | CKD | MR Egger | 9.76 | 21 | 9.82E-01 |
| Cigarettes smoked per day | CKD | IVW | 9.93 | 22 | 9.87E-01 |
| Pack years of smoking | CKD | MR Egger | 3.39 | 6 | 7.59E-01 |
| Pack years of smoking | CKD | IVW | 3.52 | 7 | 8.33E-01 |
| Maternal smoking around birth | CKD | MR Egger | 1.70 | 3 | 6.38E-01 |
| Maternal smoking around birth | CKD | IVW | 1.70 | 4 | 7.91E-01 |
| Smoking initiation | CKD | MR Egger | 63.13 | 78 | 8.89E-01 |
| Smoking initiation | CKD | IVW | 63.22 | 79 | 9.03E-01 |
| Age of smoking initiation | CKD | MR Egger | 5.72 | 3 | 1.26E-01 |
| Age of smoking initiation | CKD | IVW | 7.11 | 4 | 1.30E-01 |
| Alcoholic drinking | CKD | MR Egger | 20.23 | 30 | 9.10E-01 |
| Alcoholic drinking | CKD | IVW | 20.43 | 31 | 9.26E-01 |
| Coffee intake | CKD | MR Egger | 26.56 | 34 | 8.15E-01 |
| Coffee intake | CKD | IVW | 28.13 | 35 | 7.88E-01 |
| SBP | CKD | MR Egger | 513.15 | 612 | 9.99E-01 |
| SBP | CKD | IVW | 515.72 | 613 | 9.98E-01 |
| DBP | CKD | MR Egger | 511.93 | 608 | 9.98E-01 |
| DBP | CKD | IVW | 511.93 | 609 | 9.98E-01 |
| TG | CKD | MR Egger | 49.37 | 58 | 7.83E-01 |
| TG | CKD | IVW | 49.55 | 59 | 8.05E-01 |
| TC | CKD | MR Egger | 81.10 | 96 | 8.62E-01 |
| TC | CKD | IVW | 81.19 | 97 | 8.76E-01 |
| HDL-C | CKD | MR Egger | 82.10 | 97 | 8.60E-01 |
| HDL-C | CKD | IVW | 82.44 | 98 | 8.70E-01 |
| LDL-C | CKD | MR Egger | 72.96 | 83 | 7.77E-01 |
| LDL-C | CKD | IVW | 73.33 | 84 | 7.91E-01 |
| Fasting glucose | CKD | MR Egger | 16.55 | 25 | 8.98E-01 |
| Fasting glucose | CKD | IVW | 16.63 | 26 | 9.20E-01 |
| Fasting insulin | CKD | MR Egger | 11.15 | 11 | 4.31E-01 |
| Fasting insulin | CKD | IVW | 11.20 | 12 | 5.12E-01 |
| MVPA | CKD | MR Egger | 10.01 | 15 | 8.19E-01 |
| MVPA | CKD | IVW | 11.40 | 16 | 7.84E-01 |
| VPA | CKD | MR Egger | 3.27 | 4 | 5.14E-01 |
| VPA | CKD | IVW | 3.71 | 5 | 5.91E-01 |
| Sedentary behavior | CKD | MR Egger | 4.52 | 5 | 4.77E-01 |
| Sedentary behavior | CKD | IVW | 4.85 | 6 | 5.64E-01 |
| Household income | CKD | MR Egger | 36.27 | 41 | 6.81E-01 |
| Household income | CKD | IVW | 36.29 | 42 | 7.19E-01 |
| T1D | CKD | MR Egger | 31.89 | 36 | 6.64E-01 |
| T1D | CKD | IVW | 33.12 | 37 | 6.52E-01 |
| T2D | CKD | MR Egger | 99.95 | 109 | 7.21E-01 |
| T2D | CKD | IVW | 102.40 | 110 | 6.84E-01 |

Abbreviations: BMI, body mass index; BF%, body fat percentage; WHR, waist-to-hip ratio; HC, hip circumference; WC, waist circumference; SBP, systolic blood pressure; DBP, diastolic blood pressure; TG, triglyceride; TC, total cholesterol; HDL-C, high-density lipoprotein cholesterol; LDL-C, low-density lipoprotein cholesterol; MVPA, moderate to vigorous physical activity; VPA, vigorous physical activity; T1D, type 1 diabetes; T2D, type 2 diabetes; CKD, chronic kidney disease; IVW, Inverse variance weighted; df, degree of freedom; MR, Mendelian randomization.

Table S20. MR directional pleiotropy test (MR Egger) of the association candidate mediators with CKD

| **Candidate mediators** | **Outcome** | **Egger intercept** | **SE** | **P-value** |
| --- | --- | --- | --- | --- |
| BMI | CKD | 0.0014 | 0.0018 | 4.20E-01 |
| BF% | CKD | 0.0586 | 0.0843 | 5.25E-01 |
| WHR | CKD | 0.0012 | 0.0185 | 9.50E-01 |
| HC | CKD | -0.0123 | 0.0084 | 1.47E-01 |
| WC | CKD | -0.0114 | 0.0094 | 2.31E-01 |
| Cigarettes smoked per day | CKD | 0.0027 | 0.0066 | 6.89E-01 |
| Pack years of smoking | CKD | 0.0051 | 0.0141 | 7.28E-01 |
| Maternal smoking around birth | CKD | -0.0020 | 0.0418 | 9.65E-01 |
| Smoking initiation | CKD | -0.0028 | 0.0095 | 7.70E-01 |
| Age of smoking initiation | CKD | -0.0276 | 0.0324 | 4.57E-01 |
| Alcoholic drinking | CKD | -0.0033 | 0.0073 | 6.61E-01 |
| Coffee intake | CKD | 0.0074 | 0.0059 | 2.19E-01 |
| SBP | CKD | -0.0031 | 0.0019 | 1.09E-01 |
| DBP | CKD | 0.0000 | 0.0020 | 9.90E-01 |
| TG | CKD | -0.0016 | 0.0037 | 6.74E-01 |
| TC | CKD | 0.0010 | 0.0036 | 7.73E-01 |
| HDL-C | CKD | -0.0021 | 0.0037 | 5.59E-01 |
| LDL-C | CKD | -0.0020 | 0.0033 | 5.45E-01 |
| Fasting glucose | CKD | -0.0023 | 0.0078 | 7.72E-01 |
| Fasting insulin | CKD | -0.0052 | 0.0238 | 8.31E-01 |
| MVPA | CKD | 0.0325 | 0.0276 | 2.57E-01 |
| VPA | CKD | -0.0365 | 0.0547 | 5.41E-01 |
| Sedentary behavior | CKD | 0.0121 | 0.0212 | 5.93E-01 |
| Household income | CKD | -0.0019 | 0.0138 | 8.91E-01 |
| T1D | CKD | -0.0054 | 0.0049 | 2.76E-01 |
| T2D | CKD | -0.0061 | 0.0039 | 1.20E-01 |

Abbreviations: BMI, body mass index; BF%, body fat percentage; WHR, waist-to-hip ratio; HC, hip circumference; WC, waist circumference; SBP, systolic blood pressure; DBP, diastolic blood pressure; TG, triglyceride; TC, total cholesterol; HDL-C, high-density lipoprotein cholesterol; LDL-C, low-density lipoprotein cholesterol; MVPA, moderate to vigorous physical activity; VPA, vigorous physical activity; T1D, type 1 diabetes; T2D, type 2 diabetes; CKD, chronic kidney disease; SE, standard error; MR, Mendelian randomization.

Table S21. MVMR assessing the causal association between each mediator and CKD with adjustment for education

| **Mediators** | **method** | **Variable** | **β** | **SE** | **P** | **MVMR Instrument validity test** | | | **MVMR Heterogeneity test** | | **MVMR directional pleiotropy**  **test** | | | **NO. of SNPs** |
| --- | --- | --- | --- | --- | --- | --- | --- | --- | --- | --- | --- | --- | --- | --- |
|  |  |  |  |  |  | **F_stastistics** | **Q statistic** | **P value** | **Q statistic** | **P value** | **Egger Intercept** | **SE** | **P value** |  |
| **BMI** | MV-IVW | Education | -0.3710 | 0.0845 | 1.14E-05 | 34.1 | 1232.32 | 6.05E-07 | 1236.24 | 4.58E-07 | -0.0006 | 0.0010 | 5.16E-01 | 1003 |
|  |  | BMI | 0.3486 | 0.0477 | 2.67E-13 |  |  |  |  |  |  |  |  |  |
|  | MVMR-Egger | Education | -0.3158 | 0.1248 | 1.14E-02 |  |  |  | 1225.93 | 1.12E-06 |  |  |  |  |
|  |  | BMI | 0.3343 | 0.0485 | 5.24E-12 |  |  |  |  |  |  |  |  |  |
| **WHR** | MV-IVW | WHR | -0.3451 | 0.0809 | 1.99E-05 | 26.77 | 387.96 | 4.73E-02 | 394.71 | 3.07E-02 | 0.0024 | 0.0035 | 4.85E-01 | 346 |
|  |  | Education | 0.3658 | 0.0938 | 9.60E-05 |  |  |  |  |  |  |  |  |  |
|  | MVMR-Egger | WHR | -0.5315 | 0.2790 | 5.68E-02 |  |  |  | 394.15 | 2.95E-02 |  |  |  |  |
|  |  | Education | 0.3603 | 0.0942 | 1.31E-04 |  |  |  |  |  |  |  |  |  |
| **Cigarettes smoked per day** | MV-IVW | Cigarettes smoked per day | -0.3239 | 0.0749 | 1.53E-05 | 31.78 | 490.94 | 9.52E-03 | 494.86 | 7.49E-03 | 0.0054 | 0.0033 | 9.56E-02 | 423 |
|  |  | Education | 0.2274 | 0.0542 | 2.72E-05 |  |  |  |  |  |  |  |  |  |
|  | MVMR-Egger | Cigarettes smoked per day | -0.7209 | 0.2497 | 3.88E-03 |  |  |  | 491.6 | 9.01E-03 |  |  |  |  |
|  |  | Education | 0.2400 | 0.0546 | 1.11E-05 |  |  |  |  |  |  |  |  |  |
| **SBP** | MV-IVW | Education | -0.3566 | 0.0767 | 3.31E-06 | 41.64 | 1134.82 | 3.06E-06 | 1138.49 | 2.39E-06 | 0.0018 | 0.0010 | 7.50E-02 | 930 |
|  |  | SBP | 0.0205 | 0.0028 | 1.90E-13 |  |  |  |  |  |  |  |  |  |
|  | MVMR-Egger | Education | -0.5156 | 0.1177 | 1.18E-05 |  |  |  | 1134.51 | 3.16E-06 |  |  |  |  |
|  |  | SBP | 0.0206 | 0.0028 | 1.31E-13 |  |  |  |  |  |  |  |  |  |
| **DBP** | MV-IVW | Education | -0.4009 | 0.0810 | 7.50E-07 | 43.42 | 1258.75 | 2.33E-11 | 1262.16 | 1.75E-11 | 0.0002 | 0.0010 | 8.66E-01 | 947 |
|  |  | DBP | 0.0219 | 0.0049 | 8.58E-06 |  |  |  |  |  |  |  |  |  |
|  | MVMR-Egger | Education | -0.4170 | 0.1229 | 6.93E-04 |  |  |  | 1259.48 | 2.13E-11 |  |  |  |  |
|  |  | DBP | 0.0215 | 0.0049 | 1.21E-05 |  |  |  |  |  |  |  |  |  |
| **T2D** | MV-IVW | T2D | 0.1345 | 0.0242 | 2.54E-08 | 20.45 | 515.44 | 6.23E-03 | 518.39 | 5.31E-03 | -0.0007 | 0.0013 | 6.02E-01 | 441 |
|  |  | Education | -0.3056 | 0.0800 | 1.33E-04 |  |  |  |  |  |  |  |  |  |
|  | MVMR-Egger | T2D | 0.1450 | 0.0315 | 4.06E-06 |  |  |  | 518.06 | 4.97E-03 |  |  |  |  |
|  |  | Education | -0.3223 | 0.0862 | 1.85E-04 |  |  |  |  |  |  |  |  |  |

Abbreviations: BMI, body mass index; WHR, waist-to-hip ratio; SBP, systolic blood pressure; DBP, diastolic blood pressure; T2D, type 2 diabetes; CKD, chronic kidney disease; SE, standard error; SNP, single nucleotide polymorphism; MV-IVW, multivariable inverse variance weighted; MVMR, multivariable Mendelian randomization.

Table S22. Identification of shared susceptibility genes using TWAS

| **Exposure-outcome** | **Shared susceptibility gene** |
| --- | --- |
| Education-DKD | UQCR10 |
| BMI-DKD | CYP3A5 |
| T2D-DKD | TP53INP1 |
| SBP-DKD | TP53INP1 |
| Fasting glucose-DKD | INTS8, TP53INP1 |
| DBP-DKD | CYP3A5, INTS8, TP53INP1, UQCR10 |
| Education-CKD | ALMS1, ATP23, CEP170, DHRS11, GGNBP2, HINFP, LINC01347, MAN2B1, MYO19, PGAP3, PNMT, RP11-620J15.3, SDCCAG8, SLC7A9, ZBTB38, ZNHIT3 |
| BMI-CKD | ATG7, CEP170, CHP1, DHRS11, DHX36, FAHD2CP, GGNBP2, HINFP, L3MBTL3, MYO19, SDCCAG8, SHF, ZBTB38, ZNHIT3 |
| Cigarettes smoked per day-CKD | GGNBP2, L3MBTL3 |
| WHR-CKD | ZNHIT3 |
| SBP-CKD | ALMS1, ATG7, CEP170, CHP1, DHRS11, DHX36, GGNBP2, HSPA4, KAT5, LINC01347, MAP3K11, MYO19, SDCCAG8, ZBTB38, ZNHIT3 |
| T2D-CKD | DHX36, L3MBTL3, PGAP3, PNMT, ZBTB38 |
| DBP-CKD | CYP3A5, INTS8, TP53INP1, UQCR10 |

Abbreviations: BMI, body mass index; WHR, waist-to-hip ratio; SBP, systolic blood pressure; DBP, diastolic blood pressure; T2D, type 2 diabetes; DKD, diabetic kidney disease; CKD, chronic kidney disease.

Table S23. TWAS identified shared susceptibility genes associated with education, cardiometabolic traits, and kidney diseases

| **TWAS.Z** | **TWAS.P** | **tissue** | **TWAS.P.FDR** | **gene_name** | **Phenotype** |
| --- | --- | --- | --- | --- | --- |
| 6.8658 | 6.61E-12 | GTExv8.EUR.Whole_Blood | 3.87E-10 | RP1-140A9.1 | BMI |
| -3.8208 | 1.33E-04 | GTExv8.EUR.Whole_Blood | 1.25E-03 | RP5-892K4.1 | BMI |
| -4.3290 | 1.50E-05 | GTExv8.EUR.Whole_Blood | 1.90E-04 | RP3-395M20.8 | BMI |
| 6.0555 | 1.40E-09 | GTExv8.EUR.Whole_Blood | 4.93E-08 | RP3-395M20.9 | BMI |
| -5.5415 | 3.00E-08 | GTExv8.EUR.Whole_Blood | 7.68E-07 | FAM213B | BMI |
| -6.7480 | 1.50E-11 | GTExv8.EUR.Whole_Blood | 8.40E-10 | MMEL1 | BMI |
| 3.4359 | 5.91E-04 | GTExv8.EUR.Whole_Blood | 4.44E-03 | TTC34 | BMI |
| -2.8159 | 4.86E-03 | GTExv8.EUR.Whole_Blood | 2.44E-02 | RNF207 | BMI |
| -2.9491 | 3.19E-03 | GTExv8.EUR.Whole_Blood | 1.74E-02 | PLEKHG5 | BMI |
| 4.4762 | 7.60E-06 | GTExv8.EUR.Whole_Blood | 1.05E-04 | NOL9 | BMI |
| -6.4898 | 8.60E-11 | GTExv8.EUR.Whole_Blood | 4.06E-09 | PHF13 | BMI |
| 4.4172 | 1.00E-05 | GTExv8.EUR.Whole_Blood | 1.33E-04 | RP5-1115A15.1 | BMI |
| 4.4980 | 6.86E-06 | GTExv8.EUR.Whole_Blood | 9.57E-05 | RERE | BMI |
| 3.0602 | 2.21E-03 | GTExv8.EUR.Whole_Blood | 1.29E-02 | CTNNBIP1 | BMI |
| -7.1047 | 1.21E-12 | GTExv8.EUR.Whole_Blood | 8.48E-11 | RP4-635E18.7 | BMI |
| 7.0356 | 1.98E-12 | GTExv8.EUR.Whole_Blood | 1.33E-10 | MTOR | BMI |
| 3.3037 | 9.54E-04 | GTExv8.EUR.Whole_Blood | 6.65E-03 | FBXO44 | BMI |
| 3.7554 | 1.73E-04 | GTExv8.EUR.Whole_Blood | 1.56E-03 | DRAXIN | BMI |
| 2.9576 | 3.10E-03 | GTExv8.EUR.Whole_Blood | 1.70E-02 | NPPA | BMI |
| -3.6329 | 2.80E-04 | GTExv8.EUR.Whole_Blood | 2.36E-03 | CASP9 | BMI |
| -4.0108 | 6.05E-05 | GTExv8.EUR.Whole_Blood | 6.28E-04 | DDI2 | BMI |
| 3.6008 | 3.17E-04 | GTExv8.EUR.Whole_Blood | 2.61E-03 | SPEN | BMI |
| -2.7478 | 6.00E-03 | GTExv8.EUR.Whole_Blood | 2.88E-02 | MT1XP1 | BMI |
| 3.7283 | 1.93E-04 | GTExv8.EUR.Whole_Blood | 1.71E-03 | FBXO42 | BMI |
| -3.2573 | 1.12E-03 | GTExv8.EUR.Whole_Blood | 7.57E-03 | NBPF1 | BMI |
| -5.5567 | 2.75E-08 | GTExv8.EUR.Whole_Blood | 7.13E-07 | MST1P2 | BMI |
| -4.0031 | 6.25E-05 | GTExv8.EUR.Whole_Blood | 6.47E-04 | CROCCP2 | BMI |
| 6.4170 | 1.39E-10 | GTExv8.EUR.Whole_Blood | 6.15E-09 | MST1L | BMI |
| 4.3323 | 1.48E-05 | GTExv8.EUR.Whole_Blood | 1.89E-04 | RP11-108M9.6 | BMI |
| 3.3354 | 8.52E-04 | GTExv8.EUR.Whole_Blood | 6.07E-03 | CROCC | BMI |
| -4.0966 | 4.19E-05 | GTExv8.EUR.Whole_Blood | 4.58E-04 | ATP13A2 | BMI |
| -4.2706 | 1.95E-05 | GTExv8.EUR.Whole_Blood | 2.37E-04 | PADI2 | BMI |
| 3.0830 | 2.05E-03 | GTExv8.EUR.Whole_Blood | 1.23E-02 | NBL1 | BMI |
| 2.6008 | 9.30E-03 | GTExv8.EUR.Whole_Blood | 4.05E-02 | NBPF3 | BMI |
| -3.0797 | 2.07E-03 | GTExv8.EUR.Whole_Blood | 1.24E-02 | LUZP1 | BMI |
| -2.7485 | 5.99E-03 | GTExv8.EUR.Whole_Blood | 2.88E-02 | STPG1 | BMI |
| 3.7223 | 1.97E-04 | GTExv8.EUR.Whole_Blood | 1.74E-03 | WASF2 | BMI |
| -2.6693 | 7.60E-03 | GTExv8.EUR.Whole_Blood | 3.45E-02 | DNAJC8 | BMI |
| -4.8398 | 1.30E-06 | GTExv8.EUR.Whole_Blood | 2.14E-05 | PEF1 | BMI |
| -3.0357 | 2.40E-03 | GTExv8.EUR.Whole_Blood | 1.38E-02 | TMEM39B | BMI |
| -2.6922 | 7.10E-03 | GTExv8.EUR.Whole_Blood | 3.28E-02 | RP11-131M11.3 | BMI |
| -3.8674 | 1.10E-04 | GTExv8.EUR.Whole_Blood | 1.06E-03 | ZNF362 | BMI |
| -3.1617 | 1.57E-03 | GTExv8.EUR.Whole_Blood | 9.93E-03 | MAP7D1 | BMI |
| -5.1625 | 2.44E-07 | GTExv8.EUR.Whole_Blood | 4.89E-06 | MEAF6 | BMI |
| 3.3338 | 8.57E-04 | GTExv8.EUR.Whole_Blood | 6.09E-03 | NDUFS5 | BMI |
| 5.7354 | 9.73E-09 | GTExv8.EUR.Whole_Blood | 2.83E-07 | BMP8A | BMI |
| 5.0580 | 4.24E-07 | GTExv8.EUR.Whole_Blood | 7.87E-06 | RP11-69E11.4 | BMI |
| 5.0325 | 4.84E-07 | GTExv8.EUR.Whole_Blood | 8.82E-06 | PPIEL | BMI |
| -6.5946 | 4.26E-11 | GTExv8.EUR.Whole_Blood | 2.20E-09 | PABPC4 | BMI |
| 3.2012 | 1.37E-03 | GTExv8.EUR.Whole_Blood | 8.92E-03 | TRIT1 | BMI |
| -3.0357 | 2.40E-03 | GTExv8.EUR.Whole_Blood | 1.38E-02 | C1orf50 | BMI |
| 2.7798 | 5.44E-03 | GTExv8.EUR.Whole_Blood | 2.66E-02 | SVBP | BMI |
| 2.9285 | 3.41E-03 | GTExv8.EUR.Whole_Blood | 1.83E-02 | SZT2 | BMI |
| 2.9298 | 3.39E-03 | GTExv8.EUR.Whole_Blood | 1.82E-02 | CCDC24 | BMI |
| 3.0648 | 2.18E-03 | GTExv8.EUR.Whole_Blood | 1.28E-02 | MUTYH | BMI |
| -3.5732 | 3.53E-04 | GTExv8.EUR.Whole_Blood | 2.86E-03 | TESK2 | BMI |
| -4.1754 | 2.97E-05 | GTExv8.EUR.Whole_Blood | 3.43E-04 | AKR1A1 | BMI |
| 5.2845 | 1.26E-07 | GTExv8.EUR.Whole_Blood | 2.75E-06 | CCDC17 | BMI |
| -6.5102 | 7.51E-11 | GTExv8.EUR.Whole_Blood | 3.65E-09 | RP11-767N6.2 | BMI |
| 7.1897 | 6.49E-13 | GTExv8.EUR.Whole_Blood | 4.86E-11 | MAST2 | BMI |
| 5.9031 | 3.57E-09 | GTExv8.EUR.Whole_Blood | 1.17E-07 | PIK3R3 | BMI |
| -2.5962 | 9.43E-03 | GTExv8.EUR.Whole_Blood | 4.09E-02 | POMGNT1 | BMI |
| 3.1998 | 1.38E-03 | GTExv8.EUR.Whole_Blood | 8.96E-03 | FAAH | BMI |
| -5.4513 | 5.00E-08 | GTExv8.EUR.Whole_Blood | 1.22E-06 | PDZK1IP1 | BMI |
| 3.1788 | 1.48E-03 | GTExv8.EUR.Whole_Blood | 9.46E-03 | FOXD2-AS1 | BMI |
| -3.5701 | 3.57E-04 | GTExv8.EUR.Whole_Blood | 2.89E-03 | RP4-657D16.3 | BMI |
| 2.5471 | 1.09E-02 | GTExv8.EUR.Whole_Blood | 4.58E-02 | TXNDC12 | BMI |
| 3.5566 | 3.76E-04 | GTExv8.EUR.Whole_Blood | 3.02E-03 | DOCK7 | BMI |
| -2.5497 | 1.08E-02 | GTExv8.EUR.Whole_Blood | 4.56E-02 | CTH | BMI |
| -3.8660 | 1.11E-04 | GTExv8.EUR.Whole_Blood | 1.07E-03 | PTGFR | BMI |
| -4.1657 | 3.10E-05 | GTExv8.EUR.Whole_Blood | 3.55E-04 | RP11-33E12.2 | BMI |
| 4.2784 | 1.88E-05 | GTExv8.EUR.Whole_Blood | 2.30E-04 | BCL10 | BMI |
| 2.7991 | 5.12E-03 | GTExv8.EUR.Whole_Blood | 2.54E-02 | ODF2L | BMI |
| -2.5418 | 1.10E-02 | GTExv8.EUR.Whole_Blood | 4.60E-02 | KYAT3 | BMI |
| -2.7791 | 5.45E-03 | GTExv8.EUR.Whole_Blood | 2.66E-02 | LRRC8B | BMI |
| 5.2821 | 1.28E-07 | GTExv8.EUR.Whole_Blood | 2.79E-06 | EVI5 | BMI |
| -2.6120 | 9.00E-03 | GTExv8.EUR.Whole_Blood | 3.94E-02 | DNTTIP2 | BMI |
| -3.0065 | 2.64E-03 | GTExv8.EUR.Whole_Blood | 1.49E-02 | DPYD | BMI |
| 2.5828 | 9.80E-03 | GTExv8.EUR.Whole_Blood | 4.23E-02 | RTCA | BMI |
| -2.7971 | 5.16E-03 | GTExv8.EUR.Whole_Blood | 2.56E-02 | CDC14A | BMI |
| -6.4525 | 1.10E-10 | GTExv8.EUR.Whole_Blood | 5.01E-09 | PRMT6 | BMI |
| 3.9729 | 7.10E-05 | GTExv8.EUR.Whole_Blood | 7.18E-04 | TAF13 | BMI |
| 4.2301 | 2.34E-05 | GTExv8.EUR.Whole_Blood | 2.80E-04 | SYPL2 | BMI |
| -6.2736 | 3.53E-10 | GTExv8.EUR.Whole_Blood | 1.40E-08 | ATXN7L2 | BMI |
| 5.8678 | 4.42E-09 | GTExv8.EUR.Whole_Blood | 1.40E-07 | AMIGO1 | BMI |
| 3.2926 | 9.93E-04 | GTExv8.EUR.Whole_Blood | 6.83E-03 | GSTM4 | BMI |
| 4.0391 | 5.37E-05 | GTExv8.EUR.Whole_Blood | 5.67E-04 | STRIP1 | BMI |
| -3.1811 | 1.47E-03 | GTExv8.EUR.Whole_Blood | 9.43E-03 | TMIGD3 | BMI |
| 2.6521 | 8.00E-03 | GTExv8.EUR.Whole_Blood | 3.59E-02 | DDX20 | BMI |
| 2.6893 | 7.16E-03 | GTExv8.EUR.Whole_Blood | 3.30E-02 | TRIM33 | BMI |
| 4.7747 | 1.80E-06 | GTExv8.EUR.Whole_Blood | 2.85E-05 | SLC22A15 | BMI |
| -2.6693 | 7.60E-03 | GTExv8.EUR.Whole_Blood | 3.45E-02 | RP5-1086K13.1 | BMI |
| 2.5747 | 1.00E-02 | GTExv8.EUR.Whole_Blood | 4.29E-02 | WARS2 | BMI |
| 2.8266 | 4.70E-03 | GTExv8.EUR.Whole_Blood | 2.39E-02 | RP11-418J17.1 | BMI |
| -2.5780 | 9.94E-03 | GTExv8.EUR.Whole_Blood | 4.28E-02 | NUDT17 | BMI |
| 4.2294 | 2.34E-05 | GTExv8.EUR.Whole_Blood | 2.80E-04 | MRPS21 | BMI |
| 4.8238 | 1.41E-06 | GTExv8.EUR.Whole_Blood | 2.30E-05 | ECM1 | BMI |
| 5.5496 | 2.86E-08 | GTExv8.EUR.Whole_Blood | 7.39E-07 | RP11-54A4.2 | BMI |
| -3.9444 | 8.00E-05 | GTExv8.EUR.Whole_Blood | 7.98E-04 | ENSA | BMI |
| -3.1456 | 1.66E-03 | GTExv8.EUR.Whole_Blood | 1.04E-02 | GOLPH3L | BMI |
| -3.2389 | 1.20E-03 | GTExv8.EUR.Whole_Blood | 8.00E-03 | HORMAD1 | BMI |
| 3.1382 | 1.70E-03 | GTExv8.EUR.Whole_Blood | 1.05E-02 | SETDB1 | BMI |
| 5.0405 | 4.64E-07 | GTExv8.EUR.Whole_Blood | 8.54E-06 | SEMA6C | BMI |
| -5.6302 | 1.80E-08 | GTExv8.EUR.Whole_Blood | 4.90E-07 | LYSMD1 | BMI |
| 3.0757 | 2.10E-03 | GTExv8.EUR.Whole_Blood | 1.25E-02 | POGZ | BMI |
| 3.0233 | 2.50E-03 | GTExv8.EUR.Whole_Blood | 1.43E-02 | DCST1-AS1 | BMI |
| -3.8949 | 9.82E-05 | GTExv8.EUR.Whole_Blood | 9.59E-04 | MUC1 | BMI |
| -4.9707 | 6.67E-07 | GTExv8.EUR.Whole_Blood | 1.17E-05 | RP11-263K19.4 | BMI |
| -6.1132 | 9.77E-10 | GTExv8.EUR.Whole_Blood | 3.58E-08 | THBS3 | BMI |
| -3.8821 | 1.04E-04 | GTExv8.EUR.Whole_Blood | 1.01E-03 | RP11-263K19.6 | BMI |
| 4.1517 | 3.30E-05 | GTExv8.EUR.Whole_Blood | 3.72E-04 | GBAP1 | BMI |
| 2.9374 | 3.31E-03 | GTExv8.EUR.Whole_Blood | 1.79E-02 | FDPS | BMI |
| 2.8943 | 3.80E-03 | GTExv8.EUR.Whole_Blood | 2.00E-02 | SYT11 | BMI |
| -3.7607 | 1.69E-04 | GTExv8.EUR.Whole_Blood | 1.53E-03 | RP11-336K24.12 | BMI |
| -3.5536 | 3.80E-04 | GTExv8.EUR.Whole_Blood | 3.04E-03 | LMNA | BMI |
| -2.8944 | 3.80E-03 | GTExv8.EUR.Whole_Blood | 2.00E-02 | FCRL1 | BMI |
| -3.3183 | 9.06E-04 | GTExv8.EUR.Whole_Blood | 6.38E-03 | CD1B | BMI |
| -3.1947 | 1.40E-03 | GTExv8.EUR.Whole_Blood | 9.03E-03 | CD1E | BMI |
| -3.5265 | 4.21E-04 | GTExv8.EUR.Whole_Blood | 3.33E-03 | DCAF8 | BMI |
| 2.7370 | 6.20E-03 | GTExv8.EUR.Whole_Blood | 2.95E-02 | F11R | BMI |
| 2.6756 | 7.46E-03 | GTExv8.EUR.Whole_Blood | 3.40E-02 | NDUFS2 | BMI |
| -3.0646 | 2.18E-03 | GTExv8.EUR.Whole_Blood | 1.28E-02 | APOA2 | BMI |
| -4.0802 | 4.50E-05 | GTExv8.EUR.Whole_Blood | 4.87E-04 | PIGC | BMI |
| 6.3692 | 1.90E-10 | GTExv8.EUR.Whole_Blood | 7.99E-09 | SERPINC1 | BMI |
| 4.8918 | 9.99E-07 | GTExv8.EUR.Whole_Blood | 1.68E-05 | RABGAP1L | BMI |
| -4.1644 | 3.12E-05 | GTExv8.EUR.Whole_Blood | 3.57E-04 | RP11-318C24.2 | BMI |
| -3.8082 | 1.40E-04 | GTExv8.EUR.Whole_Blood | 1.31E-03 | EDEM3 | BMI |
| -3.4098 | 6.50E-04 | GTExv8.EUR.Whole_Blood | 4.81E-03 | PEBP1P3 | BMI |
| 2.8903 | 3.85E-03 | GTExv8.EUR.Whole_Blood | 2.02E-02 | KIF21B | BMI |
| -3.1164 | 1.83E-03 | GTExv8.EUR.Whole_Blood | 1.12E-02 | LAX1 | BMI |
| 2.6954 | 7.03E-03 | GTExv8.EUR.Whole_Blood | 3.26E-02 | LRRN2 | BMI |
| 3.6836 | 2.30E-04 | GTExv8.EUR.Whole_Blood | 1.99E-03 | ELK4 | BMI |
| 4.1974 | 2.70E-05 | GTExv8.EUR.Whole_Blood | 3.14E-04 | NUCKS1 | BMI |
| -5.1396 | 2.75E-07 | GTExv8.EUR.Whole_Blood | 5.39E-06 | RAB29 | BMI |
| -6.3538 | 2.10E-10 | GTExv8.EUR.Whole_Blood | 8.74E-09 | SLC41A1 | BMI |
| 6.2403 | 4.37E-10 | GTExv8.EUR.Whole_Blood | 1.71E-08 | PM20D1 | BMI |
| 2.6729 | 7.52E-03 | GTExv8.EUR.Whole_Blood | 3.42E-02 | PLXNA2 | BMI |
| 3.6200 | 2.95E-04 | GTExv8.EUR.Whole_Blood | 2.46E-03 | IRF6 | BMI |
| -3.2891 | 1.01E-03 | GTExv8.EUR.Whole_Blood | 6.92E-03 | DIEXF | BMI |
| -3.4403 | 5.81E-04 | GTExv8.EUR.Whole_Blood | 4.38E-03 | INTS7 | BMI |
| -2.5701 | 1.02E-02 | GTExv8.EUR.Whole_Blood | 4.35E-02 | NENF | BMI |
| 2.6476 | 8.11E-03 | GTExv8.EUR.Whole_Blood | 3.63E-02 | ANGEL2 | BMI |
| -5.3067 | 1.12E-07 | GTExv8.EUR.Whole_Blood | 2.50E-06 | RPS6KC1 | BMI |
| 2.6400 | 8.29E-03 | GTExv8.EUR.Whole_Blood | 3.69E-02 | RP11-295M18.6 | BMI |
| -2.7822 | 5.40E-03 | GTExv8.EUR.Whole_Blood | 2.64E-02 | FBXO28 | BMI |
| -2.7403 | 6.14E-03 | GTExv8.EUR.Whole_Blood | 2.93E-02 | NVL | BMI |
| -3.9451 | 7.98E-05 | GTExv8.EUR.Whole_Blood | 7.97E-04 | SNAP47 | BMI |
| -3.1747 | 1.50E-03 | GTExv8.EUR.Whole_Blood | 9.54E-03 | RNF187 | BMI |
| -2.8040 | 5.05E-03 | GTExv8.EUR.Whole_Blood | 2.51E-02 | RAB4A | BMI |
| 2.8627 | 4.20E-03 | GTExv8.EUR.Whole_Blood | 2.18E-02 | RP11-443B7.1 | BMI |
| -3.5139 | 4.41E-04 | GTExv8.EUR.Whole_Blood | 3.45E-03 | TBCE | BMI |
| 2.7370 | 6.20E-03 | GTExv8.EUR.Whole_Blood | 2.95E-02 | CEP170 | BMI |
| 2.7019 | 6.89E-03 | GTExv8.EUR.Whole_Blood | 3.21E-02 | SDCCAG8 | BMI |
| 3.0036 | 2.67E-03 | GTExv8.EUR.Whole_Blood | 1.51E-02 | EFCAB2 | BMI |
| 5.0303 | 4.90E-07 | GTExv8.EUR.Whole_Blood | 8.90E-06 | SH3YL1 | BMI |
| 5.6935 | 1.24E-08 | GTExv8.EUR.Whole_Blood | 3.51E-07 | AC116614.1 | BMI |
| -2.9430 | 3.25E-03 | GTExv8.EUR.Whole_Blood | 1.76E-02 | PXDN | BMI |
| -3.9071 | 9.34E-05 | GTExv8.EUR.Whole_Blood | 9.15E-04 | ATP6V1C2 | BMI |
| -4.2191 | 2.45E-05 | GTExv8.EUR.Whole_Blood | 2.90E-04 | RP11-791G15.2 | BMI |
| -4.6638 | 3.10E-06 | GTExv8.EUR.Whole_Blood | 4.71E-05 | PDIA6 | BMI |
| 3.8674 | 1.10E-04 | GTExv8.EUR.Whole_Blood | 1.06E-03 | PQLC3 | BMI |
| 2.7214 | 6.50E-03 | GTExv8.EUR.Whole_Blood | 3.08E-02 | ROCK2 | BMI |
| 2.5691 | 1.02E-02 | GTExv8.EUR.Whole_Blood | 4.35E-02 | NBAS | BMI |
| 3.4633 | 5.34E-04 | GTExv8.EUR.Whole_Blood | 4.07E-03 | FKBP1B | BMI |
| -3.5218 | 4.29E-04 | GTExv8.EUR.Whole_Blood | 3.38E-03 | TP53I3 | BMI |
| 15.5681 | 1.20E-54 | GTExv8.EUR.Whole_Blood | 1.04E-51 | PTRHD1 | BMI |
| 18.3554 | 2.99E-75 | GTExv8.EUR.Whole_Blood | 1.16E-71 | CENPO | BMI |
| 17.3823 | 1.12E-67 | GTExv8.EUR.Whole_Blood | 2.18E-64 | RP11-443B20.1 | BMI |
| 18.3139 | 6.41E-75 | GTExv8.EUR.Whole_Blood | 1.66E-71 | ADCY3 | BMI |
| 18.8208 | 5.10E-79 | GTExv8.EUR.Whole_Blood | 3.97E-75 | DNAJC27 | BMI |
| 5.0669 | 4.04E-07 | GTExv8.EUR.Whole_Blood | 7.56E-06 | SELENOI | BMI |
| -5.3477 | 8.91E-08 | GTExv8.EUR.Whole_Blood | 2.03E-06 | CGREF1 | BMI |
| -2.6121 | 9.00E-03 | GTExv8.EUR.Whole_Blood | 3.94E-02 | NRBP1 | BMI |
| 3.8177 | 1.35E-04 | GTExv8.EUR.Whole_Blood | 1.27E-03 | PLB1 | BMI |
| -6.1417 | 8.16E-10 | GTExv8.EUR.Whole_Blood | 3.04E-08 | PPP1CB | BMI |
| 6.3178 | 2.65E-10 | GTExv8.EUR.Whole_Blood | 1.07E-08 | TRMT61B | BMI |
| 3.1435 | 1.67E-03 | GTExv8.EUR.Whole_Blood | 1.04E-02 | TOGARAM2 | BMI |
| -3.9414 | 8.10E-05 | GTExv8.EUR.Whole_Blood | 8.07E-04 | CTC-336P14.1 | BMI |
| -3.3130 | 9.23E-04 | GTExv8.EUR.Whole_Blood | 6.48E-03 | CRIM1 | BMI |
| 5.6454 | 1.65E-08 | GTExv8.EUR.Whole_Blood | 4.54E-07 | FEZ2 | BMI |
| 6.3209 | 2.60E-10 | GTExv8.EUR.Whole_Blood | 1.06E-08 | VIT | BMI |
| -2.9911 | 2.78E-03 | GTExv8.EUR.Whole_Blood | 1.56E-02 | CEBPZOS | BMI |
| -3.1191 | 1.81E-03 | GTExv8.EUR.Whole_Blood | 1.11E-02 | PRKD3 | BMI |
| 3.3320 | 8.62E-04 | GTExv8.EUR.Whole_Blood | 6.11E-03 | CDC42EP3 | BMI |
| 2.7055 | 6.82E-03 | GTExv8.EUR.Whole_Blood | 3.19E-02 | AC010878.3 | BMI |
| 2.5436 | 1.10E-02 | GTExv8.EUR.Whole_Blood | 4.60E-02 | LINC00211 | BMI |
| -2.5570 | 1.06E-02 | GTExv8.EUR.Whole_Blood | 4.50E-02 | RP11-314C9.2 | BMI |
| 3.4716 | 5.17E-04 | GTExv8.EUR.Whole_Blood | 3.96E-03 | COX7A2L | BMI |
| 4.9060 | 9.29E-07 | GTExv8.EUR.Whole_Blood | 1.58E-05 | OXER1 | BMI |
| 5.4109 | 6.27E-08 | GTExv8.EUR.Whole_Blood | 1.48E-06 | HAAO | BMI |
| 5.8800 | 4.10E-09 | GTExv8.EUR.Whole_Blood | 1.31E-07 | PREPL | BMI |
| -6.1778 | 6.50E-10 | GTExv8.EUR.Whole_Blood | 2.49E-08 | CAMKMT | BMI |
| 4.3182 | 1.57E-05 | GTExv8.EUR.Whole_Blood | 1.98E-04 | ATP6V1E2 | BMI |
| -5.7584 | 8.49E-09 | GTExv8.EUR.Whole_Blood | 2.52E-07 | CRIPT | BMI |
| 3.6838 | 2.30E-04 | GTExv8.EUR.Whole_Blood | 1.99E-03 | RP11-536C12.1 | BMI |
| -3.1747 | 1.50E-03 | GTExv8.EUR.Whole_Blood | 9.54E-03 | C2orf61 | BMI |
| 3.3706 | 7.50E-04 | GTExv8.EUR.Whole_Blood | 5.41E-03 | GPR75 | BMI |
| 3.1798 | 1.47E-03 | GTExv8.EUR.Whole_Blood | 9.43E-03 | ACYP2 | BMI |
| -3.7882 | 1.52E-04 | GTExv8.EUR.Whole_Blood | 1.40E-03 | HMGB1P31 | BMI |
| -2.7680 | 5.64E-03 | GTExv8.EUR.Whole_Blood | 2.74E-02 | RP11-477N3.1 | BMI |
| -3.1243 | 1.78E-03 | GTExv8.EUR.Whole_Blood | 1.09E-02 | RTN4 | BMI |
| 2.5122 | 1.20E-02 | GTExv8.EUR.Whole_Blood | 4.94E-02 | NONOP2 | BMI |
| 3.0861 | 2.03E-03 | GTExv8.EUR.Whole_Blood | 1.22E-02 | OTX1 | BMI |
| 5.0682 | 4.02E-07 | GTExv8.EUR.Whole_Blood | 7.54E-06 | MDH1 | BMI |
| 4.2750 | 1.91E-05 | GTExv8.EUR.Whole_Blood | 2.33E-04 | WDPCP | BMI |
| -3.2919 | 9.95E-04 | GTExv8.EUR.Whole_Blood | 6.84E-03 | AC114752.3 | BMI |
| 3.0357 | 2.40E-03 | GTExv8.EUR.Whole_Blood | 1.38E-02 | GFPT1 | BMI |
| -3.7750 | 1.60E-04 | GTExv8.EUR.Whole_Blood | 1.46E-03 | RAB11FIP5 | BMI |
| 3.1382 | 1.70E-03 | GTExv8.EUR.Whole_Blood | 1.05E-02 | STAMBP | BMI |
| 2.6526 | 7.99E-03 | GTExv8.EUR.Whole_Blood | 3.59E-02 | RETSAT | BMI |
| 2.6972 | 6.99E-03 | GTExv8.EUR.Whole_Blood | 3.25E-02 | ELMOD3 | BMI |
| 2.6871 | 7.21E-03 | GTExv8.EUR.Whole_Blood | 3.32E-02 | GGCX | BMI |
| -8.5387 | 1.36E-17 | GTExv8.EUR.Whole_Blood | 1.45E-15 | CHMP3 | BMI |
| 3.1913 | 1.42E-03 | GTExv8.EUR.Whole_Blood | 9.14E-03 | CD8A | BMI |
| -2.7434 | 6.08E-03 | GTExv8.EUR.Whole_Blood | 2.91E-02 | MAL | BMI |
| -4.1018 | 4.10E-05 | GTExv8.EUR.Whole_Blood | 4.51E-04 | LINC00342 | BMI |
| -3.2991 | 9.70E-04 | GTExv8.EUR.Whole_Blood | 6.73E-03 | FAHD2CP | BMI |
| -3.0618 | 2.20E-03 | GTExv8.EUR.Whole_Blood | 1.29E-02 | TMEM127 | BMI |
| -3.1766 | 1.49E-03 | GTExv8.EUR.Whole_Blood | 9.51E-03 | FAHD2B | BMI |
| -3.0786 | 2.08E-03 | GTExv8.EUR.Whole_Blood | 1.24E-02 | COX5B | BMI |
| 5.2294 | 1.70E-07 | GTExv8.EUR.Whole_Blood | 3.58E-06 | ACTR1B | BMI |
| 3.3398 | 8.38E-04 | GTExv8.EUR.Whole_Blood | 5.98E-03 | RP11-527J8.1 | BMI |
| -4.2969 | 1.73E-05 | GTExv8.EUR.Whole_Blood | 2.13E-04 | CHST10 | BMI |
| 5.1767 | 2.26E-07 | GTExv8.EUR.Whole_Blood | 4.58E-06 | MRPS9 | BMI |
| -3.4225 | 6.20E-04 | GTExv8.EUR.Whole_Blood | 4.61E-03 | ANAPC1 | BMI |
| 2.5186 | 1.18E-02 | GTExv8.EUR.Whole_Blood | 4.89E-02 | SLC20A1 | BMI |
| 3.9663 | 7.30E-05 | GTExv8.EUR.Whole_Blood | 7.34E-04 | TMEM163 | BMI |
| 4.9311 | 8.18E-07 | GTExv8.EUR.Whole_Blood | 1.41E-05 | CCNT2-AS1 | BMI |
| -4.6241 | 3.76E-06 | GTExv8.EUR.Whole_Blood | 5.56E-05 | CCNT2 | BMI |
| -5.0694 | 3.99E-07 | GTExv8.EUR.Whole_Blood | 7.50E-06 | MCM6 | BMI |
| 3.5535 | 3.80E-04 | GTExv8.EUR.Whole_Blood | 3.04E-03 | DARS-AS1 | BMI |
| -3.9724 | 7.11E-05 | GTExv8.EUR.Whole_Blood | 7.18E-04 | DARS | BMI |
| 3.1149 | 1.84E-03 | GTExv8.EUR.Whole_Blood | 1.12E-02 | MTND3P9 | BMI |
| 3.0923 | 1.99E-03 | GTExv8.EUR.Whole_Blood | 1.20E-02 | MTCO3P5 | BMI |
| 2.9435 | 3.25E-03 | GTExv8.EUR.Whole_Blood | 1.76E-02 | RP11-190J23.2 | BMI |
| 3.5098 | 4.48E-04 | GTExv8.EUR.Whole_Blood | 3.50E-03 | ACVR2A | BMI |
| -3.2799 | 1.04E-03 | GTExv8.EUR.Whole_Blood | 7.10E-03 | GPD2 | BMI |
| -2.6728 | 7.52E-03 | GTExv8.EUR.Whole_Blood | 3.42E-02 | WDSUB1 | BMI |
| 2.9365 | 3.32E-03 | GTExv8.EUR.Whole_Blood | 1.79E-02 | AC009961.3 | BMI |
| 3.4975 | 4.70E-04 | GTExv8.EUR.Whole_Blood | 3.64E-03 | LY75 | BMI |
| 2.5099 | 1.21E-02 | GTExv8.EUR.Whole_Blood | 4.97E-02 | GAD1 | BMI |
| 3.7772 | 1.59E-04 | GTExv8.EUR.Whole_Blood | 1.46E-03 | CYBRD1 | BMI |
| 5.4479 | 5.10E-08 | GTExv8.EUR.Whole_Blood | 1.24E-06 | HAT1 | BMI |
| 6.9965 | 2.62E-12 | GTExv8.EUR.Whole_Blood | 1.66E-10 | SLC25A12 | BMI |
| 3.0543 | 2.26E-03 | GTExv8.EUR.Whole_Blood | 1.32E-02 | MAP3K20 | BMI |
| -4.6512 | 3.30E-06 | GTExv8.EUR.Whole_Blood | 4.99E-05 | OLA1 | BMI |
| -3.1142 | 1.84E-03 | GTExv8.EUR.Whole_Blood | 1.12E-02 | GPR155 | BMI |
| -2.8358 | 4.57E-03 | GTExv8.EUR.Whole_Blood | 2.34E-02 | AC074286.1 | BMI |
| -3.7575 | 1.72E-04 | GTExv8.EUR.Whole_Blood | 1.55E-03 | UBE2E3 | BMI |
| -4.5778 | 4.70E-06 | GTExv8.EUR.Whole_Blood | 6.76E-05 | ITGAV | BMI |
| 3.9801 | 6.89E-05 | GTExv8.EUR.Whole_Blood | 6.99E-04 | CALCRL | BMI |
| -6.4845 | 8.90E-11 | GTExv8.EUR.Whole_Blood | 4.17E-09 | PLCL1 | BMI |
| 2.6411 | 8.26E-03 | GTExv8.EUR.Whole_Blood | 3.68E-02 | FTCDNL1 | BMI |
| -5.2927 | 1.21E-07 | GTExv8.EUR.Whole_Blood | 2.66E-06 | C2orf69 | BMI |
| -5.3046 | 1.13E-07 | GTExv8.EUR.Whole_Blood | 2.52E-06 | MAIP1 | BMI |
| -4.3545 | 1.33E-05 | GTExv8.EUR.Whole_Blood | 1.72E-04 | SPATS2L | BMI |
| 3.7969 | 1.47E-04 | GTExv8.EUR.Whole_Blood | 1.36E-03 | RP11-686O6.2 | BMI |
| -7.2426 | 4.40E-13 | GTExv8.EUR.Whole_Blood | 3.53E-11 | ICA1L | BMI |
| -2.5104 | 1.21E-02 | GTExv8.EUR.Whole_Blood | 4.97E-02 | KLF7-IT1 | BMI |
| 3.5393 | 4.01E-04 | GTExv8.EUR.Whole_Blood | 3.19E-03 | AC007879.2 | BMI |
| -3.5277 | 4.19E-04 | GTExv8.EUR.Whole_Blood | 3.32E-03 | LINC01857 | BMI |
| -3.1747 | 1.50E-03 | GTExv8.EUR.Whole_Blood | 9.54E-03 | RPE | BMI |
| 3.5879 | 3.33E-04 | GTExv8.EUR.Whole_Blood | 2.73E-03 | LANCL1 | BMI |
| -4.0832 | 4.44E-05 | GTExv8.EUR.Whole_Blood | 4.81E-04 | SMARCAL1 | BMI |
| -2.7033 | 6.86E-03 | GTExv8.EUR.Whole_Blood | 3.20E-02 | CATIP | BMI |
| -6.7125 | 1.91E-11 | GTExv8.EUR.Whole_Blood | 1.05E-09 | VIL1 | BMI |
| 6.4048 | 1.51E-10 | GTExv8.EUR.Whole_Blood | 6.60E-09 | USP37 | BMI |
| -6.4313 | 1.27E-10 | GTExv8.EUR.Whole_Blood | 5.68E-09 | CNOT9 | BMI |
| 6.5920 | 4.34E-11 | GTExv8.EUR.Whole_Blood | 2.22E-09 | CYP27A1 | BMI |
| 6.5291 | 6.62E-11 | GTExv8.EUR.Whole_Blood | 3.26E-09 | RP11-459I19.1 | BMI |
| -3.2546 | 1.14E-03 | GTExv8.EUR.Whole_Blood | 7.67E-03 | FAM134A | BMI |
| -3.3153 | 9.15E-04 | GTExv8.EUR.Whole_Blood | 6.44E-03 | GLB1L | BMI |
| -6.5678 | 5.11E-11 | GTExv8.EUR.Whole_Blood | 2.58E-09 | PTPRN | BMI |
| 3.7377 | 1.86E-04 | GTExv8.EUR.Whole_Blood | 1.65E-03 | AC010980.2 | BMI |
| 3.6724 | 2.40E-04 | GTExv8.EUR.Whole_Blood | 2.06E-03 | RP11-16P6.1 | BMI |
| 3.4915 | 4.80E-04 | GTExv8.EUR.Whole_Blood | 3.70E-03 | FARSB | BMI |
| -3.0383 | 2.38E-03 | GTExv8.EUR.Whole_Blood | 1.38E-02 | IRS1 | BMI |
| -5.3153 | 1.06E-07 | GTExv8.EUR.Whole_Blood | 2.38E-06 | TRIP12 | BMI |
| 3.1637 | 1.56E-03 | GTExv8.EUR.Whole_Blood | 9.87E-03 | SP140 | BMI |
| -2.8228 | 4.76E-03 | GTExv8.EUR.Whole_Blood | 2.41E-02 | AC017104.6 | BMI |
| -2.5916 | 9.55E-03 | GTExv8.EUR.Whole_Blood | 4.14E-02 | NCL | BMI |
| -4.8834 | 1.04E-06 | GTExv8.EUR.Whole_Blood | 1.75E-05 | PDE6D | BMI |
| -4.3008 | 1.70E-05 | GTExv8.EUR.Whole_Blood | 2.10E-04 | RAMP1 | BMI |
| 2.5249 | 1.16E-02 | GTExv8.EUR.Whole_Blood | 4.82E-02 | PASK | BMI |
| 3.2075 | 1.34E-03 | GTExv8.EUR.Whole_Blood | 8.75E-03 | SEPT2 | BMI |
| 5.3308 | 9.78E-08 | GTExv8.EUR.Whole_Blood | 2.21E-06 | RAD18 | BMI |
| -5.3826 | 7.34E-08 | GTExv8.EUR.Whole_Blood | 1.71E-06 | THUMPD3-AS1 | BMI |
| -3.7580 | 1.71E-04 | GTExv8.EUR.Whole_Blood | 1.55E-03 | PRRT3 | BMI |
| -3.7244 | 1.96E-04 | GTExv8.EUR.Whole_Blood | 1.73E-03 | FANCD2 | BMI |
| -4.3811 | 1.18E-05 | GTExv8.EUR.Whole_Blood | 1.55E-04 | BRK1 | BMI |
| -3.9851 | 6.75E-05 | GTExv8.EUR.Whole_Blood | 6.87E-04 | VHL | BMI |
| 2.5109 | 1.20E-02 | GTExv8.EUR.Whole_Blood | 4.94E-02 | GHRLOS | BMI |
| -3.3254 | 8.83E-04 | GTExv8.EUR.Whole_Blood | 6.24E-03 | ATG7 | BMI |
| -4.4955 | 6.94E-06 | GTExv8.EUR.Whole_Blood | 9.66E-05 | RP11-169K17.3 | BMI |
| 4.2689 | 1.96E-05 | GTExv8.EUR.Whole_Blood | 2.38E-04 | SYN2 | BMI |
| 3.5272 | 4.20E-04 | GTExv8.EUR.Whole_Blood | 3.32E-03 | MKRN2 | BMI |
| -4.6329 | 3.61E-06 | GTExv8.EUR.Whole_Blood | 5.39E-05 | NUP210 | BMI |
| 3.0648 | 2.18E-03 | GTExv8.EUR.Whole_Blood | 1.28E-02 | COLQ | BMI |
| 2.8217 | 4.78E-03 | GTExv8.EUR.Whole_Blood | 2.42E-02 | BTD | BMI |
| -2.7047 | 6.84E-03 | GTExv8.EUR.Whole_Blood | 3.19E-02 | OXNAD1 | BMI |
| -2.9478 | 3.20E-03 | GTExv8.EUR.Whole_Blood | 1.74E-02 | RP11-415F23.4 | BMI |
| -4.2262 | 2.38E-05 | GTExv8.EUR.Whole_Blood | 2.84E-04 | UBE2E2 | BMI |
| 2.9697 | 2.98E-03 | GTExv8.EUR.Whole_Blood | 1.65E-02 | TOP2B | BMI |
| 3.1776 | 1.48E-03 | GTExv8.EUR.Whole_Blood | 9.46E-03 | STT3B | BMI |
| -2.6359 | 8.39E-03 | GTExv8.EUR.Whole_Blood | 3.73E-02 | PDCD6IP | BMI |
| 2.8564 | 4.28E-03 | GTExv8.EUR.Whole_Blood | 2.22E-02 | LRRFIP2 | BMI |
| -2.6301 | 8.54E-03 | GTExv8.EUR.Whole_Blood | 3.78E-02 | ACVR2B | BMI |
| -5.4310 | 5.60E-08 | GTExv8.EUR.Whole_Blood | 1.34E-06 | EXOG | BMI |
| -2.9147 | 3.56E-03 | GTExv8.EUR.Whole_Blood | 1.90E-02 | SCN5A | BMI |
| -5.8779 | 4.15E-09 | GTExv8.EUR.Whole_Blood | 1.32E-07 | CTNNB1 | BMI |
| 2.6828 | 7.30E-03 | GTExv8.EUR.Whole_Blood | 3.34E-02 | ULK4 | BMI |
| -4.4897 | 7.13E-06 | GTExv8.EUR.Whole_Blood | 9.91E-05 | VIPR1-AS1 | BMI |
| 2.7746 | 5.53E-03 | GTExv8.EUR.Whole_Blood | 2.69E-02 | ZBTB47 | BMI |
| 2.7478 | 6.00E-03 | GTExv8.EUR.Whole_Blood | 2.88E-02 | ANO10 | BMI |
| -2.5451 | 1.09E-02 | GTExv8.EUR.Whole_Blood | 4.58E-02 | CDCP1 | BMI |
| 2.8269 | 4.70E-03 | GTExv8.EUR.Whole_Blood | 2.39E-02 | TMEM158 | BMI |
| 4.2082 | 2.57E-05 | GTExv8.EUR.Whole_Blood | 3.02E-04 | LIMD1 | BMI |
| -2.8473 | 4.41E-03 | GTExv8.EUR.Whole_Blood | 2.27E-02 | SACM1L | BMI |
| 2.7458 | 6.04E-03 | GTExv8.EUR.Whole_Blood | 2.89E-02 | XCR1 | BMI |
| 4.2873 | 1.81E-05 | GTExv8.EUR.Whole_Blood | 2.23E-04 | FLT1P1 | BMI |
| 4.3943 | 1.11E-05 | GTExv8.EUR.Whole_Blood | 1.46E-04 | CCR3 | BMI |
| -4.3177 | 1.58E-05 | GTExv8.EUR.Whole_Blood | 1.99E-04 | CCR1 | BMI |
| 5.6449 | 1.65E-08 | GTExv8.EUR.Whole_Blood | 4.54E-07 | ELP6 | BMI |
| -6.4586 | 1.06E-10 | GTExv8.EUR.Whole_Blood | 4.85E-09 | MAP4 | BMI |
| 2.9531 | 3.15E-03 | GTExv8.EUR.Whole_Blood | 1.72E-02 | TMA7 | BMI |
| -5.5377 | 3.06E-08 | GTExv8.EUR.Whole_Blood | 7.81E-07 | ATRIP | BMI |
| -5.8757 | 4.21E-09 | GTExv8.EUR.Whole_Blood | 1.34E-07 | DAG1 | BMI |
| -3.5698 | 3.57E-04 | GTExv8.EUR.Whole_Blood | 2.89E-03 | APEH | BMI |
| 7.9152 | 2.47E-15 | GTExv8.EUR.Whole_Blood | 2.46E-13 | GMPPB | BMI |
| -11.4805 | 1.65E-30 | GTExv8.EUR.Whole_Blood | 4.14E-28 | UBA7 | BMI |
| -9.5988 | 8.08E-22 | GTExv8.EUR.Whole_Blood | 1.10E-19 | MST1R | BMI |
| -10.8111 | 3.05E-27 | GTExv8.EUR.Whole_Blood | 5.79E-25 | MON1A | BMI |
| -13.3073 | 2.10E-40 | GTExv8.EUR.Whole_Blood | 1.02E-37 | RBM6 | BMI |
| -5.6505 | 1.60E-08 | GTExv8.EUR.Whole_Blood | 4.43E-07 | HYAL3 | BMI |
| -3.1474 | 1.65E-03 | GTExv8.EUR.Whole_Blood | 1.03E-02 | HEMK1 | BMI |
| 5.4166 | 6.08E-08 | GTExv8.EUR.Whole_Blood | 1.45E-06 | MAPKAPK3 | BMI |
| 5.8927 | 3.80E-09 | GTExv8.EUR.Whole_Blood | 1.24E-07 | LINC02019 | BMI |
| -4.7862 | 1.70E-06 | GTExv8.EUR.Whole_Blood | 2.71E-05 | TEX264 | BMI |
| -4.7862 | 1.70E-06 | GTExv8.EUR.Whole_Blood | 2.71E-05 | GRM2 | BMI |
| -4.3776 | 1.20E-05 | GTExv8.EUR.Whole_Blood | 1.57E-04 | GLYCTK | BMI |
| -4.3730 | 1.23E-05 | GTExv8.EUR.Whole_Blood | 1.60E-04 | GLYCTK-AS1 | BMI |
| -4.3145 | 1.60E-05 | GTExv8.EUR.Whole_Blood | 2.00E-04 | STAB1 | BMI |
| 6.3429 | 2.25E-10 | GTExv8.EUR.Whole_Blood | 9.31E-09 | ITIH3 | BMI |
| 5.0839 | 3.70E-07 | GTExv8.EUR.Whole_Blood | 7.02E-06 | ITIH4 | BMI |
| 5.4788 | 4.28E-08 | GTExv8.EUR.Whole_Blood | 1.06E-06 | MUSTN1 | BMI |
| 7.0140 | 2.32E-12 | GTExv8.EUR.Whole_Blood | 1.52E-10 | RP11-894J14.2 | BMI |
| 5.6815 | 1.34E-08 | GTExv8.EUR.Whole_Blood | 3.75E-07 | TMEM110 | BMI |
| 7.0623 | 1.64E-12 | GTExv8.EUR.Whole_Blood | 1.12E-10 | SFMBT1 | BMI |
| 4.0245 | 5.71E-05 | GTExv8.EUR.Whole_Blood | 6.00E-04 | SERBP1P3 | BMI |
| -3.9598 | 7.50E-05 | GTExv8.EUR.Whole_Blood | 7.53E-04 | RFT1 | BMI |
| 2.9290 | 3.40E-03 | GTExv8.EUR.Whole_Blood | 1.82E-02 | DCP1A | BMI |
| -2.9772 | 2.91E-03 | GTExv8.EUR.Whole_Blood | 1.62E-02 | IL17RB | BMI |
| 2.9880 | 2.81E-03 | GTExv8.EUR.Whole_Blood | 1.57E-02 | RPP14 | BMI |
| -3.1668 | 1.54E-03 | GTExv8.EUR.Whole_Blood | 9.76E-03 | PXK | BMI |
| -2.9339 | 3.35E-03 | GTExv8.EUR.Whole_Blood | 1.80E-02 | C3orf14 | BMI |
| 3.5616 | 3.69E-04 | GTExv8.EUR.Whole_Blood | 2.97E-03 | RYBP | BMI |
| -3.0963 | 1.96E-03 | GTExv8.EUR.Whole_Blood | 1.18E-02 | GBE1 | BMI |
| 7.7431 | 9.70E-15 | GTExv8.EUR.Whole_Blood | 8.58E-13 | C3orf38 | BMI |
| 3.1559 | 1.60E-03 | GTExv8.EUR.Whole_Blood | 1.01E-02 | CMSS1 | BMI |
| -2.7118 | 6.69E-03 | GTExv8.EUR.Whole_Blood | 3.15E-02 | IMPG2 | BMI |
| -3.5184 | 4.34E-04 | GTExv8.EUR.Whole_Blood | 3.41E-03 | SENP7 | BMI |
| 3.6593 | 2.53E-04 | GTExv8.EUR.Whole_Blood | 2.16E-03 | CEP97 | BMI |
| 4.2565 | 2.08E-05 | GTExv8.EUR.Whole_Blood | 2.51E-04 | ALCAM | BMI |
| 4.4279 | 9.51E-06 | GTExv8.EUR.Whole_Blood | 1.28E-04 | BBX | BMI |
| -2.9878 | 2.81E-03 | GTExv8.EUR.Whole_Blood | 1.57E-02 | CD47 | BMI |
| -5.0619 | 4.15E-07 | GTExv8.EUR.Whole_Blood | 7.74E-06 | HHLA2 | BMI |
| 5.7323 | 9.91E-09 | GTExv8.EUR.Whole_Blood | 2.86E-07 | DZIP3 | BMI |
| -2.9415 | 3.27E-03 | GTExv8.EUR.Whole_Blood | 1.77E-02 | SIDT1 | BMI |
| 2.6711 | 7.56E-03 | GTExv8.EUR.Whole_Blood | 3.44E-02 | TIMMDC1 | BMI |
| 3.3043 | 9.52E-04 | GTExv8.EUR.Whole_Blood | 6.65E-03 | GTF2E1 | BMI |
| 3.2173 | 1.29E-03 | GTExv8.EUR.Whole_Blood | 8.51E-03 | HCLS1 | BMI |
| -4.2045 | 2.62E-05 | GTExv8.EUR.Whole_Blood | 3.06E-04 | IQCB1 | BMI |
| -2.5486 | 1.08E-02 | GTExv8.EUR.Whole_Blood | 4.56E-02 | HSPBAP1 | BMI |
| -3.6658 | 2.47E-04 | GTExv8.EUR.Whole_Blood | 2.11E-03 | LINC02035 | BMI |
| -6.0608 | 1.35E-09 | GTExv8.EUR.Whole_Blood | 4.78E-08 | HACD2 | BMI |
| -2.9397 | 3.29E-03 | GTExv8.EUR.Whole_Blood | 1.78E-02 | HEG1 | BMI |
| -2.9736 | 2.94E-03 | GTExv8.EUR.Whole_Blood | 1.63E-02 | FAM86JP | BMI |
| 2.7214 | 6.50E-03 | GTExv8.EUR.Whole_Blood | 3.08E-02 | EEFSEC | BMI |
| -3.0902 | 2.00E-03 | GTExv8.EUR.Whole_Blood | 1.20E-02 | HMCES | BMI |
| 6.7200 | 1.82E-11 | GTExv8.EUR.Whole_Blood | 1.00E-09 | IL20RB | BMI |
| 2.6708 | 7.57E-03 | GTExv8.EUR.Whole_Blood | 3.44E-02 | ZBTB38 | BMI |
| 3.6068 | 3.10E-04 | GTExv8.EUR.Whole_Blood | 2.56E-03 | ATP1B3 | BMI |
| 3.4975 | 4.70E-04 | GTExv8.EUR.Whole_Blood | 3.64E-03 | CHST2 | BMI |
| 2.9193 | 3.51E-03 | GTExv8.EUR.Whole_Blood | 1.87E-02 | RP11-80H8.4 | BMI |
| 3.9933 | 6.52E-05 | GTExv8.EUR.Whole_Blood | 6.68E-04 | GYG1 | BMI |
| 3.6074 | 3.09E-04 | GTExv8.EUR.Whole_Blood | 2.56E-03 | HLTF | BMI |
| -2.6236 | 8.70E-03 | GTExv8.EUR.Whole_Blood | 3.84E-02 | PFN2 | BMI |
| -2.8778 | 4.00E-03 | GTExv8.EUR.Whole_Blood | 2.09E-02 | RP11-166N6.2 | BMI |
| -5.0801 | 3.77E-07 | GTExv8.EUR.Whole_Blood | 7.12E-06 | DHX36 | BMI |
| 5.9590 | 2.54E-09 | GTExv8.EUR.Whole_Blood | 8.56E-08 | CCNL1 | BMI |
| -5.7078 | 1.14E-08 | GTExv8.EUR.Whole_Blood | 3.25E-07 | KRT18P34 | BMI |
| 5.6302 | 1.80E-08 | GTExv8.EUR.Whole_Blood | 4.90E-07 | RP11-550I24.2 | BMI |
| -3.8082 | 1.40E-04 | GTExv8.EUR.Whole_Blood | 1.31E-03 | SHOX2 | BMI |
| -4.2586 | 2.06E-05 | GTExv8.EUR.Whole_Blood | 2.50E-04 | RSRC1 | BMI |
| 4.0907 | 4.30E-05 | GTExv8.EUR.Whole_Blood | 4.68E-04 | RPL22L1 | BMI |
| 2.8909 | 3.84E-03 | GTExv8.EUR.Whole_Blood | 2.02E-02 | PLD1 | BMI |
| -2.6124 | 8.99E-03 | GTExv8.EUR.Whole_Blood | 3.94E-02 | ZMAT3 | BMI |
| 2.5113 | 1.20E-02 | GTExv8.EUR.Whole_Blood | 4.94E-02 | MCCC1 | BMI |
| 5.2912 | 1.21E-07 | GTExv8.EUR.Whole_Blood | 2.66E-06 | KLHL24 | BMI |
| -6.1579 | 7.37E-10 | GTExv8.EUR.Whole_Blood | 2.80E-08 | YEATS2-AS1 | BMI |
| -2.6430 | 8.22E-03 | GTExv8.EUR.Whole_Blood | 3.67E-02 | PARL | BMI |
| 4.3546 | 1.33E-05 | GTExv8.EUR.Whole_Blood | 1.72E-04 | ABCC5 | BMI |
| 4.1193 | 3.80E-05 | GTExv8.EUR.Whole_Blood | 4.22E-04 | DVL3 | BMI |
| 2.5785 | 9.92E-03 | GTExv8.EUR.Whole_Blood | 4.28E-02 | AP2M1 | BMI |
| 2.6338 | 8.44E-03 | GTExv8.EUR.Whole_Blood | 3.75E-02 | ABCF3 | BMI |
| 4.0984 | 4.16E-05 | GTExv8.EUR.Whole_Blood | 4.57E-04 | PSMD2 | BMI |
| 3.9080 | 9.31E-05 | GTExv8.EUR.Whole_Blood | 9.14E-04 | NMRAL2P | BMI |
| 3.0374 | 2.39E-03 | GTExv8.EUR.Whole_Blood | 1.38E-02 | ATP13A4 | BMI |
| 2.5480 | 1.08E-02 | GTExv8.EUR.Whole_Blood | 4.56E-02 | OPA1 | BMI |
| 4.2154 | 2.49E-05 | GTExv8.EUR.Whole_Blood | 2.94E-04 | LSG1 | BMI |
| 2.6033 | 9.23E-03 | GTExv8.EUR.Whole_Blood | 4.02E-02 | MUC20 | BMI |
| -3.8044 | 1.42E-04 | GTExv8.EUR.Whole_Blood | 1.32E-03 | TNK2 | BMI |
| 4.4720 | 7.75E-06 | GTExv8.EUR.Whole_Blood | 1.06E-04 | Y_RNA | BMI |
| 3.0501 | 2.29E-03 | GTExv8.EUR.Whole_Blood | 1.33E-02 | TCTEX1D2 | BMI |
| 5.7477 | 9.04E-09 | GTExv8.EUR.Whole_Blood | 2.65E-07 | TM4SF19-AS1 | BMI |
| 5.2526 | 1.50E-07 | GTExv8.EUR.Whole_Blood | 3.22E-06 | TM4SF19 | BMI |
| 6.4159 | 1.40E-10 | GTExv8.EUR.Whole_Blood | 6.16E-09 | UBXN7 | BMI |
| -4.7184 | 2.38E-06 | GTExv8.EUR.Whole_Blood | 3.69E-05 | MELTF | BMI |
| 3.6262 | 2.88E-04 | GTExv8.EUR.Whole_Blood | 2.41E-03 | PDE6B | BMI |
| -3.7485 | 1.78E-04 | GTExv8.EUR.Whole_Blood | 1.60E-03 | RP11-1191J2.2 | BMI |
| 3.6690 | 2.44E-04 | GTExv8.EUR.Whole_Blood | 2.09E-03 | RP11-1191J2.5 | BMI |
| -3.3727 | 7.44E-04 | GTExv8.EUR.Whole_Blood | 5.38E-03 | MYL5 | BMI |
| 3.0616 | 2.20E-03 | GTExv8.EUR.Whole_Blood | 1.29E-02 | GAK | BMI |
| -2.5473 | 1.09E-02 | GTExv8.EUR.Whole_Blood | 4.58E-02 | IDUA | BMI |
| 3.1230 | 1.79E-03 | GTExv8.EUR.Whole_Blood | 1.10E-02 | SLC26A1 | BMI |
| -2.5572 | 1.06E-02 | GTExv8.EUR.Whole_Blood | 4.50E-02 | RP11-20I20.4 | BMI |
| 4.1546 | 3.26E-05 | GTExv8.EUR.Whole_Blood | 3.69E-04 | ZFYVE28 | BMI |
| -4.4141 | 1.01E-05 | GTExv8.EUR.Whole_Blood | 1.34E-04 | ADD1 | BMI |
| 2.6334 | 8.45E-03 | GTExv8.EUR.Whole_Blood | 3.75E-02 | MFSD10 | BMI |
| 4.6206 | 3.83E-06 | GTExv8.EUR.Whole_Blood | 5.63E-05 | GRK4 | BMI |
| 3.7456 | 1.80E-04 | GTExv8.EUR.Whole_Blood | 1.61E-03 | HTT | BMI |
| -3.3446 | 8.24E-04 | GTExv8.EUR.Whole_Blood | 5.89E-03 | RGS12 | BMI |
| -4.0936 | 4.25E-05 | GTExv8.EUR.Whole_Blood | 4.64E-04 | CYTL1 | BMI |
| -3.1943 | 1.40E-03 | GTExv8.EUR.Whole_Blood | 9.03E-03 | GRPEL1 | BMI |
| -2.5906 | 9.58E-03 | GTExv8.EUR.Whole_Blood | 4.15E-02 | AFAP1 | BMI |
| -4.6257 | 3.73E-06 | GTExv8.EUR.Whole_Blood | 5.53E-05 | DCAF16 | BMI |
| -3.4435 | 5.74E-04 | GTExv8.EUR.Whole_Blood | 4.34E-03 | SEPSECS-AS1 | BMI |
| 6.9495 | 3.66E-12 | GTExv8.EUR.Whole_Blood | 2.24E-10 | ZCCHC4 | BMI |
| 7.7645 | 8.20E-15 | GTExv8.EUR.Whole_Blood | 7.42E-13 | ANAPC4 | BMI |
| 3.4642 | 5.32E-04 | GTExv8.EUR.Whole_Blood | 4.05E-03 | RP11-293A21.1 | BMI |
| -3.1917 | 1.41E-03 | GTExv8.EUR.Whole_Blood | 9.08E-03 | RP11-734I18.1 | BMI |
| -2.7542 | 5.88E-03 | GTExv8.EUR.Whole_Blood | 2.84E-02 | RP11-177C12.1 | BMI |
| 2.7991 | 5.12E-03 | GTExv8.EUR.Whole_Blood | 2.54E-02 | KLF3-AS1 | BMI |
| -3.9841 | 6.77E-05 | GTExv8.EUR.Whole_Blood | 6.88E-04 | TLR10 | BMI |
| 3.3393 | 8.40E-04 | GTExv8.EUR.Whole_Blood | 5.99E-03 | FAM114A1 | BMI |
| -3.5609 | 3.70E-04 | GTExv8.EUR.Whole_Blood | 2.97E-03 | GUF1 | BMI |
| 2.7501 | 5.96E-03 | GTExv8.EUR.Whole_Blood | 2.87E-02 | CORIN | BMI |
| 2.6288 | 8.57E-03 | GTExv8.EUR.Whole_Blood | 3.79E-02 | RP11-121C2.2 | BMI |
| 2.7658 | 5.68E-03 | GTExv8.EUR.Whole_Blood | 2.75E-02 | NFXL1 | BMI |
| 4.4172 | 1.00E-05 | GTExv8.EUR.Whole_Blood | 1.33E-04 | DANCR | BMI |
| 2.5115 | 1.20E-02 | GTExv8.EUR.Whole_Blood | 4.94E-02 | SRP72 | BMI |
| -5.2228 | 1.76E-07 | GTExv8.EUR.Whole_Blood | 3.68E-06 | CENPC | BMI |
| -3.0115 | 2.60E-03 | GTExv8.EUR.Whole_Blood | 1.47E-02 | ADAMTS3 | BMI |
| -2.9383 | 3.30E-03 | GTExv8.EUR.Whole_Blood | 1.78E-02 | RCHY1 | BMI |
| -3.1779 | 1.48E-03 | GTExv8.EUR.Whole_Blood | 9.46E-03 | STBD1 | BMI |
| -3.7380 | 1.86E-04 | GTExv8.EUR.Whole_Blood | 1.65E-03 | HPSE | BMI |
| -5.4226 | 5.87E-08 | GTExv8.EUR.Whole_Blood | 1.40E-06 | FLJ20021 | BMI |
| 5.2407 | 1.60E-07 | GTExv8.EUR.Whole_Blood | 3.39E-06 | KRT8P46 | BMI |
| 5.2407 | 1.60E-07 | GTExv8.EUR.Whole_Blood | 3.39E-06 | LRRC37A15P | BMI |
| 6.1535 | 7.58E-10 | GTExv8.EUR.Whole_Blood | 2.86E-08 | RP11-10L12.1 | BMI |
| 5.2407 | 1.60E-07 | GTExv8.EUR.Whole_Blood | 3.39E-06 | RP11-10L12.2 | BMI |
| 4.1089 | 3.98E-05 | GTExv8.EUR.Whole_Blood | 4.39E-04 | MANBA | BMI |
| -3.5065 | 4.54E-04 | GTExv8.EUR.Whole_Blood | 3.53E-03 | BDH2 | BMI |
| 2.6485 | 8.08E-03 | GTExv8.EUR.Whole_Blood | 3.62E-02 | TET2 | BMI |
| -3.4362 | 5.90E-04 | GTExv8.EUR.Whole_Blood | 4.44E-03 | SNHG8 | BMI |
| 4.9766 | 6.47E-07 | GTExv8.EUR.Whole_Blood | 1.14E-05 | C4orf3 | BMI |
| -3.1946 | 1.40E-03 | GTExv8.EUR.Whole_Blood | 9.03E-03 | FABP2 | BMI |
| 4.7636 | 1.90E-06 | GTExv8.EUR.Whole_Blood | 3.00E-05 | GTF2IP12 | BMI |
| -5.1849 | 2.16E-07 | GTExv8.EUR.Whole_Blood | 4.40E-06 | RP11-33B1.1 | BMI |
| 2.6357 | 8.40E-03 | GTExv8.EUR.Whole_Blood | 3.74E-02 | EXOSC9 | BMI |
| 3.2916 | 9.96E-04 | GTExv8.EUR.Whole_Blood | 6.84E-03 | ELF2 | BMI |
| -3.6982 | 2.17E-04 | GTExv8.EUR.Whole_Blood | 1.90E-03 | MAML3 | BMI |
| 2.9991 | 2.71E-03 | GTExv8.EUR.Whole_Blood | 1.53E-02 | ZNF330 | BMI |
| 3.0385 | 2.38E-03 | GTExv8.EUR.Whole_Blood | 1.38E-02 | RP11-362F19.1 | BMI |
| 3.0618 | 2.20E-03 | GTExv8.EUR.Whole_Blood | 1.29E-02 | IL15 | BMI |
| -2.9576 | 3.10E-03 | GTExv8.EUR.Whole_Blood | 1.70E-02 | RP11-223C24.1 | BMI |
| -4.0045 | 6.22E-05 | GTExv8.EUR.Whole_Blood | 6.45E-04 | INPP4B | BMI |
| -5.5705 | 2.54E-08 | GTExv8.EUR.Whole_Blood | 6.66E-07 | USP38 | BMI |
| 2.7431 | 6.09E-03 | GTExv8.EUR.Whole_Blood | 2.91E-02 | GYPB | BMI |
| -2.5121 | 1.20E-02 | GTExv8.EUR.Whole_Blood | 4.94E-02 | LSM6 | BMI |
| -3.7231 | 1.97E-04 | GTExv8.EUR.Whole_Blood | 1.74E-03 | RP11-6L6.4 | BMI |
| 3.7070 | 2.10E-04 | GTExv8.EUR.Whole_Blood | 1.84E-03 | RP11-6L6.2 | BMI |
| -3.4002 | 6.73E-04 | GTExv8.EUR.Whole_Blood | 4.94E-03 | RPS3A | BMI |
| -3.7382 | 1.85E-04 | GTExv8.EUR.Whole_Blood | 1.65E-03 | FNIP2 | BMI |
| 4.1675 | 3.08E-05 | GTExv8.EUR.Whole_Blood | 3.54E-04 | RAPGEF2 | BMI |
| -4.3768 | 1.20E-05 | GTExv8.EUR.Whole_Blood | 1.57E-04 | RP11-218F10.3 | BMI |
| -3.3226 | 8.92E-04 | GTExv8.EUR.Whole_Blood | 6.30E-03 | CEP44 | BMI |
| -3.7255 | 1.95E-04 | GTExv8.EUR.Whole_Blood | 1.72E-03 | CCDC127 | BMI |
| -4.5094 | 6.50E-06 | GTExv8.EUR.Whole_Blood | 9.11E-05 | SDHA | BMI |
| -4.1660 | 3.10E-05 | GTExv8.EUR.Whole_Blood | 3.55E-04 | CTD-2083E4.5 | BMI |
| -2.6553 | 7.92E-03 | GTExv8.EUR.Whole_Blood | 3.57E-02 | CTD-2228K2.7 | BMI |
| 2.7548 | 5.87E-03 | GTExv8.EUR.Whole_Blood | 2.83E-02 | MRPL36 | BMI |
| -3.6031 | 3.14E-04 | GTExv8.EUR.Whole_Blood | 2.59E-03 | ANXA2R | BMI |
| 2.9334 | 3.35E-03 | GTExv8.EUR.Whole_Blood | 1.80E-02 | NNT | BMI |
| -4.0689 | 4.72E-05 | GTExv8.EUR.Whole_Blood | 5.07E-04 | SETD9 | BMI |
| -2.9584 | 3.09E-03 | GTExv8.EUR.Whole_Blood | 1.70E-02 | CTD-2310F14.1 | BMI |
| -2.6220 | 8.74E-03 | GTExv8.EUR.Whole_Blood | 3.86E-02 | RP11-231G3.1 | BMI |
| -2.5531 | 1.07E-02 | GTExv8.EUR.Whole_Blood | 4.53E-02 | ERCC8 | BMI |
| -2.8647 | 4.17E-03 | GTExv8.EUR.Whole_Blood | 2.16E-02 | NLN | BMI |
| -2.6936 | 7.07E-03 | GTExv8.EUR.Whole_Blood | 3.28E-02 | CD180 | BMI |
| -8.5400 | 1.34E-17 | GTExv8.EUR.Whole_Blood | 1.45E-15 | POC5 | BMI |
| -3.9906 | 6.59E-05 | GTExv8.EUR.Whole_Blood | 6.75E-04 | AP3B1 | BMI |
| 5.5733 | 2.50E-08 | GTExv8.EUR.Whole_Blood | 6.59E-07 | SCAMP1 | BMI |
| 2.6520 | 8.00E-03 | GTExv8.EUR.Whole_Blood | 3.59E-02 | RP11-343L5.2 | BMI |
| 4.2471 | 2.17E-05 | GTExv8.EUR.Whole_Blood | 2.61E-04 | TMEM161B-AS1 | BMI |
| 2.5163 | 1.19E-02 | GTExv8.EUR.Whole_Blood | 4.93E-02 | TTC37 | BMI |
| 5.2787 | 1.30E-07 | GTExv8.EUR.Whole_Blood | 2.82E-06 | GLRX | BMI |
| -3.6348 | 2.78E-04 | GTExv8.EUR.Whole_Blood | 2.34E-03 | CAST | BMI |
| -5.4989 | 3.82E-08 | GTExv8.EUR.Whole_Blood | 9.56E-07 | CTD-2260A17.3 | BMI |
| -6.2175 | 5.05E-10 | GTExv8.EUR.Whole_Blood | 1.96E-08 | ERAP1 | BMI |
| -3.9981 | 6.38E-05 | GTExv8.EUR.Whole_Blood | 6.58E-04 | LINC02062 | BMI |
| 4.9787 | 6.40E-07 | GTExv8.EUR.Whole_Blood | 1.13E-05 | PAM | BMI |
| -4.1546 | 3.26E-05 | GTExv8.EUR.Whole_Blood | 3.69E-04 | GIN1 | BMI |
| -3.2829 | 1.03E-03 | GTExv8.EUR.Whole_Blood | 7.04E-03 | PPIP5K2 | BMI |
| -5.2199 | 1.79E-07 | GTExv8.EUR.Whole_Blood | 3.73E-06 | PJA2 | BMI |
| 3.1559 | 1.60E-03 | GTExv8.EUR.Whole_Blood | 1.01E-02 | MAN2A1 | BMI |
| 3.3985 | 6.78E-04 | GTExv8.EUR.Whole_Blood | 4.97E-03 | SRP19 | BMI |
| 5.8107 | 6.22E-09 | GTExv8.EUR.Whole_Blood | 1.89E-07 | CTC-487M23.5 | BMI |
| 3.1474 | 1.65E-03 | GTExv8.EUR.Whole_Blood | 1.03E-02 | CTC-487M23.6 | BMI |
| 4.9981 | 5.79E-07 | GTExv8.EUR.Whole_Blood | 1.03E-05 | REEP5 | BMI |
| 2.8948 | 3.79E-03 | GTExv8.EUR.Whole_Blood | 2.00E-02 | MCC | BMI |
| 4.3052 | 1.67E-05 | GTExv8.EUR.Whole_Blood | 2.08E-04 | YTHDC2 | BMI |
| -2.8371 | 4.55E-03 | GTExv8.EUR.Whole_Blood | 2.33E-02 | CTD-2287O16.5 | BMI |
| -4.1879 | 2.82E-05 | GTExv8.EUR.Whole_Blood | 3.27E-04 | TNFAIP8 | BMI |
| -4.2568 | 2.07E-05 | GTExv8.EUR.Whole_Blood | 2.51E-04 | HSD17B4 | BMI |
| -2.5124 | 1.20E-02 | GTExv8.EUR.Whole_Blood | 4.94E-02 | LINC01184 | BMI |
| 3.1214 | 1.80E-03 | GTExv8.EUR.Whole_Blood | 1.10E-02 | LYRM7 | BMI |
| -4.1555 | 3.25E-05 | GTExv8.EUR.Whole_Blood | 3.69E-04 | RAPGEF6 | BMI |
| -3.2134 | 1.31E-03 | GTExv8.EUR.Whole_Blood | 8.60E-03 | MEIKIN | BMI |
| -3.2105 | 1.32E-03 | GTExv8.EUR.Whole_Blood | 8.64E-03 | ACSL6 | BMI |
| -3.8624 | 1.12E-04 | GTExv8.EUR.Whole_Blood | 1.08E-03 | AC034220.3 | BMI |
| 2.9577 | 3.10E-03 | GTExv8.EUR.Whole_Blood | 1.70E-02 | SAR1B | BMI |
| 3.1947 | 1.40E-03 | GTExv8.EUR.Whole_Blood | 9.03E-03 | CAMLG | BMI |
| 4.1735 | 3.00E-05 | GTExv8.EUR.Whole_Blood | 3.45E-04 | TXNDC15 | BMI |
| -2.8480 | 4.40E-03 | GTExv8.EUR.Whole_Blood | 2.27E-02 | SMAD5 | BMI |
| -4.4830 | 7.36E-06 | GTExv8.EUR.Whole_Blood | 1.02E-04 | ETF1 | BMI |
| 2.7124 | 6.68E-03 | GTExv8.EUR.Whole_Blood | 3.14E-02 | CTNNA1 | BMI |
| -4.7656 | 1.88E-06 | GTExv8.EUR.Whole_Blood | 2.97E-05 | SLC4A9 | BMI |
| -3.0205 | 2.52E-03 | GTExv8.EUR.Whole_Blood | 1.44E-02 | TMCO6 | BMI |
| -3.6014 | 3.17E-04 | GTExv8.EUR.Whole_Blood | 2.61E-03 | IK | BMI |
| 4.6012 | 4.20E-06 | GTExv8.EUR.Whole_Blood | 6.13E-05 | NDUFA2 | BMI |
| -2.7534 | 5.90E-03 | GTExv8.EUR.Whole_Blood | 2.84E-02 | WDR55 | BMI |
| -2.7866 | 5.33E-03 | GTExv8.EUR.Whole_Blood | 2.62E-02 | ARAP3 | BMI |
| -3.5112 | 4.46E-04 | GTExv8.EUR.Whole_Blood | 3.48E-03 | LARS | BMI |
| -2.5831 | 9.79E-03 | GTExv8.EUR.Whole_Blood | 4.23E-02 | PCYOX1L | BMI |
| 3.0239 | 2.50E-03 | GTExv8.EUR.Whole_Blood | 1.43E-02 | RPS14 | BMI |
| 6.0426 | 1.52E-09 | GTExv8.EUR.Whole_Blood | 5.28E-08 | FAM114A2 | BMI |
| -2.6904 | 7.14E-03 | GTExv8.EUR.Whole_Blood | 3.30E-02 | PANK3 | BMI |
| -5.8124 | 6.16E-09 | GTExv8.EUR.Whole_Blood | 1.88E-07 | HMP19 | BMI |
| -3.0685 | 2.15E-03 | GTExv8.EUR.Whole_Blood | 1.27E-02 | THOC3 | BMI |
| -2.8337 | 4.60E-03 | GTExv8.EUR.Whole_Blood | 2.34E-02 | FAF2 | BMI |
| 3.8424 | 1.22E-04 | GTExv8.EUR.Whole_Blood | 1.16E-03 | GPRIN1 | BMI |
| -2.7163 | 6.60E-03 | GTExv8.EUR.Whole_Blood | 3.12E-02 | UIMC1 | BMI |
| 3.7067 | 2.10E-04 | GTExv8.EUR.Whole_Blood | 1.84E-03 | ZNF879 | BMI |
| -3.1947 | 1.40E-03 | GTExv8.EUR.Whole_Blood | 9.03E-03 | MGAT4B | BMI |
| 3.5755 | 3.50E-04 | GTExv8.EUR.Whole_Blood | 2.84E-03 | LINC00847 | BMI |
| 3.0056 | 2.65E-03 | GTExv8.EUR.Whole_Blood | 1.50E-02 | ZFP62 | BMI |
| -3.0164 | 2.56E-03 | GTExv8.EUR.Whole_Blood | 1.46E-02 | RACK1 | BMI |
| 2.6197 | 8.80E-03 | GTExv8.EUR.Whole_Blood | 3.87E-02 | RP11-157J24.2 | BMI |
| -4.6575 | 3.20E-06 | GTExv8.EUR.Whole_Blood | 4.84E-05 | WRNIP1 | BMI |
| 2.7689 | 5.63E-03 | GTExv8.EUR.Whole_Blood | 2.73E-02 | LYRM4 | BMI |
| 4.1764 | 2.96E-05 | GTExv8.EUR.Whole_Blood | 3.42E-04 | FARS2 | BMI |
| 5.3929 | 6.93E-08 | GTExv8.EUR.Whole_Blood | 1.62E-06 | NRN1 | BMI |
| -2.5121 | 1.20E-02 | GTExv8.EUR.Whole_Blood | 4.94E-02 | GCNT2 | BMI |
| 2.9519 | 3.16E-03 | GTExv8.EUR.Whole_Blood | 1.73E-02 | RP1-257A7.4 | BMI |
| 2.5758 | 1.00E-02 | GTExv8.EUR.Whole_Blood | 4.29E-02 | TBC1D7 | BMI |
| 4.1260 | 3.69E-05 | GTExv8.EUR.Whole_Blood | 4.11E-04 | JARID2 | BMI |
| -4.9552 | 7.22E-07 | GTExv8.EUR.Whole_Blood | 1.26E-05 | C6orf62 | BMI |
| 2.7109 | 6.71E-03 | GTExv8.EUR.Whole_Blood | 3.15E-02 | C6orf229 | BMI |
| 2.6396 | 8.30E-03 | GTExv8.EUR.Whole_Blood | 3.70E-02 | FAM65B | BMI |
| 3.9138 | 9.08E-05 | GTExv8.EUR.Whole_Blood | 8.97E-04 | PPIAP29 | BMI |
| 11.4833 | 1.60E-30 | GTExv8.EUR.Whole_Blood | 4.14E-28 | C6orf106 | BMI |
| -13.2051 | 8.20E-40 | GTExv8.EUR.Whole_Blood | 3.75E-37 | SNRPC | BMI |
| 13.8473 | 1.32E-43 | GTExv8.EUR.Whole_Blood | 7.34E-41 | UHRF1BP1 | BMI |
| -3.3539 | 7.97E-04 | GTExv8.EUR.Whole_Blood | 5.71E-03 | MDGA1 | BMI |
| 3.0871 | 2.02E-03 | GTExv8.EUR.Whole_Blood | 1.21E-02 | GLO1 | BMI |
| 2.9677 | 3.00E-03 | GTExv8.EUR.Whole_Blood | 1.66E-02 | KCNK17 | BMI |
| 3.0517 | 2.28E-03 | GTExv8.EUR.Whole_Blood | 1.33E-02 | MOCS1 | BMI |
| 7.8166 | 5.42E-15 | GTExv8.EUR.Whole_Blood | 4.96E-13 | LRFN2 | BMI |
| 4.8710 | 1.11E-06 | GTExv8.EUR.Whole_Blood | 1.85E-05 | OARD1 | BMI |
| 3.4111 | 6.47E-04 | GTExv8.EUR.Whole_Blood | 4.80E-03 | ADCY10P1 | BMI |
| -3.0232 | 2.50E-03 | GTExv8.EUR.Whole_Blood | 1.43E-02 | USP49 | BMI |
| -3.8674 | 1.10E-04 | GTExv8.EUR.Whole_Blood | 1.06E-03 | BYSL | BMI |
| -2.5427 | 1.10E-02 | GTExv8.EUR.Whole_Blood | 4.60E-02 | GNMT | BMI |
| 5.0296 | 4.92E-07 | GTExv8.EUR.Whole_Blood | 8.90E-06 | ZNF318 | BMI |
| -3.2272 | 1.25E-03 | GTExv8.EUR.Whole_Blood | 8.29E-03 | TMEM63B | BMI |
| -3.4191 | 6.28E-04 | GTExv8.EUR.Whole_Blood | 4.66E-03 | PLA2G7 | BMI |
| -3.0266 | 2.47E-03 | GTExv8.EUR.Whole_Blood | 1.42E-02 | CENPQ | BMI |
| 2.6443 | 8.19E-03 | GTExv8.EUR.Whole_Blood | 3.66E-02 | ELOVL5 | BMI |
| 3.7789 | 1.58E-04 | GTExv8.EUR.Whole_Blood | 1.45E-03 | RAB23 | BMI |
| 3.0243 | 2.49E-03 | GTExv8.EUR.Whole_Blood | 1.43E-02 | LINC00680 | BMI |
| 5.7502 | 8.91E-09 | GTExv8.EUR.Whole_Blood | 2.63E-07 | LGSN | BMI |
| -4.9052 | 9.33E-07 | GTExv8.EUR.Whole_Blood | 1.58E-05 | SDHAF4 | BMI |
| 3.2945 | 9.86E-04 | GTExv8.EUR.Whole_Blood | 6.81E-03 | SENP6 | BMI |
| 3.0710 | 2.13E-03 | GTExv8.EUR.Whole_Blood | 1.26E-02 | PHIP | BMI |
| 2.6219 | 8.75E-03 | GTExv8.EUR.Whole_Blood | 3.86E-02 | NT5E | BMI |
| -2.5758 | 1.00E-02 | GTExv8.EUR.Whole_Blood | 4.29E-02 | SNHG5 | BMI |
| 2.6856 | 7.24E-03 | GTExv8.EUR.Whole_Blood | 3.33E-02 | ZNF292 | BMI |
| -2.8922 | 3.83E-03 | GTExv8.EUR.Whole_Blood | 2.01E-02 | SLC35A1 | BMI |
| 2.9889 | 2.80E-03 | GTExv8.EUR.Whole_Blood | 1.56E-02 | GABRR2 | BMI |
| 3.9087 | 9.28E-05 | GTExv8.EUR.Whole_Blood | 9.12E-04 | MMS22L | BMI |
| 3.0733 | 2.12E-03 | GTExv8.EUR.Whole_Blood | 1.26E-02 | ASCC3 | BMI |
| -4.5778 | 4.70E-06 | GTExv8.EUR.Whole_Blood | 6.76E-05 | CCDC162P | BMI |
| -3.9891 | 6.63E-05 | GTExv8.EUR.Whole_Blood | 6.77E-04 | CD164 | BMI |
| -2.7943 | 5.20E-03 | GTExv8.EUR.Whole_Blood | 2.57E-02 | SMPD2 | BMI |
| -3.1211 | 1.80E-03 | GTExv8.EUR.Whole_Blood | 1.10E-02 | ZBTB24 | BMI |
| 3.6815 | 2.32E-04 | GTExv8.EUR.Whole_Blood | 2.01E-03 | TRAF3IP2-AS1 | BMI |
| -3.5536 | 3.80E-04 | GTExv8.EUR.Whole_Blood | 3.04E-03 | ZUFSP | BMI |
| -2.5842 | 9.76E-03 | GTExv8.EUR.Whole_Blood | 4.21E-02 | DCBLD1 | BMI |
| 3.5401 | 4.00E-04 | GTExv8.EUR.Whole_Blood | 3.19E-03 | MCM9 | BMI |
| -2.5427 | 1.10E-02 | GTExv8.EUR.Whole_Blood | 4.60E-02 | MAN1A1 | BMI |
| -4.1640 | 3.13E-05 | GTExv8.EUR.Whole_Blood | 3.57E-04 | ECHDC1 | BMI |
| -2.8171 | 4.85E-03 | GTExv8.EUR.Whole_Blood | 2.44E-02 | KIAA0408 | BMI |
| 6.4687 | 9.89E-11 | GTExv8.EUR.Whole_Blood | 4.58E-09 | L3MBTL3 | BMI |
| -2.9923 | 2.77E-03 | GTExv8.EUR.Whole_Blood | 1.55E-02 | EPB41L2 | BMI |
| -7.2900 | 3.10E-13 | GTExv8.EUR.Whole_Blood | 2.51E-11 | ARG1 | BMI |
| -7.0033 | 2.50E-12 | GTExv8.EUR.Whole_Blood | 1.59E-10 | MED23 | BMI |
| 2.6612 | 7.79E-03 | GTExv8.EUR.Whole_Blood | 3.52E-02 | STX7 | BMI |
| -2.6039 | 9.22E-03 | GTExv8.EUR.Whole_Blood | 4.02E-02 | AHI1 | BMI |
| 2.8248 | 4.73E-03 | GTExv8.EUR.Whole_Blood | 2.39E-02 | MAP3K5 | BMI |
| 3.5818 | 3.41E-04 | GTExv8.EUR.Whole_Blood | 2.78E-03 | RAB32 | BMI |
| -3.3263 | 8.80E-04 | GTExv8.EUR.Whole_Blood | 6.23E-03 | STXBP5 | BMI |
| 3.6838 | 2.30E-04 | GTExv8.EUR.Whole_Blood | 1.99E-03 | RGS17 | BMI |
| -5.4135 | 6.18E-08 | GTExv8.EUR.Whole_Blood | 1.47E-06 | CAHM | BMI |
| -4.1186 | 3.81E-05 | GTExv8.EUR.Whole_Blood | 4.23E-04 | QKI | BMI |
| 3.8674 | 1.10E-04 | GTExv8.EUR.Whole_Blood | 1.06E-03 | MRM2 | BMI |
| 3.0618 | 2.20E-03 | GTExv8.EUR.Whole_Blood | 1.29E-02 | LFNG | BMI |
| 2.7931 | 5.22E-03 | GTExv8.EUR.Whole_Blood | 2.58E-02 | BRAT1 | BMI |
| -3.3863 | 7.08E-04 | GTExv8.EUR.Whole_Blood | 5.15E-03 | GNA12 | BMI |
| -3.2174 | 1.29E-03 | GTExv8.EUR.Whole_Blood | 8.51E-03 | SDK1 | BMI |
| 2.9998 | 2.70E-03 | GTExv8.EUR.Whole_Blood | 1.52E-02 | ZNF890P | BMI |
| -2.6826 | 7.31E-03 | GTExv8.EUR.Whole_Blood | 3.35E-02 | OCM | BMI |
| 5.4045 | 6.50E-08 | GTExv8.EUR.Whole_Blood | 1.53E-06 | PMS2 | BMI |
| 3.9299 | 8.50E-05 | GTExv8.EUR.Whole_Blood | 8.44E-04 | FAM220A | BMI |
| 4.8363 | 1.32E-06 | GTExv8.EUR.Whole_Blood | 2.17E-05 | RAC1 | BMI |
| -3.0816 | 2.06E-03 | GTExv8.EUR.Whole_Blood | 1.23E-02 | ZDHHC4 | BMI |
| -3.5665 | 3.62E-04 | GTExv8.EUR.Whole_Blood | 2.92E-03 | C7orf26 | BMI |
| -5.1961 | 2.03E-07 | GTExv8.EUR.Whole_Blood | 4.17E-06 | ZNF316 | BMI |
| -3.4315 | 6.00E-04 | GTExv8.EUR.Whole_Blood | 4.50E-03 | PMS2CL | BMI |
| -3.9949 | 6.47E-05 | GTExv8.EUR.Whole_Blood | 6.65E-04 | CCZ1B | BMI |
| 2.9748 | 2.93E-03 | GTExv8.EUR.Whole_Blood | 1.63E-02 | TMEM106B | BMI |
| -3.5877 | 3.34E-04 | GTExv8.EUR.Whole_Blood | 2.73E-03 | TWISTNB | BMI |
| -3.6687 | 2.44E-04 | GTExv8.EUR.Whole_Blood | 2.09E-03 | DFNA5 | BMI |
| 2.5529 | 1.07E-02 | GTExv8.EUR.Whole_Blood | 4.53E-02 | AC004540.5 | BMI |
| 3.8602 | 1.13E-04 | GTExv8.EUR.Whole_Blood | 1.09E-03 | HOTAIRM1 | BMI |
| 3.1141 | 1.84E-03 | GTExv8.EUR.Whole_Blood | 1.12E-02 | HOXA5 | BMI |
| -6.6402 | 3.13E-11 | GTExv8.EUR.Whole_Blood | 1.68E-09 | HOXA9 | BMI |
| -5.7366 | 9.66E-09 | GTExv8.EUR.Whole_Blood | 2.82E-07 | HOXA10 | BMI |
| -2.6204 | 8.78E-03 | GTExv8.EUR.Whole_Blood | 3.87E-02 | LINC01176 | BMI |
| 2.9478 | 3.20E-03 | GTExv8.EUR.Whole_Blood | 1.74E-02 | PPP1R17 | BMI |
| -5.0538 | 4.33E-07 | GTExv8.EUR.Whole_Blood | 8.02E-06 | KBTBD2 | BMI |
| 2.9390 | 3.29E-03 | GTExv8.EUR.Whole_Blood | 1.78E-02 | FKBP9 | BMI |
| -5.1241 | 2.99E-07 | GTExv8.EUR.Whole_Blood | 5.82E-06 | DPY19L1 | BMI |
| -2.7602 | 5.78E-03 | GTExv8.EUR.Whole_Blood | 2.79E-02 | LINC00265 | BMI |
| 2.8660 | 4.16E-03 | GTExv8.EUR.Whole_Blood | 2.16E-02 | INHBA | BMI |
| -2.6946 | 7.05E-03 | GTExv8.EUR.Whole_Blood | 3.27E-02 | C7orf25 | BMI |
| 4.8051 | 1.55E-06 | GTExv8.EUR.Whole_Blood | 2.51E-05 | NUDCD3 | BMI |
| -3.8164 | 1.35E-04 | GTExv8.EUR.Whole_Blood | 1.27E-03 | OGDH | BMI |
| -6.1368 | 8.42E-10 | GTExv8.EUR.Whole_Blood | 3.12E-08 | H2AFV | BMI |
| 3.0520 | 2.27E-03 | GTExv8.EUR.Whole_Blood | 1.32E-02 | RP4-647J21.1 | BMI |
| 2.8480 | 4.40E-03 | GTExv8.EUR.Whole_Blood | 2.27E-02 | CCM2 | BMI |
| -3.5536 | 3.80E-04 | GTExv8.EUR.Whole_Blood | 3.04E-03 | IKZF1 | BMI |
| -4.1996 | 2.67E-05 | GTExv8.EUR.Whole_Blood | 3.12E-04 | FIGNL1 | BMI |
| -3.7599 | 1.70E-04 | GTExv8.EUR.Whole_Blood | 1.54E-03 | GS1-124K5.2 | BMI |
| -2.5289 | 1.14E-02 | GTExv8.EUR.Whole_Blood | 4.75E-02 | GS1-124K5.12 | BMI |
| 4.5264 | 6.00E-06 | GTExv8.EUR.Whole_Blood | 8.44E-05 | GTF2IP23 | BMI |
| 4.0454 | 5.22E-05 | GTExv8.EUR.Whole_Blood | 5.54E-04 | RP11-458F8.4 | BMI |
| -2.9209 | 3.49E-03 | GTExv8.EUR.Whole_Blood | 1.87E-02 | TMEM248 | BMI |
| -4.2045 | 2.62E-05 | GTExv8.EUR.Whole_Blood | 3.06E-04 | AUTS2 | BMI |
| 3.1464 | 1.65E-03 | GTExv8.EUR.Whole_Blood | 1.03E-02 | POM121 | BMI |
| 6.6223 | 3.54E-11 | GTExv8.EUR.Whole_Blood | 1.86E-09 | GTF2I | BMI |
| 4.9830 | 6.26E-07 | GTExv8.EUR.Whole_Blood | 1.11E-05 | NCF1 | BMI |
| 6.9482 | 3.70E-12 | GTExv8.EUR.Whole_Blood | 2.25E-10 | GTF2IRD2 | BMI |
| 4.7855 | 1.71E-06 | GTExv8.EUR.Whole_Blood | 2.72E-05 | PMS2P5 | BMI |
| 10.2219 | 1.58E-24 | GTExv8.EUR.Whole_Blood | 2.67E-22 | RCC1L | BMI |
| 2.7874 | 5.31E-03 | GTExv8.EUR.Whole_Blood | 2.61E-02 | GTF2IP1 | BMI |
| 9.8534 | 6.63E-23 | GTExv8.EUR.Whole_Blood | 9.38E-21 | STAG3L1 | BMI |
| 11.8969 | 1.23E-32 | GTExv8.EUR.Whole_Blood | 3.83E-30 | NSUN5P1 | BMI |
| 9.2606 | 2.03E-20 | GTExv8.EUR.Whole_Blood | 2.63E-18 | POM121C | BMI |
| 12.4630 | 1.19E-35 | GTExv8.EUR.Whole_Blood | 4.41E-33 | SPDYE5 | BMI |
| -12.4777 | 9.88E-36 | GTExv8.EUR.Whole_Blood | 3.84E-33 | PMS2P3 | BMI |
| -4.3012 | 1.70E-05 | GTExv8.EUR.Whole_Blood | 2.10E-04 | HIP1 | BMI |
| -2.6754 | 7.47E-03 | GTExv8.EUR.Whole_Blood | 3.41E-02 | CCL24 | BMI |
| -4.0255 | 5.69E-05 | GTExv8.EUR.Whole_Blood | 5.98E-04 | TMEM120A | BMI |
| 3.9848 | 6.75E-05 | GTExv8.EUR.Whole_Blood | 6.87E-04 | STYXL1 | BMI |
| 2.6611 | 7.79E-03 | GTExv8.EUR.Whole_Blood | 3.52E-02 | DTX2 | BMI |
| 10.9776 | 4.90E-28 | GTExv8.EUR.Whole_Blood | 1.03E-25 | UPK3BP1 | BMI |
| 10.6783 | 1.29E-26 | GTExv8.EUR.Whole_Blood | 2.33E-24 | PMS2P11 | BMI |
| -5.0847 | 3.68E-07 | GTExv8.EUR.Whole_Blood | 7.00E-06 | PMS2P9 | BMI |
| -2.6652 | 7.69E-03 | GTExv8.EUR.Whole_Blood | 3.49E-02 | SPDYE18 | BMI |
| 3.0568 | 2.24E-03 | GTExv8.EUR.Whole_Blood | 1.31E-02 | GSAP | BMI |
| -5.6602 | 1.51E-08 | GTExv8.EUR.Whole_Blood | 4.21E-07 | TMEM60 | BMI |
| -4.7303 | 2.24E-06 | GTExv8.EUR.Whole_Blood | 3.49E-05 | MAGI2-AS3 | BMI |
| 2.9750 | 2.93E-03 | GTExv8.EUR.Whole_Blood | 1.63E-02 | SLC25A13 | BMI |
| -3.6797 | 2.33E-04 | GTExv8.EUR.Whole_Blood | 2.01E-03 | ARPC1B | BMI |
| 5.8258 | 5.69E-09 | GTExv8.EUR.Whole_Blood | 1.74E-07 | PDAP1 | BMI |
| 6.8735 | 6.27E-12 | GTExv8.EUR.Whole_Blood | 3.70E-10 | ZKSCAN5 | BMI |
| 7.0633 | 1.63E-12 | GTExv8.EUR.Whole_Blood | 1.12E-10 | ZNF655 | BMI |
| -3.5171 | 4.36E-04 | GTExv8.EUR.Whole_Blood | 3.42E-03 | CYP3A5 | BMI |
| -2.8135 | 4.90E-03 | GTExv8.EUR.Whole_Blood | 2.46E-02 | ZSCAN21 | BMI |
| -3.0799 | 2.07E-03 | GTExv8.EUR.Whole_Blood | 1.24E-02 | AP4M1 | BMI |
| 2.7049 | 6.83E-03 | GTExv8.EUR.Whole_Blood | 3.19E-02 | PLOD3 | BMI |
| 4.1517 | 3.30E-05 | GTExv8.EUR.Whole_Blood | 3.72E-04 | LHFPL3-AS2 | BMI |
| 3.9710 | 7.16E-05 | GTExv8.EUR.Whole_Blood | 7.21E-04 | RP11-325F22.2 | BMI |
| 2.8782 | 4.00E-03 | GTExv8.EUR.Whole_Blood | 2.09E-02 | SRPK2 | BMI |
| 3.8639 | 1.12E-04 | GTExv8.EUR.Whole_Blood | 1.08E-03 | IMMP2L | BMI |
| 4.0899 | 4.32E-05 | GTExv8.EUR.Whole_Blood | 4.70E-04 | MDFIC | BMI |
| -4.1072 | 4.01E-05 | GTExv8.EUR.Whole_Blood | 4.42E-04 | CAV2 | BMI |
| 3.2974 | 9.76E-04 | GTExv8.EUR.Whole_Blood | 6.75E-03 | LRRC4 | BMI |
| -2.5095 | 1.21E-02 | GTExv8.EUR.Whole_Blood | 4.97E-02 | RP11-212P7.2 | BMI |
| 4.4725 | 7.73E-06 | GTExv8.EUR.Whole_Blood | 1.06E-04 | CEP41 | BMI |
| -3.8061 | 1.41E-04 | GTExv8.EUR.Whole_Blood | 1.31E-03 | COPG2 | BMI |
| -3.0115 | 2.60E-03 | GTExv8.EUR.Whole_Blood | 1.47E-02 | TMEM140 | BMI |
| 3.0115 | 2.60E-03 | GTExv8.EUR.Whole_Blood | 1.47E-02 | C7orf49 | BMI |
| -2.9027 | 3.70E-03 | GTExv8.EUR.Whole_Blood | 1.96E-02 | KDM7A | BMI |
| 2.6606 | 7.80E-03 | GTExv8.EUR.Whole_Blood | 3.52E-02 | JHDM1D-AS1 | BMI |
| 2.7724 | 5.56E-03 | GTExv8.EUR.Whole_Blood | 2.71E-02 | TRBV3-1 | BMI |
| 4.1451 | 3.40E-05 | GTExv8.EUR.Whole_Blood | 3.80E-04 | TRBV12-5 | BMI |
| 4.3025 | 1.69E-05 | GTExv8.EUR.Whole_Blood | 2.09E-04 | TRBV19 | BMI |
| -3.1258 | 1.77E-03 | GTExv8.EUR.Whole_Blood | 1.09E-02 | TRBV24-1 | BMI |
| -4.0750 | 4.60E-05 | GTExv8.EUR.Whole_Blood | 4.95E-04 | TRBV28 | BMI |
| 3.7016 | 2.14E-04 | GTExv8.EUR.Whole_Blood | 1.87E-03 | TRBV30 | BMI |
| 2.8338 | 4.60E-03 | GTExv8.EUR.Whole_Blood | 2.34E-02 | TRPV5 | BMI |
| 3.6571 | 2.55E-04 | GTExv8.EUR.Whole_Blood | 2.17E-03 | C7orf34 | BMI |
| 3.3041 | 9.53E-04 | GTExv8.EUR.Whole_Blood | 6.65E-03 | ZNF767P | BMI |
| 2.5131 | 1.20E-02 | GTExv8.EUR.Whole_Blood | 4.94E-02 | KRBA1 | BMI |
| -3.4493 | 5.62E-04 | GTExv8.EUR.Whole_Blood | 4.26E-03 | LRRC61 | BMI |
| 3.4885 | 4.86E-04 | GTExv8.EUR.Whole_Blood | 3.74E-03 | ZBED6CL | BMI |
| -3.4642 | 5.32E-04 | GTExv8.EUR.Whole_Blood | 4.05E-03 | RP4-584D14.7 | BMI |
| 3.1498 | 1.63E-03 | GTExv8.EUR.Whole_Blood | 1.02E-02 | SLC4A2 | BMI |
| -4.6493 | 3.33E-06 | GTExv8.EUR.Whole_Blood | 5.01E-05 | AGAP3 | BMI |
| -2.5357 | 1.12E-02 | GTExv8.EUR.Whole_Blood | 4.68E-02 | LINC01003 | BMI |
| -3.0757 | 2.10E-03 | GTExv8.EUR.Whole_Blood | 1.25E-02 | UBE3C | BMI |
| -3.5091 | 4.50E-04 | GTExv8.EUR.Whole_Blood | 3.51E-03 | ESYT2 | BMI |
| -2.8943 | 3.80E-03 | GTExv8.EUR.Whole_Blood | 2.00E-02 | CTD-2336O2.3 | BMI |
| 3.3679 | 7.57E-04 | GTExv8.EUR.Whole_Blood | 5.46E-03 | RP11-115C21.2 | BMI |
| 8.7321 | 2.50E-18 | GTExv8.EUR.Whole_Blood | 2.78E-16 | ALG1L13P | BMI |
| 6.7704 | 1.28E-11 | GTExv8.EUR.Whole_Blood | 7.27E-10 | RPL10P19 | BMI |
| -3.7848 | 1.54E-04 | GTExv8.EUR.Whole_Blood | 1.42E-03 | ERI1 | BMI |
| 3.6733 | 2.39E-04 | GTExv8.EUR.Whole_Blood | 2.06E-03 | RP11-10A14.5 | BMI |
| 9.4077 | 5.07E-21 | GTExv8.EUR.Whole_Blood | 6.69E-19 | LINCR-0001 | BMI |
| 5.3172 | 1.05E-07 | GTExv8.EUR.Whole_Blood | 2.37E-06 | RP11-981G7.6 | BMI |
| 7.8790 | 3.30E-15 | GTExv8.EUR.Whole_Blood | 3.17E-13 | AF131215.9 | BMI |
| 7.8428 | 4.40E-15 | GTExv8.EUR.Whole_Blood | 4.08E-13 | AF131215.2 | BMI |
| -5.1993 | 2.00E-07 | GTExv8.EUR.Whole_Blood | 4.14E-06 | SLC35G5 | BMI |
| 6.1875 | 6.11E-10 | GTExv8.EUR.Whole_Blood | 2.35E-08 | FAM167A | BMI |
| -6.4255 | 1.31E-10 | GTExv8.EUR.Whole_Blood | 5.83E-09 | BLK | BMI |
| -5.9262 | 3.10E-09 | GTExv8.EUR.Whole_Blood | 1.03E-07 | RP11-148O21.4 | BMI |
| -4.4784 | 7.52E-06 | GTExv8.EUR.Whole_Blood | 1.04E-04 | RP11-148O21.2 | BMI |
| 4.7982 | 1.60E-06 | GTExv8.EUR.Whole_Blood | 2.57E-05 | NEIL2 | BMI |
| 7.1497 | 8.69E-13 | GTExv8.EUR.Whole_Blood | 6.38E-11 | FDFT1 | BMI |
| -5.7643 | 8.20E-09 | GTExv8.EUR.Whole_Blood | 2.45E-07 | RP11-297N6.4 | BMI |
| -3.1348 | 1.72E-03 | GTExv8.EUR.Whole_Blood | 1.06E-02 | MICU3 | BMI |
| -5.5161 | 3.47E-08 | GTExv8.EUR.Whole_Blood | 8.77E-07 | ZDHHC2 | BMI |
| -2.7059 | 6.81E-03 | GTExv8.EUR.Whole_Blood | 3.19E-02 | PCM1 | BMI |
| -3.6272 | 2.87E-04 | GTExv8.EUR.Whole_Blood | 2.41E-03 | INTS10 | BMI |
| -2.6983 | 6.97E-03 | GTExv8.EUR.Whole_Blood | 3.24E-02 | NUDT18 | BMI |
| 2.5973 | 9.40E-03 | GTExv8.EUR.Whole_Blood | 4.08E-02 | TNFRSF10A | BMI |
| -4.3502 | 1.36E-05 | GTExv8.EUR.Whole_Blood | 1.75E-04 | SLC25A37 | BMI |
| 4.9173 | 8.77E-07 | GTExv8.EUR.Whole_Blood | 1.51E-05 | TRIM35 | BMI |
| -3.6461 | 2.66E-04 | GTExv8.EUR.Whole_Blood | 2.25E-03 | PTK2B | BMI |
| 4.1291 | 3.64E-05 | GTExv8.EUR.Whole_Blood | 4.06E-04 | ELP3 | BMI |
| -4.7616 | 1.92E-06 | GTExv8.EUR.Whole_Blood | 3.02E-05 | RP11-11N9.4 | BMI |
| -5.6003 | 2.14E-08 | GTExv8.EUR.Whole_Blood | 5.78E-07 | RP11-722E23.2 | BMI |
| -5.8647 | 4.50E-09 | GTExv8.EUR.Whole_Blood | 1.42E-07 | FUT10 | BMI |
| 5.8843 | 4.00E-09 | GTExv8.EUR.Whole_Blood | 1.29E-07 | TTI2 | BMI |
| 2.6197 | 8.80E-03 | GTExv8.EUR.Whole_Blood | 3.87E-02 | PROSC | BMI |
| 4.4233 | 9.72E-06 | GTExv8.EUR.Whole_Blood | 1.31E-04 | RP11-350N15.4 | BMI |
| 4.8331 | 1.34E-06 | GTExv8.EUR.Whole_Blood | 2.20E-05 | FGFR1 | BMI |
| 2.9904 | 2.79E-03 | GTExv8.EUR.Whole_Blood | 1.56E-02 | ADAM9 | BMI |
| -2.5981 | 9.38E-03 | GTExv8.EUR.Whole_Blood | 4.08E-02 | AP3M2 | BMI |
| 2.5848 | 9.74E-03 | GTExv8.EUR.Whole_Blood | 4.21E-02 | PCMTD1 | BMI |
| 2.9052 | 3.67E-03 | GTExv8.EUR.Whole_Blood | 1.95E-02 | NSMAF | BMI |
| -3.2905 | 1.00E-03 | GTExv8.EUR.Whole_Blood | 6.86E-03 | RAB2A | BMI |
| 2.6875 | 7.20E-03 | GTExv8.EUR.Whole_Blood | 3.31E-02 | CHD7 | BMI |
| 2.9404 | 3.28E-03 | GTExv8.EUR.Whole_Blood | 1.78E-02 | ASPH | BMI |
| -2.9455 | 3.22E-03 | GTExv8.EUR.Whole_Blood | 1.75E-02 | ADHFE1 | BMI |
| 4.0193 | 5.84E-05 | GTExv8.EUR.Whole_Blood | 6.10E-04 | C8orf46 | BMI |
| -3.5695 | 3.58E-04 | GTExv8.EUR.Whole_Blood | 2.89E-03 | MYBL1 | BMI |
| 3.3824 | 7.18E-04 | GTExv8.EUR.Whole_Blood | 5.21E-03 | LACTB2 | BMI |
| -3.0666 | 2.17E-03 | GTExv8.EUR.Whole_Blood | 1.28E-02 | CA3-AS1 | BMI |
| 5.0258 | 5.01E-07 | GTExv8.EUR.Whole_Blood | 9.00E-06 | RMDN1 | BMI |
| -3.0199 | 2.53E-03 | GTExv8.EUR.Whole_Blood | 1.44E-02 | RP11-267M23.1 | BMI |
| -5.9485 | 2.71E-09 | GTExv8.EUR.Whole_Blood | 9.09E-08 | YWHAZ | BMI |
| 2.7408 | 6.13E-03 | GTExv8.EUR.Whole_Blood | 2.93E-02 | DCAF13 | BMI |
| 2.5449 | 1.09E-02 | GTExv8.EUR.Whole_Blood | 4.58E-02 | UTP23 | BMI |
| 5.4865 | 4.10E-08 | GTExv8.EUR.Whole_Blood | 1.02E-06 | EXT1 | BMI |
| 3.2670 | 1.09E-03 | GTExv8.EUR.Whole_Blood | 7.41E-03 | TAF2 | BMI |
| 2.6521 | 8.00E-03 | GTExv8.EUR.Whole_Blood | 3.59E-02 | FAM84B | BMI |
| 3.1947 | 1.40E-03 | GTExv8.EUR.Whole_Blood | 9.03E-03 | SLC45A4 | BMI |
| 3.4890 | 4.85E-04 | GTExv8.EUR.Whole_Blood | 3.73E-03 | LINC01300 | BMI |
| 2.6694 | 7.60E-03 | GTExv8.EUR.Whole_Blood | 3.45E-02 | GLI4 | BMI |
| -3.1795 | 1.48E-03 | GTExv8.EUR.Whole_Blood | 9.46E-03 | MINCR | BMI |
| 3.9710 | 7.16E-05 | GTExv8.EUR.Whole_Blood | 7.21E-04 | ZNF696 | BMI |
| -2.5347 | 1.13E-02 | GTExv8.EUR.Whole_Blood | 4.71E-02 | MAF1 | BMI |
| -2.9941 | 2.75E-03 | GTExv8.EUR.Whole_Blood | 1.54E-02 | WDR97 | BMI |
| 4.1478 | 3.36E-05 | GTExv8.EUR.Whole_Blood | 3.77E-04 | SHARPIN | BMI |
| -2.5758 | 1.00E-02 | GTExv8.EUR.Whole_Blood | 4.29E-02 | TONSL | BMI |
| 4.2517 | 2.12E-05 | GTExv8.EUR.Whole_Blood | 2.55E-04 | RP11-408A13.4 | BMI |
| -3.4576 | 5.45E-04 | GTExv8.EUR.Whole_Blood | 4.14E-03 | BAG1 | BMI |
| -4.0281 | 5.62E-05 | GTExv8.EUR.Whole_Blood | 5.93E-04 | UBAP1 | BMI |
| -4.4546 | 8.41E-06 | GTExv8.EUR.Whole_Blood | 1.14E-04 | RPP25L | BMI |
| 2.6650 | 7.70E-03 | GTExv8.EUR.Whole_Blood | 3.49E-02 | GALT | BMI |
| -3.2107 | 1.32E-03 | GTExv8.EUR.Whole_Blood | 8.64E-03 | IL11RA | BMI |
| 2.8932 | 3.81E-03 | GTExv8.EUR.Whole_Blood | 2.00E-02 | RP11-195F19.9 | BMI |
| -3.9903 | 6.60E-05 | GTExv8.EUR.Whole_Blood | 6.75E-04 | TPM2 | BMI |
| 3.1850 | 1.45E-03 | GTExv8.EUR.Whole_Blood | 9.32E-03 | CREB3 | BMI |
| -4.3287 | 1.50E-05 | GTExv8.EUR.Whole_Blood | 1.90E-04 | GBA2 | BMI |
| 4.3287 | 1.50E-05 | GTExv8.EUR.Whole_Blood | 1.90E-04 | RGP1 | BMI |
| 2.8704 | 4.10E-03 | GTExv8.EUR.Whole_Blood | 2.13E-02 | SPAG8 | BMI |
| 2.8151 | 4.88E-03 | GTExv8.EUR.Whole_Blood | 2.45E-02 | SLC25A51 | BMI |
| -2.5729 | 1.01E-02 | GTExv8.EUR.Whole_Blood | 4.32E-02 | OSTF1 | BMI |
| -2.8665 | 4.15E-03 | GTExv8.EUR.Whole_Blood | 2.16E-02 | GCNT1 | BMI |
| -3.8085 | 1.40E-04 | GTExv8.EUR.Whole_Blood | 1.31E-03 | PRUNE2 | BMI |
| -2.7702 | 5.60E-03 | GTExv8.EUR.Whole_Blood | 2.72E-02 | CEP78 | BMI |
| 2.8408 | 4.50E-03 | GTExv8.EUR.Whole_Blood | 2.31E-02 | GKAP1 | BMI |
| 3.3908 | 6.97E-04 | GTExv8.EUR.Whole_Blood | 5.08E-03 | ISCA1 | BMI |
| -4.2135 | 2.51E-05 | GTExv8.EUR.Whole_Blood | 2.96E-04 | SPIN1 | BMI |
| 3.8426 | 1.22E-04 | GTExv8.EUR.Whole_Blood | 1.16E-03 | NXNL2 | BMI |
| 3.2142 | 1.31E-03 | GTExv8.EUR.Whole_Blood | 8.60E-03 | SECISBP2 | BMI |
| -4.1018 | 4.10E-05 | GTExv8.EUR.Whole_Blood | 4.51E-04 | RP13-93L13.1 | BMI |
| -5.5896 | 2.28E-08 | GTExv8.EUR.Whole_Blood | 6.08E-07 | SEMA4D | BMI |
| -2.8088 | 4.97E-03 | GTExv8.EUR.Whole_Blood | 2.48E-02 | AUH | BMI |
| -3.2063 | 1.34E-03 | GTExv8.EUR.Whole_Blood | 8.75E-03 | NFIL3 | BMI |
| 3.3339 | 8.56E-04 | GTExv8.EUR.Whole_Blood | 6.09E-03 | CENPP | BMI |
| -3.8249 | 1.31E-04 | GTExv8.EUR.Whole_Blood | 1.24E-03 | RP11-165J3.6 | BMI |
| 5.9899 | 2.10E-09 | GTExv8.EUR.Whole_Blood | 7.17E-08 | PHF2 | BMI |
| -3.9104 | 9.21E-05 | GTExv8.EUR.Whole_Blood | 9.06E-04 | MFSD14B | BMI |
| -3.2389 | 1.20E-03 | GTExv8.EUR.Whole_Blood | 8.00E-03 | ERCC6L2 | BMI |
| -3.2372 | 1.21E-03 | GTExv8.EUR.Whole_Blood | 8.05E-03 | RP11-535M15.2 | BMI |
| 5.9244 | 3.13E-09 | GTExv8.EUR.Whole_Blood | 1.03E-07 | ANKS6 | BMI |
| 3.9302 | 8.49E-05 | GTExv8.EUR.Whole_Blood | 8.44E-04 | STX17 | BMI |
| 7.8989 | 2.81E-15 | GTExv8.EUR.Whole_Blood | 2.77E-13 | INVS | BMI |
| -5.1437 | 2.69E-07 | GTExv8.EUR.Whole_Blood | 5.30E-06 | TEX10 | BMI |
| 3.6896 | 2.25E-04 | GTExv8.EUR.Whole_Blood | 1.96E-03 | MSANTD3 | BMI |
| -3.7497 | 1.77E-04 | GTExv8.EUR.Whole_Blood | 1.59E-03 | TMEM245 | BMI |
| 2.5195 | 1.18E-02 | GTExv8.EUR.Whole_Blood | 4.89E-02 | C9orf152 | BMI |
| 3.1319 | 1.74E-03 | GTExv8.EUR.Whole_Blood | 1.08E-02 | RP11-4O1.2 | BMI |
| 3.0757 | 2.10E-03 | GTExv8.EUR.Whole_Blood | 1.25E-02 | SUSD1 | BMI |
| -2.5643 | 1.03E-02 | GTExv8.EUR.Whole_Blood | 4.39E-02 | CDC26 | BMI |
| 5.1051 | 3.31E-07 | GTExv8.EUR.Whole_Blood | 6.34E-06 | PRPF4 | BMI |
| -4.7980 | 1.60E-06 | GTExv8.EUR.Whole_Blood | 2.57E-05 | TLR4 | BMI |
| -4.9080 | 9.20E-07 | GTExv8.EUR.Whole_Blood | 1.57E-05 | RP11-281A20.2 | BMI |
| 3.0552 | 2.25E-03 | GTExv8.EUR.Whole_Blood | 1.31E-02 | C5 | BMI |
| 2.7478 | 6.00E-03 | GTExv8.EUR.Whole_Blood | 2.88E-02 | RABGAP1 | BMI |
| 4.0509 | 5.10E-05 | GTExv8.EUR.Whole_Blood | 5.42E-04 | MIR181A2HG | BMI |
| -2.5362 | 1.12E-02 | GTExv8.EUR.Whole_Blood | 4.68E-02 | RABEPK | BMI |
| -3.7190 | 2.00E-04 | GTExv8.EUR.Whole_Blood | 1.76E-03 | GAPVD1 | BMI |
| 4.1134 | 3.90E-05 | GTExv8.EUR.Whole_Blood | 4.32E-04 | ZBTB34 | BMI |
| 4.0167 | 5.90E-05 | GTExv8.EUR.Whole_Blood | 6.15E-04 | SLC2A8 | BMI |
| 3.1599 | 1.58E-03 | GTExv8.EUR.Whole_Blood | 9.96E-03 | ZNF79 | BMI |
| -3.0966 | 1.96E-03 | GTExv8.EUR.Whole_Blood | 1.18E-02 | RPL12 | BMI |
| -2.5765 | 9.98E-03 | GTExv8.EUR.Whole_Blood | 4.29E-02 | ENG | BMI |
| -6.8085 | 9.86E-12 | GTExv8.EUR.Whole_Blood | 5.64E-10 | URM1 | BMI |
| 6.5167 | 7.19E-11 | GTExv8.EUR.Whole_Blood | 3.52E-09 | ODF2 | BMI |
| -3.8996 | 9.63E-05 | GTExv8.EUR.Whole_Blood | 9.41E-04 | GLE1 | BMI |
| 3.2007 | 1.37E-03 | GTExv8.EUR.Whole_Blood | 8.92E-03 | ENDOG | BMI |
| 2.6315 | 8.50E-03 | GTExv8.EUR.Whole_Blood | 3.77E-02 | SPOUT1 | BMI |
| 3.5069 | 4.53E-04 | GTExv8.EUR.Whole_Blood | 3.53E-03 | SH3GLB2 | BMI |
| 2.5757 | 1.00E-02 | GTExv8.EUR.Whole_Blood | 4.29E-02 | TOR1B | BMI |
| 2.6589 | 7.84E-03 | GTExv8.EUR.Whole_Blood | 3.54E-02 | TOR1A | BMI |
| 2.8896 | 3.86E-03 | GTExv8.EUR.Whole_Blood | 2.02E-02 | C9orf78 | BMI |
| 2.8121 | 4.92E-03 | GTExv8.EUR.Whole_Blood | 2.46E-02 | PRRC2B | BMI |
| -2.6968 | 7.00E-03 | GTExv8.EUR.Whole_Blood | 3.25E-02 | POMT1 | BMI |
| -2.8338 | 4.60E-03 | GTExv8.EUR.Whole_Blood | 2.34E-02 | UCK1 | BMI |
| 5.1582 | 2.49E-07 | GTExv8.EUR.Whole_Blood | 4.97E-06 | MED27 | BMI |
| 3.7995 | 1.45E-04 | GTExv8.EUR.Whole_Blood | 1.34E-03 | VAV2 | BMI |
| 3.2134 | 1.31E-03 | GTExv8.EUR.Whole_Blood | 8.60E-03 | DIP2C | BMI |
| -2.9373 | 3.31E-03 | GTExv8.EUR.Whole_Blood | 1.79E-02 | ADARB2 | BMI |
| -4.9162 | 8.82E-07 | GTExv8.EUR.Whole_Blood | 1.51E-05 | RP11-295P9.3 | BMI |
| -3.9863 | 6.71E-05 | GTExv8.EUR.Whole_Blood | 6.84E-04 | NMT2 | BMI |
| 3.0357 | 2.40E-03 | GTExv8.EUR.Whole_Blood | 1.38E-02 | TRDMT1 | BMI |
| -3.3277 | 8.76E-04 | GTExv8.EUR.Whole_Blood | 6.20E-03 | CACNB2 | BMI |
| 3.9939 | 6.50E-05 | GTExv8.EUR.Whole_Blood | 6.67E-04 | RP11-354E11.2 | BMI |
| 2.6785 | 7.39E-03 | GTExv8.EUR.Whole_Blood | 3.38E-02 | MLLT10 | BMI |
| -5.0889 | 3.60E-07 | GTExv8.EUR.Whole_Blood | 6.87E-06 | PIP4K2A | BMI |
| 2.5952 | 9.45E-03 | GTExv8.EUR.Whole_Blood | 4.10E-02 | APBB1IP | BMI |
| 2.9296 | 3.39E-03 | GTExv8.EUR.Whole_Blood | 1.82E-02 | LINC00202-2 | BMI |
| 4.8644 | 1.15E-06 | GTExv8.EUR.Whole_Blood | 1.91E-05 | LINC00202-1 | BMI |
| -4.7884 | 1.68E-06 | GTExv8.EUR.Whole_Blood | 2.69E-05 | YME1L1 | BMI |
| -4.0768 | 4.57E-05 | GTExv8.EUR.Whole_Blood | 4.93E-04 | MASTL | BMI |
| 4.0236 | 5.73E-05 | GTExv8.EUR.Whole_Blood | 6.01E-04 | ARHGAP12 | BMI |
| -2.9149 | 3.56E-03 | GTExv8.EUR.Whole_Blood | 1.90E-02 | RP11-479G22.8 | BMI |
| -4.6697 | 3.02E-06 | GTExv8.EUR.Whole_Blood | 4.61E-05 | RP11-342D11.3 | BMI |
| -2.5746 | 1.00E-02 | GTExv8.EUR.Whole_Blood | 4.29E-02 | ZNF248 | BMI |
| -4.1892 | 2.80E-05 | GTExv8.EUR.Whole_Blood | 3.25E-04 | SGMS1-AS1 | BMI |
| -6.0242 | 1.70E-09 | GTExv8.EUR.Whole_Blood | 5.88E-08 | REEP3 | BMI |
| -3.2446 | 1.18E-03 | GTExv8.EUR.Whole_Blood | 7.91E-03 | SIRT1 | BMI |
| 3.1218 | 1.80E-03 | GTExv8.EUR.Whole_Blood | 1.10E-02 | PSAP | BMI |
| 4.2540 | 2.10E-05 | GTExv8.EUR.Whole_Blood | 2.53E-04 | BMS1P4 | BMI |
| 4.3163 | 1.59E-05 | GTExv8.EUR.Whole_Blood | 2.00E-04 | FUT11 | BMI |
| -4.9389 | 7.85E-07 | GTExv8.EUR.Whole_Blood | 1.36E-05 | VCL | BMI |
| 2.8260 | 4.71E-03 | GTExv8.EUR.Whole_Blood | 2.39E-02 | AP3M1 | BMI |
| 3.2935 | 9.89E-04 | GTExv8.EUR.Whole_Blood | 6.82E-03 | ADK | BMI |
| -4.5289 | 5.93E-06 | GTExv8.EUR.Whole_Blood | 8.36E-05 | VDAC2 | BMI |
| 3.6975 | 2.18E-04 | GTExv8.EUR.Whole_Blood | 1.90E-03 | PPIF | BMI |
| 3.2529 | 1.14E-03 | GTExv8.EUR.Whole_Blood | 7.67E-03 | NUTM2B-AS1 | BMI |
| 2.7876 | 5.31E-03 | GTExv8.EUR.Whole_Blood | 2.61E-02 | PLAC9 | BMI |
| 2.6655 | 7.69E-03 | GTExv8.EUR.Whole_Blood | 3.49E-02 | CDHR1 | BMI |
| 5.0762 | 3.85E-07 | GTExv8.EUR.Whole_Blood | 7.25E-06 | WAPL | BMI |
| -2.7065 | 6.80E-03 | GTExv8.EUR.Whole_Blood | 3.18E-02 | GLUD1 | BMI |
| -3.7456 | 1.80E-04 | GTExv8.EUR.Whole_Blood | 1.61E-03 | PCGF5 | BMI |
| 2.7912 | 5.25E-03 | GTExv8.EUR.Whole_Blood | 2.59E-02 | TNKS2-AS1 | BMI |
| 3.2987 | 9.71E-04 | GTExv8.EUR.Whole_Blood | 6.73E-03 | EIF2S2P3 | BMI |
| 2.6828 | 7.30E-03 | GTExv8.EUR.Whole_Blood | 3.34E-02 | NOC3L | BMI |
| -2.7465 | 6.02E-03 | GTExv8.EUR.Whole_Blood | 2.89E-02 | TCTN3 | BMI |
| -3.5416 | 3.98E-04 | GTExv8.EUR.Whole_Blood | 3.18E-03 | CC2D2B | BMI |
| 4.6296 | 3.66E-06 | GTExv8.EUR.Whole_Blood | 5.45E-05 | SLIT1 | BMI |
| -3.4107 | 6.48E-04 | GTExv8.EUR.Whole_Blood | 4.80E-03 | ARHGAP19 | BMI |
| -3.1272 | 1.76E-03 | GTExv8.EUR.Whole_Blood | 1.09E-02 | FRAT2 | BMI |
| -6.5616 | 5.32E-11 | GTExv8.EUR.Whole_Blood | 2.67E-09 | R3HCC1L | BMI |
| -7.3629 | 1.80E-13 | GTExv8.EUR.Whole_Blood | 1.51E-11 | LOXL4 | BMI |
| 3.2173 | 1.29E-03 | GTExv8.EUR.Whole_Blood | 8.51E-03 | SEC31B | BMI |
| 3.6015 | 3.16E-04 | GTExv8.EUR.Whole_Blood | 2.60E-03 | HIF1AN | BMI |
| 3.6410 | 2.72E-04 | GTExv8.EUR.Whole_Blood | 2.30E-03 | NDUFB8 | BMI |
| 3.1252 | 1.78E-03 | GTExv8.EUR.Whole_Blood | 1.09E-02 | LDB1 | BMI |
| -2.8039 | 5.05E-03 | GTExv8.EUR.Whole_Blood | 2.51E-02 | RPARP-AS1 | BMI |
| 4.5299 | 5.90E-06 | GTExv8.EUR.Whole_Blood | 8.33E-05 | MFSD13A | BMI |
| -2.8782 | 4.00E-03 | GTExv8.EUR.Whole_Blood | 2.09E-02 | SFXN2 | BMI |
| -7.2047 | 5.82E-13 | GTExv8.EUR.Whole_Blood | 4.52E-11 | AS3MT | BMI |
| 8.9123 | 5.00E-19 | GTExv8.EUR.Whole_Blood | 5.72E-17 | MARCKSL1P1 | BMI |
| 4.9529 | 7.31E-07 | GTExv8.EUR.Whole_Blood | 1.27E-05 | NT5C2 | BMI |
| 2.9055 | 3.67E-03 | GTExv8.EUR.Whole_Blood | 1.95E-02 | CALHM2 | BMI |
| -3.1984 | 1.38E-03 | GTExv8.EUR.Whole_Blood | 8.96E-03 | SH3PXD2A | BMI |
| -3.0086 | 2.62E-03 | GTExv8.EUR.Whole_Blood | 1.48E-02 | GPAM | BMI |
| -4.2240 | 2.40E-05 | GTExv8.EUR.Whole_Blood | 2.85E-04 | ACSL5 | BMI |
| -3.3129 | 9.23E-04 | GTExv8.EUR.Whole_Blood | 6.48E-03 | RP11-324O2.3 | BMI |
| -2.9591 | 3.09E-03 | GTExv8.EUR.Whole_Blood | 1.70E-02 | ZDHHC6 | BMI |
| -4.3032 | 1.68E-05 | GTExv8.EUR.Whole_Blood | 2.09E-04 | CASP7 | BMI |
| -7.0033 | 2.50E-12 | GTExv8.EUR.Whole_Blood | 1.59E-10 | RP11-539I5.1 | BMI |
| 7.1450 | 9.00E-13 | GTExv8.EUR.Whole_Blood | 6.55E-11 | SHTN1 | BMI |
| 2.6829 | 7.30E-03 | GTExv8.EUR.Whole_Blood | 3.34E-02 | RP11-129M16.4 | BMI |
| -3.9156 | 9.02E-05 | GTExv8.EUR.Whole_Blood | 8.92E-04 | METTL10 | BMI |
| 2.7942 | 5.20E-03 | GTExv8.EUR.Whole_Blood | 2.57E-02 | MGMT | BMI |
| 5.3519 | 8.71E-08 | GTExv8.EUR.Whole_Blood | 1.99E-06 | DPYSL4 | BMI |
| -3.0767 | 2.09E-03 | GTExv8.EUR.Whole_Blood | 1.25E-02 | STK32C | BMI |
| -2.7048 | 6.83E-03 | GTExv8.EUR.Whole_Blood | 3.19E-02 | LRRC27 | BMI |
| 2.6457 | 8.15E-03 | GTExv8.EUR.Whole_Blood | 3.65E-02 | ECHS1 | BMI |
| -3.1128 | 1.85E-03 | GTExv8.EUR.Whole_Blood | 1.12E-02 | RIC8A | BMI |
| -4.1114 | 3.93E-05 | GTExv8.EUR.Whole_Blood | 4.34E-04 | PSMD13 | BMI |
| -4.0510 | 5.10E-05 | GTExv8.EUR.Whole_Blood | 5.42E-04 | RP11-326C3.16 | BMI |
| -2.7156 | 6.62E-03 | GTExv8.EUR.Whole_Blood | 3.12E-02 | RP11-326C3.12 | BMI |
| -2.5356 | 1.12E-02 | GTExv8.EUR.Whole_Blood | 4.68E-02 | SIGIRR | BMI |
| 3.8468 | 1.20E-04 | GTExv8.EUR.Whole_Blood | 1.15E-03 | PTDSS2 | BMI |
| 3.0939 | 1.98E-03 | GTExv8.EUR.Whole_Blood | 1.19E-02 | RNH1 | BMI |
| 4.5114 | 6.44E-06 | GTExv8.EUR.Whole_Blood | 9.05E-05 | HRAS | BMI |
| 2.9152 | 3.56E-03 | GTExv8.EUR.Whole_Blood | 1.90E-02 | LMNTD2 | BMI |
| 4.8448 | 1.27E-06 | GTExv8.EUR.Whole_Blood | 2.10E-05 | EPS8L2 | BMI |
| 5.9381 | 2.88E-09 | GTExv8.EUR.Whole_Blood | 9.58E-08 | AP006621.5 | BMI |
| 5.8570 | 4.71E-09 | GTExv8.EUR.Whole_Blood | 1.47E-07 | CMB9-55F22.1 | BMI |
| 5.9749 | 2.30E-09 | GTExv8.EUR.Whole_Blood | 7.82E-08 | AP006621.6 | BMI |
| 6.6285 | 3.39E-11 | GTExv8.EUR.Whole_Blood | 1.81E-09 | PIDD1 | BMI |
| -6.6245 | 3.48E-11 | GTExv8.EUR.Whole_Blood | 1.84E-09 | PNPLA2 | BMI |
| 3.8274 | 1.30E-04 | GTExv8.EUR.Whole_Blood | 1.23E-03 | CRACR2B | BMI |
| 6.0012 | 1.96E-09 | GTExv8.EUR.Whole_Blood | 6.72E-08 | CD151 | BMI |
| -6.6430 | 3.07E-11 | GTExv8.EUR.Whole_Blood | 1.66E-09 | POLR2L | BMI |
| -4.0973 | 4.18E-05 | GTExv8.EUR.Whole_Blood | 4.58E-04 | TSPAN4 | BMI |
| -2.7892 | 5.28E-03 | GTExv8.EUR.Whole_Blood | 2.60E-02 | TRIM68 | BMI |
| -2.6065 | 9.15E-03 | GTExv8.EUR.Whole_Blood | 4.00E-02 | RIC3 | BMI |
| -6.2596 | 3.86E-10 | GTExv8.EUR.Whole_Blood | 1.52E-08 | STK33 | BMI |
| 3.5206 | 4.31E-04 | GTExv8.EUR.Whole_Blood | 3.39E-03 | TRIM66 | BMI |
| 4.3488 | 1.37E-05 | GTExv8.EUR.Whole_Blood | 1.76E-04 | RPL27A | BMI |
| 3.0229 | 2.50E-03 | GTExv8.EUR.Whole_Blood | 1.43E-02 | AMPD3 | BMI |
| 3.7359 | 1.87E-04 | GTExv8.EUR.Whole_Blood | 1.66E-03 | MRVI1-AS1 | BMI |
| -7.8835 | 3.18E-15 | GTExv8.EUR.Whole_Blood | 3.09E-13 | ARNTL | BMI |
| -2.8129 | 4.91E-03 | GTExv8.EUR.Whole_Blood | 2.46E-02 | INSC | BMI |
| 5.8100 | 6.25E-09 | GTExv8.EUR.Whole_Blood | 1.89E-07 | GTF2H1 | BMI |
| 2.6547 | 7.94E-03 | GTExv8.EUR.Whole_Blood | 3.58E-02 | ANO5 | BMI |
| -5.3640 | 8.14E-08 | GTExv8.EUR.Whole_Blood | 1.87E-06 | RCN1 | BMI |
| -2.8247 | 4.73E-03 | GTExv8.EUR.Whole_Blood | 2.39E-02 | APIP | BMI |
| -4.3145 | 1.60E-05 | GTExv8.EUR.Whole_Blood | 2.00E-04 | RP11-63D14.1 | BMI |
| 3.7599 | 1.70E-04 | GTExv8.EUR.Whole_Blood | 1.54E-03 | API5 | BMI |
| -10.9446 | 7.05E-28 | GTExv8.EUR.Whole_Blood | 1.37E-25 | HSD17B12 | BMI |
| -10.9578 | 6.10E-28 | GTExv8.EUR.Whole_Blood | 1.25E-25 | RP11-613D13.10 | BMI |
| 7.8709 | 3.52E-15 | GTExv8.EUR.Whole_Blood | 3.34E-13 | ALKBH3 | BMI |
| -5.0468 | 4.49E-07 | GTExv8.EUR.Whole_Blood | 8.28E-06 | CHST1 | BMI |
| 5.5086 | 3.62E-08 | GTExv8.EUR.Whole_Blood | 9.09E-07 | DGKZ | BMI |
| -5.4645 | 4.64E-08 | GTExv8.EUR.Whole_Blood | 1.14E-06 | ATG13 | BMI |
| -3.7896 | 1.51E-04 | GTExv8.EUR.Whole_Blood | 1.39E-03 | ARHGAP1 | BMI |
| 5.8406 | 5.20E-09 | GTExv8.EUR.Whole_Blood | 1.62E-07 | NR1H3 | BMI |
| 6.2492 | 4.13E-10 | GTExv8.EUR.Whole_Blood | 1.62E-08 | ACP2 | BMI |
| -11.2475 | 2.38E-29 | GTExv8.EUR.Whole_Blood | 5.45E-27 | MYBPC3 | BMI |
| 4.6631 | 3.12E-06 | GTExv8.EUR.Whole_Blood | 4.73E-05 | SLC39A13 | BMI |
| 5.7995 | 6.65E-09 | GTExv8.EUR.Whole_Blood | 2.01E-07 | CELF1 | BMI |
| -14.1982 | 9.40E-46 | GTExv8.EUR.Whole_Blood | 5.63E-43 | C1QTNF4 | BMI |
| 13.5635 | 6.59E-42 | GTExv8.EUR.Whole_Blood | 3.42E-39 | FNBP4 | BMI |
| 3.1112 | 1.86E-03 | GTExv8.EUR.Whole_Blood | 1.13E-02 | SERPING1 | BMI |
| 2.9889 | 2.80E-03 | GTExv8.EUR.Whole_Blood | 1.56E-02 | TMX2 | BMI |
| -3.8461 | 1.20E-04 | GTExv8.EUR.Whole_Blood | 1.15E-03 | FADS3 | BMI |
| -3.9797 | 6.90E-05 | GTExv8.EUR.Whole_Blood | 6.99E-04 | RAB3IL1 | BMI |
| 3.0407 | 2.36E-03 | GTExv8.EUR.Whole_Blood | 1.37E-02 | BEST1 | BMI |
| -2.9067 | 3.65E-03 | GTExv8.EUR.Whole_Blood | 1.94E-02 | EEF1G | BMI |
| -2.9096 | 3.62E-03 | GTExv8.EUR.Whole_Blood | 1.93E-02 | EML3 | BMI |
| -2.7762 | 5.50E-03 | GTExv8.EUR.Whole_Blood | 2.69E-02 | GANAB | BMI |
| 5.1106 | 3.21E-07 | GTExv8.EUR.Whole_Blood | 6.18E-06 | PLA2G16 | BMI |
| -3.2040 | 1.36E-03 | GTExv8.EUR.Whole_Blood | 8.87E-03 | ATL3 | BMI |
| 2.6428 | 8.22E-03 | GTExv8.EUR.Whole_Blood | 3.67E-02 | RTN3 | BMI |
| -4.0410 | 5.32E-05 | GTExv8.EUR.Whole_Blood | 5.63E-04 | TRPT1 | BMI |
| 5.2609 | 1.43E-07 | GTExv8.EUR.Whole_Blood | 3.08E-06 | PLCB3 | BMI |
| -4.7747 | 1.80E-06 | GTExv8.EUR.Whole_Blood | 2.85E-05 | GPR137 | BMI |
| -7.0320 | 2.04E-12 | GTExv8.EUR.Whole_Blood | 1.36E-10 | PRDX5 | BMI |
| 4.5550 | 5.24E-06 | GTExv8.EUR.Whole_Blood | 7.50E-05 | AP003774.1 | BMI |
| -3.3546 | 7.95E-04 | GTExv8.EUR.Whole_Blood | 5.70E-03 | CCDC88B | BMI |
| 2.9781 | 2.90E-03 | GTExv8.EUR.Whole_Blood | 1.61E-02 | RPS6KA4 | BMI |
| 3.1977 | 1.39E-03 | GTExv8.EUR.Whole_Blood | 9.01E-03 | AP003774.6 | BMI |
| -2.7095 | 6.74E-03 | GTExv8.EUR.Whole_Blood | 3.16E-02 | MEN1 | BMI |
| -3.2941 | 9.87E-04 | GTExv8.EUR.Whole_Blood | 6.81E-03 | SNX15 | BMI |
| 2.7214 | 6.50E-03 | GTExv8.EUR.Whole_Blood | 3.08E-02 | POLA2 | BMI |
| 2.9941 | 2.75E-03 | GTExv8.EUR.Whole_Blood | 1.54E-02 | CMB9-22P13.1 | BMI |
| 5.5767 | 2.45E-08 | GTExv8.EUR.Whole_Blood | 6.49E-07 | LTBP3 | BMI |
| 4.4533 | 8.45E-06 | GTExv8.EUR.Whole_Blood | 1.15E-04 | SSSCA1-AS1 | BMI |
| -5.7543 | 8.70E-09 | GTExv8.EUR.Whole_Blood | 2.57E-07 | EHBP1L1 | BMI |
| 6.9152 | 4.67E-12 | GTExv8.EUR.Whole_Blood | 2.80E-10 | SNX32 | BMI |
| -4.7036 | 2.56E-06 | GTExv8.EUR.Whole_Blood | 3.94E-05 | MUS81 | BMI |
| -3.9763 | 7.00E-05 | GTExv8.EUR.Whole_Blood | 7.08E-04 | CFL1 | BMI |
| -7.8629 | 3.75E-15 | GTExv8.EUR.Whole_Blood | 3.52E-13 | CTSW | BMI |
| 6.9674 | 3.23E-12 | GTExv8.EUR.Whole_Blood | 1.99E-10 | FIBP | BMI |
| -3.7550 | 1.73E-04 | GTExv8.EUR.Whole_Blood | 1.56E-03 | PACS1 | BMI |
| -3.2117 | 1.32E-03 | GTExv8.EUR.Whole_Blood | 8.64E-03 | KLC2 | BMI |
| 3.2574 | 1.12E-03 | GTExv8.EUR.Whole_Blood | 7.57E-03 | RP11-867G23.3 | BMI |
| 3.1595 | 1.58E-03 | GTExv8.EUR.Whole_Blood | 9.96E-03 | PELI3 | BMI |
| 3.2447 | 1.18E-03 | GTExv8.EUR.Whole_Blood | 7.91E-03 | CTD-3074O7.5 | BMI |
| 3.4052 | 6.61E-04 | GTExv8.EUR.Whole_Blood | 4.87E-03 | DPP3 | BMI |
| 3.1862 | 1.44E-03 | GTExv8.EUR.Whole_Blood | 9.26E-03 | BBS1 | BMI |
| -3.9507 | 7.79E-05 | GTExv8.EUR.Whole_Blood | 7.79E-04 | CTSF | BMI |
| 4.5452 | 5.49E-06 | GTExv8.EUR.Whole_Blood | 7.82E-05 | CCS | BMI |
| 4.5738 | 4.79E-06 | GTExv8.EUR.Whole_Blood | 6.88E-05 | RCE1 | BMI |
| 3.7750 | 1.60E-04 | GTExv8.EUR.Whole_Blood | 1.46E-03 | LRFN4 | BMI |
| 2.7798 | 5.44E-03 | GTExv8.EUR.Whole_Blood | 2.66E-02 | RAD9A | BMI |
| 2.9190 | 3.51E-03 | GTExv8.EUR.Whole_Blood | 1.87E-02 | AIP | BMI |
| -4.4547 | 8.40E-06 | GTExv8.EUR.Whole_Blood | 1.14E-04 | PPP6R3 | BMI |
| -5.2847 | 1.26E-07 | GTExv8.EUR.Whole_Blood | 2.75E-06 | MRPL21 | BMI |
| 5.5988 | 2.16E-08 | GTExv8.EUR.Whole_Blood | 5.82E-07 | IGHMBP2 | BMI |
| -3.5164 | 4.37E-04 | GTExv8.EUR.Whole_Blood | 3.42E-03 | TPCN2 | BMI |
| -3.4316 | 6.00E-04 | GTExv8.EUR.Whole_Blood | 4.50E-03 | RELT | BMI |
| -2.9252 | 3.44E-03 | GTExv8.EUR.Whole_Blood | 1.84E-02 | RP11-809N8.4 | BMI |
| -3.0086 | 2.62E-03 | GTExv8.EUR.Whole_Blood | 1.48E-02 | FAM168A | BMI |
| 2.8565 | 4.28E-03 | GTExv8.EUR.Whole_Blood | 2.22E-02 | RAB6A | BMI |
| -3.4455 | 5.70E-04 | GTExv8.EUR.Whole_Blood | 4.31E-03 | THAP12 | BMI |
| -2.6008 | 9.30E-03 | GTExv8.EUR.Whole_Blood | 4.05E-02 | MYO7A | BMI |
| -2.7065 | 6.80E-03 | GTExv8.EUR.Whole_Blood | 3.18E-02 | RSF1 | BMI |
| 2.5587 | 1.05E-02 | GTExv8.EUR.Whole_Blood | 4.46E-02 | INTS4 | BMI |
| -5.2992 | 1.16E-07 | GTExv8.EUR.Whole_Blood | 2.57E-06 | GAB2 | BMI |
| -4.0233 | 5.74E-05 | GTExv8.EUR.Whole_Blood | 6.01E-04 | RP11-452H21.4 | BMI |
| 3.6031 | 3.14E-04 | GTExv8.EUR.Whole_Blood | 2.59E-03 | NARS2 | BMI |
| -5.1850 | 2.16E-07 | GTExv8.EUR.Whole_Blood | 4.40E-06 | SLC25A1P1 | BMI |
| 2.6096 | 9.06E-03 | GTExv8.EUR.Whole_Blood | 3.96E-02 | EED | BMI |
| -3.1175 | 1.82E-03 | GTExv8.EUR.Whole_Blood | 1.11E-02 | RAB38 | BMI |
| 3.1382 | 1.70E-03 | GTExv8.EUR.Whole_Blood | 1.05E-02 | RP11-121L10.2 | BMI |
| 3.1503 | 1.63E-03 | GTExv8.EUR.Whole_Blood | 1.02E-02 | GPR83 | BMI |
| -3.6153 | 3.00E-04 | GTExv8.EUR.Whole_Blood | 2.49E-03 | RP11-685N10.1 | BMI |
| -3.8725 | 1.08E-04 | GTExv8.EUR.Whole_Blood | 1.05E-03 | MRE11 | BMI |
| 3.4362 | 5.90E-04 | GTExv8.EUR.Whole_Blood | 4.44E-03 | CWC15 | BMI |
| -2.8364 | 4.56E-03 | GTExv8.EUR.Whole_Blood | 2.34E-02 | RP11-819C21.1 | BMI |
| -2.7139 | 6.65E-03 | GTExv8.EUR.Whole_Blood | 3.13E-02 | DDX10 | BMI |
| 2.7157 | 6.61E-03 | GTExv8.EUR.Whole_Blood | 3.12E-02 | TTC12 | BMI |
| 2.6158 | 8.90E-03 | GTExv8.EUR.Whole_Blood | 3.91E-02 | RP11-159N11.4 | BMI |
| -5.0289 | 4.93E-07 | GTExv8.EUR.Whole_Blood | 8.90E-06 | ANKK1 | BMI |
| 2.9524 | 3.15E-03 | GTExv8.EUR.Whole_Blood | 1.72E-02 | HSPE1P18 | BMI |
| -2.5427 | 1.10E-02 | GTExv8.EUR.Whole_Blood | 4.60E-02 | MPZL2 | BMI |
| 3.2389 | 1.20E-03 | GTExv8.EUR.Whole_Blood | 8.00E-03 | ARCN1 | BMI |
| -6.7412 | 1.57E-11 | GTExv8.EUR.Whole_Blood | 8.73E-10 | RP11-110I1.14 | BMI |
| -7.3711 | 1.69E-13 | GTExv8.EUR.Whole_Blood | 1.43E-11 | RP11-110I1.13 | BMI |
| -4.4795 | 7.48E-06 | GTExv8.EUR.Whole_Blood | 1.03E-04 | C2CD2L | BMI |
| 3.0538 | 2.26E-03 | GTExv8.EUR.Whole_Blood | 1.32E-02 | HINFP | BMI |
| -6.5842 | 4.57E-11 | GTExv8.EUR.Whole_Blood | 2.32E-09 | UBASH3B | BMI |
| 3.8667 | 1.10E-04 | GTExv8.EUR.Whole_Blood | 1.06E-03 | C11orf63 | BMI |
| 3.5880 | 3.33E-04 | GTExv8.EUR.Whole_Blood | 2.73E-03 | SLC37A2 | BMI |
| -3.5664 | 3.62E-04 | GTExv8.EUR.Whole_Blood | 2.92E-03 | DCPS | BMI |
| -2.9027 | 3.70E-03 | GTExv8.EUR.Whole_Blood | 1.96E-02 | ST3GAL4-AS1 | BMI |
| -4.9239 | 8.48E-07 | GTExv8.EUR.Whole_Blood | 1.46E-05 | RP11-890B15.3 | BMI |
| -4.3282 | 1.50E-05 | GTExv8.EUR.Whole_Blood | 1.90E-04 | SNX19 | BMI |
| -3.5827 | 3.40E-04 | GTExv8.EUR.Whole_Blood | 2.78E-03 | B3GAT1 | BMI |
| 3.8002 | 1.45E-04 | GTExv8.EUR.Whole_Blood | 1.34E-03 | RAD52 | BMI |
| 2.7882 | 5.30E-03 | GTExv8.EUR.Whole_Blood | 2.61E-02 | SPSB2 | BMI |
| 2.7882 | 5.30E-03 | GTExv8.EUR.Whole_Blood | 2.61E-02 | LRRC23 | BMI |
| 2.5479 | 1.08E-02 | GTExv8.EUR.Whole_Blood | 4.56E-02 | GPR19 | BMI |
| 2.7612 | 5.76E-03 | GTExv8.EUR.Whole_Blood | 2.79E-02 | ERP27 | BMI |
| 5.4963 | 3.88E-08 | GTExv8.EUR.Whole_Blood | 9.68E-07 | PLEKHA5 | BMI |
| -3.3595 | 7.81E-04 | GTExv8.EUR.Whole_Blood | 5.61E-03 | RP11-967K21.1 | BMI |
| 2.6915 | 7.11E-03 | GTExv8.EUR.Whole_Blood | 3.29E-02 | TMTC1 | BMI |
| -2.7163 | 6.60E-03 | GTExv8.EUR.Whole_Blood | 3.12E-02 | BICD1 | BMI |
| 5.2286 | 1.71E-07 | GTExv8.EUR.Whole_Blood | 3.59E-06 | FGD4 | BMI |
| 2.8395 | 4.52E-03 | GTExv8.EUR.Whole_Blood | 2.32E-02 | GXYLT1 | BMI |
| 4.3776 | 1.20E-05 | GTExv8.EUR.Whole_Blood | 1.57E-04 | PPHLN1 | BMI |
| -3.2925 | 9.93E-04 | GTExv8.EUR.Whole_Blood | 6.83E-03 | RP11-23J18.1 | BMI |
| -2.8171 | 4.85E-03 | GTExv8.EUR.Whole_Blood | 2.44E-02 | RP11-493L12.4 | BMI |
| -3.5848 | 3.37E-04 | GTExv8.EUR.Whole_Blood | 2.75E-03 | RP5-1057I20.5 | BMI |
| -3.1706 | 1.52E-03 | GTExv8.EUR.Whole_Blood | 9.65E-03 | KANSL2 | BMI |
| 3.7630 | 1.68E-04 | GTExv8.EUR.Whole_Blood | 1.53E-03 | CCNT1 | BMI |
| 4.2762 | 1.90E-05 | GTExv8.EUR.Whole_Blood | 2.32E-04 | CACNB3 | BMI |
| 5.0596 | 4.20E-07 | GTExv8.EUR.Whole_Blood | 7.82E-06 | PRKAG1 | BMI |
| -4.1647 | 3.12E-05 | GTExv8.EUR.Whole_Blood | 3.57E-04 | RP11-386G11.10 | BMI |
| -4.3581 | 1.31E-05 | GTExv8.EUR.Whole_Blood | 1.70E-04 | TUBA1A | BMI |
| -3.0902 | 2.00E-03 | GTExv8.EUR.Whole_Blood | 1.20E-02 | RP4-605O3.4 | BMI |
| -3.4430 | 5.75E-04 | GTExv8.EUR.Whole_Blood | 4.34E-03 | RP11-454E5.4 | BMI |
| -3.7206 | 1.99E-04 | GTExv8.EUR.Whole_Blood | 1.75E-03 | POU6F1 | BMI |
| -3.4919 | 4.80E-04 | GTExv8.EUR.Whole_Blood | 3.70E-03 | CELA1 | BMI |
| 3.7952 | 1.48E-04 | GTExv8.EUR.Whole_Blood | 1.37E-03 | SPRYD3 | BMI |
| -6.0620 | 1.34E-09 | GTExv8.EUR.Whole_Blood | 4.76E-08 | SOAT2 | BMI |
| 5.4495 | 5.05E-08 | GTExv8.EUR.Whole_Blood | 1.23E-06 | SP1 | BMI |
| -3.3656 | 7.64E-04 | GTExv8.EUR.Whole_Blood | 5.50E-03 | TARBP2 | BMI |
| 4.2884 | 1.80E-05 | GTExv8.EUR.Whole_Blood | 2.22E-04 | ATP5G2 | BMI |
| 5.3999 | 6.67E-08 | GTExv8.EUR.Whole_Blood | 1.56E-06 | HNRNPA1 | BMI |
| -4.5915 | 4.40E-06 | GTExv8.EUR.Whole_Blood | 6.36E-05 | RP11-644F5.11 | BMI |
| 5.7219 | 1.05E-08 | GTExv8.EUR.Whole_Blood | 3.02E-07 | SUOX | BMI |
| -7.0151 | 2.30E-12 | GTExv8.EUR.Whole_Blood | 1.52E-10 | RPS26 | BMI |
| 2.5695 | 1.02E-02 | GTExv8.EUR.Whole_Blood | 4.35E-02 | SMARCC2 | BMI |
| -6.0748 | 1.24E-09 | GTExv8.EUR.Whole_Blood | 4.47E-08 | SPRYD4 | BMI |
| -5.5571 | 2.74E-08 | GTExv8.EUR.Whole_Blood | 7.13E-07 | PIP4K2C | BMI |
| -2.9472 | 3.21E-03 | GTExv8.EUR.Whole_Blood | 1.75E-02 | OS9 | BMI |
| -3.4977 | 4.69E-04 | GTExv8.EUR.Whole_Blood | 3.64E-03 | USP15 | BMI |
| -3.5210 | 4.30E-04 | GTExv8.EUR.Whole_Blood | 3.39E-03 | FAM19A2 | BMI |
| 2.6966 | 7.00E-03 | GTExv8.EUR.Whole_Blood | 3.25E-02 | GNS | BMI |
| 2.6326 | 8.47E-03 | GTExv8.EUR.Whole_Blood | 3.76E-02 | TBC1D30 | BMI |
| 4.5950 | 4.33E-06 | GTExv8.EUR.Whole_Blood | 6.29E-05 | RP11-1143G9.5 | BMI |
| 4.5702 | 4.87E-06 | GTExv8.EUR.Whole_Blood | 6.98E-05 | LYZ | BMI |
| 4.0907 | 4.30E-05 | GTExv8.EUR.Whole_Blood | 4.68E-04 | YEATS4 | BMI |
| 2.9625 | 3.05E-03 | GTExv8.EUR.Whole_Blood | 1.68E-02 | LINC01481 | BMI |
| -5.6162 | 1.95E-08 | GTExv8.EUR.Whole_Blood | 5.29E-07 | GALNT4 | BMI |
| -4.8691 | 1.12E-06 | GTExv8.EUR.Whole_Blood | 1.87E-05 | POC1B | BMI |
| 5.3836 | 7.30E-08 | GTExv8.EUR.Whole_Blood | 1.70E-06 | RP11-981P6.1 | BMI |
| 4.6276 | 3.70E-06 | GTExv8.EUR.Whole_Blood | 5.49E-05 | APPL2 | BMI |
| -3.5238 | 4.25E-04 | GTExv8.EUR.Whole_Blood | 3.35E-03 | PRDM4 | BMI |
| 2.7646 | 5.70E-03 | GTExv8.EUR.Whole_Blood | 2.76E-02 | ISCU | BMI |
| -2.8126 | 4.91E-03 | GTExv8.EUR.Whole_Blood | 2.46E-02 | USP30-AS1 | BMI |
| 5.3541 | 8.60E-08 | GTExv8.EUR.Whole_Blood | 1.97E-06 | KCTD10 | BMI |
| 5.5943 | 2.22E-08 | GTExv8.EUR.Whole_Blood | 5.94E-07 | UBE3B | BMI |
| -5.1903 | 2.10E-07 | GTExv8.EUR.Whole_Blood | 4.30E-06 | MVK | BMI |
| -2.6190 | 8.82E-03 | GTExv8.EUR.Whole_Blood | 3.87E-02 | MMAB | BMI |
| -3.5776 | 3.47E-04 | GTExv8.EUR.Whole_Blood | 2.82E-03 | TRPV4 | BMI |
| -6.4837 | 8.95E-11 | GTExv8.EUR.Whole_Blood | 4.17E-09 | ARPC3 | BMI |
| -6.2842 | 3.30E-10 | GTExv8.EUR.Whole_Blood | 1.32E-08 | FAM216A | BMI |
| -6.3645 | 1.96E-10 | GTExv8.EUR.Whole_Blood | 8.20E-09 | GPN3 | BMI |
| 6.9825 | 2.90E-12 | GTExv8.EUR.Whole_Blood | 1.81E-10 | VPS29 | BMI |
| 7.2005 | 6.00E-13 | GTExv8.EUR.Whole_Blood | 4.53E-11 | RP3-424M6.4 | BMI |
| 6.9825 | 2.90E-12 | GTExv8.EUR.Whole_Blood | 1.81E-10 | RAD9B | BMI |
| 3.8517 | 1.17E-04 | GTExv8.EUR.Whole_Blood | 1.12E-03 | ALDH2 | BMI |
| 3.5089 | 4.50E-04 | GTExv8.EUR.Whole_Blood | 3.51E-03 | OAS3 | BMI |
| 3.4455 | 5.70E-04 | GTExv8.EUR.Whole_Blood | 4.31E-03 | RASAL1 | BMI |
| -5.0213 | 5.13E-07 | GTExv8.EUR.Whole_Blood | 9.20E-06 | RITA1 | BMI |
| 4.6923 | 2.70E-06 | GTExv8.EUR.Whole_Blood | 4.14E-05 | IQCD | BMI |
| -5.6505 | 1.60E-08 | GTExv8.EUR.Whole_Blood | 4.43E-07 | PLBD2 | BMI |
| 2.9677 | 3.00E-03 | GTExv8.EUR.Whole_Blood | 1.66E-02 | HRK | BMI |
| 3.2760 | 1.05E-03 | GTExv8.EUR.Whole_Blood | 7.16E-03 | FBXW8 | BMI |
| 2.7317 | 6.30E-03 | GTExv8.EUR.Whole_Blood | 3.00E-02 | TESC | BMI |
| -3.3562 | 7.90E-04 | GTExv8.EUR.Whole_Blood | 5.67E-03 | GATC | BMI |
| -2.5263 | 1.15E-02 | GTExv8.EUR.Whole_Blood | 4.78E-02 | OASL | BMI |
| -3.3056 | 9.48E-04 | GTExv8.EUR.Whole_Blood | 6.63E-03 | CAMKK2 | BMI |
| 2.6929 | 7.08E-03 | GTExv8.EUR.Whole_Blood | 3.28E-02 | ORAI1 | BMI |
| 2.8179 | 4.83E-03 | GTExv8.EUR.Whole_Blood | 2.43E-02 | MORN3 | BMI |
| -3.2160 | 1.30E-03 | GTExv8.EUR.Whole_Blood | 8.56E-03 | RP11-347I19.8 | BMI |
| -3.4808 | 5.00E-04 | GTExv8.EUR.Whole_Blood | 3.83E-03 | RHOF | BMI |
| 3.4227 | 6.20E-04 | GTExv8.EUR.Whole_Blood | 4.61E-03 | HPD | BMI |
| 4.8079 | 1.52E-06 | GTExv8.EUR.Whole_Blood | 2.46E-05 | PSMD9 | BMI |
| 5.0364 | 4.74E-07 | GTExv8.EUR.Whole_Blood | 8.68E-06 | LRRC43 | BMI |
| 9.6934 | 3.22E-22 | GTExv8.EUR.Whole_Blood | 4.47E-20 | CCDC150P1 | BMI |
| -10.1475 | 3.40E-24 | GTExv8.EUR.Whole_Blood | 5.40E-22 | RSRC2 | BMI |
| -4.9809 | 6.33E-07 | GTExv8.EUR.Whole_Blood | 1.12E-05 | RP11-324E6.6 | BMI |
| 2.6866 | 7.22E-03 | GTExv8.EUR.Whole_Blood | 3.32E-02 | RILPL1 | BMI |
| 5.0508 | 4.40E-07 | GTExv8.EUR.Whole_Blood | 8.13E-06 | DDX55 | BMI |
| -4.0584 | 4.94E-05 | GTExv8.EUR.Whole_Blood | 5.27E-04 | TCTN2 | BMI |
| -3.3051 | 9.49E-04 | GTExv8.EUR.Whole_Blood | 6.64E-03 | ATP6V0A2 | BMI |
| 5.4582 | 4.81E-08 | GTExv8.EUR.Whole_Blood | 1.18E-06 | CCDC92 | BMI |
| -5.5201 | 3.39E-08 | GTExv8.EUR.Whole_Blood | 8.59E-07 | ZNF664 | BMI |
| -3.6480 | 2.64E-04 | GTExv8.EUR.Whole_Blood | 2.24E-03 | AACS | BMI |
| -3.6241 | 2.90E-04 | GTExv8.EUR.Whole_Blood | 2.42E-03 | RP11-158L12.4 | BMI |
| -2.8202 | 4.80E-03 | GTExv8.EUR.Whole_Blood | 2.42E-02 | SFSWAP | BMI |
| -3.5028 | 4.60E-04 | GTExv8.EUR.Whole_Blood | 3.57E-03 | RP13-977J11.2 | BMI |
| 6.1477 | 7.86E-10 | GTExv8.EUR.Whole_Blood | 2.94E-08 | ANKLE2 | BMI |
| 4.0184 | 5.86E-05 | GTExv8.EUR.Whole_Blood | 6.11E-04 | ZNF605 | BMI |
| 3.7457 | 1.80E-04 | GTExv8.EUR.Whole_Blood | 1.61E-03 | ZNF84 | BMI |
| -5.2788 | 1.30E-07 | GTExv8.EUR.Whole_Blood | 2.82E-06 | GTF3A | BMI |
| 3.3420 | 8.32E-04 | GTExv8.EUR.Whole_Blood | 5.95E-03 | LNX2 | BMI |
| 2.9651 | 3.03E-03 | GTExv8.EUR.Whole_Blood | 1.67E-02 | POLR1D | BMI |
| -2.8761 | 4.03E-03 | GTExv8.EUR.Whole_Blood | 2.10E-02 | SLC7A1 | BMI |
| 6.2914 | 3.15E-10 | GTExv8.EUR.Whole_Blood | 1.27E-08 | KL | BMI |
| 3.8287 | 1.29E-04 | GTExv8.EUR.Whole_Blood | 1.22E-03 | SPG20 | BMI |
| -2.8764 | 4.02E-03 | GTExv8.EUR.Whole_Blood | 2.10E-02 | CCNA1 | BMI |
| 2.5382 | 1.11E-02 | GTExv8.EUR.Whole_Blood | 4.64E-02 | DNAJC15 | BMI |
| -2.6783 | 7.40E-03 | GTExv8.EUR.Whole_Blood | 3.38E-02 | ZC3H13 | BMI |
| 2.7227 | 6.48E-03 | GTExv8.EUR.Whole_Blood | 3.07E-02 | RP11-90M2.5 | BMI |
| -3.3187 | 9.04E-04 | GTExv8.EUR.Whole_Blood | 6.37E-03 | TRIM13 | BMI |
| -2.9347 | 3.34E-03 | GTExv8.EUR.Whole_Blood | 1.80E-02 | RNASEH2B | BMI |
| 2.5121 | 1.20E-02 | GTExv8.EUR.Whole_Blood | 4.94E-02 | ATP7B | BMI |
| -2.6503 | 8.04E-03 | GTExv8.EUR.Whole_Blood | 3.61E-02 | CKAP2 | BMI |
| 5.1763 | 2.26E-07 | GTExv8.EUR.Whole_Blood | 4.58E-06 | SLAIN1 | BMI |
| -4.2763 | 1.90E-05 | GTExv8.EUR.Whole_Blood | 2.32E-04 | RNF219 | BMI |
| 4.8165 | 1.46E-06 | GTExv8.EUR.Whole_Blood | 2.38E-05 | RBM26 | BMI |
| -3.6795 | 2.34E-04 | GTExv8.EUR.Whole_Blood | 2.02E-03 | CLDN10 | BMI |
| -4.5336 | 5.80E-06 | GTExv8.EUR.Whole_Blood | 8.22E-05 | DNAJC3-AS1 | BMI |
| -4.0010 | 6.31E-05 | GTExv8.EUR.Whole_Blood | 6.52E-04 | DNAJC3 | BMI |
| -5.8282 | 5.60E-09 | GTExv8.EUR.Whole_Blood | 1.72E-07 | RAP2A | BMI |
| -6.4431 | 1.17E-10 | GTExv8.EUR.Whole_Blood | 5.29E-09 | FARP1 | BMI |
| -5.5978 | 2.17E-08 | GTExv8.EUR.Whole_Blood | 5.82E-07 | STK24 | BMI |
| -2.6828 | 7.30E-03 | GTExv8.EUR.Whole_Blood | 3.34E-02 | TPP2 | BMI |
| 3.4095 | 6.51E-04 | GTExv8.EUR.Whole_Blood | 4.81E-03 | RNASE4 | BMI |
| 3.0197 | 2.53E-03 | GTExv8.EUR.Whole_Blood | 1.44E-02 | ANG | BMI |
| 3.9015 | 9.56E-05 | GTExv8.EUR.Whole_Blood | 9.36E-04 | ABHD4 | BMI |
| 2.8383 | 4.54E-03 | GTExv8.EUR.Whole_Blood | 2.33E-02 | MRPL52 | BMI |
| 2.6437 | 8.20E-03 | GTExv8.EUR.Whole_Blood | 3.66E-02 | PSMB5 | BMI |
| -2.8060 | 5.02E-03 | GTExv8.EUR.Whole_Blood | 2.50E-02 | CARMIL3 | BMI |
| -5.2653 | 1.40E-07 | GTExv8.EUR.Whole_Blood | 3.03E-06 | KIAA0391 | BMI |
| -3.2238 | 1.27E-03 | GTExv8.EUR.Whole_Blood | 8.42E-03 | PTGDR | BMI |
| -3.4900 | 4.83E-04 | GTExv8.EUR.Whole_Blood | 3.72E-03 | ERO1A | BMI |
| -4.3737 | 1.22E-05 | GTExv8.EUR.Whole_Blood | 1.59E-04 | PSMC6 | BMI |
| 4.4736 | 7.69E-06 | GTExv8.EUR.Whole_Blood | 1.06E-04 | STYX | BMI |
| 2.7809 | 5.42E-03 | GTExv8.EUR.Whole_Blood | 2.65E-02 | GCH1 | BMI |
| -4.2122 | 2.53E-05 | GTExv8.EUR.Whole_Blood | 2.98E-04 | KTN1 | BMI |
| 4.2550 | 2.09E-05 | GTExv8.EUR.Whole_Blood | 2.52E-04 | RTN1 | BMI |
| -3.1495 | 1.64E-03 | GTExv8.EUR.Whole_Blood | 1.03E-02 | FNTB | BMI |
| -3.2538 | 1.14E-03 | GTExv8.EUR.Whole_Blood | 7.67E-03 | FUT8 | BMI |
| 2.6769 | 7.43E-03 | GTExv8.EUR.Whole_Blood | 3.39E-02 | PLEKHD1 | BMI |
| 3.1759 | 1.49E-03 | GTExv8.EUR.Whole_Blood | 9.51E-03 | ADAM20P1 | BMI |
| 7.1047 | 1.21E-12 | GTExv8.EUR.Whole_Blood | 8.48E-11 | DCAF4 | BMI |
| 2.7367 | 6.21E-03 | GTExv8.EUR.Whole_Blood | 2.96E-02 | ZFYVE1 | BMI |
| 4.5418 | 5.58E-06 | GTExv8.EUR.Whole_Blood | 7.94E-05 | RP4-647C14.3 | BMI |
| 4.6217 | 3.81E-06 | GTExv8.EUR.Whole_Blood | 5.62E-05 | ACOT2 | BMI |
| -3.6073 | 3.09E-04 | GTExv8.EUR.Whole_Blood | 2.56E-03 | RP5-1021I20.1 | BMI |
| 3.2577 | 1.12E-03 | GTExv8.EUR.Whole_Blood | 7.57E-03 | ZNF410 | BMI |
| 3.7456 | 1.80E-04 | GTExv8.EUR.Whole_Blood | 1.61E-03 | COQ6 | BMI |
| 3.6241 | 2.90E-04 | GTExv8.EUR.Whole_Blood | 2.42E-03 | FAM161B | BMI |
| -4.9482 | 7.49E-07 | GTExv8.EUR.Whole_Blood | 1.30E-05 | IRF2BPL | BMI |
| -3.0133 | 2.58E-03 | GTExv8.EUR.Whole_Blood | 1.47E-02 | TMEM63C | BMI |
| 3.5752 | 3.50E-04 | GTExv8.EUR.Whole_Blood | 2.84E-03 | ALKBH1 | BMI |
| -3.3088 | 9.37E-04 | GTExv8.EUR.Whole_Blood | 6.56E-03 | SNW1 | BMI |
| 4.4038 | 1.06E-05 | GTExv8.EUR.Whole_Blood | 1.40E-04 | GPR65 | BMI |
| -3.4248 | 6.15E-04 | GTExv8.EUR.Whole_Blood | 4.59E-03 | TTC7B | BMI |
| 2.9432 | 3.25E-03 | GTExv8.EUR.Whole_Blood | 1.76E-02 | NDUFB1 | BMI |
| 3.1433 | 1.67E-03 | GTExv8.EUR.Whole_Blood | 1.04E-02 | BTBD7 | BMI |
| 3.8082 | 1.40E-04 | GTExv8.EUR.Whole_Blood | 1.31E-03 | YY1 | BMI |
| 2.5973 | 9.40E-03 | GTExv8.EUR.Whole_Blood | 4.08E-02 | RP11-8L8.2 | BMI |
| 3.2830 | 1.03E-03 | GTExv8.EUR.Whole_Blood | 7.04E-03 | RP11-1029J19.4 | BMI |
| 3.1054 | 1.90E-03 | GTExv8.EUR.Whole_Blood | 1.15E-02 | LINC00239 | BMI |
| -5.3704 | 7.86E-08 | GTExv8.EUR.Whole_Blood | 1.82E-06 | RP11-1017G21.5 | BMI |
| -5.8581 | 4.68E-09 | GTExv8.EUR.Whole_Blood | 1.47E-07 | RP11-1017G21.6 | BMI |
| 2.6875 | 7.20E-03 | GTExv8.EUR.Whole_Blood | 3.31E-02 | MARK3 | BMI |
| -3.1559 | 1.60E-03 | GTExv8.EUR.Whole_Blood | 1.01E-02 | CKB | BMI |
| -5.1141 | 3.15E-07 | GTExv8.EUR.Whole_Blood | 6.10E-06 | TRMT61A | BMI |
| 2.9191 | 3.51E-03 | GTExv8.EUR.Whole_Blood | 1.87E-02 | KLC1 | BMI |
| 5.7046 | 1.17E-08 | GTExv8.EUR.Whole_Blood | 3.32E-07 | BAG5 | BMI |
| 3.4272 | 6.10E-04 | GTExv8.EUR.Whole_Blood | 4.56E-03 | RP11-73M18.8 | BMI |
| 7.1306 | 9.99E-13 | GTExv8.EUR.Whole_Blood | 7.20E-11 | XRCC3 | BMI |
| 5.0386 | 4.69E-07 | GTExv8.EUR.Whole_Blood | 8.61E-06 | NDN | BMI |
| 2.6213 | 8.76E-03 | GTExv8.EUR.Whole_Blood | 3.86E-02 | RP11-540B6.2 | BMI |
| 3.2389 | 1.20E-03 | GTExv8.EUR.Whole_Blood | 8.00E-03 | MTMR10 | BMI |
| -2.6190 | 8.82E-03 | GTExv8.EUR.Whole_Blood | 3.87E-02 | GOLGA8A | BMI |
| 3.8082 | 1.40E-04 | GTExv8.EUR.Whole_Blood | 1.31E-03 | C15orf41 | BMI |
| 2.6917 | 7.11E-03 | GTExv8.EUR.Whole_Blood | 3.29E-02 | SRP14-AS1 | BMI |
| 2.5122 | 1.20E-02 | GTExv8.EUR.Whole_Blood | 4.94E-02 | GCHFR | BMI |
| -3.4549 | 5.50E-04 | GTExv8.EUR.Whole_Blood | 4.18E-03 | CHP1 | BMI |
| 4.7135 | 2.44E-06 | GTExv8.EUR.Whole_Blood | 3.77E-05 | NDUFAF1 | BMI |
| 3.7599 | 1.70E-04 | GTExv8.EUR.Whole_Blood | 1.54E-03 | RTF1 | BMI |
| 2.7955 | 5.18E-03 | GTExv8.EUR.Whole_Blood | 2.57E-02 | ITPKA | BMI |
| -3.7349 | 1.88E-04 | GTExv8.EUR.Whole_Blood | 1.67E-03 | RPAP1 | BMI |
| -3.4016 | 6.70E-04 | GTExv8.EUR.Whole_Blood | 4.93E-03 | PLA2G4B | BMI |
| -2.5913 | 9.56E-03 | GTExv8.EUR.Whole_Blood | 4.14E-02 | SPTBN5 | BMI |
| 3.4059 | 6.59E-04 | GTExv8.EUR.Whole_Blood | 4.86E-03 | RP11-23P13.6 | BMI |
| 4.4172 | 1.00E-05 | GTExv8.EUR.Whole_Blood | 1.33E-04 | LCMT2 | BMI |
| 4.2059 | 2.60E-05 | GTExv8.EUR.Whole_Blood | 3.05E-04 | ADAL | BMI |
| 4.3847 | 1.16E-05 | GTExv8.EUR.Whole_Blood | 1.53E-04 | ZSCAN29 | BMI |
| 5.1966 | 2.03E-07 | GTExv8.EUR.Whole_Blood | 4.17E-06 | CATSPER2 | BMI |
| 2.5866 | 9.69E-03 | GTExv8.EUR.Whole_Blood | 4.19E-02 | PDIA3 | BMI |
| -2.9100 | 3.61E-03 | GTExv8.EUR.Whole_Blood | 1.92E-02 | SORD2P | BMI |
| 4.3601 | 1.30E-05 | GTExv8.EUR.Whole_Blood | 1.69E-04 | CTD-2651B20.1 | BMI |
| -2.5122 | 1.20E-02 | GTExv8.EUR.Whole_Blood | 4.94E-02 | SHF | BMI |
| -5.7345 | 9.78E-09 | GTExv8.EUR.Whole_Blood | 2.83E-07 | TMOD2 | BMI |
| 2.7392 | 6.16E-03 | GTExv8.EUR.Whole_Blood | 2.94E-02 | CERNA1 | BMI |
| 3.1931 | 1.41E-03 | GTExv8.EUR.Whole_Blood | 9.08E-03 | FAM214A | BMI |
| -3.7374 | 1.86E-04 | GTExv8.EUR.Whole_Blood | 1.65E-03 | RSL24D1 | BMI |
| -3.0882 | 2.01E-03 | GTExv8.EUR.Whole_Blood | 1.21E-02 | C15orf65 | BMI |
| 2.8861 | 3.90E-03 | GTExv8.EUR.Whole_Blood | 2.04E-02 | RFX7 | BMI |
| -4.3219 | 1.55E-05 | GTExv8.EUR.Whole_Blood | 1.96E-04 | TCF12 | BMI |
| -3.6398 | 2.73E-04 | GTExv8.EUR.Whole_Blood | 2.30E-03 | RP11-50C13.1 | BMI |
| 4.1545 | 3.26E-05 | GTExv8.EUR.Whole_Blood | 3.69E-04 | GTF2A2 | BMI |
| -2.8338 | 4.60E-03 | GTExv8.EUR.Whole_Blood | 2.34E-02 | LACTB | BMI |
| -6.7687 | 1.30E-11 | GTExv8.EUR.Whole_Blood | 7.33E-10 | USP3 | BMI |
| -4.4180 | 9.96E-06 | GTExv8.EUR.Whole_Blood | 1.33E-04 | USP3-AS1 | BMI |
| -3.5807 | 3.43E-04 | GTExv8.EUR.Whole_Blood | 2.80E-03 | HERC1 | BMI |
| 2.6789 | 7.39E-03 | GTExv8.EUR.Whole_Blood | 3.38E-02 | SNX1 | BMI |
| 2.9921 | 2.77E-03 | GTExv8.EUR.Whole_Blood | 1.55E-02 | PPIB | BMI |
| 2.8499 | 4.37E-03 | GTExv8.EUR.Whole_Blood | 2.26E-02 | CSNK1G1 | BMI |
| 4.3310 | 1.48E-05 | GTExv8.EUR.Whole_Blood | 1.89E-04 | TRIP4 | BMI |
| 4.0673 | 4.76E-05 | GTExv8.EUR.Whole_Blood | 5.10E-04 | PIF1 | BMI |
| 4.1980 | 2.69E-05 | GTExv8.EUR.Whole_Blood | 3.13E-04 | MTFMT | BMI |
| -2.9756 | 2.92E-03 | GTExv8.EUR.Whole_Blood | 1.62E-02 | INTS14 | BMI |
| -2.6045 | 9.20E-03 | GTExv8.EUR.Whole_Blood | 4.01E-02 | RAB11A | BMI |
| -4.4426 | 8.89E-06 | GTExv8.EUR.Whole_Blood | 1.20E-04 | DENND4A | BMI |
| -4.0453 | 5.23E-05 | GTExv8.EUR.Whole_Blood | 5.54E-04 | DIS3L | BMI |
| -3.1244 | 1.78E-03 | GTExv8.EUR.Whole_Blood | 1.09E-02 | TIPIN | BMI |
| -3.8217 | 1.33E-04 | GTExv8.EUR.Whole_Blood | 1.25E-03 | SNAPC5 | BMI |
| 8.3143 | 9.23E-17 | GTExv8.EUR.Whole_Blood | 9.58E-15 | MAP2K5 | BMI |
| 11.8502 | 2.15E-32 | GTExv8.EUR.Whole_Blood | 6.44E-30 | SKOR1 | BMI |
| -3.2468 | 1.17E-03 | GTExv8.EUR.Whole_Blood | 7.86E-03 | RP11-34F13.2 | BMI |
| -4.4482 | 8.66E-06 | GTExv8.EUR.Whole_Blood | 1.17E-04 | PIAS1 | BMI |
| 2.7927 | 5.23E-03 | GTExv8.EUR.Whole_Blood | 2.58E-02 | PAQR5 | BMI |
| -4.8982 | 9.67E-07 | GTExv8.EUR.Whole_Blood | 1.64E-05 | PARP6 | BMI |
| 3.2991 | 9.70E-04 | GTExv8.EUR.Whole_Blood | 6.73E-03 | NEO1 | BMI |
| -4.0212 | 5.79E-05 | GTExv8.EUR.Whole_Blood | 6.06E-04 | LOXL1-AS1 | BMI |
| -3.2991 | 9.70E-04 | GTExv8.EUR.Whole_Blood | 6.73E-03 | STOML1 | BMI |
| -4.0709 | 4.68E-05 | GTExv8.EUR.Whole_Blood | 5.03E-04 | PML | BMI |
| -3.9535 | 7.70E-05 | GTExv8.EUR.Whole_Blood | 7.72E-04 | ULK3 | BMI |
| 5.0086 | 5.48E-07 | GTExv8.EUR.Whole_Blood | 9.78E-06 | SCAMP2 | BMI |
| 5.1658 | 2.39E-07 | GTExv8.EUR.Whole_Blood | 4.81E-06 | MPI | BMI |
| 3.3013 | 9.62E-04 | GTExv8.EUR.Whole_Blood | 6.70E-03 | UBE2Q2 | BMI |
| 3.4272 | 6.10E-04 | GTExv8.EUR.Whole_Blood | 4.56E-03 | FBXO22 | BMI |
| 3.5986 | 3.20E-04 | GTExv8.EUR.Whole_Blood | 2.63E-03 | RP11-797A18.5 | BMI |
| -6.3897 | 1.66E-10 | GTExv8.EUR.Whole_Blood | 7.18E-09 | FAM103A1 | BMI |
| 2.8143 | 4.89E-03 | GTExv8.EUR.Whole_Blood | 2.45E-02 | C15orf40 | BMI |
| -2.8978 | 3.76E-03 | GTExv8.EUR.Whole_Blood | 1.99E-02 | RP11-382A20.3 | BMI |
| -3.3958 | 6.84E-04 | GTExv8.EUR.Whole_Blood | 5.00E-03 | SCAND2P | BMI |
| 2.5465 | 1.09E-02 | GTExv8.EUR.Whole_Blood | 4.58E-02 | RP11-493E3.1 | BMI |
| 2.5230 | 1.16E-02 | GTExv8.EUR.Whole_Blood | 4.82E-02 | CIB1 | BMI |
| 5.4794 | 4.27E-08 | GTExv8.EUR.Whole_Blood | 1.06E-06 | RP11-6O2.2 | BMI |
| -3.1997 | 1.38E-03 | GTExv8.EUR.Whole_Blood | 8.96E-03 | LRRC28 | BMI |
| 2.9027 | 3.70E-03 | GTExv8.EUR.Whole_Blood | 1.96E-02 | LA16c-OS12.2 | BMI |
| 5.0194 | 5.18E-07 | GTExv8.EUR.Whole_Blood | 9.27E-06 | MRPL28 | BMI |
| -6.4620 | 1.03E-10 | GTExv8.EUR.Whole_Blood | 4.74E-09 | Z97634.3 | BMI |
| 3.4503 | 5.60E-04 | GTExv8.EUR.Whole_Blood | 4.25E-03 | NME4 | BMI |
| 3.2311 | 1.23E-03 | GTExv8.EUR.Whole_Blood | 8.17E-03 | DECR2 | BMI |
| -2.8890 | 3.86E-03 | GTExv8.EUR.Whole_Blood | 2.02E-02 | WFIKKN1 | BMI |
| -4.3406 | 1.42E-05 | GTExv8.EUR.Whole_Blood | 1.82E-04 | WDR90 | BMI |
| 2.5739 | 1.01E-02 | GTExv8.EUR.Whole_Blood | 4.32E-02 | RHOT2 | BMI |
| -2.8296 | 4.66E-03 | GTExv8.EUR.Whole_Blood | 2.37E-02 | STUB1 | BMI |
| -2.6525 | 7.99E-03 | GTExv8.EUR.Whole_Blood | 3.59E-02 | WDR24 | BMI |
| 2.6502 | 8.04E-03 | GTExv8.EUR.Whole_Blood | 3.61E-02 | IFT140 | BMI |
| 4.1531 | 3.28E-05 | GTExv8.EUR.Whole_Blood | 3.71E-04 | MLST8 | BMI |
| -3.2494 | 1.16E-03 | GTExv8.EUR.Whole_Blood | 7.80E-03 | RNPS1 | BMI |
| 2.5680 | 1.02E-02 | GTExv8.EUR.Whole_Blood | 4.35E-02 | RP11-473M20.5 | BMI |
| 3.6261 | 2.88E-04 | GTExv8.EUR.Whole_Blood | 2.41E-03 | IL32 | BMI |
| 2.7150 | 6.63E-03 | GTExv8.EUR.Whole_Blood | 3.13E-02 | ZNF205 | BMI |
| 6.1476 | 7.87E-10 | GTExv8.EUR.Whole_Blood | 2.94E-08 | CLUAP1 | BMI |
| -5.7774 | 7.59E-09 | GTExv8.EUR.Whole_Blood | 2.28E-07 | DNASE1 | BMI |
| -6.4897 | 8.60E-11 | GTExv8.EUR.Whole_Blood | 4.06E-09 | RP11-461A8.5 | BMI |
| 7.2007 | 5.99E-13 | GTExv8.EUR.Whole_Blood | 4.53E-11 | TRAP1 | BMI |
| 5.3999 | 6.67E-08 | GTExv8.EUR.Whole_Blood | 1.56E-06 | SEPT12 | BMI |
| 5.3765 | 7.60E-08 | GTExv8.EUR.Whole_Blood | 1.76E-06 | SMIM22 | BMI |
| 7.2959 | 2.97E-13 | GTExv8.EUR.Whole_Blood | 2.43E-11 | RP11-127I20.5 | BMI |
| 3.0703 | 2.14E-03 | GTExv8.EUR.Whole_Blood | 1.27E-02 | ROGDI | BMI |
| -5.4554 | 4.89E-08 | GTExv8.EUR.Whole_Blood | 1.20E-06 | GLYR1 | BMI |
| 2.6444 | 8.18E-03 | GTExv8.EUR.Whole_Blood | 3.66E-02 | RP11-876N24.1 | BMI |
| -3.0431 | 2.34E-03 | GTExv8.EUR.Whole_Blood | 1.36E-02 | ZC3H7A | BMI |
| 3.1747 | 1.50E-03 | GTExv8.EUR.Whole_Blood | 9.54E-03 | NPIPB2 | BMI |
| 4.0787 | 4.53E-05 | GTExv8.EUR.Whole_Blood | 4.90E-04 | RP11-958N24.1 | BMI |
| 4.0567 | 4.98E-05 | GTExv8.EUR.Whole_Blood | 5.31E-04 | RRN3 | BMI |
| 4.2240 | 2.40E-05 | GTExv8.EUR.Whole_Blood | 2.85E-04 | NPIPP1 | BMI |
| 3.5170 | 4.36E-04 | GTExv8.EUR.Whole_Blood | 3.42E-03 | PKD1P6 | BMI |
| 3.2181 | 1.29E-03 | GTExv8.EUR.Whole_Blood | 8.51E-03 | NPIPA5 | BMI |
| 3.4009 | 6.72E-04 | GTExv8.EUR.Whole_Blood | 4.94E-03 | ABCC1 | BMI |
| -2.5899 | 9.60E-03 | GTExv8.EUR.Whole_Blood | 4.15E-02 | GDE1 | BMI |
| -10.1935 | 2.12E-24 | GTExv8.EUR.Whole_Blood | 3.51E-22 | KNOP1 | BMI |
| -2.7826 | 5.39E-03 | GTExv8.EUR.Whole_Blood | 2.64E-02 | PRKCB | BMI |
| -2.9566 | 3.11E-03 | GTExv8.EUR.Whole_Blood | 1.70E-02 | TNRC6A | BMI |
| -6.4914 | 8.51E-11 | GTExv8.EUR.Whole_Blood | 4.06E-09 | SLC5A11 | BMI |
| 2.8806 | 3.97E-03 | GTExv8.EUR.Whole_Blood | 2.08E-02 | GSG1L | BMI |
| 9.9731 | 2.00E-23 | GTExv8.EUR.Whole_Blood | 2.94E-21 | NPIPB6 | BMI |
| -15.2804 | 1.03E-52 | GTExv8.EUR.Whole_Blood | 7.29E-50 | NPIPB7 | BMI |
| -11.2545 | 2.20E-29 | GTExv8.EUR.Whole_Blood | 5.19E-27 | CLN3 | BMI |
| 11.5401 | 8.28E-31 | GTExv8.EUR.Whole_Blood | 2.30E-28 | IL27 | BMI |
| -10.6949 | 1.07E-26 | GTExv8.EUR.Whole_Blood | 1.98E-24 | SGF29 | BMI |
| 16.1114 | 2.12E-58 | GTExv8.EUR.Whole_Blood | 3.30E-55 | SULT1A2 | BMI |
| -8.3495 | 6.85E-17 | GTExv8.EUR.Whole_Blood | 7.20E-15 | SULT1A1 | BMI |
| 15.9549 | 2.63E-57 | GTExv8.EUR.Whole_Blood | 2.56E-54 | EIF3C | BMI |
| -16.0211 | 9.10E-58 | GTExv8.EUR.Whole_Blood | 1.01E-54 | ATXN2L | BMI |
| 15.4627 | 6.20E-54 | GTExv8.EUR.Whole_Blood | 4.82E-51 | TUFM | BMI |
| -14.7357 | 3.80E-49 | GTExv8.EUR.Whole_Blood | 2.46E-46 | SH2B1 | BMI |
| 16.0823 | 3.40E-58 | GTExv8.EUR.Whole_Blood | 4.41E-55 | ATP2A1 | BMI |
| 11.1072 | 1.16E-28 | GTExv8.EUR.Whole_Blood | 2.58E-26 | SPNS1 | BMI |
| 2.6575 | 7.87E-03 | GTExv8.EUR.Whole_Blood | 3.55E-02 | RP11-426C22.8 | BMI |
| -2.5130 | 1.20E-02 | GTExv8.EUR.Whole_Blood | 4.94E-02 | SNX29P2 | BMI |
| -7.2063 | 5.75E-13 | GTExv8.EUR.Whole_Blood | 4.52E-11 | BOLA2 | BMI |
| 3.3789 | 7.28E-04 | GTExv8.EUR.Whole_Blood | 5.27E-03 | SLX1B | BMI |
| 3.4066 | 6.58E-04 | GTExv8.EUR.Whole_Blood | 4.86E-03 | SMG1P2 | BMI |
| 4.5038 | 6.68E-06 | GTExv8.EUR.Whole_Blood | 9.33E-05 | RP11-368N21.5 | BMI |
| -2.7486 | 5.98E-03 | GTExv8.EUR.Whole_Blood | 2.88E-02 | PAGR1 | BMI |
| 7.4860 | 7.10E-14 | GTExv8.EUR.Whole_Blood | 6.07E-12 | MVP | BMI |
| -6.6965 | 2.13E-11 | GTExv8.EUR.Whole_Blood | 1.16E-09 | CDIPT-AS1 | BMI |
| -9.0141 | 1.98E-19 | GTExv8.EUR.Whole_Blood | 2.33E-17 | CTD-2574D22.4 | BMI |
| -12.6981 | 6.06E-37 | GTExv8.EUR.Whole_Blood | 2.48E-34 | INO80E | BMI |
| 10.1645 | 2.86E-24 | GTExv8.EUR.Whole_Blood | 4.64E-22 | TBX6 | BMI |
| 10.3675 | 3.49E-25 | GTExv8.EUR.Whole_Blood | 6.04E-23 | YPEL3 | BMI |
| 10.0531 | 8.90E-24 | GTExv8.EUR.Whole_Blood | 1.36E-21 | RP11-455F5.3 | BMI |
| -7.0096 | 2.39E-12 | GTExv8.EUR.Whole_Blood | 1.55E-10 | GDPD3 | BMI |
| -13.1026 | 3.18E-39 | GTExv8.EUR.Whole_Blood | 1.37E-36 | MAPK3 | BMI |
| -3.4808 | 5.00E-04 | GTExv8.EUR.Whole_Blood | 3.83E-03 | NPIPB13 | BMI |
| -10.5445 | 5.38E-26 | GTExv8.EUR.Whole_Blood | 9.52E-24 | SMG1P5 | BMI |
| -10.0418 | 9.98E-24 | GTExv8.EUR.Whole_Blood | 1.49E-21 | RP11-347C12.12 | BMI |
| -3.0648 | 2.18E-03 | GTExv8.EUR.Whole_Blood | 1.28E-02 | SEPT1 | BMI |
| 9.1575 | 5.31E-20 | GTExv8.EUR.Whole_Blood | 6.77E-18 | AC002310.14 | BMI |
| -2.6125 | 8.99E-03 | GTExv8.EUR.Whole_Blood | 3.94E-02 | RNF40 | BMI |
| -12.2060 | 2.89E-34 | GTExv8.EUR.Whole_Blood | 1.02E-31 | RP11-1072A3.3 | BMI |
| -12.0197 | 2.80E-33 | GTExv8.EUR.Whole_Blood | 9.08E-31 | HSD3B7 | BMI |
| 7.1640 | 7.84E-13 | GTExv8.EUR.Whole_Blood | 5.81E-11 | RP11-196G11.2 | BMI |
| -11.4627 | 2.03E-30 | GTExv8.EUR.Whole_Blood | 4.94E-28 | RP11-196G11.6 | BMI |
| -11.6765 | 1.68E-31 | GTExv8.EUR.Whole_Blood | 4.84E-29 | ZNF668 | BMI |
| -7.2037 | 5.86E-13 | GTExv8.EUR.Whole_Blood | 4.52E-11 | PRSS53 | BMI |
| 4.7579 | 1.96E-06 | GTExv8.EUR.Whole_Blood | 3.07E-05 | RP11-196G11.3 | BMI |
| -11.5157 | 1.10E-30 | GTExv8.EUR.Whole_Blood | 2.95E-28 | VKORC1 | BMI |
| -11.0615 | 1.93E-28 | GTExv8.EUR.Whole_Blood | 4.17E-26 | BCKDK | BMI |
| 6.3692 | 1.90E-10 | GTExv8.EUR.Whole_Blood | 7.99E-09 | KAT8 | BMI |
| 10.9492 | 6.70E-28 | GTExv8.EUR.Whole_Blood | 1.34E-25 | RP11-196G11.4 | BMI |
| -6.3692 | 1.90E-10 | GTExv8.EUR.Whole_Blood | 7.99E-09 | RP11-196G11.5 | BMI |
| -4.1166 | 3.84E-05 | GTExv8.EUR.Whole_Blood | 4.26E-04 | ITGAX | BMI |
| -5.1173 | 3.10E-07 | GTExv8.EUR.Whole_Blood | 6.02E-06 | RP11-120K18.3 | BMI |
| 2.9781 | 2.90E-03 | GTExv8.EUR.Whole_Blood | 1.61E-02 | RP11-44I10.3 | BMI |
| -3.0179 | 2.55E-03 | GTExv8.EUR.Whole_Blood | 1.45E-02 | CTC-527H23.3 | BMI |
| 6.6172 | 3.66E-11 | GTExv8.EUR.Whole_Blood | 1.91E-09 | AKTIP | BMI |
| -3.1214 | 1.80E-03 | GTExv8.EUR.Whole_Blood | 1.10E-02 | GNAO1 | BMI |
| 4.0144 | 5.96E-05 | GTExv8.EUR.Whole_Blood | 6.20E-04 | AMFR | BMI |
| -4.6500 | 3.32E-06 | GTExv8.EUR.Whole_Blood | 5.01E-05 | OGFOD1 | BMI |
| 3.7800 | 1.57E-04 | GTExv8.EUR.Whole_Blood | 1.44E-03 | GOT2 | BMI |
| 5.2089 | 1.90E-07 | GTExv8.EUR.Whole_Blood | 3.94E-06 | B3GNT9 | BMI |
| 2.8338 | 4.60E-03 | GTExv8.EUR.Whole_Blood | 2.34E-02 | ELMO3 | BMI |
| -2.6256 | 8.65E-03 | GTExv8.EUR.Whole_Blood | 3.83E-02 | LRRC29 | BMI |
| -3.0027 | 2.68E-03 | GTExv8.EUR.Whole_Blood | 1.51E-02 | ATP6V0D1 | BMI |
| -3.2421 | 1.19E-03 | GTExv8.EUR.Whole_Blood | 7.96E-03 | C16orf86 | BMI |
| -5.5681 | 2.58E-08 | GTExv8.EUR.Whole_Blood | 6.74E-07 | NFATC3 | BMI |
| 3.1418 | 1.68E-03 | GTExv8.EUR.Whole_Blood | 1.05E-02 | PLA2G15 | BMI |
| -6.1724 | 6.73E-10 | GTExv8.EUR.Whole_Blood | 2.57E-08 | SLC7A6 | BMI |
| -5.9626 | 2.48E-09 | GTExv8.EUR.Whole_Blood | 8.39E-08 | RP11-96D1.8 | BMI |
| 5.1504 | 2.60E-07 | GTExv8.EUR.Whole_Blood | 5.14E-06 | UTP4 | BMI |
| 5.7189 | 1.07E-08 | GTExv8.EUR.Whole_Blood | 3.06E-07 | TMED6 | BMI |
| 5.1615 | 2.45E-07 | GTExv8.EUR.Whole_Blood | 4.90E-06 | CYB5B | BMI |
| 7.0697 | 1.55E-12 | GTExv8.EUR.Whole_Blood | 1.08E-10 | NFAT5 | BMI |
| -5.2512 | 1.51E-07 | GTExv8.EUR.Whole_Blood | 3.23E-06 | WWP2 | BMI |
| -9.9005 | 4.14E-23 | GTExv8.EUR.Whole_Blood | 5.97E-21 | CLEC18A | BMI |
| -6.5006 | 8.00E-11 | GTExv8.EUR.Whole_Blood | 3.87E-09 | NPIPB14P | BMI |
| -3.6303 | 2.83E-04 | GTExv8.EUR.Whole_Blood | 2.38E-03 | PDXDC2P | BMI |
| -7.0461 | 1.84E-12 | GTExv8.EUR.Whole_Blood | 1.25E-10 | PDPR | BMI |
| 7.2289 | 4.87E-13 | GTExv8.EUR.Whole_Blood | 3.87E-11 | CLEC18C | BMI |
| -9.0578 | 1.33E-19 | GTExv8.EUR.Whole_Blood | 1.64E-17 | SMG1P7 | BMI |
| -9.0534 | 1.39E-19 | GTExv8.EUR.Whole_Blood | 1.69E-17 | EXOSC6 | BMI |
| -10.1137 | 4.81E-24 | GTExv8.EUR.Whole_Blood | 7.49E-22 | DDX19B | BMI |
| 4.9909 | 6.01E-07 | GTExv8.EUR.Whole_Blood | 1.07E-05 | DDX19A | BMI |
| -5.4409 | 5.30E-08 | GTExv8.EUR.Whole_Blood | 1.28E-06 | COG4 | BMI |
| -2.8120 | 4.92E-03 | GTExv8.EUR.Whole_Blood | 2.46E-02 | SF3B3 | BMI |
| -5.1341 | 2.83E-07 | GTExv8.EUR.Whole_Blood | 5.53E-06 | CMTR2 | BMI |
| -3.3278 | 8.75E-04 | GTExv8.EUR.Whole_Blood | 6.20E-03 | PHLPP2 | BMI |
| -3.8397 | 1.23E-04 | GTExv8.EUR.Whole_Blood | 1.17E-03 | HP | BMI |
| -3.9167 | 8.98E-05 | GTExv8.EUR.Whole_Blood | 8.89E-04 | HPR | BMI |
| -3.6727 | 2.40E-04 | GTExv8.EUR.Whole_Blood | 2.06E-03 | AC009120.5 | BMI |
| -2.5639 | 1.04E-02 | GTExv8.EUR.Whole_Blood | 4.42E-02 | GLG1 | BMI |
| -3.6326 | 2.81E-04 | GTExv8.EUR.Whole_Blood | 2.36E-03 | LDHD | BMI |
| -3.1200 | 1.81E-03 | GTExv8.EUR.Whole_Blood | 1.11E-02 | ZFP1 | BMI |
| 2.9433 | 3.25E-03 | GTExv8.EUR.Whole_Blood | 1.76E-02 | BCAR1 | BMI |
| 2.6700 | 7.58E-03 | GTExv8.EUR.Whole_Blood | 3.45E-02 | RP11-252K23.2 | BMI |
| 3.5332 | 4.11E-04 | GTExv8.EUR.Whole_Blood | 3.26E-03 | CFDP1 | BMI |
| 2.6887 | 7.17E-03 | GTExv8.EUR.Whole_Blood | 3.30E-02 | RP11-391L3.5 | BMI |
| 2.8465 | 4.42E-03 | GTExv8.EUR.Whole_Blood | 2.28E-02 | NECAB2 | BMI |
| 2.9957 | 2.74E-03 | GTExv8.EUR.Whole_Blood | 1.54E-02 | KLHDC4 | BMI |
| -3.1466 | 1.65E-03 | GTExv8.EUR.Whole_Blood | 1.03E-02 | CBFA2T3 | BMI |
| -4.6234 | 3.78E-06 | GTExv8.EUR.Whole_Blood | 5.58E-05 | ZNF778 | BMI |
| -5.7379 | 9.59E-09 | GTExv8.EUR.Whole_Blood | 2.81E-07 | AC137932.5 | BMI |
| -3.2190 | 1.29E-03 | GTExv8.EUR.Whole_Blood | 8.51E-03 | RPL13 | BMI |
| 2.7741 | 5.54E-03 | GTExv8.EUR.Whole_Blood | 2.70E-02 | SPATA33 | BMI |
| -3.3755 | 7.37E-04 | GTExv8.EUR.Whole_Blood | 5.33E-03 | SERPINF1 | BMI |
| 4.8280 | 1.38E-06 | GTExv8.EUR.Whole_Blood | 2.26E-05 | SRR | BMI |
| -2.6586 | 7.85E-03 | GTExv8.EUR.Whole_Blood | 3.54E-02 | ALOX15 | BMI |
| 4.4681 | 7.89E-06 | GTExv8.EUR.Whole_Blood | 1.08E-04 | PLD2 | BMI |
| 5.5200 | 3.39E-08 | GTExv8.EUR.Whole_Blood | 8.59E-07 | C17orf107 | BMI |
| 5.0820 | 3.73E-07 | GTExv8.EUR.Whole_Blood | 7.06E-06 | CHRNE | BMI |
| -5.0946 | 3.49E-07 | GTExv8.EUR.Whole_Blood | 6.67E-06 | SLC25A11 | BMI |
| 2.6124 | 8.99E-03 | GTExv8.EUR.Whole_Blood | 3.94E-02 | SPAG7 | BMI |
| 6.0684 | 1.29E-09 | GTExv8.EUR.Whole_Blood | 4.63E-08 | CAMTA2 | BMI |
| 5.5709 | 2.53E-08 | GTExv8.EUR.Whole_Blood | 6.65E-07 | INCA1 | BMI |
| 6.0426 | 1.52E-09 | GTExv8.EUR.Whole_Blood | 5.28E-08 | KIF1C | BMI |
| -3.3876 | 7.05E-04 | GTExv8.EUR.Whole_Blood | 5.13E-03 | AC012146.7 | BMI |
| 6.3901 | 1.66E-10 | GTExv8.EUR.Whole_Blood | 7.18E-09 | RABEP1 | BMI |
| -6.0790 | 1.21E-09 | GTExv8.EUR.Whole_Blood | 4.38E-08 | NUP88 | BMI |
| 3.6417 | 2.71E-04 | GTExv8.EUR.Whole_Blood | 2.29E-03 | C1QBP | BMI |
| -3.7387 | 1.85E-04 | GTExv8.EUR.Whole_Blood | 1.65E-03 | NLRP1 | BMI |
| -3.2607 | 1.11E-03 | GTExv8.EUR.Whole_Blood | 7.52E-03 | ACADVL | BMI |
| -2.5516 | 1.07E-02 | GTExv8.EUR.Whole_Blood | 4.53E-02 | DVL2 | BMI |
| 3.4702 | 5.20E-04 | GTExv8.EUR.Whole_Blood | 3.98E-03 | CTDNEP1 | BMI |
| -3.4361 | 5.90E-04 | GTExv8.EUR.Whole_Blood | 4.44E-03 | ELP5 | BMI |
| -2.6892 | 7.16E-03 | GTExv8.EUR.Whole_Blood | 3.30E-02 | GPS2 | BMI |
| -4.6451 | 3.40E-06 | GTExv8.EUR.Whole_Blood | 5.09E-05 | CHRNB1 | BMI |
| 3.0291 | 2.45E-03 | GTExv8.EUR.Whole_Blood | 1.41E-02 | ATP1B2 | BMI |
| -4.6296 | 3.66E-06 | GTExv8.EUR.Whole_Blood | 5.45E-05 | CHD3 | BMI |
| -3.1095 | 1.87E-03 | GTExv8.EUR.Whole_Blood | 1.14E-02 | KCNAB3 | BMI |
| -3.1720 | 1.51E-03 | GTExv8.EUR.Whole_Blood | 9.59E-03 | CNTROB | BMI |
| 2.9722 | 2.96E-03 | GTExv8.EUR.Whole_Blood | 1.64E-02 | CTC1 | BMI |
| -3.3931 | 6.91E-04 | GTExv8.EUR.Whole_Blood | 5.05E-03 | RCVRN | BMI |
| -3.0449 | 2.33E-03 | GTExv8.EUR.Whole_Blood | 1.35E-02 | GAS7 | BMI |
| -7.7623 | 8.34E-15 | GTExv8.EUR.Whole_Blood | 7.46E-13 | ADORA2B | BMI |
| 6.3318 | 2.42E-10 | GTExv8.EUR.Whole_Blood | 9.91E-09 | TTC19 | BMI |
| 6.3740 | 1.84E-10 | GTExv8.EUR.Whole_Blood | 7.87E-09 | CTC-529I10.1 | BMI |
| -4.2819 | 1.85E-05 | GTExv8.EUR.Whole_Blood | 2.27E-04 | CTC-529I10.2 | BMI |
| -5.8321 | 5.47E-09 | GTExv8.EUR.Whole_Blood | 1.70E-07 | NCOR1 | BMI |
| -4.3965 | 1.10E-05 | GTExv8.EUR.Whole_Blood | 1.45E-04 | PIGL | BMI |
| -3.9183 | 8.92E-05 | GTExv8.EUR.Whole_Blood | 8.84E-04 | CENPV | BMI |
| 2.5682 | 1.02E-02 | GTExv8.EUR.Whole_Blood | 4.35E-02 | NT5M | BMI |
| 3.6241 | 2.90E-04 | GTExv8.EUR.Whole_Blood | 2.42E-03 | RP11-524F11.1 | BMI |
| 3.1636 | 1.56E-03 | GTExv8.EUR.Whole_Blood | 9.87E-03 | PEMT | BMI |
| 3.6739 | 2.39E-04 | GTExv8.EUR.Whole_Blood | 2.06E-03 | SREBF1 | BMI |
| 3.2116 | 1.32E-03 | GTExv8.EUR.Whole_Blood | 8.64E-03 | TOM1L2 | BMI |
| 3.0357 | 2.40E-03 | GTExv8.EUR.Whole_Blood | 1.38E-02 | DRG2 | BMI |
| 2.7275 | 6.38E-03 | GTExv8.EUR.Whole_Blood | 3.03E-02 | ALKBH5 | BMI |
| -3.3647 | 7.66E-04 | GTExv8.EUR.Whole_Blood | 5.51E-03 | LLGL1 | BMI |
| -2.5122 | 1.20E-02 | GTExv8.EUR.Whole_Blood | 4.94E-02 | MIEF2 | BMI |
| -2.7121 | 6.69E-03 | GTExv8.EUR.Whole_Blood | 3.15E-02 | TVP23B | BMI |
| -2.8362 | 4.57E-03 | GTExv8.EUR.Whole_Blood | 2.34E-02 | FAM83G | BMI |
| -2.5427 | 1.10E-02 | GTExv8.EUR.Whole_Blood | 4.60E-02 | CTC-457L16.2 | BMI |
| 5.1577 | 2.50E-07 | GTExv8.EUR.Whole_Blood | 4.98E-06 | NATD1 | BMI |
| 3.2105 | 1.33E-03 | GTExv8.EUR.Whole_Blood | 8.70E-03 | C17orf51 | BMI |
| 2.9704 | 2.97E-03 | GTExv8.EUR.Whole_Blood | 1.64E-02 | TMEM97 | BMI |
| 3.0764 | 2.10E-03 | GTExv8.EUR.Whole_Blood | 1.25E-02 | POLDIP2 | BMI |
| 3.0262 | 2.48E-03 | GTExv8.EUR.Whole_Blood | 1.42E-02 | TMEM199 | BMI |
| 3.0779 | 2.08E-03 | GTExv8.EUR.Whole_Blood | 1.24E-02 | SARM1 | BMI |
| 4.1508 | 3.31E-05 | GTExv8.EUR.Whole_Blood | 3.72E-04 | ERAL1 | BMI |
| 3.9528 | 7.72E-05 | GTExv8.EUR.Whole_Blood | 7.73E-04 | TIAF1 | BMI |
| -3.4694 | 5.22E-04 | GTExv8.EUR.Whole_Blood | 3.99E-03 | MYO18A | BMI |
| 2.9388 | 3.29E-03 | GTExv8.EUR.Whole_Blood | 1.78E-02 | GIT1 | BMI |
| 4.6166 | 3.90E-06 | GTExv8.EUR.Whole_Blood | 5.73E-05 | CORO6 | BMI |
| -6.0789 | 1.21E-09 | GTExv8.EUR.Whole_Blood | 4.38E-08 | SSH2 | BMI |
| -6.0665 | 1.31E-09 | GTExv8.EUR.Whole_Blood | 4.68E-08 | BLMH | BMI |
| 5.0000 | 5.73E-07 | GTExv8.EUR.Whole_Blood | 1.02E-05 | GOSR1 | BMI |
| -3.7787 | 1.58E-04 | GTExv8.EUR.Whole_Blood | 1.45E-03 | EVI2A | BMI |
| 2.7656 | 5.68E-03 | GTExv8.EUR.Whole_Blood | 2.75E-02 | RAB11FIP4 | BMI |
| 4.0136 | 5.98E-05 | GTExv8.EUR.Whole_Blood | 6.21E-04 | RHOT1 | BMI |
| 2.7746 | 5.53E-03 | GTExv8.EUR.Whole_Blood | 2.69E-02 | C17orf75 | BMI |
| 2.8225 | 4.76E-03 | GTExv8.EUR.Whole_Blood | 2.41E-02 | CDK5R1 | BMI |
| 3.0604 | 2.21E-03 | GTExv8.EUR.Whole_Blood | 1.29E-02 | AC131056.3 | BMI |
| -9.0819 | 1.07E-19 | GTExv8.EUR.Whole_Blood | 1.34E-17 | ZNHIT3 | BMI |
| 9.0345 | 1.65E-19 | GTExv8.EUR.Whole_Blood | 1.98E-17 | MYO19 | BMI |
| -9.5644 | 1.13E-21 | GTExv8.EUR.Whole_Blood | 1.52E-19 | GGNBP2 | BMI |
| 8.9792 | 2.73E-19 | GTExv8.EUR.Whole_Blood | 3.17E-17 | DHRS11 | BMI |
| -2.5495 | 1.08E-02 | GTExv8.EUR.Whole_Blood | 4.56E-02 | ORMDL3 | BMI |
| 5.1113 | 3.20E-07 | GTExv8.EUR.Whole_Blood | 6.18E-06 | MED24 | BMI |
| -4.4886 | 7.17E-06 | GTExv8.EUR.Whole_Blood | 9.95E-05 | WIPF2 | BMI |
| 4.3145 | 1.60E-05 | GTExv8.EUR.Whole_Blood | 2.00E-04 | SMARCE1 | BMI |
| -2.9577 | 3.10E-03 | GTExv8.EUR.Whole_Blood | 1.70E-02 | RP11-458J1.1 | BMI |
| -3.6498 | 2.62E-04 | GTExv8.EUR.Whole_Blood | 2.23E-03 | HSD17B1 | BMI |
| 3.1214 | 1.80E-03 | GTExv8.EUR.Whole_Blood | 1.10E-02 | EZH1 | BMI |
| 3.7672 | 1.65E-04 | GTExv8.EUR.Whole_Blood | 1.50E-03 | BECN1 | BMI |
| 3.5678 | 3.60E-04 | GTExv8.EUR.Whole_Blood | 2.91E-03 | VAT1 | BMI |
| 3.4034 | 6.66E-04 | GTExv8.EUR.Whole_Blood | 4.90E-03 | NBR2 | BMI |
| 3.9981 | 6.38E-05 | GTExv8.EUR.Whole_Blood | 6.58E-04 | CTD-3199J23.6 | BMI |
| 4.0432 | 5.27E-05 | GTExv8.EUR.Whole_Blood | 5.58E-04 | NBR1 | BMI |
| 3.7456 | 1.80E-04 | GTExv8.EUR.Whole_Blood | 1.61E-03 | LINC00854 | BMI |
| -3.5186 | 4.34E-04 | GTExv8.EUR.Whole_Blood | 3.41E-03 | CTD-3014M21.1 | BMI |
| 5.1424 | 2.71E-07 | GTExv8.EUR.Whole_Blood | 5.33E-06 | ARL4D | BMI |
| 3.3139 | 9.20E-04 | GTExv8.EUR.Whole_Blood | 6.47E-03 | G6PC3 | BMI |
| -4.4061 | 1.05E-05 | GTExv8.EUR.Whole_Blood | 1.39E-04 | ASB16 | BMI |
| -5.1672 | 2.38E-07 | GTExv8.EUR.Whole_Blood | 4.80E-06 | ASB16-AS1 | BMI |
| -2.8404 | 4.51E-03 | GTExv8.EUR.Whole_Blood | 2.32E-02 | DBF4B | BMI |
| -4.7103 | 2.47E-06 | GTExv8.EUR.Whole_Blood | 3.81E-05 | ADAM11 | BMI |
| -3.0658 | 2.17E-03 | GTExv8.EUR.Whole_Blood | 1.28E-02 | RP11-798G7.6 | BMI |
| -2.7285 | 6.36E-03 | GTExv8.EUR.Whole_Blood | 3.03E-02 | KANSL1 | BMI |
| 3.8320 | 1.27E-04 | GTExv8.EUR.Whole_Blood | 1.21E-03 | RP11-156P1.3 | BMI |
| 4.8667 | 1.13E-06 | GTExv8.EUR.Whole_Blood | 1.88E-05 | NPEPPS | BMI |
| 3.2905 | 1.00E-03 | GTExv8.EUR.Whole_Blood | 6.86E-03 | OSBPL7 | BMI |
| 4.2057 | 2.60E-05 | GTExv8.EUR.Whole_Blood | 3.05E-04 | SCRN2 | BMI |
| 2.8254 | 4.72E-03 | GTExv8.EUR.Whole_Blood | 2.39E-02 | RP11-6N17.4 | BMI |
| -2.5758 | 1.00E-02 | GTExv8.EUR.Whole_Blood | 4.29E-02 | RP11-6N17.3 | BMI |
| -2.6129 | 8.98E-03 | GTExv8.EUR.Whole_Blood | 3.94E-02 | COPZ2 | BMI |
| 6.0134 | 1.82E-09 | GTExv8.EUR.Whole_Blood | 6.27E-08 | NFE2L1 | BMI |
| 4.4215 | 9.80E-06 | GTExv8.EUR.Whole_Blood | 1.32E-04 | SKAP1 | BMI |
| 5.4355 | 5.47E-08 | GTExv8.EUR.Whole_Blood | 1.31E-06 | HOXB-AS1 | BMI |
| 4.7943 | 1.63E-06 | GTExv8.EUR.Whole_Blood | 2.62E-05 | HOXB2 | BMI |
| 8.2704 | 1.33E-16 | GTExv8.EUR.Whole_Blood | 1.36E-14 | HOXB4 | BMI |
| 5.1987 | 2.01E-07 | GTExv8.EUR.Whole_Blood | 4.15E-06 | HOXB3 | BMI |
| -4.7483 | 2.05E-06 | GTExv8.EUR.Whole_Blood | 3.20E-05 | HOXB7 | BMI |
| -4.5084 | 6.53E-06 | GTExv8.EUR.Whole_Blood | 9.14E-05 | ATP5G1 | BMI |
| -5.5793 | 2.41E-08 | GTExv8.EUR.Whole_Blood | 6.40E-07 | UBE2Z | BMI |
| -3.8373 | 1.24E-04 | GTExv8.EUR.Whole_Blood | 1.18E-03 | RP11-81K2.1 | BMI |
| -3.4730 | 5.15E-04 | GTExv8.EUR.Whole_Blood | 3.94E-03 | DYNLL2 | BMI |
| 3.6458 | 2.67E-04 | GTExv8.EUR.Whole_Blood | 2.26E-03 | TSPOAP1 | BMI |
| 2.8260 | 4.71E-03 | GTExv8.EUR.Whole_Blood | 2.39E-02 | TEX14 | BMI |
| -3.7089 | 2.08E-04 | GTExv8.EUR.Whole_Blood | 1.83E-03 | RAD51C | BMI |
| 3.6836 | 2.30E-04 | GTExv8.EUR.Whole_Blood | 1.99E-03 | SMG8 | BMI |
| -2.6974 | 6.99E-03 | GTExv8.EUR.Whole_Blood | 3.25E-02 | NDUFB8P2 | BMI |
| 3.2505 | 1.15E-03 | GTExv8.EUR.Whole_Blood | 7.73E-03 | RPS6KB1 | BMI |
| -6.3360 | 2.36E-10 | GTExv8.EUR.Whole_Blood | 9.72E-09 | MAP3K3 | BMI |
| -6.8632 | 6.73E-12 | GTExv8.EUR.Whole_Blood | 3.91E-10 | EEF1DP7 | BMI |
| -7.1162 | 1.11E-12 | GTExv8.EUR.Whole_Blood | 7.92E-11 | LIMD2 | BMI |
| 5.2137 | 1.85E-07 | GTExv8.EUR.Whole_Blood | 3.85E-06 | RP11-51F16.1 | BMI |
| -6.8169 | 9.30E-12 | GTExv8.EUR.Whole_Blood | 5.36E-10 | STRADA | BMI |
| 4.7584 | 1.95E-06 | GTExv8.EUR.Whole_Blood | 3.06E-05 | DDX42 | BMI |
| -4.7081 | 2.50E-06 | GTExv8.EUR.Whole_Blood | 3.85E-05 | FTSJ3 | BMI |
| -6.9026 | 5.10E-12 | GTExv8.EUR.Whole_Blood | 3.03E-10 | SMARCD2 | BMI |
| -2.8029 | 5.06E-03 | GTExv8.EUR.Whole_Blood | 2.52E-02 | ERN1 | BMI |
| 2.7703 | 5.60E-03 | GTExv8.EUR.Whole_Blood | 2.72E-02 | CEP95 | BMI |
| -2.7703 | 5.60E-03 | GTExv8.EUR.Whole_Blood | 2.72E-02 | DDX5 | BMI |
| -2.9001 | 3.73E-03 | GTExv8.EUR.Whole_Blood | 1.97E-02 | PLEKHM1P1 | BMI |
| 2.7065 | 6.80E-03 | GTExv8.EUR.Whole_Blood | 3.18E-02 | RGS9 | BMI |
| -3.4284 | 6.07E-04 | GTExv8.EUR.Whole_Blood | 4.54E-03 | PITPNC1 | BMI |
| 6.4984 | 8.12E-11 | GTExv8.EUR.Whole_Blood | 3.90E-09 | LINC00674 | BMI |
| -2.5335 | 1.13E-02 | GTExv8.EUR.Whole_Blood | 4.71E-02 | RP11-147L13.15 | BMI |
| 2.5427 | 1.10E-02 | GTExv8.EUR.Whole_Blood | 4.60E-02 | MAP2K6 | BMI |
| 2.5427 | 1.10E-02 | GTExv8.EUR.Whole_Blood | 4.60E-02 | RP1-193H18.2 | BMI |
| -2.7822 | 5.40E-03 | GTExv8.EUR.Whole_Blood | 2.64E-02 | GGA3 | BMI |
| 2.6994 | 6.95E-03 | GTExv8.EUR.Whole_Blood | 3.24E-02 | MRPS7 | BMI |
| -5.1512 | 2.59E-07 | GTExv8.EUR.Whole_Blood | 5.13E-06 | GALK1 | BMI |
| -4.5383 | 5.67E-06 | GTExv8.EUR.Whole_Blood | 8.05E-05 | UNK | BMI |
| -5.2603 | 1.44E-07 | GTExv8.EUR.Whole_Blood | 3.10E-06 | UNC13D | BMI |
| 5.2296 | 1.70E-07 | GTExv8.EUR.Whole_Blood | 3.58E-06 | RP11-552F3.9 | BMI |
| -3.6081 | 3.08E-04 | GTExv8.EUR.Whole_Blood | 2.56E-03 | RP11-552F3.10 | BMI |
| -4.4607 | 8.17E-06 | GTExv8.EUR.Whole_Blood | 1.12E-04 | MRPL38 | BMI |
| -3.0899 | 2.00E-03 | GTExv8.EUR.Whole_Blood | 1.20E-02 | TEN1 | BMI |
| -2.8070 | 5.00E-03 | GTExv8.EUR.Whole_Blood | 2.49E-02 | DNAH17 | BMI |
| 2.5961 | 9.43E-03 | GTExv8.EUR.Whole_Blood | 4.09E-02 | LINC02081 | BMI |
| 4.7001 | 2.60E-06 | GTExv8.EUR.Whole_Blood | 3.99E-05 | CEP295NL | BMI |
| 4.7275 | 2.27E-06 | GTExv8.EUR.Whole_Blood | 3.53E-05 | CTD-2373H9.5 | BMI |
| -2.9248 | 3.45E-03 | GTExv8.EUR.Whole_Blood | 1.85E-02 | TBC1D16 | BMI |
| -2.8083 | 4.98E-03 | GTExv8.EUR.Whole_Blood | 2.49E-02 | ENDOV | BMI |
| 4.9543 | 7.26E-07 | GTExv8.EUR.Whole_Blood | 1.27E-05 | RPTOR | BMI |
| 3.5752 | 3.50E-04 | GTExv8.EUR.Whole_Blood | 2.84E-03 | AATK | BMI |
| -3.2687 | 1.08E-03 | GTExv8.EUR.Whole_Blood | 7.35E-03 | SLC38A10 | BMI |
| -2.5282 | 1.15E-02 | GTExv8.EUR.Whole_Blood | 4.78E-02 | RP11-1055B8.4 | BMI |
| -2.5695 | 1.02E-02 | GTExv8.EUR.Whole_Blood | 4.35E-02 | ARL16 | BMI |
| -3.5373 | 4.04E-04 | GTExv8.EUR.Whole_Blood | 3.21E-03 | CENPX | BMI |
| 2.6045 | 9.20E-03 | GTExv8.EUR.Whole_Blood | 4.01E-02 | DCXR | BMI |
| 5.0290 | 4.93E-07 | GTExv8.EUR.Whole_Blood | 8.90E-06 | FASN | BMI |
| -5.7623 | 8.30E-09 | GTExv8.EUR.Whole_Blood | 2.47E-07 | RP11-1376P16.2 | BMI |
| -5.9210 | 3.20E-09 | GTExv8.EUR.Whole_Blood | 1.05E-07 | CCDC57 | BMI |
| 6.5478 | 5.84E-11 | GTExv8.EUR.Whole_Blood | 2.91E-09 | RP13-516M14.2 | BMI |
| -5.0355 | 4.77E-07 | GTExv8.EUR.Whole_Blood | 8.71E-06 | SLC16A3 | BMI |
| 2.6908 | 7.13E-03 | GTExv8.EUR.Whole_Blood | 3.29E-02 | CSNK1D | BMI |
| 2.8260 | 4.71E-03 | GTExv8.EUR.Whole_Blood | 2.39E-02 | OGFOD3 | BMI |
| -2.7878 | 5.31E-03 | GTExv8.EUR.Whole_Blood | 2.61E-02 | HEXDC | BMI |
| 2.8135 | 4.90E-03 | GTExv8.EUR.Whole_Blood | 2.46E-02 | C17orf62 | BMI |
| 3.4935 | 4.77E-04 | GTExv8.EUR.Whole_Blood | 3.69E-03 | NAPG | BMI |
| -2.8820 | 3.95E-03 | GTExv8.EUR.Whole_Blood | 2.07E-02 | IMPA2 | BMI |
| -4.1449 | 3.40E-05 | GTExv8.EUR.Whole_Blood | 3.80E-04 | LINC01882 | BMI |
| -4.3496 | 1.36E-05 | GTExv8.EUR.Whole_Blood | 1.75E-04 | RP11-973H7.1 | BMI |
| -2.7212 | 6.50E-03 | GTExv8.EUR.Whole_Blood | 3.08E-02 | SEH1L | BMI |
| -2.9330 | 3.36E-03 | GTExv8.EUR.Whole_Blood | 1.81E-02 | CEP192 | BMI |
| 2.9070 | 3.65E-03 | GTExv8.EUR.Whole_Blood | 1.94E-02 | TMEM241 | BMI |
| -12.0399 | 2.19E-33 | GTExv8.EUR.Whole_Blood | 7.41E-31 | C18orf8 | BMI |
| -3.0757 | 2.10E-03 | GTExv8.EUR.Whole_Blood | 1.25E-02 | HRH4 | BMI |
| -3.1012 | 1.93E-03 | GTExv8.EUR.Whole_Blood | 1.17E-02 | RP11-178F10.2 | BMI |
| 2.8703 | 4.10E-03 | GTExv8.EUR.Whole_Blood | 2.13E-02 | INO80C | BMI |
| -6.2793 | 3.40E-10 | GTExv8.EUR.Whole_Blood | 1.36E-08 | PIK3C3 | BMI |
| -3.8447 | 1.21E-04 | GTExv8.EUR.Whole_Blood | 1.16E-03 | PIAS2 | BMI |
| -3.2961 | 9.81E-04 | GTExv8.EUR.Whole_Blood | 6.78E-03 | RP11-426J5.3 | BMI |
| -2.9953 | 2.74E-03 | GTExv8.EUR.Whole_Blood | 1.54E-02 | DYM | BMI |
| 2.5485 | 1.08E-02 | GTExv8.EUR.Whole_Blood | 4.56E-02 | RP11-850A17.1 | BMI |
| 3.3013 | 9.62E-04 | GTExv8.EUR.Whole_Blood | 6.70E-03 | POLI | BMI |
| -3.1370 | 1.71E-03 | GTExv8.EUR.Whole_Blood | 1.06E-02 | C18orf54 | BMI |
| -5.1331 | 2.85E-07 | GTExv8.EUR.Whole_Blood | 5.56E-06 | ZNF532 | BMI |
| -4.0837 | 4.43E-05 | GTExv8.EUR.Whole_Blood | 4.81E-04 | PIGN | BMI |
| 2.7906 | 5.26E-03 | GTExv8.EUR.Whole_Blood | 2.59E-02 | ZCCHC2 | BMI |
| -2.8526 | 4.34E-03 | GTExv8.EUR.Whole_Blood | 2.25E-02 | TMX3 | BMI |
| -2.5620 | 1.04E-02 | GTExv8.EUR.Whole_Blood | 4.42E-02 | ZADH2 | BMI |
| 2.8645 | 4.18E-03 | GTExv8.EUR.Whole_Blood | 2.17E-02 | MIER2 | BMI |
| 3.0968 | 1.96E-03 | GTExv8.EUR.Whole_Blood | 1.18E-02 | MED16 | BMI |
| 3.0599 | 2.21E-03 | GTExv8.EUR.Whole_Blood | 1.29E-02 | KISS1R | BMI |
| 6.5415 | 6.09E-11 | GTExv8.EUR.Whole_Blood | 3.02E-09 | CSNK1G2 | BMI |
| -4.9491 | 7.45E-07 | GTExv8.EUR.Whole_Blood | 1.29E-05 | AC005306.3 | BMI |
| -3.3911 | 6.96E-04 | GTExv8.EUR.Whole_Blood | 5.08E-03 | DIRAS1 | BMI |
| -3.1274 | 1.76E-03 | GTExv8.EUR.Whole_Blood | 1.09E-02 | AC006538.1 | BMI |
| -3.2384 | 1.20E-03 | GTExv8.EUR.Whole_Blood | 8.00E-03 | SLC39A3 | BMI |
| -2.8071 | 5.00E-03 | GTExv8.EUR.Whole_Blood | 2.49E-02 | ZNF554 | BMI |
| -3.1467 | 1.65E-03 | GTExv8.EUR.Whole_Blood | 1.03E-02 | PIP5K1C | BMI |
| -2.9889 | 2.80E-03 | GTExv8.EUR.Whole_Blood | 1.56E-02 | ZFR2 | BMI |
| -8.9063 | 5.28E-19 | GTExv8.EUR.Whole_Blood | 5.95E-17 | ZBTB7A | BMI |
| 4.3467 | 1.38E-05 | GTExv8.EUR.Whole_Blood | 1.77E-04 | ANKRD24 | BMI |
| 5.8299 | 5.55E-09 | GTExv8.EUR.Whole_Blood | 1.71E-07 | MPND | BMI |
| -4.0254 | 5.69E-05 | GTExv8.EUR.Whole_Blood | 5.98E-04 | CTB-50L17.9 | BMI |
| -5.1733 | 2.30E-07 | GTExv8.EUR.Whole_Blood | 4.65E-06 | UBXN6 | BMI |
| 2.9366 | 3.32E-03 | GTExv8.EUR.Whole_Blood | 1.79E-02 | FEM1A | BMI |
| -3.7623 | 1.68E-04 | GTExv8.EUR.Whole_Blood | 1.53E-03 | KDM4B | BMI |
| 3.2765 | 1.05E-03 | GTExv8.EUR.Whole_Blood | 7.16E-03 | PET100 | BMI |
| 2.9723 | 2.96E-03 | GTExv8.EUR.Whole_Blood | 1.64E-02 | EVI5L | BMI |
| 2.6741 | 7.49E-03 | GTExv8.EUR.Whole_Blood | 3.41E-02 | CD320 | BMI |
| -2.5427 | 1.10E-02 | GTExv8.EUR.Whole_Blood | 4.60E-02 | ZNF317 | BMI |
| 3.9108 | 9.20E-05 | GTExv8.EUR.Whole_Blood | 9.06E-04 | ZNF699 | BMI |
| 3.3663 | 7.62E-04 | GTExv8.EUR.Whole_Blood | 5.49E-03 | ZNF266 | BMI |
| -4.3072 | 1.65E-05 | GTExv8.EUR.Whole_Blood | 2.05E-04 | CTC-543D15.8 | BMI |
| 2.7662 | 5.67E-03 | GTExv8.EUR.Whole_Blood | 2.75E-02 | ZNF121 | BMI |
| 3.0215 | 2.52E-03 | GTExv8.EUR.Whole_Blood | 1.44E-02 | ZNF561 | BMI |
| 3.0279 | 2.46E-03 | GTExv8.EUR.Whole_Blood | 1.41E-02 | ZNF812P | BMI |
| 2.9978 | 2.72E-03 | GTExv8.EUR.Whole_Blood | 1.53E-02 | CTD-3116E22.8 | BMI |
| 4.2228 | 2.41E-05 | GTExv8.EUR.Whole_Blood | 2.86E-04 | FBXL12 | BMI |
| 2.6343 | 8.43E-03 | GTExv8.EUR.Whole_Blood | 3.75E-02 | C19orf66 | BMI |
| 3.6721 | 2.41E-04 | GTExv8.EUR.Whole_Blood | 2.07E-03 | ANGPTL6 | BMI |
| 2.7016 | 6.90E-03 | GTExv8.EUR.Whole_Blood | 3.22E-02 | PPAN | BMI |
| 3.0311 | 2.44E-03 | GTExv8.EUR.Whole_Blood | 1.40E-02 | EIF3G | BMI |
| 4.9113 | 9.05E-07 | GTExv8.EUR.Whole_Blood | 1.54E-05 | ICAM1 | BMI |
| 3.1603 | 1.58E-03 | GTExv8.EUR.Whole_Blood | 9.96E-03 | TYK2 | BMI |
| -4.6860 | 2.79E-06 | GTExv8.EUR.Whole_Blood | 4.27E-05 | TMED1 | BMI |
| -2.9814 | 2.87E-03 | GTExv8.EUR.Whole_Blood | 1.60E-02 | ZNF490 | BMI |
| -3.2271 | 1.25E-03 | GTExv8.EUR.Whole_Blood | 8.29E-03 | WDR83OS | BMI |
| -4.6687 | 3.03E-06 | GTExv8.EUR.Whole_Blood | 4.61E-05 | PRDX2 | BMI |
| 4.6114 | 4.00E-06 | GTExv8.EUR.Whole_Blood | 5.85E-05 | HOOK2 | BMI |
| -6.3862 | 1.70E-10 | GTExv8.EUR.Whole_Blood | 7.31E-09 | DNASE2 | BMI |
| -2.6641 | 7.72E-03 | GTExv8.EUR.Whole_Blood | 3.49E-02 | FARSA | BMI |
| 2.8475 | 4.41E-03 | GTExv8.EUR.Whole_Blood | 2.27E-02 | CTB-55O6.12 | BMI |
| 2.7381 | 6.18E-03 | GTExv8.EUR.Whole_Blood | 2.95E-02 | F2RL3 | BMI |
| 2.7150 | 6.63E-03 | GTExv8.EUR.Whole_Blood | 3.13E-02 | CPAMD8 | BMI |
| 3.4197 | 6.27E-04 | GTExv8.EUR.Whole_Blood | 4.66E-03 | BABAM1 | BMI |
| 2.6080 | 9.11E-03 | GTExv8.EUR.Whole_Blood | 3.98E-02 | INSL3 | BMI |
| -8.6178 | 6.83E-18 | GTExv8.EUR.Whole_Blood | 7.49E-16 | MAST3 | BMI |
| -3.6949 | 2.20E-04 | GTExv8.EUR.Whole_Blood | 1.92E-03 | IL12RB1 | BMI |
| 3.7911 | 1.50E-04 | GTExv8.EUR.Whole_Blood | 1.39E-03 | MPV17L2 | BMI |
| -3.4614 | 5.37E-04 | GTExv8.EUR.Whole_Blood | 4.08E-03 | GDF15 | BMI |
| 2.8361 | 4.57E-03 | GTExv8.EUR.Whole_Blood | 2.34E-02 | RFXANK | BMI |
| -3.8937 | 9.87E-05 | GTExv8.EUR.Whole_Blood | 9.63E-04 | GATAD2A | BMI |
| -7.6506 | 2.00E-14 | GTExv8.EUR.Whole_Blood | 1.75E-12 | TSSK6 | BMI |
| -3.3394 | 8.40E-04 | GTExv8.EUR.Whole_Blood | 5.99E-03 | NDUFA13 | BMI |
| 4.0980 | 4.17E-05 | GTExv8.EUR.Whole_Blood | 4.57E-04 | YJEFN3 | BMI |
| -4.1449 | 3.40E-05 | GTExv8.EUR.Whole_Blood | 3.80E-04 | PBX4 | BMI |
| 3.3973 | 6.81E-04 | GTExv8.EUR.Whole_Blood | 4.99E-03 | LPAR2 | BMI |
| 3.2791 | 1.04E-03 | GTExv8.EUR.Whole_Blood | 7.10E-03 | GMIP | BMI |
| -3.2537 | 1.14E-03 | GTExv8.EUR.Whole_Blood | 7.67E-03 | ATP13A1 | BMI |
| 6.1127 | 9.80E-10 | GTExv8.EUR.Whole_Blood | 3.58E-08 | ZNF101 | BMI |
| 3.4227 | 6.20E-04 | GTExv8.EUR.Whole_Blood | 4.61E-03 | ZNF14 | BMI |
| -2.5612 | 1.04E-02 | GTExv8.EUR.Whole_Blood | 4.42E-02 | RP11-420K14.6 | BMI |
| -2.7256 | 6.42E-03 | GTExv8.EUR.Whole_Blood | 3.05E-02 | ZNF100 | BMI |
| 4.6010 | 4.21E-06 | GTExv8.EUR.Whole_Blood | 6.14E-05 | AC007773.2 | BMI |
| 2.7944 | 5.20E-03 | GTExv8.EUR.Whole_Blood | 2.57E-02 | CHST8 | BMI |
| 3.4949 | 4.74E-04 | GTExv8.EUR.Whole_Blood | 3.67E-03 | GRAMD1A | BMI |
| 3.7750 | 1.60E-04 | GTExv8.EUR.Whole_Blood | 1.46E-03 | HPN | BMI |
| 2.6630 | 7.74E-03 | GTExv8.EUR.Whole_Blood | 3.50E-02 | CAPNS1 | BMI |
| 2.6050 | 9.19E-03 | GTExv8.EUR.Whole_Blood | 4.01E-02 | ZNF529 | BMI |
| 2.7935 | 5.21E-03 | GTExv8.EUR.Whole_Blood | 2.58E-02 | PPP1R14A | BMI |
| -2.8327 | 4.62E-03 | GTExv8.EUR.Whole_Blood | 2.35E-02 | CATSPERG | BMI |
| -2.5305 | 1.14E-02 | GTExv8.EUR.Whole_Blood | 4.75E-02 | ZNF780A | BMI |
| 3.1280 | 1.76E-03 | GTExv8.EUR.Whole_Blood | 1.09E-02 | C19orf47 | BMI |
| 3.2616 | 1.11E-03 | GTExv8.EUR.Whole_Blood | 7.52E-03 | EGLN2 | BMI |
| -3.0431 | 2.34E-03 | GTExv8.EUR.Whole_Blood | 1.36E-02 | CYP2T1P | BMI |
| 5.3036 | 1.14E-07 | GTExv8.EUR.Whole_Blood | 2.53E-06 | ZNF574 | BMI |
| 3.6529 | 2.59E-04 | GTExv8.EUR.Whole_Blood | 2.20E-03 | DEDD2 | BMI |
| 2.6486 | 8.08E-03 | GTExv8.EUR.Whole_Blood | 3.62E-02 | LIPE | BMI |
| 2.7865 | 5.33E-03 | GTExv8.EUR.Whole_Blood | 2.62E-02 | ZNF155 | BMI |
| 3.2999 | 9.67E-04 | GTExv8.EUR.Whole_Blood | 6.72E-03 | PPP1R37 | BMI |
| -3.1378 | 1.70E-03 | GTExv8.EUR.Whole_Blood | 1.05E-02 | BLOC1S3 | BMI |
| 3.7067 | 2.10E-04 | GTExv8.EUR.Whole_Blood | 1.84E-03 | PPM1N | BMI |
| 4.5918 | 4.39E-06 | GTExv8.EUR.Whole_Blood | 6.36E-05 | CCDC61 | BMI |
| -2.9478 | 3.20E-03 | GTExv8.EUR.Whole_Blood | 1.74E-02 | AC007193.9 | BMI |
| 3.8265 | 1.30E-04 | GTExv8.EUR.Whole_Blood | 1.23E-03 | PNMAL1 | BMI |
| 3.5553 | 3.78E-04 | GTExv8.EUR.Whole_Blood | 3.03E-03 | PNMAL2 | BMI |
| -7.5081 | 6.00E-14 | GTExv8.EUR.Whole_Blood | 5.19E-12 | SAE1 | BMI |
| 4.0780 | 4.54E-05 | GTExv8.EUR.Whole_Blood | 4.90E-04 | KPTN | BMI |
| -2.8413 | 4.49E-03 | GTExv8.EUR.Whole_Blood | 2.31E-02 | GRWD1 | BMI |
| -3.0211 | 2.52E-03 | GTExv8.EUR.Whole_Blood | 1.44E-02 | CYTH2 | BMI |
| -2.5673 | 1.03E-02 | GTExv8.EUR.Whole_Blood | 4.39E-02 | NTN5 | BMI |
| 2.6452 | 8.16E-03 | GTExv8.EUR.Whole_Blood | 3.65E-02 | BCAT2 | BMI |
| -2.9050 | 3.67E-03 | GTExv8.EUR.Whole_Blood | 1.95E-02 | PRMT1 | BMI |
| -3.2979 | 9.74E-04 | GTExv8.EUR.Whole_Blood | 6.74E-03 | SIGLEC11 | BMI |
| -2.9994 | 2.70E-03 | GTExv8.EUR.Whole_Blood | 1.52E-02 | SIGLEC16 | BMI |
| -2.6828 | 7.30E-03 | GTExv8.EUR.Whole_Blood | 3.34E-02 | ZNF473 | BMI |
| -2.7944 | 5.20E-03 | GTExv8.EUR.Whole_Blood | 2.57E-02 | EMC10 | BMI |
| -2.7746 | 5.53E-03 | GTExv8.EUR.Whole_Blood | 2.69E-02 | JOSD2 | BMI |
| -3.0357 | 2.40E-03 | GTExv8.EUR.Whole_Blood | 1.38E-02 | SIGLEC22P | BMI |
| 2.6304 | 8.53E-03 | GTExv8.EUR.Whole_Blood | 3.78E-02 | ZNF613 | BMI |
| 2.5121 | 1.20E-02 | GTExv8.EUR.Whole_Blood | 4.94E-02 | KIR2DL1 | BMI |
| 2.6209 | 8.77E-03 | GTExv8.EUR.Whole_Blood | 3.87E-02 | ZNF579 | BMI |
| 3.5319 | 4.13E-04 | GTExv8.EUR.Whole_Blood | 3.28E-03 | A1BG-AS1 | BMI |
| 4.2114 | 2.54E-05 | GTExv8.EUR.Whole_Blood | 2.99E-04 | A1BG | BMI |
| 2.5124 | 1.20E-02 | GTExv8.EUR.Whole_Blood | 4.94E-02 | PSMF1 | BMI |
| -2.5541 | 1.06E-02 | GTExv8.EUR.Whole_Blood | 4.50E-02 | FKBP1A | BMI |
| -5.8895 | 3.87E-09 | GTExv8.EUR.Whole_Blood | 1.25E-07 | PCED1A | BMI |
| 4.8729 | 1.10E-06 | GTExv8.EUR.Whole_Blood | 1.84E-05 | PTPRA | BMI |
| -3.4842 | 4.94E-04 | GTExv8.EUR.Whole_Blood | 3.79E-03 | LZTS3 | BMI |
| 3.2697 | 1.08E-03 | GTExv8.EUR.Whole_Blood | 7.35E-03 | CDC25B | BMI |
| -3.1969 | 1.39E-03 | GTExv8.EUR.Whole_Blood | 9.01E-03 | RNF24 | BMI |
| 4.3776 | 1.20E-05 | GTExv8.EUR.Whole_Blood | 1.57E-04 | PLCB1 | BMI |
| -3.6824 | 2.31E-04 | GTExv8.EUR.Whole_Blood | 2.00E-03 | LAMP5 | BMI |
| 2.7396 | 6.15E-03 | GTExv8.EUR.Whole_Blood | 2.94E-02 | KIZ | BMI |
| -5.4353 | 5.47E-08 | GTExv8.EUR.Whole_Blood | 1.31E-06 | RP4-568C11.4 | BMI |
| -5.8920 | 3.82E-09 | GTExv8.EUR.Whole_Blood | 1.24E-07 | CST7 | BMI |
| -3.0940 | 1.97E-03 | GTExv8.EUR.Whole_Blood | 1.19E-02 | APMAP | BMI |
| 4.0607 | 4.89E-05 | GTExv8.EUR.Whole_Blood | 5.23E-04 | ACSS1 | BMI |
| 4.8763 | 1.08E-06 | GTExv8.EUR.Whole_Blood | 1.81E-05 | ENTPD6 | BMI |
| 6.0456 | 1.49E-09 | GTExv8.EUR.Whole_Blood | 5.22E-08 | RP5-965G21.3 | BMI |
| -5.6861 | 1.30E-08 | GTExv8.EUR.Whole_Blood | 3.65E-07 | RP5-965G21.4 | BMI |
| 5.1059 | 3.29E-07 | GTExv8.EUR.Whole_Blood | 6.32E-06 | PYGB | BMI |
| 6.1176 | 9.50E-10 | GTExv8.EUR.Whole_Blood | 3.50E-08 | NINL | BMI |
| -4.2286 | 2.35E-05 | GTExv8.EUR.Whole_Blood | 2.80E-04 | NOL4L | BMI |
| -3.1416 | 1.68E-03 | GTExv8.EUR.Whole_Blood | 1.05E-02 | RP5-1085F17.3 | BMI |
| -7.3535 | 1.93E-13 | GTExv8.EUR.Whole_Blood | 1.60E-11 | RP5-1125A11.7 | BMI |
| 5.5107 | 3.57E-08 | GTExv8.EUR.Whole_Blood | 8.99E-07 | MAP1LC3A | BMI |
| 3.5368 | 4.05E-04 | GTExv8.EUR.Whole_Blood | 3.22E-03 | GGT7 | BMI |
| 3.2337 | 1.22E-03 | GTExv8.EUR.Whole_Blood | 8.11E-03 | MYH7B | BMI |
| -4.5328 | 5.82E-06 | GTExv8.EUR.Whole_Blood | 8.23E-05 | EDEM2 | BMI |
| -3.8714 | 1.08E-04 | GTExv8.EUR.Whole_Blood | 1.05E-03 | EIF6 | BMI |
| -3.3994 | 6.75E-04 | GTExv8.EUR.Whole_Blood | 4.95E-03 | FER1L4 | BMI |
| -2.5576 | 1.05E-02 | GTExv8.EUR.Whole_Blood | 4.46E-02 | AAR2 | BMI |
| -2.5453 | 1.09E-02 | GTExv8.EUR.Whole_Blood | 4.58E-02 | RBL1 | BMI |
| -3.2160 | 1.30E-03 | GTExv8.EUR.Whole_Blood | 8.56E-03 | FAM83D | BMI |
| -2.8205 | 4.79E-03 | GTExv8.EUR.Whole_Blood | 2.42E-02 | OSER1 | BMI |
| -4.8916 | 1.00E-06 | GTExv8.EUR.Whole_Blood | 1.68E-05 | PKIG | BMI |
| 2.6919 | 7.10E-03 | GTExv8.EUR.Whole_Blood | 3.28E-02 | RP11-445H22.3 | BMI |
| -5.2980 | 1.17E-07 | GTExv8.EUR.Whole_Blood | 2.59E-06 | YWHAB | BMI |
| 5.3412 | 9.23E-08 | GTExv8.EUR.Whole_Blood | 2.09E-06 | PABPC1L | BMI |
| -2.8202 | 4.80E-03 | GTExv8.EUR.Whole_Blood | 2.42E-02 | PLTP | BMI |
| -3.7750 | 1.60E-04 | GTExv8.EUR.Whole_Blood | 1.46E-03 | PCIF1 | BMI |
| -2.5758 | 1.00E-02 | GTExv8.EUR.Whole_Blood | 4.29E-02 | ZNF335 | BMI |
| 3.8042 | 1.42E-04 | GTExv8.EUR.Whole_Blood | 1.32E-03 | RP11-465L10.10 | BMI |
| 4.7472 | 2.06E-06 | GTExv8.EUR.Whole_Blood | 3.21E-05 | CTD-2653D5.1 | BMI |
| -3.6080 | 3.09E-04 | GTExv8.EUR.Whole_Blood | 2.56E-03 | LINC00494 | BMI |
| 4.8085 | 1.52E-06 | GTExv8.EUR.Whole_Blood | 2.46E-05 | ARFGEF2 | BMI |
| 3.1256 | 1.77E-03 | GTExv8.EUR.Whole_Blood | 1.09E-02 | STAU1 | BMI |
| 2.7114 | 6.70E-03 | GTExv8.EUR.Whole_Blood | 3.15E-02 | ZNFX1 | BMI |
| 4.5534 | 5.28E-06 | GTExv8.EUR.Whole_Blood | 7.54E-05 | ZFAS1 | BMI |
| 3.3516 | 8.03E-04 | GTExv8.EUR.Whole_Blood | 5.75E-03 | SNORD12B | BMI |
| 4.6486 | 3.34E-06 | GTExv8.EUR.Whole_Blood | 5.02E-05 | RP4-791K14.2 | BMI |
| 5.9428 | 2.80E-09 | GTExv8.EUR.Whole_Blood | 9.35E-08 | KCNB1 | BMI |
| -3.8082 | 1.40E-04 | GTExv8.EUR.Whole_Blood | 1.31E-03 | B4GALT5 | BMI |
| 5.6858 | 1.30E-08 | GTExv8.EUR.Whole_Blood | 3.65E-07 | TSHZ2 | BMI |
| 5.5460 | 2.92E-08 | GTExv8.EUR.Whole_Blood | 7.52E-07 | RP4-563E14.1 | BMI |
| 6.6164 | 3.68E-11 | GTExv8.EUR.Whole_Blood | 1.91E-09 | DIDO1 | BMI |
| -3.6270 | 2.87E-04 | GTExv8.EUR.Whole_Blood | 2.41E-03 | SLC17A9 | BMI |
| -2.8983 | 3.75E-03 | GTExv8.EUR.Whole_Blood | 1.98E-02 | BIRC7 | BMI |
| -2.8748 | 4.04E-03 | GTExv8.EUR.Whole_Blood | 2.10E-02 | GMEB2 | BMI |
| 3.1421 | 1.68E-03 | GTExv8.EUR.Whole_Blood | 1.05E-02 | STMN3 | BMI |
| -4.1526 | 3.29E-05 | GTExv8.EUR.Whole_Blood | 3.72E-04 | ARFRP1 | BMI |
| -3.0902 | 2.00E-03 | GTExv8.EUR.Whole_Blood | 1.20E-02 | LIME1 | BMI |
| 2.7183 | 6.56E-03 | GTExv8.EUR.Whole_Blood | 3.10E-02 | TCEA2 | BMI |
| -4.7623 | 1.91E-06 | GTExv8.EUR.Whole_Blood | 3.01E-05 | OPRL1 | BMI |
| -3.5246 | 4.24E-04 | GTExv8.EUR.Whole_Blood | 3.35E-03 | RWDD2B | BMI |
| -2.9609 | 3.07E-03 | GTExv8.EUR.Whole_Blood | 1.69E-02 | IFNAR1 | BMI |
| -4.5819 | 4.61E-06 | GTExv8.EUR.Whole_Blood | 6.66E-05 | BRWD1-AS2 | BMI |
| -3.8526 | 1.17E-04 | GTExv8.EUR.Whole_Blood | 1.12E-03 | BRWD1 | BMI |
| -6.4352 | 1.23E-10 | GTExv8.EUR.Whole_Blood | 5.53E-09 | HMGN1 | BMI |
| -2.6197 | 8.80E-03 | GTExv8.EUR.Whole_Blood | 3.87E-02 | WRB | BMI |
| -2.6197 | 8.80E-03 | GTExv8.EUR.Whole_Blood | 3.87E-02 | LCA5L | BMI |
| -2.8189 | 4.82E-03 | GTExv8.EUR.Whole_Blood | 2.43E-02 | PRDM15 | BMI |
| -3.0608 | 2.21E-03 | GTExv8.EUR.Whole_Blood | 1.29E-02 | LINC01678 | BMI |
| -3.3206 | 8.98E-04 | GTExv8.EUR.Whole_Blood | 6.34E-03 | AP001056.1 | BMI |
| -4.1735 | 3.00E-05 | GTExv8.EUR.Whole_Blood | 3.45E-04 | SSR4P1 | BMI |
| -5.0259 | 5.01E-07 | GTExv8.EUR.Whole_Blood | 9.00E-06 | LINC00205 | BMI |
| 2.9479 | 3.20E-03 | GTExv8.EUR.Whole_Blood | 1.74E-02 | COL6A2 | BMI |
| 2.9706 | 2.97E-03 | GTExv8.EUR.Whole_Blood | 1.64E-02 | SPATC1L | BMI |
| 3.0517 | 2.28E-03 | GTExv8.EUR.Whole_Blood | 1.33E-02 | S100B | BMI |
| 2.5570 | 1.06E-02 | GTExv8.EUR.Whole_Blood | 4.50E-02 | PRMT2 | BMI |
| 3.3107 | 9.31E-04 | GTExv8.EUR.Whole_Blood | 6.53E-03 | DSTNP1 | BMI |
| -3.2160 | 1.30E-03 | GTExv8.EUR.Whole_Blood | 8.56E-03 | ATP6V1E1 | BMI |
| 2.5184 | 1.18E-02 | GTExv8.EUR.Whole_Blood | 4.89E-02 | BCL2L13 | BMI |
| 3.1745 | 1.50E-03 | GTExv8.EUR.Whole_Blood | 9.54E-03 | ARVCF | BMI |
| 4.6470 | 3.37E-06 | GTExv8.EUR.Whole_Blood | 5.05E-05 | TANGO2 | BMI |
| -2.8367 | 4.56E-03 | GTExv8.EUR.Whole_Blood | 2.34E-02 | DGCR8 | BMI |
| -4.9155 | 8.86E-07 | GTExv8.EUR.Whole_Blood | 1.52E-05 | YDJC | BMI |
| -2.5526 | 1.07E-02 | GTExv8.EUR.Whole_Blood | 4.53E-02 | PPIL2 | BMI |
| -2.6236 | 8.70E-03 | GTExv8.EUR.Whole_Blood | 3.84E-02 | FBXW4P1 | BMI |
| -2.5342 | 1.13E-02 | GTExv8.EUR.Whole_Blood | 4.71E-02 | VPREB3 | BMI |
| 4.2763 | 1.90E-05 | GTExv8.EUR.Whole_Blood | 2.32E-04 | ASPHD2 | BMI |
| 2.8449 | 4.44E-03 | GTExv8.EUR.Whole_Blood | 2.29E-02 | TFIP11 | BMI |
| 2.8164 | 4.86E-03 | GTExv8.EUR.Whole_Blood | 2.44E-02 | LINC01422 | BMI |
| 2.7269 | 6.39E-03 | GTExv8.EUR.Whole_Blood | 3.04E-02 | MN1 | BMI |
| 3.6560 | 2.56E-04 | GTExv8.EUR.Whole_Blood | 2.18E-03 | RNF185 | BMI |
| -3.9953 | 6.46E-05 | GTExv8.EUR.Whole_Blood | 6.65E-04 | PIK3IP1 | BMI |
| -4.4095 | 1.04E-05 | GTExv8.EUR.Whole_Blood | 1.38E-04 | SFI1 | BMI |
| -4.3439 | 1.40E-05 | GTExv8.EUR.Whole_Blood | 1.79E-04 | EIF4ENIF1 | BMI |
| -5.3086 | 1.10E-07 | GTExv8.EUR.Whole_Blood | 2.47E-06 | PISD | BMI |
| -5.3499 | 8.80E-08 | GTExv8.EUR.Whole_Blood | 2.01E-06 | DEPDC5 | BMI |
| -5.5414 | 3.00E-08 | GTExv8.EUR.Whole_Blood | 7.68E-07 | IFT27 | BMI |
| -3.9373 | 8.24E-05 | GTExv8.EUR.Whole_Blood | 8.20E-04 | C1QTNF6 | BMI |
| 3.4295 | 6.05E-04 | GTExv8.EUR.Whole_Blood | 4.53E-03 | GGA1 | BMI |
| 4.4162 | 1.00E-05 | GTExv8.EUR.Whole_Blood | 1.33E-04 | SH3BP1 | BMI |
| 4.3115 | 1.62E-05 | GTExv8.EUR.Whole_Blood | 2.02E-04 | Z83844.1 | BMI |
| -2.7468 | 6.02E-03 | GTExv8.EUR.Whole_Blood | 2.89E-02 | TRIOBP | BMI |
| 4.8250 | 1.40E-06 | GTExv8.EUR.Whole_Blood | 2.29E-05 | H1F0 | BMI |
| -4.7994 | 1.59E-06 | GTExv8.EUR.Whole_Blood | 2.57E-05 | ANKRD54 | BMI |
| 4.1543 | 3.26E-05 | GTExv8.EUR.Whole_Blood | 3.69E-04 | MAFF | BMI |
| 4.0652 | 4.80E-05 | GTExv8.EUR.Whole_Blood | 5.14E-04 | PLA2G6 | BMI |
| 3.3781 | 7.30E-04 | GTExv8.EUR.Whole_Blood | 5.28E-03 | NPTXR | BMI |
| 3.6165 | 2.99E-04 | GTExv8.EUR.Whole_Blood | 2.49E-03 | SYNGR1 | BMI |
| -6.1941 | 5.86E-10 | GTExv8.EUR.Whole_Blood | 2.27E-08 | ADSL | BMI |
| 8.1825 | 2.78E-16 | GTExv8.EUR.Whole_Blood | 2.81E-14 | MKL1 | BMI |
| 4.6145 | 3.94E-06 | GTExv8.EUR.Whole_Blood | 5.77E-05 | MCHR1 | BMI |
| -5.1558 | 2.53E-07 | GTExv8.EUR.Whole_Blood | 5.02E-06 | EP300 | BMI |
| 5.2295 | 1.70E-07 | GTExv8.EUR.Whole_Blood | 3.58E-06 | L3MBTL2 | BMI |
| 5.6317 | 1.78E-08 | GTExv8.EUR.Whole_Blood | 4.88E-07 | RP4-756G23.5 | BMI |
| -4.5963 | 4.30E-06 | GTExv8.EUR.Whole_Blood | 6.25E-05 | RANGAP1 | BMI |
| -2.5718 | 1.01E-02 | GTExv8.EUR.Whole_Blood | 4.32E-02 | PMM1 | BMI |
| 4.3085 | 1.64E-05 | GTExv8.EUR.Whole_Blood | 2.05E-04 | DESI1 | BMI |
| 3.3327 | 8.60E-04 | GTExv8.EUR.Whole_Blood | 6.11E-03 | MEI1 | BMI |
| 4.3178 | 1.58E-05 | GTExv8.EUR.Whole_Blood | 1.99E-04 | CCDC134 | BMI |
| 4.4554 | 8.37E-06 | GTExv8.EUR.Whole_Blood | 1.14E-04 | RP5-821D11.7 | BMI |
| 4.2415 | 2.22E-05 | GTExv8.EUR.Whole_Blood | 2.66E-04 | CENPM | BMI |
| 3.3888 | 7.02E-04 | GTExv8.EUR.Whole_Blood | 5.12E-03 | RP1-257I20.14 | BMI |
| 2.7068 | 6.79E-03 | GTExv8.EUR.Whole_Blood | 3.18E-02 | NDUFA6 | BMI |
| -2.7559 | 5.85E-03 | GTExv8.EUR.Whole_Blood | 2.83E-02 | CYP2D6 | BMI |
| 4.8546 | 1.21E-06 | GTExv8.EUR.Whole_Blood | 2.00E-05 | OGFRP1 | BMI |
| -3.9118 | 9.16E-05 | GTExv8.EUR.Whole_Blood | 9.03E-04 | TCF20 | BMI |
| -3.2406 | 1.19E-03 | GTExv8.EUR.Whole_Blood | 7.96E-03 | RRP7A | BMI |
| -3.2632 | 1.10E-03 | GTExv8.EUR.Whole_Blood | 7.46E-03 | RP1-47A17.1 | BMI |
| -2.7519 | 5.93E-03 | GTExv8.EUR.Whole_Blood | 2.86E-02 | ARFGAP3 | BMI |
| 3.3813 | 7.22E-04 | GTExv8.EUR.Whole_Blood | 5.24E-03 | TSPO | BMI |
| -3.1054 | 1.90E-03 | GTExv8.EUR.Whole_Blood | 1.15E-02 | RP4-671O14.5 | BMI |
| 3.1677 | 1.54E-03 | GTExv8.EUR.Whole_Blood | 9.76E-03 | RIBC2 | BMI |
| -3.0569 | 2.24E-03 | GTExv8.EUR.Whole_Blood | 1.31E-02 | TTC38 | BMI |
| 3.4210 | 6.24E-04 | GTExv8.EUR.Whole_Blood | 4.64E-03 | TRMU | BMI |
| -4.1434 | 3.42E-05 | GTExv8.EUR.Whole_Blood | 3.82E-04 | SELENOO | BMI |
| 6.9212 | 4.48E-12 | GTExv8.EUR.Whole_Blood | 2.70E-10 | RP3-402G11.28 | BMI |
| -3.5930 | 3.27E-04 | GTExv8.EUR.Whole_Blood | 2.68E-03 | TUBGCP6 | BMI |
| 2.8805 | 3.97E-03 | GTExv8.EUR.Whole_Blood | 2.08E-02 | CTA-384D8.36 | BMI |
| 4.1901 | 2.79E-05 | GTExv8.EUR.Whole_Blood | 3.24E-04 | TYMP | BMI |
| 3.6608 | 2.51E-04 | GTExv8.EUR.Whole_Blood | 2.14E-03 | ODF3B | BMI |
| 3.3851 | 7.11E-04 | GTExv8.EUR.Whole_Blood | 5.17E-03 | CTA-384D8.35 | BMI |
| 3.2636 | 1.10E-03 | GTExv8.EUR.Whole_Blood | 7.46E-03 | KLHDC7B | BMI |
| 3.0929 | 1.98E-03 | GTExv8.EUR.Whole_Blood | 1.19E-02 | SYCE3 | BMI |
| 4.2301 | 2.34E-05 | GTExv8.EUR.Whole_Blood | 6.22E-03 | ZMYM1 | Cigarettes smoked per day |
| -3.9584 | 7.55E-05 | GTExv8.EUR.Whole_Blood | 1.18E-02 | INPP5B | Cigarettes smoked per day |
| -3.4729 | 5.15E-04 | GTExv8.EUR.Whole_Blood | 3.91E-02 | SF3A3 | Cigarettes smoked per day |
| -3.6972 | 2.18E-04 | GTExv8.EUR.Whole_Blood | 2.15E-02 | LYSMD1 | Cigarettes smoked per day |
| -4.1144 | 3.88E-05 | GTExv8.EUR.Whole_Blood | 8.36E-03 | RP11-160H22.5 | Cigarettes smoked per day |
| -3.7992 | 1.45E-04 | GTExv8.EUR.Whole_Blood | 1.81E-02 | RP11-318C24.2 | Cigarettes smoked per day |
| -3.6652 | 2.47E-04 | GTExv8.EUR.Whole_Blood | 2.40E-02 | BATF3 | Cigarettes smoked per day |
| -3.5206 | 4.31E-04 | GTExv8.EUR.Whole_Blood | 3.44E-02 | INPP4A | Cigarettes smoked per day |
| 3.7812 | 1.56E-04 | GTExv8.EUR.Whole_Blood | 1.82E-02 | CCNT2-AS1 | Cigarettes smoked per day |
| -4.1538 | 3.27E-05 | GTExv8.EUR.Whole_Blood | 7.63E-03 | CCNT2 | Cigarettes smoked per day |
| 3.7850 | 1.54E-04 | GTExv8.EUR.Whole_Blood | 1.82E-02 | MFSD6 | Cigarettes smoked per day |
| 3.5628 | 3.67E-04 | GTExv8.EUR.Whole_Blood | 3.22E-02 | ARPC2 | Cigarettes smoked per day |
| 3.5122 | 4.44E-04 | GTExv8.EUR.Whole_Blood | 3.50E-02 | RP11-378A13.1 | Cigarettes smoked per day |
| -3.3978 | 6.79E-04 | GTExv8.EUR.Whole_Blood | 4.75E-02 | GPBAR1 | Cigarettes smoked per day |
| 3.6997 | 2.16E-04 | GTExv8.EUR.Whole_Blood | 2.15E-02 | PNKD | Cigarettes smoked per day |
| -3.4166 | 6.34E-04 | GTExv8.EUR.Whole_Blood | 4.55E-02 | HDLBP | Cigarettes smoked per day |
| 3.5825 | 3.40E-04 | GTExv8.EUR.Whole_Blood | 3.01E-02 | PLCL2 | Cigarettes smoked per day |
| 3.7442 | 1.81E-04 | GTExv8.EUR.Whole_Blood | 1.92E-02 | GPD1L | Cigarettes smoked per day |
| -3.4542 | 5.52E-04 | GTExv8.EUR.Whole_Blood | 4.11E-02 | ELP6 | Cigarettes smoked per day |
| -5.8363 | 5.34E-09 | GTExv8.EUR.Whole_Blood | 4.73E-06 | NCKIPSD | Cigarettes smoked per day |
| -3.9616 | 7.44E-05 | GTExv8.EUR.Whole_Blood | 1.18E-02 | P4HTM | Cigarettes smoked per day |
| 5.9258 | 3.11E-09 | GTExv8.EUR.Whole_Blood | 3.10E-06 | WDR6 | Cigarettes smoked per day |
| 3.9832 | 6.80E-05 | GTExv8.EUR.Whole_Blood | 1.15E-02 | QRICH1 | Cigarettes smoked per day |
| 5.3179 | 1.05E-07 | GTExv8.EUR.Whole_Blood | 6.44E-05 | LAMB2 | Cigarettes smoked per day |
| 5.4793 | 4.27E-08 | GTExv8.EUR.Whole_Blood | 2.84E-05 | CCDC71 | Cigarettes smoked per day |
| 3.7758 | 1.60E-04 | GTExv8.EUR.Whole_Blood | 1.82E-02 | RHOA-IT1 | Cigarettes smoked per day |
| -3.3943 | 6.88E-04 | GTExv8.EUR.Whole_Blood | 4.77E-02 | AMT | Cigarettes smoked per day |
| 3.5253 | 4.23E-04 | GTExv8.EUR.Whole_Blood | 3.42E-02 | UBXN7 | Cigarettes smoked per day |
| -4.9628 | 6.95E-07 | GTExv8.EUR.Whole_Blood | 3.46E-04 | ADD1 | Cigarettes smoked per day |
| 4.4974 | 6.88E-06 | GTExv8.EUR.Whole_Blood | 2.89E-03 | GRK4 | Cigarettes smoked per day |
| -4.0555 | 5.00E-05 | GTExv8.EUR.Whole_Blood | 9.72E-03 | RP11-665G4.1 | Cigarettes smoked per day |
| -3.7937 | 1.48E-04 | GTExv8.EUR.Whole_Blood | 1.81E-02 | CC2D2A | Cigarettes smoked per day |
| 4.0688 | 4.73E-05 | GTExv8.EUR.Whole_Blood | 9.51E-03 | RP11-10L12.1 | Cigarettes smoked per day |
| 3.9908 | 6.59E-05 | GTExv8.EUR.Whole_Blood | 1.14E-02 | GATB | Cigarettes smoked per day |
| -3.9191 | 8.89E-05 | GTExv8.EUR.Whole_Blood | 1.29E-02 | LARS | Cigarettes smoked per day |
| -3.4361 | 5.90E-04 | GTExv8.EUR.Whole_Blood | 4.28E-02 | RP11-560J1.2 | Cigarettes smoked per day |
| 3.8677 | 1.10E-04 | GTExv8.EUR.Whole_Blood | 1.44E-02 | PHIP | Cigarettes smoked per day |
| 3.4075 | 6.56E-04 | GTExv8.EUR.Whole_Blood | 4.66E-02 | L3MBTL3 | Cigarettes smoked per day |
| -4.0741 | 4.62E-05 | GTExv8.EUR.Whole_Blood | 9.51E-03 | ARG1 | Cigarettes smoked per day |
| -3.9079 | 9.31E-05 | GTExv8.EUR.Whole_Blood | 1.33E-02 | MED23 | Cigarettes smoked per day |
| 3.7098 | 2.07E-04 | GTExv8.EUR.Whole_Blood | 2.12E-02 | GINM1 | Cigarettes smoked per day |
| 3.7255 | 1.95E-04 | GTExv8.EUR.Whole_Blood | 2.05E-02 | NUP43 | Cigarettes smoked per day |
| 3.6972 | 2.18E-04 | GTExv8.EUR.Whole_Blood | 2.15E-02 | MRM2 | Cigarettes smoked per day |
| -3.5238 | 4.25E-04 | GTExv8.EUR.Whole_Blood | 3.42E-02 | H2AFV | Cigarettes smoked per day |
| 3.9683 | 7.24E-05 | GTExv8.EUR.Whole_Blood | 1.18E-02 | IKZF1 | Cigarettes smoked per day |
| 4.6014 | 4.20E-06 | GTExv8.EUR.Whole_Blood | 1.86E-03 | GTF2I | Cigarettes smoked per day |
| 4.2870 | 1.81E-05 | GTExv8.EUR.Whole_Blood | 4.98E-03 | NCF1 | Cigarettes smoked per day |
| 3.8742 | 1.07E-04 | GTExv8.EUR.Whole_Blood | 1.42E-02 | GTF2IRD2 | Cigarettes smoked per day |
| 3.8389 | 1.24E-04 | GTExv8.EUR.Whole_Blood | 1.59E-02 | STAG3L2 | Cigarettes smoked per day |
| 4.8086 | 1.52E-06 | GTExv8.EUR.Whole_Blood | 7.13E-04 | RCC1L | Cigarettes smoked per day |
| 3.5839 | 3.38E-04 | GTExv8.EUR.Whole_Blood | 3.01E-02 | SPDYE5 | Cigarettes smoked per day |
| -3.7457 | 1.80E-04 | GTExv8.EUR.Whole_Blood | 1.92E-02 | PMS2P3 | Cigarettes smoked per day |
| 4.0477 | 5.17E-05 | GTExv8.EUR.Whole_Blood | 9.81E-03 | ZKSCAN5 | Cigarettes smoked per day |
| 3.5297 | 4.16E-04 | GTExv8.EUR.Whole_Blood | 3.42E-02 | ZNF655 | Cigarettes smoked per day |
| -3.4979 | 4.69E-04 | GTExv8.EUR.Whole_Blood | 3.65E-02 | ASAH1 | Cigarettes smoked per day |
| -3.9676 | 7.26E-05 | GTExv8.EUR.Whole_Blood | 1.18E-02 | ENTPD4 | Cigarettes smoked per day |
| 4.4037 | 1.06E-05 | GTExv8.EUR.Whole_Blood | 3.92E-03 | UTP23 | Cigarettes smoked per day |
| -3.6296 | 2.84E-04 | GTExv8.EUR.Whole_Blood | 2.70E-02 | DENND3 | Cigarettes smoked per day |
| 3.7649 | 1.67E-04 | GTExv8.EUR.Whole_Blood | 1.87E-02 | WHRN | Cigarettes smoked per day |
| -4.1574 | 3.22E-05 | GTExv8.EUR.Whole_Blood | 7.63E-03 | TLR4 | Cigarettes smoked per day |
| -4.1483 | 3.35E-05 | GTExv8.EUR.Whole_Blood | 7.63E-03 | RP11-281A20.2 | Cigarettes smoked per day |
| 3.8860 | 1.02E-04 | GTExv8.EUR.Whole_Blood | 1.40E-02 | PPP6C | Cigarettes smoked per day |
| 3.4871 | 4.88E-04 | GTExv8.EUR.Whole_Blood | 3.74E-02 | RABEPK | Cigarettes smoked per day |
| -3.5350 | 4.08E-04 | GTExv8.EUR.Whole_Blood | 3.39E-02 | CACFD1 | Cigarettes smoked per day |
| -3.7500 | 1.74E-04 | GTExv8.EUR.Whole_Blood | 1.90E-02 | BORCS7 | Cigarettes smoked per day |
| 3.5505 | 3.85E-04 | GTExv8.EUR.Whole_Blood | 3.26E-02 | LDHA | Cigarettes smoked per day |
| 4.4259 | 9.61E-06 | GTExv8.EUR.Whole_Blood | 3.83E-03 | DGKZ | Cigarettes smoked per day |
| -4.3346 | 1.46E-05 | GTExv8.EUR.Whole_Blood | 4.64E-03 | ATG13 | Cigarettes smoked per day |
| -3.4053 | 6.61E-04 | GTExv8.EUR.Whole_Blood | 4.66E-02 | NR1H3 | Cigarettes smoked per day |
| 4.3899 | 1.13E-05 | GTExv8.EUR.Whole_Blood | 3.92E-03 | MADD | Cigarettes smoked per day |
| 3.5483 | 3.88E-04 | GTExv8.EUR.Whole_Blood | 3.26E-02 | KBTBD3 | Cigarettes smoked per day |
| -3.9535 | 7.70E-05 | GTExv8.EUR.Whole_Blood | 1.18E-02 | ANKK1 | Cigarettes smoked per day |
| -4.2921 | 1.77E-05 | GTExv8.EUR.Whole_Blood | 4.98E-03 | MVK | Cigarettes smoked per day |
| -3.4370 | 5.88E-04 | GTExv8.EUR.Whole_Blood | 4.28E-02 | MMAB | Cigarettes smoked per day |
| -3.4446 | 5.72E-04 | GTExv8.EUR.Whole_Blood | 4.22E-02 | P2RX7 | Cigarettes smoked per day |
| 4.3913 | 1.13E-05 | GTExv8.EUR.Whole_Blood | 3.92E-03 | KLC1 | Cigarettes smoked per day |
| 3.8921 | 9.94E-05 | GTExv8.EUR.Whole_Blood | 1.39E-02 | BAG5 | Cigarettes smoked per day |
| 4.3251 | 1.52E-05 | GTExv8.EUR.Whole_Blood | 4.64E-03 | RP11-73M18.7 | Cigarettes smoked per day |
| 4.1739 | 2.99E-05 | GTExv8.EUR.Whole_Blood | 7.52E-03 | RP11-73M18.8 | Cigarettes smoked per day |
| 5.1719 | 2.32E-07 | GTExv8.EUR.Whole_Blood | 1.23E-04 | XRCC3 | Cigarettes smoked per day |
| 3.7911 | 1.50E-04 | GTExv8.EUR.Whole_Blood | 1.81E-02 | RP11-133K1.12 | Cigarettes smoked per day |
| -3.9907 | 6.59E-05 | GTExv8.EUR.Whole_Blood | 1.14E-02 | TCF12 | Cigarettes smoked per day |
| -8.0691 | 7.08E-16 | GTExv8.EUR.Whole_Blood | 1.41E-12 | IREB2 | Cigarettes smoked per day |
| 31.5370 | 2.71E-218 | GTExv8.EUR.Whole_Blood | 2.16E-214 | PSMA4 | Cigarettes smoked per day |
| -10.7322 | 7.19E-27 | GTExv8.EUR.Whole_Blood | 2.87E-23 | CHRNA5 | Cigarettes smoked per day |
| 5.2762 | 1.32E-07 | GTExv8.EUR.Whole_Blood | 7.52E-05 | MORF4L1 | Cigarettes smoked per day |
| 3.6050 | 3.12E-04 | GTExv8.EUR.Whole_Blood | 2.87E-02 | CTSH | Cigarettes smoked per day |
| 3.8742 | 1.07E-04 | GTExv8.EUR.Whole_Blood | 1.42E-02 | NPIPB2 | Cigarettes smoked per day |
| -3.6194 | 2.95E-04 | GTExv8.EUR.Whole_Blood | 2.77E-02 | RP11-1072A3.3 | Cigarettes smoked per day |
| -3.5481 | 3.88E-04 | GTExv8.EUR.Whole_Blood | 3.26E-02 | HSD3B7 | Cigarettes smoked per day |
| 4.1718 | 3.02E-05 | GTExv8.EUR.Whole_Blood | 7.52E-03 | RP11-196G11.2 | Cigarettes smoked per day |
| -4.0345 | 5.47E-05 | GTExv8.EUR.Whole_Blood | 1.01E-02 | RP11-196G11.6 | Cigarettes smoked per day |
| -3.6607 | 2.51E-04 | GTExv8.EUR.Whole_Blood | 2.41E-02 | ZNF668 | Cigarettes smoked per day |
| -4.3435 | 1.40E-05 | GTExv8.EUR.Whole_Blood | 4.64E-03 | PRSS53 | Cigarettes smoked per day |
| -4.0237 | 5.73E-05 | GTExv8.EUR.Whole_Blood | 1.04E-02 | VKORC1 | Cigarettes smoked per day |
| -4.0667 | 4.77E-05 | GTExv8.EUR.Whole_Blood | 9.51E-03 | BCKDK | Cigarettes smoked per day |
| 3.6043 | 3.13E-04 | GTExv8.EUR.Whole_Blood | 2.87E-02 | RP11-196G11.4 | Cigarettes smoked per day |
| -3.9433 | 8.04E-05 | GTExv8.EUR.Whole_Blood | 1.19E-02 | RNF166 | Cigarettes smoked per day |
| -3.4673 | 5.26E-04 | GTExv8.EUR.Whole_Blood | 3.96E-02 | AC137932.5 | Cigarettes smoked per day |
| 6.0165 | 1.78E-09 | GTExv8.EUR.Whole_Blood | 2.03E-06 | SPATA33 | Cigarettes smoked per day |
| -5.6856 | 1.30E-08 | GTExv8.EUR.Whole_Blood | 1.04E-05 | SPATA2L | Cigarettes smoked per day |
| 3.8156 | 1.36E-04 | GTExv8.EUR.Whole_Blood | 1.72E-02 | ERAL1 | Cigarettes smoked per day |
| -3.7612 | 1.69E-04 | GTExv8.EUR.Whole_Blood | 1.87E-02 | GGNBP2 | Cigarettes smoked per day |
| -5.5065 | 3.66E-08 | GTExv8.EUR.Whole_Blood | 2.65E-05 | ZBTB7A | Cigarettes smoked per day |
| 3.9472 | 7.91E-05 | GTExv8.EUR.Whole_Blood | 1.19E-02 | ANKRD24 | Cigarettes smoked per day |
| 3.7171 | 2.02E-04 | GTExv8.EUR.Whole_Blood | 2.09E-02 | ZNF333 | Cigarettes smoked per day |
| -3.4968 | 4.71E-04 | GTExv8.EUR.Whole_Blood | 3.65E-02 | ZNF430 | Cigarettes smoked per day |
| -7.6908 | 1.46E-14 | GTExv8.EUR.Whole_Blood | 1.94E-11 | C19orf54 | Cigarettes smoked per day |
| 7.8482 | 4.22E-15 | GTExv8.EUR.Whole_Blood | 6.73E-12 | EGLN2 | Cigarettes smoked per day |
| -8.5749 | 9.92E-18 | GTExv8.EUR.Whole_Blood | 2.64E-14 | CYP2T1P | Cigarettes smoked per day |
| 4.1350 | 3.55E-05 | GTExv8.EUR.Whole_Blood | 7.86E-03 | CCDC9 | Cigarettes smoked per day |
| 3.5969 | 3.22E-04 | GTExv8.EUR.Whole_Blood | 2.92E-02 | BTBD3 | Cigarettes smoked per day |
| -3.7746 | 1.60E-04 | GTExv8.EUR.Whole_Blood | 1.82E-02 | ARFRP1 | Cigarettes smoked per day |
| -3.5591 | 3.72E-04 | GTExv8.EUR.Whole_Blood | 3.22E-02 | DGCR9 | Cigarettes smoked per day |
| -4.3191 | 1.57E-05 | GTExv8.EUR.Whole_Blood | 4.64E-03 | CA15P1 | Cigarettes smoked per day |
| -4.3414 | 1.42E-05 | GTExv8.EUR.Whole_Blood | 8.07E-03 | RP11-261C10.7 | CKD |
| -7.5438 | 4.56E-14 | GTExv8.EUR.Whole_Blood | 3.63E-10 | LINC01347 | CKD |
| 6.9329 | 4.12E-12 | GTExv8.EUR.Whole_Blood | 6.91E-09 | CEP170 | CKD |
| 7.1949 | 6.25E-13 | GTExv8.EUR.Whole_Blood | 2.49E-09 | SDCCAG8 | CKD |
| -3.7200 | 1.98E-04 | GTExv8.EUR.Whole_Blood | 4.63E-02 | ALMS1 | CKD |
| -3.9400 | 7.98E-05 | GTExv8.EUR.Whole_Blood | 3.16E-02 | FAHD2CP | CKD |
| 3.7454 | 1.80E-04 | GTExv8.EUR.Whole_Blood | 4.48E-02 | ATG7 | CKD |
| 3.7842 | 1.54E-04 | GTExv8.EUR.Whole_Blood | 4.08E-02 | PSMD6-AS1 | CKD |
| 3.7869 | 1.53E-04 | GTExv8.EUR.Whole_Blood | 4.08E-02 | ZBTB38 | CKD |
| -3.9343 | 8.34E-05 | GTExv8.EUR.Whole_Blood | 3.16E-02 | DHX36 | CKD |
| -3.9831 | 6.80E-05 | GTExv8.EUR.Whole_Blood | 3.01E-02 | FAM13A-AS1 | CKD |
| -3.8981 | 9.69E-05 | GTExv8.EUR.Whole_Blood | 3.34E-02 | FAM13A | CKD |
| -3.9429 | 8.05E-05 | GTExv8.EUR.Whole_Blood | 3.16E-02 | HSPA4 | CKD |
| 5.4751 | 4.37E-08 | GTExv8.EUR.Whole_Blood | 4.97E-05 | LMAN2 | CKD |
| 4.0652 | 4.80E-05 | GTExv8.EUR.Whole_Blood | 2.25E-02 | L3MBTL3 | CKD |
| 3.8667 | 1.10E-04 | GTExv8.EUR.Whole_Blood | 3.37E-02 | COL17A1 | CKD |
| -4.6100 | 4.04E-06 | GTExv8.EUR.Whole_Blood | 2.92E-03 | SBF2 | CKD |
| 5.7600 | 8.50E-09 | GTExv8.EUR.Whole_Blood | 1.13E-05 | MAP3K11 | CKD |
| -5.1400 | 2.81E-07 | GTExv8.EUR.Whole_Blood | 2.48E-04 | KAT5 | CKD |
| -3.9000 | 9.70E-05 | GTExv8.EUR.Whole_Blood | 3.34E-02 | HINFP | CKD |
| 4.1039 | 4.06E-05 | GTExv8.EUR.Whole_Blood | 2.02E-02 | RP11-620J15.3 | CKD |
| 3.7532 | 1.75E-04 | GTExv8.EUR.Whole_Blood | 4.48E-02 | ATP23 | CKD |
| -3.8246 | 1.31E-04 | GTExv8.EUR.Whole_Blood | 3.86E-02 | CHP1 | CKD |
| -6.9256 | 4.34E-12 | GTExv8.EUR.Whole_Blood | 6.91E-09 | SHF | CKD |
| -5.1541 | 2.55E-07 | GTExv8.EUR.Whole_Blood | 2.48E-04 | GATM | CKD |
| 7.0680 | 1.57E-12 | GTExv8.EUR.Whole_Blood | 4.16E-09 | SPATA5L1 | CKD |
| -4.2400 | 2.26E-05 | GTExv8.EUR.Whole_Blood | 1.20E-02 | ZNHIT3 | CKD |
| 4.3500 | 1.37E-05 | GTExv8.EUR.Whole_Blood | 8.07E-03 | MYO19 | CKD |
| -4.7200 | 2.31E-06 | GTExv8.EUR.Whole_Blood | 1.84E-03 | GGNBP2 | CKD |
| 4.3900 | 1.13E-05 | GTExv8.EUR.Whole_Blood | 7.49E-03 | DHRS11 | CKD |
| -3.8100 | 1.42E-04 | GTExv8.EUR.Whole_Blood | 4.03E-02 | PNMT | CKD |
| -3.7300 | 1.89E-04 | GTExv8.EUR.Whole_Blood | 4.56E-02 | PGAP3 | CKD |
| 3.8853 | 1.02E-04 | GTExv8.EUR.Whole_Blood | 3.34E-02 | MAN2B1 | CKD |
| -3.8795 | 1.05E-04 | GTExv8.EUR.Whole_Blood | 3.34E-02 | SLC7A9 | CKD |
| 3.4546 | 5.51E-04 | GTExv8.EUR.Whole_Blood | 4.62E-03 | PLEKHN1 | DBP |
| 2.5626 | 1.04E-02 | GTExv8.EUR.Whole_Blood | 4.87E-02 | ISG15 | DBP |
| -6.5543 | 5.59E-11 | GTExv8.EUR.Whole_Blood | 2.97E-09 | RP1-140A9.1 | DBP |
| 4.8166 | 1.46E-06 | GTExv8.EUR.Whole_Blood | 2.67E-05 | PRKCZ | DBP |
| 3.6080 | 3.09E-04 | GTExv8.EUR.Whole_Blood | 2.84E-03 | RP5-892K4.1 | DBP |
| 4.8037 | 1.56E-06 | GTExv8.EUR.Whole_Blood | 2.83E-05 | PRKCZ-AS1 | DBP |
| -3.1814 | 1.47E-03 | GTExv8.EUR.Whole_Blood | 1.03E-02 | PHF13 | DBP |
| 5.2174 | 1.82E-07 | GTExv8.EUR.Whole_Blood | 4.18E-06 | RP5-1115A15.1 | DBP |
| 4.8883 | 1.02E-06 | GTExv8.EUR.Whole_Blood | 1.96E-05 | RERE | DBP |
| 3.2501 | 1.15E-03 | GTExv8.EUR.Whole_Blood | 8.33E-03 | RP3-477M7.5 | DBP |
| 3.0264 | 2.47E-03 | GTExv8.EUR.Whole_Blood | 1.55E-02 | RP4-635E18.7 | DBP |
| -3.1248 | 1.78E-03 | GTExv8.EUR.Whole_Blood | 1.20E-02 | MTOR | DBP |
| 2.7199 | 6.53E-03 | GTExv8.EUR.Whole_Blood | 3.36E-02 | FBXO6 | DBP |
| -12.8103 | 1.44E-37 | GTExv8.EUR.Whole_Blood | 7.61E-35 | MTHFR | DBP |
| -11.8998 | 1.19E-32 | GTExv8.EUR.Whole_Blood | 5.55E-30 | CLCN6 | DBP |
| -5.0318 | 4.86E-07 | GTExv8.EUR.Whole_Blood | 1.01E-05 | NPPA-AS1 | DBP |
| -13.7477 | 5.26E-43 | GTExv8.EUR.Whole_Blood | 5.21E-40 | NPPA | DBP |
| 2.9835 | 2.85E-03 | GTExv8.EUR.Whole_Blood | 1.74E-02 | TMEM51 | DBP |
| 6.3380 | 2.33E-10 | GTExv8.EUR.Whole_Blood | 1.04E-08 | DDI2 | DBP |
| -4.6710 | 3.00E-06 | GTExv8.EUR.Whole_Blood | 5.07E-05 | PLEKHM2 | DBP |
| 3.3854 | 7.11E-04 | GTExv8.EUR.Whole_Blood | 5.66E-03 | SLC25A34 | DBP |
| 3.3818 | 7.20E-04 | GTExv8.EUR.Whole_Blood | 5.71E-03 | RP11-169K16.8 | DBP |
| 2.7013 | 6.91E-03 | GTExv8.EUR.Whole_Blood | 3.53E-02 | FLJ37453 | DBP |
| -6.9551 | 3.52E-12 | GTExv8.EUR.Whole_Blood | 2.49E-10 | SPEN | DBP |
| -4.7012 | 2.59E-06 | GTExv8.EUR.Whole_Blood | 4.45E-05 | RSG1 | DBP |
| -3.3107 | 9.31E-04 | GTExv8.EUR.Whole_Blood | 7.03E-03 | NECAP2 | DBP |
| 5.3130 | 1.08E-07 | GTExv8.EUR.Whole_Blood | 2.61E-06 | MST1P2 | DBP |
| -4.0661 | 4.78E-05 | GTExv8.EUR.Whole_Blood | 5.81E-04 | MST1L | DBP |
| -3.9472 | 7.91E-05 | GTExv8.EUR.Whole_Blood | 9.01E-04 | RP11-108M9.6 | DBP |
| -3.0136 | 2.58E-03 | GTExv8.EUR.Whole_Blood | 1.61E-02 | CROCC | DBP |
| 3.3743 | 7.40E-04 | GTExv8.EUR.Whole_Blood | 5.85E-03 | PADI2 | DBP |
| -2.7728 | 5.56E-03 | GTExv8.EUR.Whole_Blood | 2.97E-02 | MINOS1 | DBP |
| -5.1491 | 2.62E-07 | GTExv8.EUR.Whole_Blood | 5.86E-06 | MUL1 | DBP |
| -3.7574 | 1.72E-04 | GTExv8.EUR.Whole_Blood | 1.74E-03 | CDC42 | DBP |
| 2.9721 | 2.96E-03 | GTExv8.EUR.Whole_Blood | 1.79E-02 | ELOA | DBP |
| 3.2521 | 1.15E-03 | GTExv8.EUR.Whole_Blood | 8.33E-03 | ELOA-AS1 | DBP |
| 3.2757 | 1.05E-03 | GTExv8.EUR.Whole_Blood | 7.78E-03 | HMGCL | DBP |
| 7.8638 | 3.73E-15 | GTExv8.EUR.Whole_Blood | 4.28E-13 | CLIC4 | DBP |
| 3.5907 | 3.30E-04 | GTExv8.EUR.Whole_Blood | 3.01E-03 | RP3-465N24.5 | DBP |
| 3.5930 | 3.27E-04 | GTExv8.EUR.Whole_Blood | 2.99E-03 | RHD | DBP |
| 3.4838 | 4.94E-04 | GTExv8.EUR.Whole_Blood | 4.20E-03 | SDHDP6 | DBP |
| 3.7489 | 1.78E-04 | GTExv8.EUR.Whole_Blood | 1.79E-03 | TMEM50A | DBP |
| -2.6969 | 7.00E-03 | GTExv8.EUR.Whole_Blood | 3.56E-02 | RHCE | DBP |
| -3.2382 | 1.20E-03 | GTExv8.EUR.Whole_Blood | 8.62E-03 | LDLRAP1 | DBP |
| 3.5647 | 3.64E-04 | GTExv8.EUR.Whole_Blood | 3.27E-03 | DHDDS | DBP |
| -6.4196 | 1.37E-10 | GTExv8.EUR.Whole_Blood | 6.58E-09 | RP5-968P14.2 | DBP |
| -6.3225 | 2.57E-10 | GTExv8.EUR.Whole_Blood | 1.14E-08 | PIGV | DBP |
| 5.4298 | 5.64E-08 | GTExv8.EUR.Whole_Blood | 1.43E-06 | ZDHHC18 | DBP |
| -4.2473 | 2.16E-05 | GTExv8.EUR.Whole_Blood | 2.97E-04 | SLC9A1 | DBP |
| -5.4630 | 4.68E-08 | GTExv8.EUR.Whole_Blood | 1.21E-06 | SYTL1 | DBP |
| -4.0704 | 4.69E-05 | GTExv8.EUR.Whole_Blood | 5.73E-04 | MAP3K6 | DBP |
| -4.7285 | 2.26E-06 | GTExv8.EUR.Whole_Blood | 3.92E-05 | WASF2 | DBP |
| -2.5532 | 1.07E-02 | GTExv8.EUR.Whole_Blood | 4.98E-02 | RPA2 | DBP |
| 3.3111 | 9.29E-04 | GTExv8.EUR.Whole_Blood | 7.02E-03 | SESN2 | DBP |
| 4.2081 | 2.58E-05 | GTExv8.EUR.Whole_Blood | 3.45E-04 | PHACTR4 | DBP |
| 4.1803 | 2.91E-05 | GTExv8.EUR.Whole_Blood | 3.81E-04 | SNHG12 | DBP |
| 3.7434 | 1.82E-04 | GTExv8.EUR.Whole_Blood | 1.82E-03 | YTHDF2 | DBP |
| 4.6251 | 3.74E-06 | GTExv8.EUR.Whole_Blood | 6.11E-05 | EPB41 | DBP |
| -4.5243 | 6.06E-06 | GTExv8.EUR.Whole_Blood | 9.49E-05 | MECR | DBP |
| -4.3280 | 1.51E-05 | GTExv8.EUR.Whole_Blood | 2.14E-04 | PEF1 | DBP |
| -2.7542 | 5.88E-03 | GTExv8.EUR.Whole_Blood | 3.09E-02 | ZBTB8OS | DBP |
| -2.8268 | 4.70E-03 | GTExv8.EUR.Whole_Blood | 2.58E-02 | RBBP4 | DBP |
| -3.1905 | 1.42E-03 | GTExv8.EUR.Whole_Blood | 9.94E-03 | KIAA1522 | DBP |
| -2.8747 | 4.04E-03 | GTExv8.EUR.Whole_Blood | 2.30E-02 | GJB5 | DBP |
| 3.4557 | 5.49E-04 | GTExv8.EUR.Whole_Blood | 4.61E-03 | SMIM12 | DBP |
| -3.8175 | 1.35E-04 | GTExv8.EUR.Whole_Blood | 1.43E-03 | YRDC | DBP |
| 4.6670 | 3.06E-06 | GTExv8.EUR.Whole_Blood | 5.15E-05 | INPP5B | DBP |
| 5.2736 | 1.34E-07 | GTExv8.EUR.Whole_Blood | 3.16E-06 | UTP11 | DBP |
| -4.4467 | 8.72E-06 | GTExv8.EUR.Whole_Blood | 1.30E-04 | NDUFS5 | DBP |
| 4.7776 | 1.77E-06 | GTExv8.EUR.Whole_Blood | 3.17E-05 | BMP8A | DBP |
| 5.9131 | 3.36E-09 | GTExv8.EUR.Whole_Blood | 1.17E-07 | RP11-69E11.4 | DBP |
| 4.2622 | 2.02E-05 | GTExv8.EUR.Whole_Blood | 2.79E-04 | PPIEL | DBP |
| -5.9859 | 2.15E-09 | GTExv8.EUR.Whole_Blood | 7.78E-08 | PABPC4 | DBP |
| -4.0047 | 6.21E-05 | GTExv8.EUR.Whole_Blood | 7.37E-04 | HPCAL4 | DBP |
| 6.0334 | 1.61E-09 | GTExv8.EUR.Whole_Blood | 5.96E-08 | HIVEP3 | DBP |
| -3.5728 | 3.53E-04 | GTExv8.EUR.Whole_Blood | 3.17E-03 | CCDC30 | DBP |
| -3.9501 | 7.81E-05 | GTExv8.EUR.Whole_Blood | 8.92E-04 | P3H1 | DBP |
| -2.5714 | 1.01E-02 | GTExv8.EUR.Whole_Blood | 4.76E-02 | TMEM269 | DBP |
| 3.3595 | 7.81E-04 | GTExv8.EUR.Whole_Blood | 6.12E-03 | SLC2A1-AS1 | DBP |
| 5.7639 | 8.22E-09 | GTExv8.EUR.Whole_Blood | 2.51E-07 | TIE1 | DBP |
| 8.3957 | 4.63E-17 | GTExv8.EUR.Whole_Blood | 6.67E-15 | SZT2 | DBP |
| -3.5459 | 3.91E-04 | GTExv8.EUR.Whole_Blood | 3.46E-03 | DMAP1 | DBP |
| -4.6389 | 3.50E-06 | GTExv8.EUR.Whole_Blood | 5.79E-05 | C1orf228 | DBP |
| -2.6822 | 7.31E-03 | GTExv8.EUR.Whole_Blood | 3.68E-02 | RPS8 | DBP |
| -3.1980 | 1.38E-03 | GTExv8.EUR.Whole_Blood | 9.71E-03 | BTBD19 | DBP |
| -2.9416 | 3.26E-03 | GTExv8.EUR.Whole_Blood | 1.94E-02 | CCDC17 | DBP |
| 4.2187 | 2.46E-05 | GTExv8.EUR.Whole_Blood | 3.31E-04 | RP11-767N6.2 | DBP |
| -3.0877 | 2.02E-03 | GTExv8.EUR.Whole_Blood | 1.32E-02 | MAST2 | DBP |
| -2.8112 | 4.94E-03 | GTExv8.EUR.Whole_Blood | 2.70E-02 | RP4-657D16.3 | DBP |
| -3.4864 | 4.90E-04 | GTExv8.EUR.Whole_Blood | 4.18E-03 | NRDC | DBP |
| 3.0045 | 2.66E-03 | GTExv8.EUR.Whole_Blood | 1.65E-02 | RP11-155O18.6 | DBP |
| 3.3366 | 8.48E-04 | GTExv8.EUR.Whole_Blood | 6.52E-03 | CC2D1B | DBP |
| 2.7973 | 5.15E-03 | GTExv8.EUR.Whole_Blood | 2.78E-02 | PRPF38A | DBP |
| 2.7973 | 5.15E-03 | GTExv8.EUR.Whole_Blood | 2.78E-02 | ZCCHC11 | DBP |
| 4.1321 | 3.60E-05 | GTExv8.EUR.Whole_Blood | 4.55E-04 | GPX7 | DBP |
| 3.3747 | 7.39E-04 | GTExv8.EUR.Whole_Blood | 5.84E-03 | PLPP3 | DBP |
| -6.7170 | 1.86E-11 | GTExv8.EUR.Whole_Blood | 1.13E-09 | SGIP1 | DBP |
| 2.9651 | 3.03E-03 | GTExv8.EUR.Whole_Blood | 1.83E-02 | TCTEX1D1 | DBP |
| 2.7672 | 5.65E-03 | GTExv8.EUR.Whole_Blood | 3.00E-02 | TNNI3K | DBP |
| 2.6388 | 8.32E-03 | GTExv8.EUR.Whole_Blood | 4.10E-02 | SPATA1 | DBP |
| -2.8972 | 3.76E-03 | GTExv8.EUR.Whole_Blood | 2.17E-02 | KYAT3 | DBP |
| 3.6612 | 2.51E-04 | GTExv8.EUR.Whole_Blood | 2.41E-03 | LRRC8D | DBP |
| 3.7224 | 1.97E-04 | GTExv8.EUR.Whole_Blood | 1.95E-03 | EVI5 | DBP |
| 3.1174 | 1.82E-03 | GTExv8.EUR.Whole_Blood | 1.21E-02 | CCDC18-AS1 | DBP |
| -2.9307 | 3.38E-03 | GTExv8.EUR.Whole_Blood | 2.00E-02 | DR1 | DBP |
| 2.9988 | 2.71E-03 | GTExv8.EUR.Whole_Blood | 1.67E-02 | ABCD3 | DBP |
| -3.4086 | 6.53E-04 | GTExv8.EUR.Whole_Blood | 5.29E-03 | DPYD | DBP |
| -2.7722 | 5.57E-03 | GTExv8.EUR.Whole_Blood | 2.97E-02 | GSTM4 | DBP |
| -5.0889 | 3.60E-07 | GTExv8.EUR.Whole_Blood | 7.71E-06 | GSTM2 | DBP |
| -5.1809 | 2.21E-07 | GTExv8.EUR.Whole_Blood | 4.99E-06 | GSTM1 | DBP |
| -3.9205 | 8.84E-05 | GTExv8.EUR.Whole_Blood | 9.95E-04 | GSTM5 | DBP |
| -3.7912 | 1.50E-04 | GTExv8.EUR.Whole_Blood | 1.57E-03 | FAM212B | DBP |
| -6.1345 | 8.54E-10 | GTExv8.EUR.Whole_Blood | 3.38E-08 | SLC16A1-AS1 | DBP |
| -3.3694 | 7.53E-04 | GTExv8.EUR.Whole_Blood | 5.93E-03 | AP4B1-AS1 | DBP |
| -3.1732 | 1.51E-03 | GTExv8.EUR.Whole_Blood | 1.05E-02 | PTPN22 | DBP |
| -4.1322 | 3.59E-05 | GTExv8.EUR.Whole_Blood | 4.55E-04 | BCL2L15 | DBP |
| -3.2125 | 1.32E-03 | GTExv8.EUR.Whole_Blood | 9.36E-03 | BCAS2 | DBP |
| -3.8704 | 1.09E-04 | GTExv8.EUR.Whole_Blood | 1.19E-03 | WARS2 | DBP |
| 3.1454 | 1.66E-03 | GTExv8.EUR.Whole_Blood | 1.13E-02 | NBPF8 | DBP |
| 3.3599 | 7.80E-04 | GTExv8.EUR.Whole_Blood | 6.12E-03 | RNF115 | DBP |
| 3.0112 | 2.60E-03 | GTExv8.EUR.Whole_Blood | 1.62E-02 | LIX1L-AS1 | DBP |
| -2.8992 | 3.74E-03 | GTExv8.EUR.Whole_Blood | 2.17E-02 | MRPS21 | DBP |
| -3.2906 | 1.00E-03 | GTExv8.EUR.Whole_Blood | 7.47E-03 | ECM1 | DBP |
| -3.4040 | 6.64E-04 | GTExv8.EUR.Whole_Blood | 5.35E-03 | MCL1 | DBP |
| 2.7601 | 5.78E-03 | GTExv8.EUR.Whole_Blood | 3.06E-02 | ENSA | DBP |
| -2.9800 | 2.88E-03 | GTExv8.EUR.Whole_Blood | 1.75E-02 | POGZ | DBP |
| -4.8952 | 9.82E-07 | GTExv8.EUR.Whole_Blood | 1.90E-05 | DENND4B | DBP |
| -6.4107 | 1.45E-10 | GTExv8.EUR.Whole_Blood | 6.92E-09 | SLC39A1 | DBP |
| -4.4763 | 7.60E-06 | GTExv8.EUR.Whole_Blood | 1.16E-04 | CREB3L4 | DBP |
| -4.9105 | 9.08E-07 | GTExv8.EUR.Whole_Blood | 1.79E-05 | TPM3 | DBP |
| 4.1166 | 3.84E-05 | GTExv8.EUR.Whole_Blood | 4.84E-04 | IL6R | DBP |
| 4.4337 | 9.26E-06 | GTExv8.EUR.Whole_Blood | 1.37E-04 | ADAM15 | DBP |
| -3.2342 | 1.22E-03 | GTExv8.EUR.Whole_Blood | 8.76E-03 | SEMA4A | DBP |
| 4.1164 | 3.85E-05 | GTExv8.EUR.Whole_Blood | 4.84E-04 | PMF1 | DBP |
| 3.8035 | 1.43E-04 | GTExv8.EUR.Whole_Blood | 1.49E-03 | BGLAP | DBP |
| 2.7763 | 5.50E-03 | GTExv8.EUR.Whole_Blood | 2.94E-02 | COPA | DBP |
| 3.3160 | 9.13E-04 | GTExv8.EUR.Whole_Blood | 6.93E-03 | RGS5 | DBP |
| -4.2358 | 2.28E-05 | GTExv8.EUR.Whole_Blood | 3.09E-04 | MGST3 | DBP |
| 3.0650 | 2.18E-03 | GTExv8.EUR.Whole_Blood | 1.40E-02 | DUTP6 | DBP |
| -3.7519 | 1.76E-04 | GTExv8.EUR.Whole_Blood | 1.77E-03 | CREG1 | DBP |
| -3.6305 | 2.83E-04 | GTExv8.EUR.Whole_Blood | 2.65E-03 | METTL13 | DBP |
| 3.6413 | 2.71E-04 | GTExv8.EUR.Whole_Blood | 2.57E-03 | KIAA0040 | DBP |
| -4.9026 | 9.46E-07 | GTExv8.EUR.Whole_Blood | 1.85E-05 | LAMC1 | DBP |
| -2.7954 | 5.18E-03 | GTExv8.EUR.Whole_Blood | 2.80E-02 | EDEM3 | DBP |
| 3.3369 | 8.47E-04 | GTExv8.EUR.Whole_Blood | 6.52E-03 | PLEKHA6 | DBP |
| -7.7202 | 1.16E-14 | GTExv8.EUR.Whole_Blood | 1.21E-12 | MDM4 | DBP |
| -4.9392 | 7.84E-07 | GTExv8.EUR.Whole_Blood | 1.56E-05 | LRRN2 | DBP |
| 3.1626 | 1.56E-03 | GTExv8.EUR.Whole_Blood | 1.08E-02 | TRAF3IP3 | DBP |
| -2.6246 | 8.67E-03 | GTExv8.EUR.Whole_Blood | 4.22E-02 | IRF6 | DBP |
| -3.5462 | 3.91E-04 | GTExv8.EUR.Whole_Blood | 3.46E-03 | TRAF5 | DBP |
| 3.2663 | 1.09E-03 | GTExv8.EUR.Whole_Blood | 7.99E-03 | INTS7 | DBP |
| -2.8256 | 4.72E-03 | GTExv8.EUR.Whole_Blood | 2.59E-02 | PPP2R5A | DBP |
| -3.3846 | 7.13E-04 | GTExv8.EUR.Whole_Blood | 5.67E-03 | RP11-384C4.2 | DBP |
| 2.6533 | 7.97E-03 | GTExv8.EUR.Whole_Blood | 3.95E-02 | FLVCR1 | DBP |
| 2.5963 | 9.42E-03 | GTExv8.EUR.Whole_Blood | 4.51E-02 | RAB3GAP2 | DBP |
| 2.8633 | 4.19E-03 | GTExv8.EUR.Whole_Blood | 2.36E-02 | MARC2 | DBP |
| 2.6775 | 7.42E-03 | GTExv8.EUR.Whole_Blood | 3.71E-02 | PARP1 | DBP |
| -3.9503 | 7.80E-05 | GTExv8.EUR.Whole_Blood | 8.92E-04 | CDC42BPA | DBP |
| -4.1856 | 2.84E-05 | GTExv8.EUR.Whole_Blood | 3.74E-04 | RP5-881P19.7 | DBP |
| -5.5731 | 2.50E-08 | GTExv8.EUR.Whole_Blood | 6.95E-07 | IBA57 | DBP |
| 7.9189 | 2.40E-15 | GTExv8.EUR.Whole_Blood | 2.93E-13 | C1orf145 | DBP |
| -4.5363 | 5.72E-06 | GTExv8.EUR.Whole_Blood | 8.99E-05 | RP11-261C10.7 | DBP |
| -13.2677 | 3.56E-40 | GTExv8.EUR.Whole_Blood | 2.56E-37 | LINC01347 | DBP |
| 13.2020 | 8.55E-40 | GTExv8.EUR.Whole_Blood | 5.65E-37 | CEP170 | DBP |
| 13.4242 | 4.36E-41 | GTExv8.EUR.Whole_Blood | 3.84E-38 | SDCCAG8 | DBP |
| -2.8642 | 4.18E-03 | GTExv8.EUR.Whole_Blood | 2.36E-02 | RP11-488L18.4 | DBP |
| -3.7795 | 1.57E-04 | GTExv8.EUR.Whole_Blood | 1.62E-03 | RNF144A | DBP |
| 3.6083 | 3.08E-04 | GTExv8.EUR.Whole_Blood | 2.84E-03 | CPSF3 | DBP |
| -2.7350 | 6.24E-03 | GTExv8.EUR.Whole_Blood | 3.25E-02 | FKBP1B | DBP |
| 2.7335 | 6.27E-03 | GTExv8.EUR.Whole_Blood | 3.26E-02 | FAM228A | DBP |
| -8.8805 | 6.66E-19 | GTExv8.EUR.Whole_Blood | 1.12E-16 | PTRHD1 | DBP |
| -8.8561 | 8.29E-19 | GTExv8.EUR.Whole_Blood | 1.36E-16 | CENPO | DBP |
| -8.2368 | 1.77E-16 | GTExv8.EUR.Whole_Blood | 2.34E-14 | RP11-443B20.1 | DBP |
| -8.9479 | 3.62E-19 | GTExv8.EUR.Whole_Blood | 6.37E-17 | ADCY3 | DBP |
| -9.2799 | 1.70E-20 | GTExv8.EUR.Whole_Blood | 3.29E-18 | DNAJC27 | DBP |
| 7.4484 | 9.45E-14 | GTExv8.EUR.Whole_Blood | 8.91E-12 | SELENOI | DBP |
| -2.8167 | 4.85E-03 | GTExv8.EUR.Whole_Blood | 2.66E-02 | DRC1 | DBP |
| 3.1761 | 1.49E-03 | GTExv8.EUR.Whole_Blood | 1.04E-02 | EMILIN1 | DBP |
| 2.5855 | 9.72E-03 | GTExv8.EUR.Whole_Blood | 4.61E-02 | PREB | DBP |
| -3.9348 | 8.33E-05 | GTExv8.EUR.Whole_Blood | 9.43E-04 | ATRAID | DBP |
| 3.7311 | 1.91E-04 | GTExv8.EUR.Whole_Blood | 1.90E-03 | SLC5A6 | DBP |
| 3.3099 | 9.33E-04 | GTExv8.EUR.Whole_Blood | 7.03E-03 | CAD | DBP |
| 2.9273 | 3.42E-03 | GTExv8.EUR.Whole_Blood | 2.02E-02 | PPM1G | DBP |
| 2.7224 | 6.48E-03 | GTExv8.EUR.Whole_Blood | 3.35E-02 | NRBP1 | DBP |
| -3.4410 | 5.80E-04 | GTExv8.EUR.Whole_Blood | 4.80E-03 | KRTCAP3 | DBP |
| -2.6050 | 9.19E-03 | GTExv8.EUR.Whole_Blood | 4.42E-02 | SLC4A1AP | DBP |
| -3.2296 | 1.24E-03 | GTExv8.EUR.Whole_Blood | 8.88E-03 | PPP1CB | DBP |
| 3.1210 | 1.80E-03 | GTExv8.EUR.Whole_Blood | 1.20E-02 | TRMT61B | DBP |
| 2.9630 | 3.05E-03 | GTExv8.EUR.Whole_Blood | 1.84E-02 | STRN | DBP |
| 7.1336 | 9.78E-13 | GTExv8.EUR.Whole_Blood | 7.91E-11 | CEBPZOS | DBP |
| 5.8661 | 4.46E-09 | GTExv8.EUR.Whole_Blood | 1.51E-07 | NDUFAF7 | DBP |
| 4.8330 | 1.34E-06 | GTExv8.EUR.Whole_Blood | 2.48E-05 | PRKD3 | DBP |
| 5.8618 | 4.58E-09 | GTExv8.EUR.Whole_Blood | 1.54E-07 | QPCT | DBP |
| -4.2629 | 2.02E-05 | GTExv8.EUR.Whole_Blood | 2.79E-04 | CDC42EP3 | DBP |
| -4.1962 | 2.71E-05 | GTExv8.EUR.Whole_Blood | 3.60E-04 | AC010878.3 | DBP |
| -4.1002 | 4.13E-05 | GTExv8.EUR.Whole_Blood | 5.09E-04 | LINC00211 | DBP |
| -4.6085 | 4.06E-06 | GTExv8.EUR.Whole_Blood | 6.59E-05 | COX7A2L | DBP |
| -3.1952 | 1.40E-03 | GTExv8.EUR.Whole_Blood | 9.83E-03 | OXER1 | DBP |
| -5.9255 | 3.11E-09 | GTExv8.EUR.Whole_Blood | 1.09E-07 | HAAO | DBP |
| -3.3958 | 6.84E-04 | GTExv8.EUR.Whole_Blood | 5.49E-03 | LINC01126 | DBP |
| 4.8952 | 9.82E-07 | GTExv8.EUR.Whole_Blood | 1.90E-05 | PLEKHH2 | DBP |
| -3.1964 | 1.39E-03 | GTExv8.EUR.Whole_Blood | 9.76E-03 | LRPPRC | DBP |
| 5.5163 | 3.46E-08 | GTExv8.EUR.Whole_Blood | 9.26E-07 | PRKCE | DBP |
| -3.5006 | 4.64E-04 | GTExv8.EUR.Whole_Blood | 4.00E-03 | CHAC2 | DBP |
| -3.5896 | 3.31E-04 | GTExv8.EUR.Whole_Blood | 3.01E-03 | ACYP2 | DBP |
| -2.8386 | 4.53E-03 | GTExv8.EUR.Whole_Blood | 2.52E-02 | RTN4 | DBP |
| -3.4615 | 5.37E-04 | GTExv8.EUR.Whole_Blood | 4.54E-03 | PPP4R3B | DBP |
| 4.1980 | 2.69E-05 | GTExv8.EUR.Whole_Blood | 3.58E-04 | NONOP2 | DBP |
| 2.8330 | 4.61E-03 | GTExv8.EUR.Whole_Blood | 2.54E-02 | PUS10 | DBP |
| 6.2944 | 3.09E-10 | GTExv8.EUR.Whole_Blood | 1.35E-08 | AC016747.3 | DBP |
| -5.4644 | 4.65E-08 | GTExv8.EUR.Whole_Blood | 1.21E-06 | C2orf74 | DBP |
| 6.2096 | 5.31E-10 | GTExv8.EUR.Whole_Blood | 2.25E-08 | AHSA2 | DBP |
| -6.9182 | 4.57E-12 | GTExv8.EUR.Whole_Blood | 3.12E-10 | RP11-493E12.2 | DBP |
| -5.1990 | 2.00E-07 | GTExv8.EUR.Whole_Blood | 4.58E-06 | MDH1 | DBP |
| -3.8740 | 1.07E-04 | GTExv8.EUR.Whole_Blood | 1.17E-03 | AC016734.2 | DBP |
| 3.5940 | 3.26E-04 | GTExv8.EUR.Whole_Blood | 2.98E-03 | UGP2 | DBP |
| -2.6156 | 8.91E-03 | GTExv8.EUR.Whole_Blood | 4.32E-02 | AC074289.1 | DBP |
| 3.0092 | 2.62E-03 | GTExv8.EUR.Whole_Blood | 1.63E-02 | AC008074.5 | DBP |
| 3.4304 | 6.03E-04 | GTExv8.EUR.Whole_Blood | 4.96E-03 | CEP68 | DBP |
| 2.8425 | 4.48E-03 | GTExv8.EUR.Whole_Blood | 2.49E-02 | GFPT1 | DBP |
| -3.4343 | 5.94E-04 | GTExv8.EUR.Whole_Blood | 4.90E-03 | ASPRV1 | DBP |
| -2.8204 | 4.80E-03 | GTExv8.EUR.Whole_Blood | 2.63E-02 | PCBP1-AS1 | DBP |
| -5.8173 | 5.98E-09 | GTExv8.EUR.Whole_Blood | 1.93E-07 | CYP26B1 | DBP |
| 3.4553 | 5.50E-04 | GTExv8.EUR.Whole_Blood | 4.62E-03 | RAB11FIP5 | DBP |
| 5.9932 | 2.06E-09 | GTExv8.EUR.Whole_Blood | 7.49E-08 | ALMS1 | DBP |
| 3.0524 | 2.27E-03 | GTExv8.EUR.Whole_Blood | 1.45E-02 | NAT8B | DBP |
| 5.1805 | 2.21E-07 | GTExv8.EUR.Whole_Blood | 4.99E-06 | RP11-434P11.2 | DBP |
| 3.4946 | 4.75E-04 | GTExv8.EUR.Whole_Blood | 4.07E-03 | STAMBP | DBP |
| 3.5036 | 4.59E-04 | GTExv8.EUR.Whole_Blood | 3.97E-03 | TET3 | DBP |
| 2.7551 | 5.87E-03 | GTExv8.EUR.Whole_Blood | 3.09E-02 | INO80B | DBP |
| 2.6783 | 7.40E-03 | GTExv8.EUR.Whole_Blood | 3.71E-02 | WBP1 | DBP |
| -2.7045 | 6.84E-03 | GTExv8.EUR.Whole_Blood | 3.50E-02 | LBX2 | DBP |
| -2.7927 | 5.23E-03 | GTExv8.EUR.Whole_Blood | 2.82E-02 | LBX2-AS1 | DBP |
| -4.3064 | 1.66E-05 | GTExv8.EUR.Whole_Blood | 2.34E-04 | ELMOD3 | DBP |
| -2.8553 | 4.30E-03 | GTExv8.EUR.Whole_Blood | 2.40E-02 | GGCX | DBP |
| -5.2189 | 1.80E-07 | GTExv8.EUR.Whole_Blood | 4.15E-06 | AC012454.4 | DBP |
| -5.3278 | 9.94E-08 | GTExv8.EUR.Whole_Blood | 2.41E-06 | GNLY | DBP |
| -5.1410 | 2.73E-07 | GTExv8.EUR.Whole_Blood | 6.06E-06 | ATOH8 | DBP |
| -5.1374 | 2.79E-07 | GTExv8.EUR.Whole_Blood | 6.12E-06 | ST3GAL5 | DBP |
| -4.6390 | 3.50E-06 | GTExv8.EUR.Whole_Blood | 5.79E-05 | IMMT | DBP |
| 4.3213 | 1.55E-05 | GTExv8.EUR.Whole_Blood | 2.19E-04 | RP11-301O19.1 | DBP |
| -3.1513 | 1.63E-03 | GTExv8.EUR.Whole_Blood | 1.11E-02 | CD8A | DBP |
| 3.3344 | 8.55E-04 | GTExv8.EUR.Whole_Blood | 6.55E-03 | ZNF514 | DBP |
| 10.1260 | 4.24E-24 | GTExv8.EUR.Whole_Blood | 9.88E-22 | LINC00342 | DBP |
| 10.6750 | 1.33E-26 | GTExv8.EUR.Whole_Blood | 3.76E-24 | FAHD2CP | DBP |
| 10.2279 | 1.49E-24 | GTExv8.EUR.Whole_Blood | 3.58E-22 | TMEM127 | DBP |
| 7.7789 | 7.32E-15 | GTExv8.EUR.Whole_Blood | 7.95E-13 | CIAO1 | DBP |
| 3.7211 | 1.98E-04 | GTExv8.EUR.Whole_Blood | 1.96E-03 | ITPRIPL1 | DBP |
| 9.0537 | 1.38E-19 | GTExv8.EUR.Whole_Blood | 2.54E-17 | ARID5A | DBP |
| 6.5636 | 5.25E-11 | GTExv8.EUR.Whole_Blood | 2.81E-09 | LMAN2L | DBP |
| 5.6190 | 1.92E-08 | GTExv8.EUR.Whole_Blood | 5.51E-07 | CNNM4 | DBP |
| 4.0004 | 6.32E-05 | GTExv8.EUR.Whole_Blood | 7.47E-04 | FAHD2B | DBP |
| 5.1466 | 2.65E-07 | GTExv8.EUR.Whole_Blood | 5.90E-06 | ANKRD36 | DBP |
| -4.7400 | 2.14E-06 | GTExv8.EUR.Whole_Blood | 3.74E-05 | ANKRD36B | DBP |
| 5.2517 | 1.51E-07 | GTExv8.EUR.Whole_Blood | 3.54E-06 | ACTR1B | DBP |
| -2.8878 | 3.88E-03 | GTExv8.EUR.Whole_Blood | 2.23E-02 | LINC01125 | DBP |
| -5.7138 | 1.10E-08 | GTExv8.EUR.Whole_Blood | 3.29E-07 | TMEM131 | DBP |
| -3.8401 | 1.23E-04 | GTExv8.EUR.Whole_Blood | 1.32E-03 | IL18R1 | DBP |
| -3.0643 | 2.18E-03 | GTExv8.EUR.Whole_Blood | 1.40E-02 | SDR42E1P5 | DBP |
| 3.0711 | 2.13E-03 | GTExv8.EUR.Whole_Blood | 1.38E-02 | MRPS9 | DBP |
| -2.5751 | 1.00E-02 | GTExv8.EUR.Whole_Blood | 4.72E-02 | UXS1 | DBP |
| -3.5014 | 4.63E-04 | GTExv8.EUR.Whole_Blood | 3.99E-03 | TMEM87B | DBP |
| -3.0430 | 2.34E-03 | GTExv8.EUR.Whole_Blood | 1.48E-02 | RGPD8 | DBP |
| 3.9661 | 7.31E-05 | GTExv8.EUR.Whole_Blood | 8.44E-04 | AC079922.3 | DBP |
| 3.9424 | 8.07E-05 | GTExv8.EUR.Whole_Blood | 9.16E-04 | SLC20A1 | DBP |
| -3.4342 | 5.94E-04 | GTExv8.EUR.Whole_Blood | 4.90E-03 | WASH2P | DBP |
| 4.1309 | 3.61E-05 | GTExv8.EUR.Whole_Blood | 4.56E-04 | DDX11L2 | DBP |
| 3.6229 | 2.91E-04 | GTExv8.EUR.Whole_Blood | 2.71E-03 | RP11-395L14.18 | DBP |
| 4.1457 | 3.39E-05 | GTExv8.EUR.Whole_Blood | 4.34E-04 | RPL23AP7 | DBP |
| -3.4073 | 6.56E-04 | GTExv8.EUR.Whole_Blood | 5.30E-03 | RABL2A | DBP |
| 3.3220 | 8.94E-04 | GTExv8.EUR.Whole_Blood | 6.79E-03 | AC017074.2 | DBP |
| 3.2130 | 1.31E-03 | GTExv8.EUR.Whole_Blood | 9.31E-03 | LINC01191 | DBP |
| 3.1181 | 1.82E-03 | GTExv8.EUR.Whole_Blood | 1.21E-02 | BIN1 | DBP |
| 3.6297 | 2.84E-04 | GTExv8.EUR.Whole_Blood | 2.66E-03 | CCDC115 | DBP |
| 3.4327 | 5.98E-04 | GTExv8.EUR.Whole_Blood | 4.93E-03 | IMP4 | DBP |
| 5.1191 | 3.07E-07 | GTExv8.EUR.Whole_Blood | 6.70E-06 | TMEM163 | DBP |
| 6.6660 | 2.63E-11 | GTExv8.EUR.Whole_Blood | 1.53E-09 | CCNT2-AS1 | DBP |
| -6.0277 | 1.66E-09 | GTExv8.EUR.Whole_Blood | 6.12E-08 | CCNT2 | DBP |
| 3.8987 | 9.67E-05 | GTExv8.EUR.Whole_Blood | 1.07E-03 | DARS-AS1 | DBP |
| -3.4851 | 4.92E-04 | GTExv8.EUR.Whole_Blood | 4.19E-03 | DARS | DBP |
| 4.4887 | 7.17E-06 | GTExv8.EUR.Whole_Blood | 1.11E-04 | ACVR2A | DBP |
| 3.3473 | 8.16E-04 | GTExv8.EUR.Whole_Blood | 6.35E-03 | MBD5 | DBP |
| 2.5980 | 9.38E-03 | GTExv8.EUR.Whole_Blood | 4.50E-02 | HAT1 | DBP |
| 3.4124 | 6.44E-04 | GTExv8.EUR.Whole_Blood | 5.23E-03 | SLC25A12 | DBP |
| -3.5004 | 4.65E-04 | GTExv8.EUR.Whole_Blood | 4.00E-03 | MAP3K20 | DBP |
| -3.8899 | 1.00E-04 | GTExv8.EUR.Whole_Blood | 1.10E-03 | OLA1 | DBP |
| -3.3909 | 6.97E-04 | GTExv8.EUR.Whole_Blood | 5.57E-03 | LNPK | DBP |
| 3.6385 | 2.74E-04 | GTExv8.EUR.Whole_Blood | 2.58E-03 | RBM45 | DBP |
| 4.1332 | 3.58E-05 | GTExv8.EUR.Whole_Blood | 4.55E-04 | PLEKHA3 | DBP |
| 4.7656 | 1.88E-06 | GTExv8.EUR.Whole_Blood | 3.34E-05 | RP11-171I2.2 | DBP |
| -6.9567 | 3.48E-12 | GTExv8.EUR.Whole_Blood | 2.48E-10 | UBE2E3 | DBP |
| 3.4292 | 6.05E-04 | GTExv8.EUR.Whole_Blood | 4.96E-03 | CALCRL | DBP |
| -3.4309 | 6.02E-04 | GTExv8.EUR.Whole_Blood | 4.95E-03 | ASNSD1 | DBP |
| -3.3364 | 8.49E-04 | GTExv8.EUR.Whole_Blood | 6.52E-03 | RP11-455J20.3 | DBP |
| -6.1450 | 7.99E-10 | GTExv8.EUR.Whole_Blood | 3.20E-08 | OSGEPL1-AS1 | DBP |
| 2.9721 | 2.96E-03 | GTExv8.EUR.Whole_Blood | 1.79E-02 | ORMDL1 | DBP |
| 2.9048 | 3.67E-03 | GTExv8.EUR.Whole_Blood | 2.14E-02 | PMS1 | DBP |
| -5.5419 | 2.99E-08 | GTExv8.EUR.Whole_Blood | 8.17E-07 | C2orf88 | DBP |
| 4.2021 | 2.65E-05 | GTExv8.EUR.Whole_Blood | 3.54E-04 | HIBCH | DBP |
| -6.2250 | 4.82E-10 | GTExv8.EUR.Whole_Blood | 2.05E-08 | RP11-647K16.1 | DBP |
| -7.7303 | 1.07E-14 | GTExv8.EUR.Whole_Blood | 1.13E-12 | GLS | DBP |
| -3.0666 | 2.17E-03 | GTExv8.EUR.Whole_Blood | 1.40E-02 | ANKRD44 | DBP |
| -3.0038 | 2.67E-03 | GTExv8.EUR.Whole_Blood | 1.65E-02 | FTCDNL1 | DBP |
| 3.1115 | 1.86E-03 | GTExv8.EUR.Whole_Blood | 1.24E-02 | C2orf69 | DBP |
| 2.7783 | 5.46E-03 | GTExv8.EUR.Whole_Blood | 2.93E-02 | MAIP1 | DBP |
| 3.4506 | 5.59E-04 | GTExv8.EUR.Whole_Blood | 4.67E-03 | SPATS2L | DBP |
| -2.9426 | 3.25E-03 | GTExv8.EUR.Whole_Blood | 1.94E-02 | STRADB | DBP |
| 5.1809 | 2.21E-07 | GTExv8.EUR.Whole_Blood | 4.99E-06 | FAM117B | DBP |
| 3.6858 | 2.28E-04 | GTExv8.EUR.Whole_Blood | 2.22E-03 | ICA1L | DBP |
| -4.9820 | 6.29E-07 | GTExv8.EUR.Whole_Blood | 1.27E-05 | CREB1 | DBP |
| 6.2738 | 3.52E-10 | GTExv8.EUR.Whole_Blood | 1.53E-08 | LINC01857 | DBP |
| 2.6940 | 7.06E-03 | GTExv8.EUR.Whole_Blood | 3.58E-02 | RPE | DBP |
| 5.8003 | 6.62E-09 | GTExv8.EUR.Whole_Blood | 2.09E-07 | TNS1 | DBP |
| -2.5656 | 1.03E-02 | GTExv8.EUR.Whole_Blood | 4.83E-02 | CXCR2 | DBP |
| -3.4525 | 5.56E-04 | GTExv8.EUR.Whole_Blood | 4.65E-03 | ARPC2 | DBP |
| -3.4092 | 6.52E-04 | GTExv8.EUR.Whole_Blood | 5.28E-03 | RP11-378A13.1 | DBP |
| 3.6417 | 2.71E-04 | GTExv8.EUR.Whole_Blood | 2.57E-03 | GPBAR1 | DBP |
| -3.3094 | 9.35E-04 | GTExv8.EUR.Whole_Blood | 7.04E-03 | PNKD | DBP |
| -6.5414 | 6.09E-11 | GTExv8.EUR.Whole_Blood | 3.20E-09 | ZNF142 | DBP |
| -4.4144 | 1.01E-05 | GTExv8.EUR.Whole_Blood | 1.48E-04 | CYP27A1 | DBP |
| -4.3120 | 1.62E-05 | GTExv8.EUR.Whole_Blood | 2.28E-04 | RP11-459I19.1 | DBP |
| 3.0555 | 2.25E-03 | GTExv8.EUR.Whole_Blood | 1.44E-02 | FAM134A | DBP |
| 3.0602 | 2.21E-03 | GTExv8.EUR.Whole_Blood | 1.42E-02 | AC017104.6 | DBP |
| 3.0078 | 2.63E-03 | GTExv8.EUR.Whole_Blood | 1.63E-02 | PDE6D | DBP |
| -3.3557 | 7.92E-04 | GTExv8.EUR.Whole_Blood | 6.18E-03 | AC096574.5 | DBP |
| 2.6310 | 8.51E-03 | GTExv8.EUR.Whole_Blood | 4.17E-02 | RBM44 | DBP |
| -2.7792 | 5.45E-03 | GTExv8.EUR.Whole_Blood | 2.92E-02 | ASB1 | DBP |
| 3.4723 | 5.16E-04 | GTExv8.EUR.Whole_Blood | 4.37E-03 | GPR35 | DBP |
| 4.0330 | 5.51E-05 | GTExv8.EUR.Whole_Blood | 6.61E-04 | FARP2 | DBP |
| -2.9318 | 3.37E-03 | GTExv8.EUR.Whole_Blood | 2.00E-02 | PDCD1 | DBP |
| 3.2391 | 1.20E-03 | GTExv8.EUR.Whole_Blood | 8.62E-03 | SUMF1 | DBP |
| 3.5120 | 4.45E-04 | GTExv8.EUR.Whole_Blood | 3.87E-03 | TATDN2 | DBP |
| 2.9012 | 3.72E-03 | GTExv8.EUR.Whole_Blood | 2.16E-02 | LINC00852 | DBP |
| 2.9066 | 3.65E-03 | GTExv8.EUR.Whole_Blood | 2.13E-02 | GHRLOS | DBP |
| 4.8824 | 1.05E-06 | GTExv8.EUR.Whole_Blood | 2.01E-05 | RP11-169K17.3 | DBP |
| 2.9567 | 3.11E-03 | GTExv8.EUR.Whole_Blood | 1.87E-02 | VGLL4 | DBP |
| -3.7877 | 1.52E-04 | GTExv8.EUR.Whole_Blood | 1.58E-03 | METTL6 | DBP |
| -2.7880 | 5.30E-03 | GTExv8.EUR.Whole_Blood | 2.85E-02 | DPH3 | DBP |
| -2.8475 | 4.41E-03 | GTExv8.EUR.Whole_Blood | 2.46E-02 | PDCD6IP | DBP |
| -3.6043 | 3.13E-04 | GTExv8.EUR.Whole_Blood | 2.88E-03 | LRRFIP2 | DBP |
| -2.9453 | 3.23E-03 | GTExv8.EUR.Whole_Blood | 1.93E-02 | EIF1B-AS1 | DBP |
| -3.3239 | 8.88E-04 | GTExv8.EUR.Whole_Blood | 6.75E-03 | ENTPD3-AS1 | DBP |
| -3.2167 | 1.30E-03 | GTExv8.EUR.Whole_Blood | 9.25E-03 | RPL14 | DBP |
| -5.9532 | 2.63E-09 | GTExv8.EUR.Whole_Blood | 9.30E-08 | CTNNB1 | DBP |
| 13.8480 | 1.31E-43 | GTExv8.EUR.Whole_Blood | 1.48E-40 | ULK4 | DBP |
| -5.4020 | 6.59E-08 | GTExv8.EUR.Whole_Blood | 1.65E-06 | ELP6 | DBP |
| 10.9685 | 5.42E-28 | GTExv8.EUR.Whole_Blood | 1.72E-25 | MAP4 | DBP |
| -11.4830 | 1.61E-30 | GTExv8.EUR.Whole_Blood | 6.38E-28 | ZNF589 | DBP |
| -11.6268 | 3.01E-31 | GTExv8.EUR.Whole_Blood | 1.33E-28 | MRPS18AP1 | DBP |
| 11.4737 | 1.79E-30 | GTExv8.EUR.Whole_Blood | 6.75E-28 | FCF1P2 | DBP |
| 10.0478 | 9.40E-24 | GTExv8.EUR.Whole_Blood | 2.07E-21 | NME6 | DBP |
| -12.1525 | 5.57E-34 | GTExv8.EUR.Whole_Blood | 2.76E-31 | SPINK8 | DBP |
| 7.9801 | 1.46E-15 | GTExv8.EUR.Whole_Blood | 1.81E-13 | SHISA5 | DBP |
| -2.6634 | 7.74E-03 | GTExv8.EUR.Whole_Blood | 3.86E-02 | NCKIPSD | DBP |
| 4.5906 | 4.42E-06 | GTExv8.EUR.Whole_Blood | 7.15E-05 | IP6K2 | DBP |
| 2.9720 | 2.96E-03 | GTExv8.EUR.Whole_Blood | 1.79E-02 | WDR6 | DBP |
| -5.8703 | 4.35E-09 | GTExv8.EUR.Whole_Blood | 1.49E-07 | DAG1 | DBP |
| -3.6526 | 2.60E-04 | GTExv8.EUR.Whole_Blood | 2.49E-03 | APEH | DBP |
| -4.6970 | 2.64E-06 | GTExv8.EUR.Whole_Blood | 4.51E-05 | MST1 | DBP |
| -6.1310 | 8.73E-10 | GTExv8.EUR.Whole_Blood | 3.44E-08 | UBA7 | DBP |
| -4.4702 | 7.81E-06 | GTExv8.EUR.Whole_Blood | 1.18E-04 | MST1R | DBP |
| -4.3319 | 1.48E-05 | GTExv8.EUR.Whole_Blood | 2.10E-04 | MON1A | DBP |
| -3.9093 | 9.26E-05 | GTExv8.EUR.Whole_Blood | 1.04E-03 | RBM6 | DBP |
| -5.4996 | 3.81E-08 | GTExv8.EUR.Whole_Blood | 1.01E-06 | MAPKAPK3 | DBP |
| -5.1464 | 2.65E-07 | GTExv8.EUR.Whole_Blood | 5.90E-06 | LINC02019 | DBP |
| 3.0987 | 1.94E-03 | GTExv8.EUR.Whole_Blood | 1.28E-02 | TEX264 | DBP |
| 3.0941 | 1.97E-03 | GTExv8.EUR.Whole_Blood | 1.30E-02 | GRM2 | DBP |
| 3.1501 | 1.63E-03 | GTExv8.EUR.Whole_Blood | 1.11E-02 | ACTR8 | DBP |
| 2.6299 | 8.54E-03 | GTExv8.EUR.Whole_Blood | 4.18E-02 | ARF4 | DBP |
| 6.0392 | 1.55E-09 | GTExv8.EUR.Whole_Blood | 5.77E-08 | FLNB | DBP |
| 2.9901 | 2.79E-03 | GTExv8.EUR.Whole_Blood | 1.71E-02 | RPP14 | DBP |
| -3.2727 | 1.07E-03 | GTExv8.EUR.Whole_Blood | 7.89E-03 | C3orf14 | DBP |
| 2.8629 | 4.20E-03 | GTExv8.EUR.Whole_Blood | 2.36E-02 | SCAANT1 | DBP |
| 2.5685 | 1.02E-02 | GTExv8.EUR.Whole_Blood | 4.80E-02 | PSMD6-AS2 | DBP |
| 5.7777 | 7.57E-09 | GTExv8.EUR.Whole_Blood | 2.33E-07 | PSMD6-AS1 | DBP |
| 3.4420 | 5.77E-04 | GTExv8.EUR.Whole_Blood | 4.78E-03 | PROK2 | DBP |
| 2.7734 | 5.55E-03 | GTExv8.EUR.Whole_Blood | 2.97E-02 | LINC00877 | DBP |
| -4.5540 | 5.26E-06 | GTExv8.EUR.Whole_Blood | 8.32E-05 | C3orf38 | DBP |
| -3.6101 | 3.06E-04 | GTExv8.EUR.Whole_Blood | 2.83E-03 | IMPG2 | DBP |
| -6.3660 | 1.94E-10 | GTExv8.EUR.Whole_Blood | 8.94E-09 | SENP7 | DBP |
| -5.1061 | 3.29E-07 | GTExv8.EUR.Whole_Blood | 7.14E-06 | PCNP | DBP |
| 3.7134 | 2.04E-04 | GTExv8.EUR.Whole_Blood | 2.01E-03 | ZBTB11-AS1 | DBP |
| 3.4863 | 4.90E-04 | GTExv8.EUR.Whole_Blood | 4.18E-03 | CD47 | DBP |
| 3.3423 | 8.31E-04 | GTExv8.EUR.Whole_Blood | 6.42E-03 | IFT57 | DBP |
| 4.1152 | 3.87E-05 | GTExv8.EUR.Whole_Blood | 4.86E-04 | HHLA2 | DBP |
| 2.7882 | 5.30E-03 | GTExv8.EUR.Whole_Blood | 2.85E-02 | RP11-435F17.3 | DBP |
| -2.6333 | 8.46E-03 | GTExv8.EUR.Whole_Blood | 4.15E-02 | ARHGAP31 | DBP |
| -3.5581 | 3.74E-04 | GTExv8.EUR.Whole_Blood | 3.34E-03 | POGLUT1 | DBP |
| 2.7474 | 6.01E-03 | GTExv8.EUR.Whole_Blood | 3.14E-02 | POPDC2 | DBP |
| -3.8627 | 1.12E-04 | GTExv8.EUR.Whole_Blood | 1.21E-03 | FBXO40 | DBP |
| 3.1787 | 1.48E-03 | GTExv8.EUR.Whole_Blood | 1.03E-02 | RN7SL172P | DBP |
| -2.5971 | 9.40E-03 | GTExv8.EUR.Whole_Blood | 4.51E-02 | CD86 | DBP |
| 4.3631 | 1.28E-05 | GTExv8.EUR.Whole_Blood | 1.84E-04 | CSTA | DBP |
| -4.6271 | 3.71E-06 | GTExv8.EUR.Whole_Blood | 6.07E-05 | FAM162A | DBP |
| 2.5667 | 1.03E-02 | GTExv8.EUR.Whole_Blood | 4.83E-02 | HACD2 | DBP |
| -3.7851 | 1.54E-04 | GTExv8.EUR.Whole_Blood | 1.60E-03 | TPRA1 | DBP |
| -2.9954 | 2.74E-03 | GTExv8.EUR.Whole_Blood | 1.69E-02 | ABTB1 | DBP |
| 2.8525 | 4.34E-03 | GTExv8.EUR.Whole_Blood | 2.43E-02 | EEFSEC | DBP |
| -5.0920 | 3.54E-07 | GTExv8.EUR.Whole_Blood | 7.60E-06 | CEP63 | DBP |
| -5.1393 | 2.76E-07 | GTExv8.EUR.Whole_Blood | 6.10E-06 | ANAPC13 | DBP |
| -3.4200 | 6.26E-04 | GTExv8.EUR.Whole_Blood | 5.10E-03 | IL20RB | DBP |
| 7.8432 | 4.39E-15 | GTExv8.EUR.Whole_Blood | 4.90E-13 | ZBTB38 | DBP |
| -3.7627 | 1.68E-04 | GTExv8.EUR.Whole_Blood | 1.70E-03 | RP11-651P23.4 | DBP |
| -3.4532 | 5.54E-04 | GTExv8.EUR.Whole_Blood | 4.64E-03 | EIF2A | DBP |
| 6.1883 | 6.08E-10 | GTExv8.EUR.Whole_Blood | 2.54E-08 | DHX36 | DBP |
| -3.2441 | 1.18E-03 | GTExv8.EUR.Whole_Blood | 8.52E-03 | RSRC1 | DBP |
| -5.0053 | 5.58E-07 | GTExv8.EUR.Whole_Blood | 1.13E-05 | MFSD1 | DBP |
| 3.1986 | 1.38E-03 | GTExv8.EUR.Whole_Blood | 9.71E-03 | NMD3 | DBP |
| -4.4352 | 9.20E-06 | GTExv8.EUR.Whole_Blood | 1.37E-04 | YEATS2-AS1 | DBP |
| -2.6469 | 8.12E-03 | GTExv8.EUR.Whole_Blood | 4.02E-02 | PARL | DBP |
| 3.0245 | 2.49E-03 | GTExv8.EUR.Whole_Blood | 1.56E-02 | ABCC5 | DBP |
| 3.0720 | 2.13E-03 | GTExv8.EUR.Whole_Blood | 1.38E-02 | DVL3 | DBP |
| -3.2831 | 1.03E-03 | GTExv8.EUR.Whole_Blood | 7.64E-03 | PSMD2 | DBP |
| 2.7094 | 6.74E-03 | GTExv8.EUR.Whole_Blood | 3.45E-02 | LINC00884 | DBP |
| 2.7578 | 5.82E-03 | GTExv8.EUR.Whole_Blood | 3.07E-02 | ATP13A3 | DBP |
| -4.3666 | 1.26E-05 | GTExv8.EUR.Whole_Blood | 1.82E-04 | TMEM44-AS1 | DBP |
| -4.8949 | 9.83E-07 | GTExv8.EUR.Whole_Blood | 1.90E-05 | TMEM44 | DBP |
| -2.8609 | 4.22E-03 | GTExv8.EUR.Whole_Blood | 2.37E-02 | FAM43A | DBP |
| -3.6648 | 2.48E-04 | GTExv8.EUR.Whole_Blood | 2.39E-03 | Y_RNA | DBP |
| -3.1885 | 1.43E-03 | GTExv8.EUR.Whole_Blood | 9.99E-03 | SLC51A | DBP |
| -4.0284 | 5.62E-05 | GTExv8.EUR.Whole_Blood | 6.72E-04 | TCTEX1D2 | DBP |
| -5.8177 | 5.97E-09 | GTExv8.EUR.Whole_Blood | 1.93E-07 | TM4SF19-AS1 | DBP |
| -5.0061 | 5.56E-07 | GTExv8.EUR.Whole_Blood | 1.13E-05 | TM4SF19 | DBP |
| -5.6024 | 2.11E-08 | GTExv8.EUR.Whole_Blood | 5.99E-07 | UBXN7 | DBP |
| 3.2591 | 1.12E-03 | GTExv8.EUR.Whole_Blood | 8.16E-03 | TACC3 | DBP |
| -2.7244 | 6.44E-03 | GTExv8.EUR.Whole_Blood | 3.33E-02 | RP11-689P11.2 | DBP |
| -4.0672 | 4.76E-05 | GTExv8.EUR.Whole_Blood | 5.79E-04 | CPEB2 | DBP |
| 3.0576 | 2.23E-03 | GTExv8.EUR.Whole_Blood | 1.43E-02 | TAPT1-AS1 | DBP |
| 3.0475 | 2.31E-03 | GTExv8.EUR.Whole_Blood | 1.47E-02 | LAP3 | DBP |
| -5.0773 | 3.83E-07 | GTExv8.EUR.Whole_Blood | 8.18E-06 | DCAF16 | DBP |
| -3.7800 | 1.57E-04 | GTExv8.EUR.Whole_Blood | 1.62E-03 | SMIM20 | DBP |
| 4.0470 | 5.19E-05 | GTExv8.EUR.Whole_Blood | 6.26E-04 | ARAP2 | DBP |
| 3.5054 | 4.56E-04 | GTExv8.EUR.Whole_Blood | 3.94E-03 | KLF3-AS1 | DBP |
| 2.7984 | 5.14E-03 | GTExv8.EUR.Whole_Blood | 2.78E-02 | TLR10 | DBP |
| 3.4775 | 5.06E-04 | GTExv8.EUR.Whole_Blood | 4.30E-03 | APBB2 | DBP |
| -3.6118 | 3.04E-04 | GTExv8.EUR.Whole_Blood | 2.81E-03 | OCIAD1 | DBP |
| -5.1016 | 3.37E-07 | GTExv8.EUR.Whole_Blood | 7.30E-06 | SRD5A3 | DBP |
| -7.8554 | 3.99E-15 | GTExv8.EUR.Whole_Blood | 4.52E-13 | SRD5A3-AS1 | DBP |
| -5.8257 | 5.69E-09 | GTExv8.EUR.Whole_Blood | 1.86E-07 | TMEM165 | DBP |
| -5.8696 | 4.37E-09 | GTExv8.EUR.Whole_Blood | 1.49E-07 | CLOCK | DBP |
| -2.9111 | 3.60E-03 | GTExv8.EUR.Whole_Blood | 2.11E-02 | HOPX | DBP |
| -3.1588 | 1.58E-03 | GTExv8.EUR.Whole_Blood | 1.09E-02 | NUP54 | DBP |
| -3.9670 | 7.28E-05 | GTExv8.EUR.Whole_Blood | 8.42E-04 | SCARB2 | DBP |
| -2.9116 | 3.60E-03 | GTExv8.EUR.Whole_Blood | 2.11E-02 | FAM13A-AS1 | DBP |
| -2.7297 | 6.34E-03 | GTExv8.EUR.Whole_Blood | 3.29E-02 | FAM13A | DBP |
| 4.5138 | 6.37E-06 | GTExv8.EUR.Whole_Blood | 9.92E-05 | FLJ20021 | DBP |
| 5.5089 | 3.61E-08 | GTExv8.EUR.Whole_Blood | 9.60E-07 | SLC39A8 | DBP |
| 7.9016 | 2.75E-15 | GTExv8.EUR.Whole_Blood | 3.20E-13 | KRT8P46 | DBP |
| 7.9016 | 2.75E-15 | GTExv8.EUR.Whole_Blood | 3.20E-13 | LRRC37A15P | DBP |
| 7.1134 | 1.13E-12 | GTExv8.EUR.Whole_Blood | 9.04E-11 | RP11-10L12.1 | DBP |
| 7.9016 | 2.75E-15 | GTExv8.EUR.Whole_Blood | 3.20E-13 | RP11-10L12.2 | DBP |
| 4.5529 | 5.29E-06 | GTExv8.EUR.Whole_Blood | 8.35E-05 | MANBA | DBP |
| -4.7314 | 2.23E-06 | GTExv8.EUR.Whole_Blood | 3.88E-05 | BDH2 | DBP |
| 4.4493 | 8.61E-06 | GTExv8.EUR.Whole_Blood | 1.29E-04 | HADH | DBP |
| -6.5854 | 4.54E-11 | GTExv8.EUR.Whole_Blood | 2.52E-09 | C4orf3 | DBP |
| 3.3427 | 8.30E-04 | GTExv8.EUR.Whole_Blood | 6.42E-03 | FABP2 | DBP |
| -6.2979 | 3.02E-10 | GTExv8.EUR.Whole_Blood | 1.34E-08 | GTF2IP12 | DBP |
| 5.5864 | 2.32E-08 | GTExv8.EUR.Whole_Blood | 6.52E-07 | RP11-33B1.1 | DBP |
| 2.9297 | 3.39E-03 | GTExv8.EUR.Whole_Blood | 2.00E-02 | PRDM5 | DBP |
| -2.7571 | 5.83E-03 | GTExv8.EUR.Whole_Blood | 3.07E-02 | KIAA1109 | DBP |
| -3.1532 | 1.61E-03 | GTExv8.EUR.Whole_Blood | 1.10E-02 | RP11-223C24.1 | DBP |
| -2.8692 | 4.11E-03 | GTExv8.EUR.Whole_Blood | 2.33E-02 | INPP4B | DBP |
| -6.6814 | 2.37E-11 | GTExv8.EUR.Whole_Blood | 1.39E-09 | USP38 | DBP |
| 2.6193 | 8.81E-03 | GTExv8.EUR.Whole_Blood | 4.28E-02 | GUSBP5 | DBP |
| -2.8989 | 3.74E-03 | GTExv8.EUR.Whole_Blood | 2.17E-02 | DCLK2 | DBP |
| -4.8259 | 1.39E-06 | GTExv8.EUR.Whole_Blood | 2.56E-05 | RPS3A | DBP |
| -3.8485 | 1.19E-04 | GTExv8.EUR.Whole_Blood | 1.28E-03 | RP11-372K14.2 | DBP |
| -5.1897 | 2.11E-07 | GTExv8.EUR.Whole_Blood | 4.80E-06 | FAM160A1 | DBP |
| -4.6245 | 3.76E-06 | GTExv8.EUR.Whole_Blood | 6.13E-05 | RP11-164P12.5 | DBP |
| -4.3530 | 1.34E-05 | GTExv8.EUR.Whole_Blood | 1.92E-04 | CTSO | DBP |
| -3.7666 | 1.65E-04 | GTExv8.EUR.Whole_Blood | 1.68E-03 | CCDC127 | DBP |
| -3.9784 | 6.94E-05 | GTExv8.EUR.Whole_Blood | 8.12E-04 | CTD-2083E4.6 | DBP |
| 3.4912 | 4.81E-04 | GTExv8.EUR.Whole_Blood | 4.11E-03 | AHRR | DBP |
| -3.0270 | 2.47E-03 | GTExv8.EUR.Whole_Blood | 1.55E-02 | ANKH | DBP |
| 4.6760 | 2.93E-06 | GTExv8.EUR.Whole_Blood | 4.96E-05 | CTD-2124B8.2 | DBP |
| 4.1751 | 2.98E-05 | GTExv8.EUR.Whole_Blood | 3.87E-04 | WDR70 | DBP |
| 5.3340 | 9.61E-08 | GTExv8.EUR.Whole_Blood | 2.34E-06 | MAP3K1 | DBP |
| 3.9279 | 8.57E-05 | GTExv8.EUR.Whole_Blood | 9.66E-04 | SETD9 | DBP |
| 4.5576 | 5.17E-06 | GTExv8.EUR.Whole_Blood | 8.19E-05 | CTD-2310F14.1 | DBP |
| -3.3896 | 7.00E-04 | GTExv8.EUR.Whole_Blood | 5.59E-03 | GAPT | DBP |
| 2.8025 | 5.07E-03 | GTExv8.EUR.Whole_Blood | 2.76E-02 | CD180 | DBP |
| 4.2844 | 1.83E-05 | GTExv8.EUR.Whole_Blood | 2.56E-04 | ANKRA2 | DBP |
| 5.0524 | 4.36E-07 | GTExv8.EUR.Whole_Blood | 9.14E-06 | POC5 | DBP |
| 2.7610 | 5.76E-03 | GTExv8.EUR.Whole_Blood | 3.05E-02 | TMEM161B-AS1 | DBP |
| -2.8150 | 4.88E-03 | GTExv8.EUR.Whole_Blood | 2.67E-02 | CAST | DBP |
| -5.6218 | 1.89E-08 | GTExv8.EUR.Whole_Blood | 5.47E-07 | CTD-2260A17.3 | DBP |
| -6.5638 | 5.25E-11 | GTExv8.EUR.Whole_Blood | 2.81E-09 | ERAP1 | DBP |
| 6.9897 | 2.76E-12 | GTExv8.EUR.Whole_Blood | 2.04E-10 | ERAP2 | DBP |
| 5.9830 | 2.19E-09 | GTExv8.EUR.Whole_Blood | 7.89E-08 | LNPEP | DBP |
| 2.8885 | 3.87E-03 | GTExv8.EUR.Whole_Blood | 2.23E-02 | LINC02062 | DBP |
| 2.6302 | 8.53E-03 | GTExv8.EUR.Whole_Blood | 4.18E-02 | SLCO4C1 | DBP |
| -4.9009 | 9.54E-07 | GTExv8.EUR.Whole_Blood | 1.86E-05 | NREP | DBP |
| -4.4654 | 7.99E-06 | GTExv8.EUR.Whole_Blood | 1.21E-04 | APC | DBP |
| 3.8212 | 1.33E-04 | GTExv8.EUR.Whole_Blood | 1.41E-03 | CTC-487M23.5 | DBP |
| 4.5672 | 4.94E-06 | GTExv8.EUR.Whole_Blood | 7.88E-05 | MCC | DBP |
| 4.7253 | 2.30E-06 | GTExv8.EUR.Whole_Blood | 3.98E-05 | TNFAIP8 | DBP |
| -8.8548 | 8.38E-19 | GTExv8.EUR.Whole_Blood | 1.36E-16 | KRT8P33 | DBP |
| 3.6953 | 2.20E-04 | GTExv8.EUR.Whole_Blood | 2.15E-03 | LINC01184 | DBP |
| 4.8382 | 1.31E-06 | GTExv8.EUR.Whole_Blood | 2.43E-05 | FBN2 | DBP |
| 7.6704 | 1.71E-14 | GTExv8.EUR.Whole_Blood | 1.76E-12 | SLC22A4 | DBP |
| -5.7966 | 6.77E-09 | GTExv8.EUR.Whole_Blood | 2.13E-07 | AC034220.3 | DBP |
| 8.1193 | 4.69E-16 | GTExv8.EUR.Whole_Blood | 5.99E-14 | SLC22A5 | DBP |
| -6.3730 | 1.85E-10 | GTExv8.EUR.Whole_Blood | 8.57E-09 | C5orf56 | DBP |
| -9.5742 | 1.03E-21 | GTExv8.EUR.Whole_Blood | 2.04E-19 | AC116366.6 | DBP |
| -2.9447 | 3.23E-03 | GTExv8.EUR.Whole_Blood | 1.93E-02 | AFF4 | DBP |
| 6.0995 | 1.06E-09 | GTExv8.EUR.Whole_Blood | 4.04E-08 | HSPA4 | DBP |
| -2.8976 | 3.76E-03 | GTExv8.EUR.Whole_Blood | 2.17E-02 | SAR1B | DBP |
| -3.0729 | 2.12E-03 | GTExv8.EUR.Whole_Blood | 1.37E-02 | CAMLG | DBP |
| -3.2223 | 1.27E-03 | GTExv8.EUR.Whole_Blood | 9.06E-03 | CTNNA1 | DBP |
| 4.3972 | 1.10E-05 | GTExv8.EUR.Whole_Blood | 1.60E-04 | PROB1 | DBP |
| -4.3722 | 1.23E-05 | GTExv8.EUR.Whole_Blood | 1.78E-04 | SPATA24 | DBP |
| -4.5756 | 4.75E-06 | GTExv8.EUR.Whole_Blood | 7.59E-05 | DNAJC18 | DBP |
| 3.5804 | 3.43E-04 | GTExv8.EUR.Whole_Blood | 3.10E-03 | SLC4A9 | DBP |
| 4.7320 | 2.22E-06 | GTExv8.EUR.Whole_Blood | 3.87E-05 | TMCO6 | DBP |
| 3.4732 | 5.14E-04 | GTExv8.EUR.Whole_Blood | 4.36E-03 | IK | DBP |
| -4.2558 | 2.08E-05 | GTExv8.EUR.Whole_Blood | 2.87E-04 | NDUFA2 | DBP |
| 4.3943 | 1.11E-05 | GTExv8.EUR.Whole_Blood | 1.61E-04 | WDR55 | DBP |
| -2.9864 | 2.82E-03 | GTExv8.EUR.Whole_Blood | 1.73E-02 | KIAA0141 | DBP |
| -2.9298 | 3.39E-03 | GTExv8.EUR.Whole_Blood | 2.00E-02 | YIPF5 | DBP |
| 3.4280 | 6.08E-04 | GTExv8.EUR.Whole_Blood | 4.98E-03 | PCYOX1L | DBP |
| 2.7072 | 6.79E-03 | GTExv8.EUR.Whole_Blood | 3.48E-02 | ATOX1 | DBP |
| 3.3328 | 8.60E-04 | GTExv8.EUR.Whole_Blood | 6.58E-03 | SAP30L | DBP |
| 3.7643 | 1.67E-04 | GTExv8.EUR.Whole_Blood | 1.70E-03 | CTB-109A12.1 | DBP |
| 2.9391 | 3.29E-03 | GTExv8.EUR.Whole_Blood | 1.96E-02 | CLINT1 | DBP |
| -3.3422 | 8.31E-04 | GTExv8.EUR.Whole_Blood | 6.42E-03 | UBTD2 | DBP |
| -6.1609 | 7.23E-10 | GTExv8.EUR.Whole_Blood | 2.92E-08 | CPEB4 | DBP |
| -10.6187 | 2.44E-26 | GTExv8.EUR.Whole_Blood | 6.24E-24 | HMP19 | DBP |
| 3.1290 | 1.75E-03 | GTExv8.EUR.Whole_Blood | 1.18E-02 | FAM153B | DBP |
| -5.4970 | 3.86E-08 | GTExv8.EUR.Whole_Blood | 1.02E-06 | RAB24 | DBP |
| 5.7413 | 9.40E-09 | GTExv8.EUR.Whole_Blood | 2.84E-07 | PRELID1 | DBP |
| -5.8346 | 5.39E-09 | GTExv8.EUR.Whole_Blood | 1.77E-07 | MXD3 | DBP |
| 3.2852 | 1.02E-03 | GTExv8.EUR.Whole_Blood | 7.59E-03 | LMAN2 | DBP |
| -3.5678 | 3.60E-04 | GTExv8.EUR.Whole_Blood | 3.23E-03 | DBN1 | DBP |
| -3.0144 | 2.58E-03 | GTExv8.EUR.Whole_Blood | 1.61E-02 | B4GALT7 | DBP |
| -2.9406 | 3.28E-03 | GTExv8.EUR.Whole_Blood | 1.95E-02 | RP11-1277A3.2 | DBP |
| 2.9490 | 3.19E-03 | GTExv8.EUR.Whole_Blood | 1.91E-02 | RUFY1 | DBP |
| 2.6559 | 7.91E-03 | GTExv8.EUR.Whole_Blood | 3.93E-02 | TXNDC5 | DBP |
| 3.8297 | 1.28E-04 | GTExv8.EUR.Whole_Blood | 1.37E-03 | TMEM170B | DBP |
| -3.5763 | 3.48E-04 | GTExv8.EUR.Whole_Blood | 3.14E-03 | ADTRP | DBP |
| 4.4980 | 6.86E-06 | GTExv8.EUR.Whole_Blood | 1.06E-04 | ATXN1 | DBP |
| 3.0711 | 2.13E-03 | GTExv8.EUR.Whole_Blood | 1.38E-02 | RNF144B | DBP |
| 3.4569 | 5.46E-04 | GTExv8.EUR.Whole_Blood | 4.60E-03 | CDKAL1 | DBP |
| -2.7264 | 6.40E-03 | GTExv8.EUR.Whole_Blood | 3.31E-02 | PPIAP29 | DBP |
| -3.0202 | 2.53E-03 | GTExv8.EUR.Whole_Blood | 1.58E-02 | CMAHP | DBP |
| -4.4873 | 7.21E-06 | GTExv8.EUR.Whole_Blood | 1.11E-04 | CARMIL1 | DBP |
| -3.0238 | 2.50E-03 | GTExv8.EUR.Whole_Blood | 1.57E-02 | ANKS1A | DBP |
| 2.6226 | 8.73E-03 | GTExv8.EUR.Whole_Blood | 4.24E-02 | RPL10A | DBP |
| 2.6425 | 8.23E-03 | GTExv8.EUR.Whole_Blood | 4.07E-02 | RP1-179N16.6 | DBP |
| -2.5680 | 1.02E-02 | GTExv8.EUR.Whole_Blood | 4.80E-02 | KCNK17 | DBP |
| 2.6034 | 9.23E-03 | GTExv8.EUR.Whole_Blood | 4.43E-02 | TREML4 | DBP |
| 2.6559 | 7.91E-03 | GTExv8.EUR.Whole_Blood | 3.93E-02 | RP1-229K20.8 | DBP |
| 4.6159 | 3.91E-06 | GTExv8.EUR.Whole_Blood | 6.36E-05 | GLTSCR1L | DBP |
| -3.5011 | 4.63E-04 | GTExv8.EUR.Whole_Blood | 3.99E-03 | RPL7L1 | DBP |
| 2.6373 | 8.36E-03 | GTExv8.EUR.Whole_Blood | 4.11E-02 | PPP2R5D | DBP |
| 2.7094 | 6.74E-03 | GTExv8.EUR.Whole_Blood | 3.45E-02 | RP1-8B1.4 | DBP |
| 2.9423 | 3.26E-03 | GTExv8.EUR.Whole_Blood | 1.94E-02 | SENP6 | DBP |
| 6.9449 | 3.79E-12 | GTExv8.EUR.Whole_Blood | 2.66E-10 | IRAK1BP1 | DBP |
| 9.7250 | 2.36E-22 | GTExv8.EUR.Whole_Blood | 4.92E-20 | PHIP | DBP |
| 2.7990 | 5.13E-03 | GTExv8.EUR.Whole_Blood | 2.78E-02 | DOPEY1 | DBP |
| -2.8633 | 4.19E-03 | GTExv8.EUR.Whole_Blood | 2.36E-02 | C6orf163 | DBP |
| 4.4189 | 9.92E-06 | GTExv8.EUR.Whole_Blood | 1.47E-04 | RRAGD | DBP |
| 5.4715 | 4.46E-08 | GTExv8.EUR.Whole_Blood | 1.16E-06 | UFL1 | DBP |
| -2.6088 | 9.09E-03 | GTExv8.EUR.Whole_Blood | 4.38E-02 | LINC00222 | DBP |
| -4.1320 | 3.60E-05 | GTExv8.EUR.Whole_Blood | 4.55E-04 | CCDC162P | DBP |
| -5.0766 | 3.84E-07 | GTExv8.EUR.Whole_Blood | 8.18E-06 | CD164 | DBP |
| 4.2985 | 1.72E-05 | GTExv8.EUR.Whole_Blood | 2.41E-04 | TRAF3IP2 | DBP |
| 5.5216 | 3.36E-08 | GTExv8.EUR.Whole_Blood | 9.09E-07 | NT5DC1 | DBP |
| 3.5844 | 3.38E-04 | GTExv8.EUR.Whole_Blood | 3.06E-03 | COL10A1 | DBP |
| -2.7927 | 5.23E-03 | GTExv8.EUR.Whole_Blood | 2.82E-02 | TSPYL4 | DBP |
| 3.5253 | 4.23E-04 | GTExv8.EUR.Whole_Blood | 3.70E-03 | DSE | DBP |
| -3.5866 | 3.35E-04 | GTExv8.EUR.Whole_Blood | 3.04E-03 | FAM26F | DBP |
| 4.8969 | 9.74E-07 | GTExv8.EUR.Whole_Blood | 1.90E-05 | DCBLD1 | DBP |
| -9.2565 | 2.11E-20 | GTExv8.EUR.Whole_Blood | 3.98E-18 | CEP85L | DBP |
| 3.3804 | 7.24E-04 | GTExv8.EUR.Whole_Blood | 5.74E-03 | MAN1A1 | DBP |
| -6.2965 | 3.04E-10 | GTExv8.EUR.Whole_Blood | 1.34E-08 | CENPW | DBP |
| 2.6931 | 7.08E-03 | GTExv8.EUR.Whole_Blood | 3.59E-02 | ARG1 | DBP |
| 2.7666 | 5.66E-03 | GTExv8.EUR.Whole_Blood | 3.01E-02 | MED23 | DBP |
| -4.4492 | 8.62E-06 | GTExv8.EUR.Whole_Blood | 1.29E-04 | TBPL1 | DBP |
| 4.2250 | 2.39E-05 | GTExv8.EUR.Whole_Blood | 3.22E-04 | HBS1L | DBP |
| 3.7712 | 1.62E-04 | GTExv8.EUR.Whole_Blood | 1.66E-03 | PHACTR2 | DBP |
| -3.0222 | 2.51E-03 | GTExv8.EUR.Whole_Blood | 1.57E-02 | STXBP5-AS1 | DBP |
| -6.1458 | 7.96E-10 | GTExv8.EUR.Whole_Blood | 3.20E-08 | STXBP5 | DBP |
| -2.5695 | 1.02E-02 | GTExv8.EUR.Whole_Blood | 4.80E-02 | UST | DBP |
| 2.8873 | 3.89E-03 | GTExv8.EUR.Whole_Blood | 2.23E-02 | TAB2 | DBP |
| 3.2058 | 1.35E-03 | GTExv8.EUR.Whole_Blood | 9.53E-03 | CCDC170 | DBP |
| -3.1476 | 1.65E-03 | GTExv8.EUR.Whole_Blood | 1.13E-02 | XXyac-YX65C7_A.2 | DBP |
| 5.4161 | 6.09E-08 | GTExv8.EUR.Whole_Blood | 1.54E-06 | FAM120B | DBP |
| 4.2474 | 2.16E-05 | GTExv8.EUR.Whole_Blood | 2.97E-04 | DLL1 | DBP |
| -2.8362 | 4.57E-03 | GTExv8.EUR.Whole_Blood | 2.53E-02 | TBP | DBP |
| 2.6560 | 7.91E-03 | GTExv8.EUR.Whole_Blood | 3.93E-02 | AC093627.7 | DBP |
| -3.6475 | 2.65E-04 | GTExv8.EUR.Whole_Blood | 2.53E-03 | ADAP1 | DBP |
| 4.4477 | 8.68E-06 | GTExv8.EUR.Whole_Blood | 1.30E-04 | GPR146 | DBP |
| 5.9005 | 3.63E-09 | GTExv8.EUR.Whole_Blood | 1.26E-07 | GPER1 | DBP |
| -6.1852 | 6.20E-10 | GTExv8.EUR.Whole_Blood | 2.57E-08 | AC091729.9 | DBP |
| -6.2032 | 5.53E-10 | GTExv8.EUR.Whole_Blood | 2.33E-08 | ZFAND2A | DBP |
| 3.7531 | 1.75E-04 | GTExv8.EUR.Whole_Blood | 1.76E-03 | MAD1L1 | DBP |
| -4.4743 | 7.67E-06 | GTExv8.EUR.Whole_Blood | 1.16E-04 | MRM2 | DBP |
| 4.6639 | 3.10E-06 | GTExv8.EUR.Whole_Blood | 5.19E-05 | GNA12 | DBP |
| 2.8821 | 3.95E-03 | GTExv8.EUR.Whole_Blood | 2.26E-02 | FOXK1 | DBP |
| 3.8095 | 1.39E-04 | GTExv8.EUR.Whole_Blood | 1.46E-03 | PMS2 | DBP |
| -2.5586 | 1.05E-02 | GTExv8.EUR.Whole_Blood | 4.91E-02 | FAM220A | DBP |
| -3.9816 | 6.84E-05 | GTExv8.EUR.Whole_Blood | 8.03E-04 | DAGLB | DBP |
| 3.9163 | 8.99E-05 | GTExv8.EUR.Whole_Blood | 1.01E-03 | GLCCI1 | DBP |
| 3.1287 | 1.76E-03 | GTExv8.EUR.Whole_Blood | 1.18E-02 | CDCA7L | DBP |
| -3.2757 | 1.05E-03 | GTExv8.EUR.Whole_Blood | 7.78E-03 | NFE2L3 | DBP |
| 2.7983 | 5.14E-03 | GTExv8.EUR.Whole_Blood | 2.78E-02 | HOXA9 | DBP |
| -3.3861 | 7.09E-04 | GTExv8.EUR.Whole_Blood | 5.65E-03 | HERPUD2 | DBP |
| -2.8495 | 4.38E-03 | GTExv8.EUR.Whole_Blood | 2.45E-02 | RP11-379H18.1 | DBP |
| 3.0508 | 2.28E-03 | GTExv8.EUR.Whole_Blood | 1.45E-02 | RP11-605P22.1 | DBP |
| 3.1678 | 1.54E-03 | GTExv8.EUR.Whole_Blood | 1.06E-02 | NME8 | DBP |
| 2.9946 | 2.75E-03 | GTExv8.EUR.Whole_Blood | 1.69E-02 | EPDR1 | DBP |
| 2.7548 | 5.87E-03 | GTExv8.EUR.Whole_Blood | 3.09E-02 | TRGC2 | DBP |
| 2.7445 | 6.06E-03 | GTExv8.EUR.Whole_Blood | 3.17E-02 | TRGJP2 | DBP |
| -3.0600 | 2.21E-03 | GTExv8.EUR.Whole_Blood | 1.42E-02 | TRGJ1 | DBP |
| 2.8487 | 4.39E-03 | GTExv8.EUR.Whole_Blood | 2.45E-02 | TRGV8 | DBP |
| 2.7930 | 5.22E-03 | GTExv8.EUR.Whole_Blood | 2.82E-02 | TRGV2 | DBP |
| 3.1527 | 1.62E-03 | GTExv8.EUR.Whole_Blood | 1.11E-02 | AC004951.5 | DBP |
| -3.1679 | 1.54E-03 | GTExv8.EUR.Whole_Blood | 1.06E-02 | LINC00957 | DBP |
| -3.8153 | 1.36E-04 | GTExv8.EUR.Whole_Blood | 1.44E-03 | RASA4CP | DBP |
| 2.5938 | 9.49E-03 | GTExv8.EUR.Whole_Blood | 4.53E-02 | ZMIZ2 | DBP |
| 2.9675 | 3.00E-03 | GTExv8.EUR.Whole_Blood | 1.81E-02 | RP4-647J21.1 | DBP |
| 2.7054 | 6.82E-03 | GTExv8.EUR.Whole_Blood | 3.49E-02 | CCM2 | DBP |
| 2.7166 | 6.60E-03 | GTExv8.EUR.Whole_Blood | 3.40E-02 | TBRG4 | DBP |
| 3.5058 | 4.55E-04 | GTExv8.EUR.Whole_Blood | 3.94E-03 | UPP1 | DBP |
| -2.6677 | 7.64E-03 | GTExv8.EUR.Whole_Blood | 3.81E-02 | SUMF2 | DBP |
| -3.0573 | 2.23E-03 | GTExv8.EUR.Whole_Blood | 1.43E-02 | ZNF736 | DBP |
| -2.6587 | 7.84E-03 | GTExv8.EUR.Whole_Blood | 3.90E-02 | TRIM60P18 | DBP |
| 2.7130 | 6.67E-03 | GTExv8.EUR.Whole_Blood | 3.43E-02 | ZNF107 | DBP |
| -2.8029 | 5.06E-03 | GTExv8.EUR.Whole_Blood | 2.75E-02 | EEF1DP4 | DBP |
| -3.2583 | 1.12E-03 | GTExv8.EUR.Whole_Blood | 8.16E-03 | MTDHP1 | DBP |
| 3.0562 | 2.24E-03 | GTExv8.EUR.Whole_Blood | 1.43E-02 | ERV3-1 | DBP |
| 3.0389 | 2.37E-03 | GTExv8.EUR.Whole_Blood | 1.50E-02 | CCT6P3 | DBP |
| 3.1272 | 1.76E-03 | GTExv8.EUR.Whole_Blood | 1.18E-02 | GTF2IP14 | DBP |
| -2.9995 | 2.70E-03 | GTExv8.EUR.Whole_Blood | 1.67E-02 | CCT6P1 | DBP |
| -3.3467 | 8.18E-04 | GTExv8.EUR.Whole_Blood | 6.35E-03 | AUTS2 | DBP |
| 4.3349 | 1.46E-05 | GTExv8.EUR.Whole_Blood | 2.08E-04 | GTF2I | DBP |
| 3.1595 | 1.58E-03 | GTExv8.EUR.Whole_Blood | 1.09E-02 | NCF1 | DBP |
| 3.7155 | 2.03E-04 | GTExv8.EUR.Whole_Blood | 2.00E-03 | GTF2IRD2 | DBP |
| 3.3320 | 8.62E-04 | GTExv8.EUR.Whole_Blood | 6.59E-03 | PMS2P5 | DBP |
| 4.8775 | 1.07E-06 | GTExv8.EUR.Whole_Blood | 2.05E-05 | RCC1L | DBP |
| 2.7985 | 5.13E-03 | GTExv8.EUR.Whole_Blood | 2.78E-02 | GTF2IP1 | DBP |
| 2.9838 | 2.85E-03 | GTExv8.EUR.Whole_Blood | 1.74E-02 | STAG3L1 | DBP |
| 3.3841 | 7.14E-04 | GTExv8.EUR.Whole_Blood | 5.67E-03 | NSUN5P1 | DBP |
| 4.4902 | 7.12E-06 | GTExv8.EUR.Whole_Blood | 1.10E-04 | POM121C | DBP |
| 3.9732 | 7.09E-05 | GTExv8.EUR.Whole_Blood | 8.26E-04 | SPDYE5 | DBP |
| -3.8189 | 1.34E-04 | GTExv8.EUR.Whole_Blood | 1.42E-03 | PMS2P3 | DBP |
| -3.0312 | 2.44E-03 | GTExv8.EUR.Whole_Blood | 1.54E-02 | SSC4D | DBP |
| 3.0069 | 2.64E-03 | GTExv8.EUR.Whole_Blood | 1.64E-02 | PTPN12 | DBP |
| 4.3666 | 1.26E-05 | GTExv8.EUR.Whole_Blood | 1.82E-04 | SEMA3C | DBP |
| -3.4031 | 6.66E-04 | GTExv8.EUR.Whole_Blood | 5.36E-03 | GTPBP10 | DBP |
| -3.0883 | 2.01E-03 | GTExv8.EUR.Whole_Blood | 1.32E-02 | PEG10 | DBP |
| -5.0080 | 5.50E-07 | GTExv8.EUR.Whole_Blood | 1.12E-05 | ARPC1B | DBP |
| 4.3990 | 1.09E-05 | GTExv8.EUR.Whole_Blood | 1.59E-04 | PDAP1 | DBP |
| -2.8080 | 4.99E-03 | GTExv8.EUR.Whole_Blood | 2.72E-02 | PTCD1 | DBP |
| 3.3148 | 9.17E-04 | GTExv8.EUR.Whole_Blood | 6.95E-03 | ZNF655 | DBP |
| -5.3744 | 7.68E-08 | GTExv8.EUR.Whole_Blood | 1.90E-06 | CYP3A5 | DBP |
| 2.8623 | 4.21E-03 | GTExv8.EUR.Whole_Blood | 2.37E-02 | ZNF3 | DBP |
| 7.2541 | 4.04E-13 | GTExv8.EUR.Whole_Blood | 3.44E-11 | CNPY4 | DBP |
| 2.7216 | 6.50E-03 | GTExv8.EUR.Whole_Blood | 3.35E-02 | MBLAC1 | DBP |
| -4.5827 | 4.59E-06 | GTExv8.EUR.Whole_Blood | 7.36E-05 | LAMTOR4 | DBP |
| 5.5695 | 2.55E-08 | GTExv8.EUR.Whole_Blood | 7.07E-07 | GIGYF1 | DBP |
| -3.9880 | 6.66E-05 | GTExv8.EUR.Whole_Blood | 7.83E-04 | EPHB4 | DBP |
| -6.7802 | 1.20E-11 | GTExv8.EUR.Whole_Blood | 7.61E-10 | UFSP1 | DBP |
| 2.8366 | 4.56E-03 | GTExv8.EUR.Whole_Blood | 2.53E-02 | PRKRIP1 | DBP |
| -3.1680 | 1.53E-03 | GTExv8.EUR.Whole_Blood | 1.06E-02 | ORAI2 | DBP |
| -4.0295 | 5.59E-05 | GTExv8.EUR.Whole_Blood | 6.69E-04 | RP11-514P8.2 | DBP |
| -2.8850 | 3.91E-03 | GTExv8.EUR.Whole_Blood | 2.24E-02 | ALKBH4 | DBP |
| 3.2323 | 1.23E-03 | GTExv8.EUR.Whole_Blood | 8.81E-03 | RASA4B | DBP |
| -3.0452 | 2.33E-03 | GTExv8.EUR.Whole_Blood | 1.48E-02 | POLR2J2 | DBP |
| 3.4325 | 5.98E-04 | GTExv8.EUR.Whole_Blood | 4.93E-03 | SRPK2 | DBP |
| -2.6393 | 8.31E-03 | GTExv8.EUR.Whole_Blood | 4.10E-02 | THAP5 | DBP |
| 2.6901 | 7.14E-03 | GTExv8.EUR.Whole_Blood | 3.61E-02 | IFRD1 | DBP |
| 2.5962 | 9.43E-03 | GTExv8.EUR.Whole_Blood | 4.51E-02 | MDFIC | DBP |
| -3.9308 | 8.47E-05 | GTExv8.EUR.Whole_Blood | 9.57E-04 | IRF5 | DBP |
| 3.0507 | 2.28E-03 | GTExv8.EUR.Whole_Blood | 1.45E-02 | RP11-309L24.10 | DBP |
| -3.0724 | 2.12E-03 | GTExv8.EUR.Whole_Blood | 1.37E-02 | NRF1 | DBP |
| -2.5826 | 9.81E-03 | GTExv8.EUR.Whole_Blood | 4.65E-02 | UBE2H | DBP |
| -3.5229 | 4.27E-04 | GTExv8.EUR.Whole_Blood | 3.73E-03 | AKR1B1 | DBP |
| 3.2825 | 1.03E-03 | GTExv8.EUR.Whole_Blood | 7.64E-03 | C7orf73 | DBP |
| 3.1286 | 1.76E-03 | GTExv8.EUR.Whole_Blood | 1.18E-02 | RP11-634H22.1 | DBP |
| 3.4263 | 6.12E-04 | GTExv8.EUR.Whole_Blood | 5.00E-03 | TBXAS1 | DBP |
| 3.2383 | 1.20E-03 | GTExv8.EUR.Whole_Blood | 8.62E-03 | ZNF425 | DBP |
| 2.5622 | 1.04E-02 | GTExv8.EUR.Whole_Blood | 4.87E-02 | ZNF398 | DBP |
| 3.8675 | 1.10E-04 | GTExv8.EUR.Whole_Blood | 1.20E-03 | ZNF767P | DBP |
| 2.9260 | 3.43E-03 | GTExv8.EUR.Whole_Blood | 2.02E-02 | KRBA1 | DBP |
| -2.9515 | 3.16E-03 | GTExv8.EUR.Whole_Blood | 1.90E-02 | LRRC61 | DBP |
| 3.3094 | 9.35E-04 | GTExv8.EUR.Whole_Blood | 7.04E-03 | ZBED6CL | DBP |
| -2.7974 | 5.15E-03 | GTExv8.EUR.Whole_Blood | 2.78E-02 | RP4-584D14.7 | DBP |
| 2.8243 | 4.74E-03 | GTExv8.EUR.Whole_Blood | 2.60E-02 | ZNF775 | DBP |
| 4.6918 | 2.71E-06 | GTExv8.EUR.Whole_Blood | 4.61E-05 | GIMAP7 | DBP |
| -5.2911 | 1.22E-07 | GTExv8.EUR.Whole_Blood | 2.90E-06 | GIMAP1 | DBP |
| -2.6234 | 8.71E-03 | GTExv8.EUR.Whole_Blood | 4.24E-02 | ABCF2 | DBP |
| -2.5559 | 1.06E-02 | GTExv8.EUR.Whole_Blood | 4.95E-02 | CHPF2 | DBP |
| -3.0822 | 2.05E-03 | GTExv8.EUR.Whole_Blood | 1.34E-02 | RPL23AP53 | DBP |
| 4.1604 | 3.18E-05 | GTExv8.EUR.Whole_Blood | 4.10E-04 | ERICH1 | DBP |
| 5.8169 | 5.99E-09 | GTExv8.EUR.Whole_Blood | 1.93E-07 | CLN8 | DBP |
| -4.1837 | 2.87E-05 | GTExv8.EUR.Whole_Blood | 3.77E-04 | CTD-2336O2.3 | DBP |
| 5.0733 | 3.91E-07 | GTExv8.EUR.Whole_Blood | 8.31E-06 | CTD-2336O2.1 | DBP |
| 6.4476 | 1.14E-10 | GTExv8.EUR.Whole_Blood | 5.68E-09 | ALG1L13P | DBP |
| 5.3056 | 1.12E-07 | GTExv8.EUR.Whole_Blood | 2.70E-06 | MFHAS1 | DBP |
| 6.5930 | 4.31E-11 | GTExv8.EUR.Whole_Blood | 2.41E-09 | RPL10P19 | DBP |
| -3.3821 | 7.19E-04 | GTExv8.EUR.Whole_Blood | 5.71E-03 | ERI1 | DBP |
| 3.2474 | 1.16E-03 | GTExv8.EUR.Whole_Blood | 8.39E-03 | RP11-10A14.5 | DBP |
| 6.7402 | 1.58E-11 | GTExv8.EUR.Whole_Blood | 9.71E-10 | LINCR-0001 | DBP |
| 5.5221 | 3.35E-08 | GTExv8.EUR.Whole_Blood | 9.09E-07 | RP11-981G7.6 | DBP |
| 7.0177 | 2.26E-12 | GTExv8.EUR.Whole_Blood | 1.69E-10 | AF131215.9 | DBP |
| 6.9807 | 2.94E-12 | GTExv8.EUR.Whole_Blood | 2.12E-10 | AF131215.2 | DBP |
| -5.0171 | 5.25E-07 | GTExv8.EUR.Whole_Blood | 1.08E-05 | SLC35G5 | DBP |
| 6.6921 | 2.20E-11 | GTExv8.EUR.Whole_Blood | 1.30E-09 | FAM167A | DBP |
| -6.6286 | 3.39E-11 | GTExv8.EUR.Whole_Blood | 1.93E-09 | BLK | DBP |
| -6.7260 | 1.74E-11 | GTExv8.EUR.Whole_Blood | 1.06E-09 | RP11-148O21.4 | DBP |
| -5.4120 | 6.23E-08 | GTExv8.EUR.Whole_Blood | 1.57E-06 | RP11-148O21.2 | DBP |
| 7.2987 | 2.91E-13 | GTExv8.EUR.Whole_Blood | 2.65E-11 | NEIL2 | DBP |
| 5.8458 | 5.04E-09 | GTExv8.EUR.Whole_Blood | 1.69E-07 | FDFT1 | DBP |
| -4.1338 | 3.57E-05 | GTExv8.EUR.Whole_Blood | 4.54E-04 | RP11-297N6.4 | DBP |
| -2.5864 | 9.70E-03 | GTExv8.EUR.Whole_Blood | 4.61E-02 | CTSB | DBP |
| -3.3944 | 6.88E-04 | GTExv8.EUR.Whole_Blood | 5.51E-03 | DLC1 | DBP |
| -2.8737 | 4.06E-03 | GTExv8.EUR.Whole_Blood | 2.30E-02 | NUDT18 | DBP |
| 2.8591 | 4.25E-03 | GTExv8.EUR.Whole_Blood | 2.38E-02 | REEP4 | DBP |
| 2.8678 | 4.13E-03 | GTExv8.EUR.Whole_Blood | 2.34E-02 | C8orf58 | DBP |
| 3.1782 | 1.48E-03 | GTExv8.EUR.Whole_Blood | 1.03E-02 | CCAR2 | DBP |
| 2.7323 | 6.29E-03 | GTExv8.EUR.Whole_Blood | 3.27E-02 | BIN3 | DBP |
| -2.8339 | 4.60E-03 | GTExv8.EUR.Whole_Blood | 2.54E-02 | CHMP7 | DBP |
| -4.1840 | 2.86E-05 | GTExv8.EUR.Whole_Blood | 3.76E-04 | NKX3-1 | DBP |
| -3.6555 | 2.57E-04 | GTExv8.EUR.Whole_Blood | 2.46E-03 | SDAD1P1 | DBP |
| -2.5780 | 9.94E-03 | GTExv8.EUR.Whole_Blood | 4.70E-02 | CCDC25 | DBP |
| 6.5720 | 4.96E-11 | GTExv8.EUR.Whole_Blood | 2.69E-09 | BAG4 | DBP |
| -6.5942 | 4.28E-11 | GTExv8.EUR.Whole_Blood | 2.41E-09 | RP11-350N15.5 | DBP |
| -3.6476 | 2.65E-04 | GTExv8.EUR.Whole_Blood | 2.53E-03 | RP11-350N15.4 | DBP |
| 2.6059 | 9.16E-03 | GTExv8.EUR.Whole_Blood | 4.41E-02 | GOLGA7 | DBP |
| 2.9159 | 3.55E-03 | GTExv8.EUR.Whole_Blood | 2.08E-02 | KAT6A | DBP |
| -2.6822 | 7.31E-03 | GTExv8.EUR.Whole_Blood | 3.68E-02 | SMIM19 | DBP |
| -2.6916 | 7.11E-03 | GTExv8.EUR.Whole_Blood | 3.60E-02 | MRPL15 | DBP |
| -3.3445 | 8.24E-04 | GTExv8.EUR.Whole_Blood | 6.39E-03 | ADHFE1 | DBP |
| 3.7363 | 1.87E-04 | GTExv8.EUR.Whole_Blood | 1.86E-03 | C8orf46 | DBP |
| -2.5966 | 9.41E-03 | GTExv8.EUR.Whole_Blood | 4.51E-02 | MYBL1 | DBP |
| -2.5995 | 9.33E-03 | GTExv8.EUR.Whole_Blood | 4.48E-02 | TPD52 | DBP |
| -3.1350 | 1.72E-03 | GTExv8.EUR.Whole_Blood | 1.16E-02 | PAG1 | DBP |
| 2.6773 | 7.42E-03 | GTExv8.EUR.Whole_Blood | 3.71E-02 | IMPA1 | DBP |
| 4.3623 | 1.29E-05 | GTExv8.EUR.Whole_Blood | 1.86E-04 | SNX16 | DBP |
| -4.4874 | 7.21E-06 | GTExv8.EUR.Whole_Blood | 1.11E-04 | OSGIN2 | DBP |
| 4.6286 | 3.68E-06 | GTExv8.EUR.Whole_Blood | 6.04E-05 | NBN | DBP |
| -2.6982 | 6.97E-03 | GTExv8.EUR.Whole_Blood | 3.54E-02 | INTS8 | DBP |
| 5.8139 | 6.10E-09 | GTExv8.EUR.Whole_Blood | 1.96E-07 | TP53INP1 | DBP |
| 6.1036 | 1.04E-09 | GTExv8.EUR.Whole_Blood | 3.98E-08 | RP11-347C18.3 | DBP |
| 3.7019 | 2.14E-04 | GTExv8.EUR.Whole_Blood | 2.10E-03 | PLEKHF2 | DBP |
| -3.7374 | 1.86E-04 | GTExv8.EUR.Whole_Blood | 1.86E-03 | TSPYL5 | DBP |
| 5.5180 | 3.43E-08 | GTExv8.EUR.Whole_Blood | 9.21E-07 | AZIN1-AS1 | DBP |
| -2.6369 | 8.37E-03 | GTExv8.EUR.Whole_Blood | 4.11E-02 | RAD21 | DBP |
| 3.8923 | 9.93E-05 | GTExv8.EUR.Whole_Blood | 1.10E-03 | EXT1 | DBP |
| -2.7749 | 5.52E-03 | GTExv8.EUR.Whole_Blood | 2.95E-02 | TAF2 | DBP |
| 3.7530 | 1.75E-04 | GTExv8.EUR.Whole_Blood | 1.76E-03 | SLA | DBP |
| -3.1219 | 1.80E-03 | GTExv8.EUR.Whole_Blood | 1.20E-02 | ZFAT | DBP |
| 3.8305 | 1.28E-04 | GTExv8.EUR.Whole_Blood | 1.37E-03 | CTD-3064M3.7 | DBP |
| -4.9761 | 6.49E-07 | GTExv8.EUR.Whole_Blood | 1.30E-05 | TSNARE1 | DBP |
| 2.8679 | 4.13E-03 | GTExv8.EUR.Whole_Blood | 2.34E-02 | ADGRB1 | DBP |
| -2.6799 | 7.37E-03 | GTExv8.EUR.Whole_Blood | 3.70E-02 | THEM6 | DBP |
| -2.8677 | 4.13E-03 | GTExv8.EUR.Whole_Blood | 2.34E-02 | C8orf31 | DBP |
| -2.7482 | 5.99E-03 | GTExv8.EUR.Whole_Blood | 3.14E-02 | COMMD5 | DBP |
| -3.8052 | 1.42E-04 | GTExv8.EUR.Whole_Blood | 1.49E-03 | WASHC1 | DBP |
| -3.6517 | 2.61E-04 | GTExv8.EUR.Whole_Blood | 2.49E-03 | FOXD4 | DBP |
| -3.8919 | 9.95E-05 | GTExv8.EUR.Whole_Blood | 1.10E-03 | CBWD1 | DBP |
| -3.1972 | 1.39E-03 | GTExv8.EUR.Whole_Blood | 9.76E-03 | VLDLR | DBP |
| -6.3838 | 1.73E-10 | GTExv8.EUR.Whole_Blood | 8.06E-09 | MTAP | DBP |
| -3.0914 | 1.99E-03 | GTExv8.EUR.Whole_Blood | 1.31E-02 | CDKN2B | DBP |
| 2.8657 | 4.16E-03 | GTExv8.EUR.Whole_Blood | 2.35E-02 | C9orf72 | DBP |
| -4.3528 | 1.34E-05 | GTExv8.EUR.Whole_Blood | 1.92E-04 | NUDT2 | DBP |
| -4.1095 | 3.96E-05 | GTExv8.EUR.Whole_Blood | 4.95E-04 | RPP25L | DBP |
| 3.5588 | 3.72E-04 | GTExv8.EUR.Whole_Blood | 3.33E-03 | RP11-195F19.9 | DBP |
| -3.8238 | 1.31E-04 | GTExv8.EUR.Whole_Blood | 1.40E-03 | ARHGEF39 | DBP |
| 6.3457 | 2.21E-10 | GTExv8.EUR.Whole_Blood | 1.01E-08 | TPM2 | DBP |
| 2.6647 | 7.71E-03 | GTExv8.EUR.Whole_Blood | 3.84E-02 | ALDH1A1 | DBP |
| -3.0338 | 2.42E-03 | GTExv8.EUR.Whole_Blood | 1.53E-02 | RP11-522I20.3 | DBP |
| 2.9815 | 2.87E-03 | GTExv8.EUR.Whole_Blood | 1.75E-02 | SPIN1 | DBP |
| -3.0042 | 2.66E-03 | GTExv8.EUR.Whole_Blood | 1.65E-02 | NXNL2 | DBP |
| 3.1009 | 1.93E-03 | GTExv8.EUR.Whole_Blood | 1.27E-02 | ANKRD19P | DBP |
| -2.8468 | 4.42E-03 | GTExv8.EUR.Whole_Blood | 2.46E-02 | RP11-498P14.5 | DBP |
| 2.5614 | 1.04E-02 | GTExv8.EUR.Whole_Blood | 4.87E-02 | ANKS6 | DBP |
| -2.9172 | 3.53E-03 | GTExv8.EUR.Whole_Blood | 2.07E-02 | TGFBR1 | DBP |
| 2.5858 | 9.71E-03 | GTExv8.EUR.Whole_Blood | 4.61E-02 | EPB41L4B | DBP |
| -2.7994 | 5.12E-03 | GTExv8.EUR.Whole_Blood | 2.78E-02 | PTGR1 | DBP |
| 3.6717 | 2.41E-04 | GTExv8.EUR.Whole_Blood | 2.33E-03 | PSMD5-AS1 | DBP |
| -3.5217 | 4.29E-04 | GTExv8.EUR.Whole_Blood | 3.74E-03 | RBM18 | DBP |
| -3.3566 | 7.89E-04 | GTExv8.EUR.Whole_Blood | 6.17E-03 | CRB2 | DBP |
| -2.9573 | 3.10E-03 | GTExv8.EUR.Whole_Blood | 1.87E-02 | PPP6C | DBP |
| -2.7373 | 6.19E-03 | GTExv8.EUR.Whole_Blood | 3.23E-02 | GAPVD1 | DBP |
| 4.9470 | 7.54E-07 | GTExv8.EUR.Whole_Blood | 1.50E-05 | ZBTB34 | DBP |
| -4.3729 | 1.23E-05 | GTExv8.EUR.Whole_Blood | 1.78E-04 | TTC16 | DBP |
| -3.8257 | 1.30E-04 | GTExv8.EUR.Whole_Blood | 1.39E-03 | FPGS | DBP |
| -3.0338 | 2.41E-03 | GTExv8.EUR.Whole_Blood | 1.52E-02 | RP11-228B15.4 | DBP |
| -2.9731 | 2.95E-03 | GTExv8.EUR.Whole_Blood | 1.79E-02 | ENG | DBP |
| 3.4504 | 5.60E-04 | GTExv8.EUR.Whole_Blood | 4.67E-03 | GLE1 | DBP |
| 3.0296 | 2.45E-03 | GTExv8.EUR.Whole_Blood | 1.54E-02 | PTPA | DBP |
| 3.3597 | 7.80E-04 | GTExv8.EUR.Whole_Blood | 6.12E-03 | CRAT | DBP |
| -3.2399 | 1.20E-03 | GTExv8.EUR.Whole_Blood | 8.62E-03 | PRRC2B | DBP |
| 4.1071 | 4.01E-05 | GTExv8.EUR.Whole_Blood | 5.00E-04 | UCK1 | DBP |
| 7.2717 | 3.55E-13 | GTExv8.EUR.Whole_Blood | 3.13E-11 | ABO | DBP |
| 3.5471 | 3.90E-04 | GTExv8.EUR.Whole_Blood | 3.46E-03 | SURF1 | DBP |
| -2.7582 | 5.81E-03 | GTExv8.EUR.Whole_Blood | 3.07E-02 | SEC16A | DBP |
| 3.1426 | 1.67E-03 | GTExv8.EUR.Whole_Blood | 1.14E-02 | NALT1 | DBP |
| -2.9176 | 3.53E-03 | GTExv8.EUR.Whole_Blood | 2.07E-02 | CCDC183 | DBP |
| 3.5737 | 3.52E-04 | GTExv8.EUR.Whole_Blood | 3.17E-03 | TRAF2 | DBP |
| 3.8815 | 1.04E-04 | GTExv8.EUR.Whole_Blood | 1.14E-03 | FBXW5 | DBP |
| 3.6744 | 2.38E-04 | GTExv8.EUR.Whole_Blood | 2.31E-03 | C9orf142 | DBP |
| -3.4199 | 6.26E-04 | GTExv8.EUR.Whole_Blood | 5.10E-03 | ABCA2 | DBP |
| -3.2542 | 1.14E-03 | GTExv8.EUR.Whole_Blood | 8.29E-03 | DPH7 | DBP |
| 2.9519 | 3.16E-03 | GTExv8.EUR.Whole_Blood | 1.90E-02 | ARRDC1 | DBP |
| 2.8595 | 4.24E-03 | GTExv8.EUR.Whole_Blood | 2.38E-02 | EHMT1 | DBP |
| 3.2224 | 1.27E-03 | GTExv8.EUR.Whole_Blood | 9.06E-03 | RP11-188C12.2 | DBP |
| 3.1278 | 1.76E-03 | GTExv8.EUR.Whole_Blood | 1.18E-02 | WDR37 | DBP |
| 3.2832 | 1.03E-03 | GTExv8.EUR.Whole_Blood | 7.64E-03 | GDI2 | DBP |
| 2.8760 | 4.03E-03 | GTExv8.EUR.Whole_Blood | 2.29E-02 | RP11-186N15.3 | DBP |
| 4.8691 | 1.12E-06 | GTExv8.EUR.Whole_Blood | 2.12E-05 | BEND7 | DBP |
| 2.9007 | 3.72E-03 | GTExv8.EUR.Whole_Blood | 2.16E-02 | RP11-295P9.3 | DBP |
| -2.8968 | 3.77E-03 | GTExv8.EUR.Whole_Blood | 2.18E-02 | PTER | DBP |
| 3.7622 | 1.68E-04 | GTExv8.EUR.Whole_Blood | 1.70E-03 | CUBN | DBP |
| 3.3022 | 9.59E-04 | GTExv8.EUR.Whole_Blood | 7.20E-03 | TRDMT1 | DBP |
| 8.3539 | 6.61E-17 | GTExv8.EUR.Whole_Blood | 9.35E-15 | CACNB2 | DBP |
| 3.1363 | 1.71E-03 | GTExv8.EUR.Whole_Blood | 1.16E-02 | RP11-499P20.2 | DBP |
| 6.9851 | 2.85E-12 | GTExv8.EUR.Whole_Blood | 2.08E-10 | NSUN6 | DBP |
| -4.6479 | 3.35E-06 | GTExv8.EUR.Whole_Blood | 5.59E-05 | RP11-354E11.2 | DBP |
| -3.2149 | 1.30E-03 | GTExv8.EUR.Whole_Blood | 9.25E-03 | LINC00202-2 | DBP |
| 3.7496 | 1.77E-04 | GTExv8.EUR.Whole_Blood | 1.78E-03 | RP13-16H11.8 | DBP |
| 3.6119 | 3.04E-04 | GTExv8.EUR.Whole_Blood | 2.81E-03 | PDSS1 | DBP |
| 4.5274 | 5.97E-06 | GTExv8.EUR.Whole_Blood | 9.37E-05 | ABI1 | DBP |
| 3.3973 | 6.80E-04 | GTExv8.EUR.Whole_Blood | 5.46E-03 | WAC | DBP |
| 3.4148 | 6.38E-04 | GTExv8.EUR.Whole_Blood | 5.19E-03 | SVIL-AS1 | DBP |
| -3.9447 | 7.99E-05 | GTExv8.EUR.Whole_Blood | 9.08E-04 | RP11-330O11.3 | DBP |
| -2.8343 | 4.59E-03 | GTExv8.EUR.Whole_Blood | 2.54E-02 | CCDC7 | DBP |
| 3.1916 | 1.42E-03 | GTExv8.EUR.Whole_Blood | 9.94E-03 | CUL2 | DBP |
| -3.0937 | 1.98E-03 | GTExv8.EUR.Whole_Blood | 1.30E-02 | OR13A1 | DBP |
| 3.8667 | 1.10E-04 | GTExv8.EUR.Whole_Blood | 1.20E-03 | ALOX5 | DBP |
| -2.9387 | 3.30E-03 | GTExv8.EUR.Whole_Blood | 1.96E-02 | MARCH8 | DBP |
| 3.9932 | 6.52E-05 | GTExv8.EUR.Whole_Blood | 7.68E-04 | AGAP10P | DBP |
| -3.4928 | 4.78E-04 | GTExv8.EUR.Whole_Blood | 4.09E-03 | WASHC2C | DBP |
| -3.6232 | 2.91E-04 | GTExv8.EUR.Whole_Blood | 2.71E-03 | AGAP4 | DBP |
| 3.3452 | 8.22E-04 | GTExv8.EUR.Whole_Blood | 6.38E-03 | AGAP14 | DBP |
| 2.8598 | 4.24E-03 | GTExv8.EUR.Whole_Blood | 2.38E-02 | BMS1P1 | DBP |
| -3.4681 | 5.24E-04 | GTExv8.EUR.Whole_Blood | 4.43E-03 | AGAP9 | DBP |
| -3.6468 | 2.66E-04 | GTExv8.EUR.Whole_Blood | 2.53E-03 | BMS1P2 | DBP |
| 3.6170 | 2.98E-04 | GTExv8.EUR.Whole_Blood | 2.76E-03 | AGAP12P | DBP |
| 2.6257 | 8.65E-03 | GTExv8.EUR.Whole_Blood | 4.22E-02 | MAPK8 | DBP |
| -3.1581 | 1.59E-03 | GTExv8.EUR.Whole_Blood | 1.09E-02 | ARHGAP22 | DBP |
| 3.5295 | 4.16E-04 | GTExv8.EUR.Whole_Blood | 3.65E-03 | SGMS1-AS1 | DBP |
| 3.5445 | 3.93E-04 | GTExv8.EUR.Whole_Blood | 3.47E-03 | ANK3 | DBP |
| -3.0464 | 2.32E-03 | GTExv8.EUR.Whole_Blood | 1.48E-02 | RTKN2 | DBP |
| -2.9888 | 2.80E-03 | GTExv8.EUR.Whole_Blood | 1.72E-02 | ZNF365 | DBP |
| 7.6177 | 2.58E-14 | GTExv8.EUR.Whole_Blood | 2.62E-12 | ADO | DBP |
| -7.5872 | 3.27E-14 | GTExv8.EUR.Whole_Blood | 3.24E-12 | REEP3 | DBP |
| 4.5668 | 4.95E-06 | GTExv8.EUR.Whole_Blood | 7.88E-05 | SIRT1 | DBP |
| 3.9653 | 7.33E-05 | GTExv8.EUR.Whole_Blood | 8.45E-04 | STOX1 | DBP |
| -2.7362 | 6.21E-03 | GTExv8.EUR.Whole_Blood | 3.24E-02 | C10orf105 | DBP |
| -4.8632 | 1.16E-06 | GTExv8.EUR.Whole_Blood | 2.18E-05 | NUDT13 | DBP |
| 4.2167 | 2.48E-05 | GTExv8.EUR.Whole_Blood | 3.33E-04 | FAM149B1 | DBP |
| 5.5191 | 3.41E-08 | GTExv8.EUR.Whole_Blood | 9.19E-07 | MRPS16 | DBP |
| -4.1784 | 2.94E-05 | GTExv8.EUR.Whole_Blood | 3.84E-04 | DNAJC9-AS1 | DBP |
| -4.7968 | 1.61E-06 | GTExv8.EUR.Whole_Blood | 2.90E-05 | CFAP70 | DBP |
| -6.9124 | 4.77E-12 | GTExv8.EUR.Whole_Blood | 3.23E-10 | BMS1P4 | DBP |
| -8.2382 | 1.75E-16 | GTExv8.EUR.Whole_Blood | 2.34E-14 | FUT11 | DBP |
| -3.1287 | 1.76E-03 | GTExv8.EUR.Whole_Blood | 1.18E-02 | VCL | DBP |
| -2.7913 | 5.25E-03 | GTExv8.EUR.Whole_Blood | 2.83E-02 | FAM213A | DBP |
| -3.2026 | 1.36E-03 | GTExv8.EUR.Whole_Blood | 9.59E-03 | FAM35A | DBP |
| 2.7037 | 6.86E-03 | GTExv8.EUR.Whole_Blood | 3.50E-02 | PAPSS2 | DBP |
| -3.3364 | 8.49E-04 | GTExv8.EUR.Whole_Blood | 6.52E-03 | IFIT5 | DBP |
| -2.7728 | 5.56E-03 | GTExv8.EUR.Whole_Blood | 2.97E-02 | TNKS2-AS1 | DBP |
| 3.6441 | 2.68E-04 | GTExv8.EUR.Whole_Blood | 2.54E-03 | EIF2S2P3 | DBP |
| -11.2723 | 1.80E-29 | GTExv8.EUR.Whole_Blood | 6.48E-27 | NOC3L | DBP |
| -5.4435 | 5.23E-08 | GTExv8.EUR.Whole_Blood | 1.34E-06 | TBC1D12 | DBP |
| -8.8929 | 5.95E-19 | GTExv8.EUR.Whole_Blood | 1.02E-16 | HELLS | DBP |
| 3.4562 | 5.48E-04 | GTExv8.EUR.Whole_Blood | 4.61E-03 | R3HCC1L | DBP |
| 2.8818 | 3.95E-03 | GTExv8.EUR.Whole_Blood | 2.26E-02 | HPS1 | DBP |
| -3.6181 | 2.97E-04 | GTExv8.EUR.Whole_Blood | 2.76E-03 | ABCC2 | DBP |
| 3.1052 | 1.90E-03 | GTExv8.EUR.Whole_Blood | 1.26E-02 | PHBP9 | DBP |
| 2.6969 | 7.00E-03 | GTExv8.EUR.Whole_Blood | 3.56E-02 | SEC31B | DBP |
| -2.5519 | 1.07E-02 | GTExv8.EUR.Whole_Blood | 4.98E-02 | LDB1 | DBP |
| -7.0748 | 1.50E-12 | GTExv8.EUR.Whole_Blood | 1.18E-10 | MFSD13A | DBP |
| 4.0026 | 6.26E-05 | GTExv8.EUR.Whole_Blood | 7.41E-04 | ACTR1A | DBP |
| -4.9791 | 6.39E-07 | GTExv8.EUR.Whole_Blood | 1.29E-05 | SUFU | DBP |
| 6.0912 | 1.12E-09 | GTExv8.EUR.Whole_Blood | 4.23E-08 | TRIM8 | DBP |
| 5.0942 | 3.50E-07 | GTExv8.EUR.Whole_Blood | 7.56E-06 | SFXN2 | DBP |
| -7.2687 | 3.63E-13 | GTExv8.EUR.Whole_Blood | 3.14E-11 | BORCS7 | DBP |
| -4.6950 | 2.67E-06 | GTExv8.EUR.Whole_Blood | 4.55E-05 | CNNM2 | DBP |
| -15.9211 | 4.52E-57 | GTExv8.EUR.Whole_Blood | 7.16E-54 | MARCKSL1P1 | DBP |
| -13.1954 | 9.32E-40 | GTExv8.EUR.Whole_Blood | 5.68E-37 | NT5C2 | DBP |
| -4.6288 | 3.68E-06 | GTExv8.EUR.Whole_Blood | 6.04E-05 | STN1 | DBP |
| -2.7118 | 6.69E-03 | GTExv8.EUR.Whole_Blood | 3.44E-02 | GSTO2 | DBP |
| 3.2859 | 1.02E-03 | GTExv8.EUR.Whole_Blood | 7.59E-03 | ADD3 | DBP |
| -2.8965 | 3.77E-03 | GTExv8.EUR.Whole_Blood | 2.18E-02 | BBIP1 | DBP |
| -2.7004 | 6.93E-03 | GTExv8.EUR.Whole_Blood | 3.53E-02 | CASP7 | DBP |
| -5.0349 | 4.78E-07 | GTExv8.EUR.Whole_Blood | 9.94E-06 | FAM160B1 | DBP |
| -3.4456 | 5.70E-04 | GTExv8.EUR.Whole_Blood | 4.73E-03 | RGS10 | DBP |
| -3.2605 | 1.11E-03 | GTExv8.EUR.Whole_Blood | 8.10E-03 | ACADSB | DBP |
| 4.1771 | 2.95E-05 | GTExv8.EUR.Whole_Blood | 3.84E-04 | METTL10 | DBP |
| -5.2720 | 1.35E-07 | GTExv8.EUR.Whole_Blood | 3.17E-06 | PPP2R2D | DBP |
| 3.8188 | 1.34E-04 | GTExv8.EUR.Whole_Blood | 1.42E-03 | MOB2 | DBP |
| 3.9518 | 7.76E-05 | GTExv8.EUR.Whole_Blood | 8.89E-04 | KRTAP5-AS1 | DBP |
| -9.9777 | 1.91E-23 | GTExv8.EUR.Whole_Blood | 4.09E-21 | TNNT3 | DBP |
| 4.2412 | 2.22E-05 | GTExv8.EUR.Whole_Blood | 3.02E-04 | MRPL23 | DBP |
| 9.7059 | 2.85E-22 | GTExv8.EUR.Whole_Blood | 5.79E-20 | MRPL23-AS1 | DBP |
| -2.6035 | 9.23E-03 | GTExv8.EUR.Whole_Blood | 4.43E-02 | ASCL2 | DBP |
| -2.6894 | 7.16E-03 | GTExv8.EUR.Whole_Blood | 3.62E-02 | TRIM68 | DBP |
| -3.5548 | 3.78E-04 | GTExv8.EUR.Whole_Blood | 3.37E-03 | TRIM66 | DBP |
| -3.0352 | 2.40E-03 | GTExv8.EUR.Whole_Blood | 1.52E-02 | RPL27A | DBP |
| -4.5158 | 6.31E-06 | GTExv8.EUR.Whole_Blood | 9.84E-05 | ST5 | DBP |
| 4.5487 | 5.40E-06 | GTExv8.EUR.Whole_Blood | 8.51E-05 | SBF2-AS1 | DBP |
| -5.6917 | 1.26E-08 | GTExv8.EUR.Whole_Blood | 3.74E-07 | EIF4G2 | DBP |
| -5.6355 | 1.75E-08 | GTExv8.EUR.Whole_Blood | 5.10E-07 | ARNTL | DBP |
| 4.6361 | 3.55E-06 | GTExv8.EUR.Whole_Blood | 5.86E-05 | BTBD10 | DBP |
| 4.7983 | 1.60E-06 | GTExv8.EUR.Whole_Blood | 2.89E-05 | COPB1 | DBP |
| -5.2290 | 1.70E-07 | GTExv8.EUR.Whole_Blood | 3.94E-06 | NUCB2 | DBP |
| 2.6934 | 7.07E-03 | GTExv8.EUR.Whole_Blood | 3.58E-02 | SERGEF | DBP |
| -2.7007 | 6.92E-03 | GTExv8.EUR.Whole_Blood | 3.53E-02 | ANO5 | DBP |
| 3.0784 | 2.08E-03 | GTExv8.EUR.Whole_Blood | 1.35E-02 | PRRG4 | DBP |
| 4.2091 | 2.56E-05 | GTExv8.EUR.Whole_Blood | 3.43E-04 | ARHGAP1 | DBP |
| 5.8778 | 4.16E-09 | GTExv8.EUR.Whole_Blood | 1.43E-07 | NR1H3 | DBP |
| 5.8004 | 6.62E-09 | GTExv8.EUR.Whole_Blood | 2.09E-07 | ACP2 | DBP |
| 3.1534 | 1.61E-03 | GTExv8.EUR.Whole_Blood | 1.10E-02 | MYBPC3 | DBP |
| 6.8034 | 1.02E-11 | GTExv8.EUR.Whole_Blood | 6.57E-10 | SLC39A13 | DBP |
| -7.6101 | 2.74E-14 | GTExv8.EUR.Whole_Blood | 2.75E-12 | CELF1 | DBP |
| 7.4928 | 6.74E-14 | GTExv8.EUR.Whole_Blood | 6.43E-12 | C1QTNF4 | DBP |
| -11.1155 | 1.05E-28 | GTExv8.EUR.Whole_Blood | 3.62E-26 | FNBP4 | DBP |
| 4.5597 | 5.12E-06 | GTExv8.EUR.Whole_Blood | 8.13E-05 | NUP160 | DBP |
| -4.6061 | 4.10E-06 | GTExv8.EUR.Whole_Blood | 6.64E-05 | PTPRJ | DBP |
| 3.1407 | 1.69E-03 | GTExv8.EUR.Whole_Blood | 1.15E-02 | SMTNL1 | DBP |
| 7.2680 | 3.65E-13 | GTExv8.EUR.Whole_Blood | 3.14E-11 | MED19 | DBP |
| -3.2691 | 1.08E-03 | GTExv8.EUR.Whole_Blood | 7.95E-03 | TMX2 | DBP |
| -5.5784 | 2.43E-08 | GTExv8.EUR.Whole_Blood | 6.80E-07 | ZFP91 | DBP |
| 2.8345 | 4.59E-03 | GTExv8.EUR.Whole_Blood | 2.54E-02 | MYRF | DBP |
| 2.7520 | 5.92E-03 | GTExv8.EUR.Whole_Blood | 3.11E-02 | ASRGL1 | DBP |
| -4.7752 | 1.79E-06 | GTExv8.EUR.Whole_Blood | 3.19E-05 | AHNAK | DBP |
| -2.9491 | 3.19E-03 | GTExv8.EUR.Whole_Blood | 1.91E-02 | EEF1G | DBP |
| -3.5336 | 4.10E-04 | GTExv8.EUR.Whole_Blood | 3.61E-03 | INTS5 | DBP |
| -3.5166 | 4.37E-04 | GTExv8.EUR.Whole_Blood | 3.81E-03 | LBHD1 | DBP |
| -3.1026 | 1.92E-03 | GTExv8.EUR.Whole_Blood | 1.27E-02 | TMEM223 | DBP |
| -2.9913 | 2.78E-03 | GTExv8.EUR.Whole_Blood | 1.71E-02 | PLA2G16 | DBP |
| 2.5781 | 9.94E-03 | GTExv8.EUR.Whole_Blood | 4.70E-02 | ATL3 | DBP |
| -2.7636 | 5.72E-03 | GTExv8.EUR.Whole_Blood | 3.03E-02 | NAA40 | DBP |
| 3.2859 | 1.02E-03 | GTExv8.EUR.Whole_Blood | 7.59E-03 | COX8A | DBP |
| 3.2054 | 1.35E-03 | GTExv8.EUR.Whole_Blood | 9.53E-03 | GPR137 | DBP |
| -4.4843 | 7.32E-06 | GTExv8.EUR.Whole_Blood | 1.12E-04 | SNX15 | DBP |
| -3.0678 | 2.16E-03 | GTExv8.EUR.Whole_Blood | 1.39E-02 | SPDYC | DBP |
| -2.8339 | 4.60E-03 | GTExv8.EUR.Whole_Blood | 2.54E-02 | PGAM1P8 | DBP |
| 2.9933 | 2.76E-03 | GTExv8.EUR.Whole_Blood | 1.70E-02 | CAPN1 | DBP |
| -3.0107 | 2.61E-03 | GTExv8.EUR.Whole_Blood | 1.62E-02 | AP003068.23 | DBP |
| -4.1041 | 4.06E-05 | GTExv8.EUR.Whole_Blood | 5.03E-04 | FAM89B | DBP |
| 5.8446 | 5.08E-09 | GTExv8.EUR.Whole_Blood | 1.70E-07 | MAP3K11 | DBP |
| 11.5348 | 8.81E-31 | GTExv8.EUR.Whole_Blood | 3.67E-28 | SIPA1 | DBP |
| 3.9160 | 9.00E-05 | GTExv8.EUR.Whole_Blood | 1.01E-03 | KAT5 | DBP |
| -6.7067 | 1.99E-11 | GTExv8.EUR.Whole_Blood | 1.19E-09 | RNASEH2C | DBP |
| 2.9203 | 3.50E-03 | GTExv8.EUR.Whole_Blood | 2.06E-02 | KRT8P26 | DBP |
| 2.9425 | 3.26E-03 | GTExv8.EUR.Whole_Blood | 1.94E-02 | RP11-770G2.2 | DBP |
| 4.7966 | 1.61E-06 | GTExv8.EUR.Whole_Blood | 2.90E-05 | SNX32 | DBP |
| -5.2296 | 1.70E-07 | GTExv8.EUR.Whole_Blood | 3.94E-06 | MUS81 | DBP |
| -5.3994 | 6.68E-08 | GTExv8.EUR.Whole_Blood | 1.67E-06 | CFL1 | DBP |
| 2.8638 | 4.19E-03 | GTExv8.EUR.Whole_Blood | 2.36E-02 | EFEMP2 | DBP |
| -4.0506 | 5.11E-05 | GTExv8.EUR.Whole_Blood | 6.17E-04 | CTSW | DBP |
| 5.7217 | 1.05E-08 | GTExv8.EUR.Whole_Blood | 3.16E-07 | FIBP | DBP |
| -2.5559 | 1.06E-02 | GTExv8.EUR.Whole_Blood | 4.95E-02 | BANF1 | DBP |
| -3.0518 | 2.27E-03 | GTExv8.EUR.Whole_Blood | 1.45E-02 | ALDH3B1 | DBP |
| -4.2417 | 2.22E-05 | GTExv8.EUR.Whole_Blood | 3.02E-04 | RP11-802E16.3 | DBP |
| -3.9805 | 6.88E-05 | GTExv8.EUR.Whole_Blood | 8.06E-04 | C11orf24 | DBP |
| 2.9872 | 2.82E-03 | GTExv8.EUR.Whole_Blood | 1.73E-02 | TESMIN | DBP |
| -5.6505 | 1.60E-08 | GTExv8.EUR.Whole_Blood | 4.68E-07 | FADD | DBP |
| -3.8135 | 1.37E-04 | GTExv8.EUR.Whole_Blood | 1.45E-03 | NUMA1 | DBP |
| -4.8422 | 1.28E-06 | GTExv8.EUR.Whole_Blood | 2.38E-05 | CLPB | DBP |
| -4.3160 | 1.59E-05 | GTExv8.EUR.Whole_Blood | 2.25E-04 | ARHGEF17 | DBP |
| -5.0679 | 4.02E-07 | GTExv8.EUR.Whole_Blood | 8.49E-06 | RELT | DBP |
| -5.6043 | 2.09E-08 | GTExv8.EUR.Whole_Blood | 5.96E-07 | RP11-809N8.4 | DBP |
| -5.7944 | 6.86E-09 | GTExv8.EUR.Whole_Blood | 2.15E-07 | FAM168A | DBP |
| -4.6609 | 3.15E-06 | GTExv8.EUR.Whole_Blood | 5.27E-05 | RP11-147I3.1 | DBP |
| -5.5954 | 2.20E-08 | GTExv8.EUR.Whole_Blood | 6.20E-07 | AQP11 | DBP |
| -7.5517 | 4.30E-14 | GTExv8.EUR.Whole_Blood | 4.21E-12 | RSF1 | DBP |
| -5.6306 | 1.80E-08 | GTExv8.EUR.Whole_Blood | 5.22E-07 | RP11-91P24.6 | DBP |
| 3.4538 | 5.53E-04 | GTExv8.EUR.Whole_Blood | 4.63E-03 | ALG8 | DBP |
| 3.8748 | 1.07E-04 | GTExv8.EUR.Whole_Blood | 1.17E-03 | KCTD21-AS1 | DBP |
| -5.8429 | 5.13E-09 | GTExv8.EUR.Whole_Blood | 1.71E-07 | GAB2 | DBP |
| -4.1786 | 2.93E-05 | GTExv8.EUR.Whole_Blood | 3.83E-04 | RP11-452H21.4 | DBP |
| 4.1533 | 3.28E-05 | GTExv8.EUR.Whole_Blood | 4.21E-04 | NARS2 | DBP |
| 2.7686 | 5.63E-03 | GTExv8.EUR.Whole_Blood | 3.00E-02 | SLC25A1P1 | DBP |
| 2.5858 | 9.71E-03 | GTExv8.EUR.Whole_Blood | 4.61E-02 | HIKESHI | DBP |
| 2.9897 | 2.79E-03 | GTExv8.EUR.Whole_Blood | 1.71E-02 | RP11-121L10.2 | DBP |
| -3.1489 | 1.64E-03 | GTExv8.EUR.Whole_Blood | 1.12E-02 | C11orf54 | DBP |
| -5.4612 | 4.73E-08 | GTExv8.EUR.Whole_Blood | 1.22E-06 | RP11-819C21.1 | DBP |
| 6.1093 | 1.00E-09 | GTExv8.EUR.Whole_Blood | 3.87E-08 | CWF19L2 | DBP |
| 3.1526 | 1.62E-03 | GTExv8.EUR.Whole_Blood | 1.11E-02 | ALKBH8 | DBP |
| -2.5551 | 1.06E-02 | GTExv8.EUR.Whole_Blood | 4.95E-02 | ACAT1 | DBP |
| 6.1237 | 9.14E-10 | GTExv8.EUR.Whole_Blood | 3.57E-08 | SIK2 | DBP |
| -3.3331 | 8.59E-04 | GTExv8.EUR.Whole_Blood | 6.58E-03 | PPP2R1B | DBP |
| -4.2691 | 1.96E-05 | GTExv8.EUR.Whole_Blood | 2.72E-04 | ZPR1 | DBP |
| 3.6074 | 3.09E-04 | GTExv8.EUR.Whole_Blood | 2.84E-03 | RP11-109L13.1 | DBP |
| -6.4395 | 1.20E-10 | GTExv8.EUR.Whole_Blood | 5.94E-09 | SIDT2 | DBP |
| -3.9050 | 9.42E-05 | GTExv8.EUR.Whole_Blood | 1.05E-03 | TAGLN | DBP |
| -4.4856 | 7.27E-06 | GTExv8.EUR.Whole_Blood | 1.11E-04 | AP000892.6 | DBP |
| -4.3471 | 1.38E-05 | GTExv8.EUR.Whole_Blood | 1.97E-04 | CEP164 | DBP |
| -4.8280 | 1.38E-06 | GTExv8.EUR.Whole_Blood | 2.54E-05 | BACE1 | DBP |
| -3.8961 | 9.78E-05 | GTExv8.EUR.Whole_Blood | 1.09E-03 | UBASH3B | DBP |
| -3.2184 | 1.29E-03 | GTExv8.EUR.Whole_Blood | 9.19E-03 | SLC6A13 | DBP |
| -3.3872 | 7.06E-04 | GTExv8.EUR.Whole_Blood | 5.63E-03 | RP11-283I3.6 | DBP |
| -3.8076 | 1.40E-04 | GTExv8.EUR.Whole_Blood | 1.47E-03 | KDM5A | DBP |
| -3.8088 | 1.40E-04 | GTExv8.EUR.Whole_Blood | 1.47E-03 | CCDC77 | DBP |
| -2.5968 | 9.41E-03 | GTExv8.EUR.Whole_Blood | 4.51E-02 | RP5-1154L15.2 | DBP |
| -3.2084 | 1.33E-03 | GTExv8.EUR.Whole_Blood | 9.43E-03 | RAD52 | DBP |
| -3.0503 | 2.29E-03 | GTExv8.EUR.Whole_Blood | 1.46E-02 | AKAP3 | DBP |
| 2.8925 | 3.82E-03 | GTExv8.EUR.Whole_Blood | 2.20E-02 | PRH1 | DBP |
| 3.4497 | 5.61E-04 | GTExv8.EUR.Whole_Blood | 4.67E-03 | TAS2R14 | DBP |
| 3.5828 | 3.40E-04 | GTExv8.EUR.Whole_Blood | 3.08E-03 | TAS2R20 | DBP |
| 3.6460 | 2.66E-04 | GTExv8.EUR.Whole_Blood | 2.53E-03 | PRR4 | DBP |
| 3.2025 | 1.36E-03 | GTExv8.EUR.Whole_Blood | 9.59E-03 | LINC01252 | DBP |
| 5.7561 | 8.61E-09 | GTExv8.EUR.Whole_Blood | 2.62E-07 | APOLD1 | DBP |
| -2.8387 | 4.53E-03 | GTExv8.EUR.Whole_Blood | 2.52E-02 | PLEKHA5 | DBP |
| -4.1337 | 3.57E-05 | GTExv8.EUR.Whole_Blood | 4.54E-04 | RP11-582E3.6 | DBP |
| -3.7341 | 1.88E-04 | GTExv8.EUR.Whole_Blood | 1.87E-03 | GXYLT1 | DBP |
| -5.6639 | 1.48E-08 | GTExv8.EUR.Whole_Blood | 4.38E-07 | YAF2 | DBP |
| -2.6147 | 8.93E-03 | GTExv8.EUR.Whole_Blood | 4.32E-02 | PPHLN1 | DBP |
| -2.5520 | 1.07E-02 | GTExv8.EUR.Whole_Blood | 4.98E-02 | PUS7L | DBP |
| 3.5450 | 3.93E-04 | GTExv8.EUR.Whole_Blood | 3.47E-03 | IRAK4 | DBP |
| -4.7511 | 2.02E-06 | GTExv8.EUR.Whole_Blood | 3.56E-05 | RP1-197B17.3 | DBP |
| -4.1040 | 4.06E-05 | GTExv8.EUR.Whole_Blood | 5.03E-04 | RP1-197B17.7 | DBP |
| -4.1040 | 4.06E-05 | GTExv8.EUR.Whole_Blood | 5.03E-04 | RP1-197B17.4 | DBP |
| -5.4874 | 4.08E-08 | GTExv8.EUR.Whole_Blood | 1.07E-06 | SLC48A1 | DBP |
| -3.7780 | 1.58E-04 | GTExv8.EUR.Whole_Blood | 1.63E-03 | RP5-1057I20.4 | DBP |
| -3.9540 | 7.68E-05 | GTExv8.EUR.Whole_Blood | 8.81E-04 | RP5-1057I20.5 | DBP |
| 4.8433 | 1.28E-06 | GTExv8.EUR.Whole_Blood | 2.38E-05 | ASB8 | DBP |
| 4.3028 | 1.69E-05 | GTExv8.EUR.Whole_Blood | 2.37E-04 | RP11-370I10.12 | DBP |
| 3.1665 | 1.54E-03 | GTExv8.EUR.Whole_Blood | 1.06E-02 | H1FNT | DBP |
| 4.1813 | 2.90E-05 | GTExv8.EUR.Whole_Blood | 3.80E-04 | ZNF641 | DBP |
| 4.1738 | 3.00E-05 | GTExv8.EUR.Whole_Blood | 3.89E-04 | RP11-370I10.11 | DBP |
| -3.4215 | 6.23E-04 | GTExv8.EUR.Whole_Blood | 5.09E-03 | RP11-386G11.10 | DBP |
| -3.1644 | 1.55E-03 | GTExv8.EUR.Whole_Blood | 1.07E-02 | TUBA1A | DBP |
| 10.3828 | 2.97E-25 | GTExv8.EUR.Whole_Blood | 7.35E-23 | RP4-605O3.4 | DBP |
| -8.4422 | 3.12E-17 | GTExv8.EUR.Whole_Blood | 4.58E-15 | LIMA1 | DBP |
| -4.7535 | 2.00E-06 | GTExv8.EUR.Whole_Blood | 3.53E-05 | DIP2B | DBP |
| 10.1100 | 4.99E-24 | GTExv8.EUR.Whole_Blood | 1.13E-21 | ATF1 | DBP |
| 4.7637 | 1.90E-06 | GTExv8.EUR.Whole_Blood | 3.37E-05 | LETMD1 | DBP |
| 2.8978 | 3.76E-03 | GTExv8.EUR.Whole_Blood | 2.17E-02 | CSRNP2 | DBP |
| 5.6541 | 1.57E-08 | GTExv8.EUR.Whole_Blood | 4.61E-07 | EIF4B | DBP |
| -6.5753 | 4.86E-11 | GTExv8.EUR.Whole_Blood | 2.66E-09 | TNS2 | DBP |
| -5.8012 | 6.58E-09 | GTExv8.EUR.Whole_Blood | 2.09E-07 | SPRYD3 | DBP |
| 5.3450 | 9.04E-08 | GTExv8.EUR.Whole_Blood | 2.22E-06 | SOAT2 | DBP |
| -4.1101 | 3.95E-05 | GTExv8.EUR.Whole_Blood | 4.94E-04 | ZNF740 | DBP |
| 3.9542 | 7.68E-05 | GTExv8.EUR.Whole_Blood | 8.81E-04 | SUOX | DBP |
| -4.8713 | 1.11E-06 | GTExv8.EUR.Whole_Blood | 2.11E-05 | RPS26 | DBP |
| 2.5754 | 1.00E-02 | GTExv8.EUR.Whole_Blood | 4.72E-02 | TBC1D30 | DBP |
| 4.2416 | 2.22E-05 | GTExv8.EUR.Whole_Blood | 3.02E-04 | LINC01481 | DBP |
| 6.8771 | 6.11E-12 | GTExv8.EUR.Whole_Blood | 4.07E-10 | GALNT4 | DBP |
| 13.3319 | 1.51E-40 | GTExv8.EUR.Whole_Blood | 1.20E-37 | POC1B | DBP |
| -7.4469 | 9.56E-14 | GTExv8.EUR.Whole_Blood | 8.91E-12 | RP11-981P6.1 | DBP |
| 4.1357 | 3.54E-05 | GTExv8.EUR.Whole_Blood | 4.52E-04 | MRPL42 | DBP |
| -2.8782 | 4.00E-03 | GTExv8.EUR.Whole_Blood | 2.28E-02 | AMDHD1 | DBP |
| 3.3568 | 7.89E-04 | GTExv8.EUR.Whole_Blood | 6.17E-03 | WASHC3 | DBP |
| 3.8823 | 1.03E-04 | GTExv8.EUR.Whole_Blood | 1.13E-03 | TCP11L2 | DBP |
| 4.0780 | 4.54E-05 | GTExv8.EUR.Whole_Blood | 5.55E-04 | USP30-AS1 | DBP |
| 3.3364 | 8.49E-04 | GTExv8.EUR.Whole_Blood | 6.52E-03 | ACACB | DBP |
| 3.9030 | 9.50E-05 | GTExv8.EUR.Whole_Blood | 1.06E-03 | MMAB | DBP |
| 5.7152 | 1.10E-08 | GTExv8.EUR.Whole_Blood | 3.29E-07 | TRPV4 | DBP |
| 4.9075 | 9.23E-07 | GTExv8.EUR.Whole_Blood | 1.81E-05 | ANKRD13A | DBP |
| 6.7430 | 1.55E-11 | GTExv8.EUR.Whole_Blood | 9.60E-10 | ARPC3 | DBP |
| 7.0199 | 2.22E-12 | GTExv8.EUR.Whole_Blood | 1.68E-10 | FAM216A | DBP |
| 7.0222 | 2.18E-12 | GTExv8.EUR.Whole_Blood | 1.66E-10 | GPN3 | DBP |
| -5.9783 | 2.26E-09 | GTExv8.EUR.Whole_Blood | 8.07E-08 | VPS29 | DBP |
| -6.0664 | 1.31E-09 | GTExv8.EUR.Whole_Blood | 4.90E-08 | RP3-424M6.4 | DBP |
| -5.9783 | 2.26E-09 | GTExv8.EUR.Whole_Blood | 8.07E-08 | RAD9B | DBP |
| -10.7295 | 7.40E-27 | GTExv8.EUR.Whole_Blood | 2.17E-24 | ALDH2 | DBP |
| -14.7129 | 5.33E-49 | GTExv8.EUR.Whole_Blood | 7.04E-46 | MAPKAPK5-AS1 | DBP |
| 11.0980 | 1.28E-28 | GTExv8.EUR.Whole_Blood | 4.23E-26 | MAPKAPK5 | DBP |
| -10.6271 | 2.23E-26 | GTExv8.EUR.Whole_Blood | 5.89E-24 | ADAM1B | DBP |
| -10.9597 | 5.97E-28 | GTExv8.EUR.Whole_Blood | 1.82E-25 | TMEM116 | DBP |
| -13.1838 | 1.09E-39 | GTExv8.EUR.Whole_Blood | 6.17E-37 | NAA25 | DBP |
| -2.7516 | 5.93E-03 | GTExv8.EUR.Whole_Blood | 3.11E-02 | RPH3A | DBP |
| 3.3377 | 8.45E-04 | GTExv8.EUR.Whole_Blood | 6.52E-03 | OAS1 | DBP |
| -2.8150 | 4.88E-03 | GTExv8.EUR.Whole_Blood | 2.67E-02 | RASAL1 | DBP |
| 3.8178 | 1.35E-04 | GTExv8.EUR.Whole_Blood | 1.43E-03 | RITA1 | DBP |
| -3.6362 | 2.77E-04 | GTExv8.EUR.Whole_Blood | 2.61E-03 | IQCD | DBP |
| 2.8189 | 4.82E-03 | GTExv8.EUR.Whole_Blood | 2.64E-02 | PLBD2 | DBP |
| -2.6775 | 7.42E-03 | GTExv8.EUR.Whole_Blood | 3.71E-02 | PRKAB1 | DBP |
| -5.4635 | 4.67E-08 | GTExv8.EUR.Whole_Blood | 1.21E-06 | GCN1 | DBP |
| -2.9574 | 3.10E-03 | GTExv8.EUR.Whole_Blood | 1.87E-02 | COQ5 | DBP |
| 3.5582 | 3.73E-04 | GTExv8.EUR.Whole_Blood | 3.33E-03 | SPPL3 | DBP |
| -3.2691 | 1.08E-03 | GTExv8.EUR.Whole_Blood | 7.95E-03 | ANAPC5 | DBP |
| -3.3350 | 8.53E-04 | GTExv8.EUR.Whole_Blood | 6.54E-03 | PSMD9 | DBP |
| -5.7806 | 7.44E-09 | GTExv8.EUR.Whole_Blood | 2.31E-07 | LRRC43 | DBP |
| -3.7089 | 2.08E-04 | GTExv8.EUR.Whole_Blood | 2.04E-03 | ABCB9 | DBP |
| 4.8965 | 9.76E-07 | GTExv8.EUR.Whole_Blood | 1.90E-05 | ARL6IP4 | DBP |
| 4.6306 | 3.65E-06 | GTExv8.EUR.Whole_Blood | 6.01E-05 | C12orf65 | DBP |
| 4.9748 | 6.53E-07 | GTExv8.EUR.Whole_Blood | 1.31E-05 | RP11-282O18.3 | DBP |
| 7.2434 | 4.37E-13 | GTExv8.EUR.Whole_Blood | 3.68E-11 | CDK2AP1 | DBP |
| -5.6206 | 1.90E-08 | GTExv8.EUR.Whole_Blood | 5.47E-07 | KMT5A | DBP |
| -3.2557 | 1.13E-03 | GTExv8.EUR.Whole_Blood | 8.22E-03 | RILPL2 | DBP |
| 3.9355 | 8.30E-05 | GTExv8.EUR.Whole_Blood | 9.41E-04 | ZNF605 | DBP |
| 4.0566 | 4.98E-05 | GTExv8.EUR.Whole_Blood | 6.03E-04 | ZDHHC20 | DBP |
| -2.6135 | 8.96E-03 | GTExv8.EUR.Whole_Blood | 4.33E-02 | SACS | DBP |
| 3.3131 | 9.23E-04 | GTExv8.EUR.Whole_Blood | 6.99E-03 | MTMR6 | DBP |
| -3.5158 | 4.38E-04 | GTExv8.EUR.Whole_Blood | 3.81E-03 | RASL11A | DBP |
| 3.1100 | 1.87E-03 | GTExv8.EUR.Whole_Blood | 1.24E-02 | GTF3A | DBP |
| 3.3297 | 8.69E-04 | GTExv8.EUR.Whole_Blood | 6.63E-03 | SLC7A1 | DBP |
| -2.7589 | 5.80E-03 | GTExv8.EUR.Whole_Blood | 3.07E-02 | EXOSC8 | DBP |
| 3.3019 | 9.60E-04 | GTExv8.EUR.Whole_Blood | 7.20E-03 | FOXO1 | DBP |
| -2.9690 | 2.99E-03 | GTExv8.EUR.Whole_Blood | 1.81E-02 | CYCSP34 | DBP |
| 5.8391 | 5.25E-09 | GTExv8.EUR.Whole_Blood | 1.74E-07 | MTRF1 | DBP |
| 3.5859 | 3.36E-04 | GTExv8.EUR.Whole_Blood | 3.05E-03 | CCDC122 | DBP |
| -4.7446 | 2.09E-06 | GTExv8.EUR.Whole_Blood | 3.66E-05 | LRCH1 | DBP |
| 5.7796 | 7.49E-09 | GTExv8.EUR.Whole_Blood | 2.32E-07 | TRIM13 | DBP |
| -4.7000 | 2.60E-06 | GTExv8.EUR.Whole_Blood | 4.46E-05 | RBM26 | DBP |
| 3.5334 | 4.10E-04 | GTExv8.EUR.Whole_Blood | 3.61E-03 | DNAJC3-AS1 | DBP |
| 2.8319 | 4.63E-03 | GTExv8.EUR.Whole_Blood | 2.55E-02 | DNAJC3 | DBP |
| -2.7016 | 6.90E-03 | GTExv8.EUR.Whole_Blood | 3.52E-02 | RAP2A | DBP |
| -3.5597 | 3.71E-04 | GTExv8.EUR.Whole_Blood | 3.32E-03 | PROZ | DBP |
| 3.0583 | 2.23E-03 | GTExv8.EUR.Whole_Blood | 1.43E-02 | PCID2 | DBP |
| 3.0749 | 2.11E-03 | GTExv8.EUR.Whole_Blood | 1.37E-02 | GAS6-AS1 | DBP |
| 3.7701 | 1.63E-04 | GTExv8.EUR.Whole_Blood | 1.67E-03 | ABC13-47488600E17.1 | DBP |
| 6.1639 | 7.10E-10 | GTExv8.EUR.Whole_Blood | 2.89E-08 | RASA3 | DBP |
| 7.2275 | 4.92E-13 | GTExv8.EUR.Whole_Blood | 4.10E-11 | CDC16 | DBP |
| 6.3637 | 1.97E-10 | GTExv8.EUR.Whole_Blood | 9.02E-09 | UPF3A | DBP |
| 2.8238 | 4.75E-03 | GTExv8.EUR.Whole_Blood | 2.61E-02 | PARP2 | DBP |
| 4.5224 | 6.12E-06 | GTExv8.EUR.Whole_Blood | 9.57E-05 | TGM1 | DBP |
| -3.4481 | 5.65E-04 | GTExv8.EUR.Whole_Blood | 4.70E-03 | DHRS1 | DBP |
| -3.0462 | 2.32E-03 | GTExv8.EUR.Whole_Blood | 1.48E-02 | LTB4R | DBP |
| 2.5680 | 1.02E-02 | GTExv8.EUR.Whole_Blood | 4.80E-02 | RIPK3 | DBP |
| -3.4945 | 4.75E-04 | GTExv8.EUR.Whole_Blood | 4.07E-03 | AP4S1 | DBP |
| -3.2876 | 1.01E-03 | GTExv8.EUR.Whole_Blood | 7.54E-03 | ARHGAP5 | DBP |
| -2.5719 | 1.01E-02 | GTExv8.EUR.Whole_Blood | 4.76E-02 | RALGAPA1 | DBP |
| -3.1446 | 1.66E-03 | GTExv8.EUR.Whole_Blood | 1.13E-02 | STYX | DBP |
| 5.3352 | 9.54E-08 | GTExv8.EUR.Whole_Blood | 2.33E-06 | GCH1 | DBP |
| -3.2626 | 1.10E-03 | GTExv8.EUR.Whole_Blood | 8.04E-03 | PSMA3-AS1 | DBP |
| -3.7754 | 1.60E-04 | GTExv8.EUR.Whole_Blood | 1.65E-03 | GPR135 | DBP |
| 4.1359 | 3.54E-05 | GTExv8.EUR.Whole_Blood | 4.52E-04 | L3HYPDH | DBP |
| -2.5539 | 1.07E-02 | GTExv8.EUR.Whole_Blood | 4.98E-02 | DHRS7 | DBP |
| -3.9710 | 7.16E-05 | GTExv8.EUR.Whole_Blood | 8.32E-04 | PLEKHG3 | DBP |
| -4.6441 | 3.41E-06 | GTExv8.EUR.Whole_Blood | 5.68E-05 | TMEM229B | DBP |
| -2.9594 | 3.08E-03 | GTExv8.EUR.Whole_Blood | 1.86E-02 | RP1-292L20.3 | DBP |
| 2.8884 | 3.87E-03 | GTExv8.EUR.Whole_Blood | 2.23E-02 | SIPA1L1 | DBP |
| -3.8048 | 1.42E-04 | GTExv8.EUR.Whole_Blood | 1.49E-03 | RGS6 | DBP |
| -3.9725 | 7.11E-05 | GTExv8.EUR.Whole_Blood | 8.27E-04 | DCAF4 | DBP |
| 4.0636 | 4.83E-05 | GTExv8.EUR.Whole_Blood | 5.86E-04 | PSEN1 | DBP |
| 3.3909 | 6.97E-04 | GTExv8.EUR.Whole_Blood | 5.57E-03 | PAPLN | DBP |
| 5.1192 | 3.07E-07 | GTExv8.EUR.Whole_Blood | 6.70E-06 | RP4-647C14.2 | DBP |
| 3.1371 | 1.71E-03 | GTExv8.EUR.Whole_Blood | 1.16E-02 | RP4-647C14.3 | DBP |
| 2.5895 | 9.61E-03 | GTExv8.EUR.Whole_Blood | 4.58E-02 | PNMA1 | DBP |
| -2.6119 | 9.00E-03 | GTExv8.EUR.Whole_Blood | 4.35E-02 | COQ6 | DBP |
| -2.5873 | 9.67E-03 | GTExv8.EUR.Whole_Blood | 4.60E-02 | FAM161B | DBP |
| -3.3629 | 7.71E-04 | GTExv8.EUR.Whole_Blood | 6.06E-03 | LTBP2 | DBP |
| 2.5941 | 9.48E-03 | GTExv8.EUR.Whole_Blood | 4.53E-02 | MLH3 | DBP |
| 2.8161 | 4.86E-03 | GTExv8.EUR.Whole_Blood | 2.66E-02 | NEK9 | DBP |
| -4.8428 | 1.28E-06 | GTExv8.EUR.Whole_Blood | 2.38E-05 | LINC01629 | DBP |
| -4.8428 | 1.28E-06 | GTExv8.EUR.Whole_Blood | 2.38E-05 | RP11-7F17.5 | DBP |
| -4.8428 | 1.28E-06 | GTExv8.EUR.Whole_Blood | 2.38E-05 | RP11-7F17.3 | DBP |
| -3.2522 | 1.15E-03 | GTExv8.EUR.Whole_Blood | 8.33E-03 | CIPC | DBP |
| -2.9221 | 3.48E-03 | GTExv8.EUR.Whole_Blood | 2.05E-02 | TC2N | DBP |
| -3.7884 | 1.52E-04 | GTExv8.EUR.Whole_Blood | 1.58E-03 | SLC25A29 | DBP |
| -3.6685 | 2.44E-04 | GTExv8.EUR.Whole_Blood | 2.36E-03 | RP11-638I2.8 | DBP |
| -3.8741 | 1.07E-04 | GTExv8.EUR.Whole_Blood | 1.17E-03 | RP11-638I2.10 | DBP |
| -4.1093 | 3.97E-05 | GTExv8.EUR.Whole_Blood | 4.95E-04 | WARS | DBP |
| 2.5860 | 9.71E-03 | GTExv8.EUR.Whole_Blood | 4.61E-02 | RP11-1029J19.4 | DBP |
| 2.6287 | 8.57E-03 | GTExv8.EUR.Whole_Blood | 4.19E-02 | LINC00239 | DBP |
| 2.6850 | 7.25E-03 | GTExv8.EUR.Whole_Blood | 3.66E-02 | PPP2R5C | DBP |
| -2.6648 | 7.70E-03 | GTExv8.EUR.Whole_Blood | 3.84E-02 | EIF5 | DBP |
| -3.7410 | 1.83E-04 | GTExv8.EUR.Whole_Blood | 1.83E-03 | RPL10AP1 | DBP |
| 4.8617 | 1.16E-06 | GTExv8.EUR.Whole_Blood | 2.18E-05 | BAG5 | DBP |
| 4.6424 | 3.44E-06 | GTExv8.EUR.Whole_Blood | 5.71E-05 | XRCC3 | DBP |
| -2.6033 | 9.23E-03 | GTExv8.EUR.Whole_Blood | 4.43E-02 | RN7SL634P | DBP |
| 3.9765 | 6.99E-05 | GTExv8.EUR.Whole_Blood | 8.17E-04 | KIF26A | DBP |
| 2.7645 | 5.70E-03 | GTExv8.EUR.Whole_Blood | 3.03E-02 | PLD4 | DBP |
| 2.7478 | 6.00E-03 | GTExv8.EUR.Whole_Blood | 3.14E-02 | MTMR10 | DBP |
| -5.6003 | 2.14E-08 | GTExv8.EUR.Whole_Blood | 6.06E-07 | EIF2AK4 | DBP |
| -7.7753 | 7.53E-15 | GTExv8.EUR.Whole_Blood | 8.06E-13 | CHP1 | DBP |
| 6.8052 | 1.01E-11 | GTExv8.EUR.Whole_Blood | 6.56E-10 | RTF1 | DBP |
| 4.6675 | 3.05E-06 | GTExv8.EUR.Whole_Blood | 5.14E-05 | ITPKA | DBP |
| -3.0936 | 1.98E-03 | GTExv8.EUR.Whole_Blood | 1.30E-02 | RPAP1 | DBP |
| 8.0037 | 1.21E-15 | GTExv8.EUR.Whole_Blood | 1.52E-13 | JMJD7 | DBP |
| 9.0264 | 1.78E-19 | GTExv8.EUR.Whole_Blood | 3.21E-17 | PLA2G4B | DBP |
| 3.0678 | 2.16E-03 | GTExv8.EUR.Whole_Blood | 1.39E-02 | SPTBN5 | DBP |
| -2.5926 | 9.52E-03 | GTExv8.EUR.Whole_Blood | 4.54E-02 | EHD4 | DBP |
| -2.9701 | 2.98E-03 | GTExv8.EUR.Whole_Blood | 1.80E-02 | ADAL | DBP |
| -5.8242 | 5.74E-09 | GTExv8.EUR.Whole_Blood | 1.87E-07 | ZSCAN29 | DBP |
| -2.7716 | 5.58E-03 | GTExv8.EUR.Whole_Blood | 2.97E-02 | CATSPER2 | DBP |
| -6.4688 | 9.88E-11 | GTExv8.EUR.Whole_Blood | 5.05E-09 | PDIA3 | DBP |
| -6.1035 | 1.04E-09 | GTExv8.EUR.Whole_Blood | 3.98E-08 | SERF2 | DBP |
| -5.0923 | 3.54E-07 | GTExv8.EUR.Whole_Blood | 7.60E-06 | FBN1 | DBP |
| 2.9961 | 2.73E-03 | GTExv8.EUR.Whole_Blood | 1.68E-02 | GALK2 | DBP |
| -3.1363 | 1.71E-03 | GTExv8.EUR.Whole_Blood | 1.16E-02 | TNFAIP8L3 | DBP |
| -3.2921 | 9.95E-04 | GTExv8.EUR.Whole_Blood | 7.45E-03 | CYP19A1 | DBP |
| 2.6837 | 7.28E-03 | GTExv8.EUR.Whole_Blood | 3.67E-02 | TMOD2 | DBP |
| 3.7741 | 1.61E-04 | GTExv8.EUR.Whole_Blood | 1.65E-03 | C15orf65 | DBP |
| 2.5788 | 9.91E-03 | GTExv8.EUR.Whole_Blood | 4.69E-02 | RPS27L | DBP |
| 4.1546 | 3.26E-05 | GTExv8.EUR.Whole_Blood | 4.19E-04 | USP3 | DBP |
| 2.9839 | 2.85E-03 | GTExv8.EUR.Whole_Blood | 1.74E-02 | DPP8 | DBP |
| 2.7371 | 6.20E-03 | GTExv8.EUR.Whole_Blood | 3.23E-02 | TIPIN | DBP |
| 3.5506 | 3.84E-04 | GTExv8.EUR.Whole_Blood | 3.42E-03 | MAP2K5 | DBP |
| 2.7107 | 6.71E-03 | GTExv8.EUR.Whole_Blood | 3.44E-02 | SKOR1 | DBP |
| 5.0232 | 5.08E-07 | GTExv8.EUR.Whole_Blood | 1.05E-05 | PIAS1 | DBP |
| 5.4313 | 5.60E-08 | GTExv8.EUR.Whole_Blood | 1.43E-06 | CALML4 | DBP |
| 5.4140 | 6.16E-08 | GTExv8.EUR.Whole_Blood | 1.55E-06 | CLN6 | DBP |
| -4.6653 | 3.08E-06 | GTExv8.EUR.Whole_Blood | 5.17E-05 | PARP6 | DBP |
| 17.4542 | 3.20E-68 | GTExv8.EUR.Whole_Blood | 8.45E-65 | ULK3 | DBP |
| -16.0205 | 9.19E-58 | GTExv8.EUR.Whole_Blood | 1.82E-54 | SCAMP2 | DBP |
| -18.4421 | 6.03E-76 | GTExv8.EUR.Whole_Blood | 2.39E-72 | MPI | DBP |
| 8.6555 | 4.91E-18 | GTExv8.EUR.Whole_Blood | 7.48E-16 | PPCDC | DBP |
| 3.7446 | 1.81E-04 | GTExv8.EUR.Whole_Blood | 1.81E-03 | RP11-69H7.2 | DBP |
| 2.9864 | 2.82E-03 | GTExv8.EUR.Whole_Blood | 1.73E-02 | RP11-797A18.5 | DBP |
| 3.1533 | 1.61E-03 | GTExv8.EUR.Whole_Blood | 1.10E-02 | RP11-797A18.6 | DBP |
| 3.3404 | 8.37E-04 | GTExv8.EUR.Whole_Blood | 6.46E-03 | IREB2 | DBP |
| 3.0805 | 2.07E-03 | GTExv8.EUR.Whole_Blood | 1.35E-02 | PSMA4 | DBP |
| 4.8077 | 1.53E-06 | GTExv8.EUR.Whole_Blood | 2.78E-05 | MORF4L1 | DBP |
| 2.6257 | 8.65E-03 | GTExv8.EUR.Whole_Blood | 4.22E-02 | KIAA1024 | DBP |
| -4.7377 | 2.16E-06 | GTExv8.EUR.Whole_Blood | 3.77E-05 | ABHD17C | DBP |
| 2.6814 | 7.33E-03 | GTExv8.EUR.Whole_Blood | 3.69E-02 | UBE2Q2P2 | DBP |
| -3.5574 | 3.75E-04 | GTExv8.EUR.Whole_Blood | 3.34E-03 | AP3B2 | DBP |
| 4.1943 | 2.74E-05 | GTExv8.EUR.Whole_Blood | 3.63E-04 | C15orf40 | DBP |
| -3.2907 | 9.99E-04 | GTExv8.EUR.Whole_Blood | 7.47E-03 | GOLGA6L5P | DBP |
| -2.6231 | 8.71E-03 | GTExv8.EUR.Whole_Blood | 4.24E-02 | UBE2Q2P1 | DBP |
| 5.5474 | 2.90E-08 | GTExv8.EUR.Whole_Blood | 7.95E-07 | RP11-182J1.18 | DBP |
| -4.4511 | 8.54E-06 | GTExv8.EUR.Whole_Blood | 1.28E-04 | CIB1 | DBP |
| -3.4979 | 4.69E-04 | GTExv8.EUR.Whole_Blood | 4.03E-03 | GDPGP1 | DBP |
| 3.3586 | 7.83E-04 | GTExv8.EUR.Whole_Blood | 6.13E-03 | IQGAP1 | DBP |
| -4.7920 | 1.65E-06 | GTExv8.EUR.Whole_Blood | 2.96E-05 | CRTC3 | DBP |
| -19.5261 | 6.59E-85 | GTExv8.EUR.Whole_Blood | 5.22E-81 | FES | DBP |
| 4.5828 | 4.59E-06 | GTExv8.EUR.Whole_Blood | 7.36E-05 | MAN2A2 | DBP |
| 3.1141 | 1.85E-03 | GTExv8.EUR.Whole_Blood | 1.23E-02 | PRC1-AS1 | DBP |
| -2.8846 | 3.92E-03 | GTExv8.EUR.Whole_Blood | 2.24E-02 | LYSMD4 | DBP |
| 3.4365 | 5.89E-04 | GTExv8.EUR.Whole_Blood | 4.87E-03 | CHSY1 | DBP |
| 3.4298 | 6.04E-04 | GTExv8.EUR.Whole_Blood | 4.96E-03 | PIGQ | DBP |
| 3.1106 | 1.87E-03 | GTExv8.EUR.Whole_Blood | 1.24E-02 | RAB40C | DBP |
| 3.6936 | 2.21E-04 | GTExv8.EUR.Whole_Blood | 2.16E-03 | WFIKKN1 | DBP |
| 5.0521 | 4.37E-07 | GTExv8.EUR.Whole_Blood | 9.14E-06 | METTL26 | DBP |
| 2.8453 | 4.44E-03 | GTExv8.EUR.Whole_Blood | 2.47E-02 | RHOT2 | DBP |
| 4.9929 | 5.95E-07 | GTExv8.EUR.Whole_Blood | 1.20E-05 | RHBDL1 | DBP |
| 3.3151 | 9.16E-04 | GTExv8.EUR.Whole_Blood | 6.95E-03 | STUB1 | DBP |
| 4.7234 | 2.32E-06 | GTExv8.EUR.Whole_Blood | 4.01E-05 | WDR24 | DBP |
| 2.6147 | 8.93E-03 | GTExv8.EUR.Whole_Blood | 4.32E-02 | METRN | DBP |
| 3.7616 | 1.69E-04 | GTExv8.EUR.Whole_Blood | 1.71E-03 | NARFL | DBP |
| 4.2751 | 1.91E-05 | GTExv8.EUR.Whole_Blood | 2.66E-04 | BAIAP3 | DBP |
| 2.5715 | 1.01E-02 | GTExv8.EUR.Whole_Blood | 4.76E-02 | UNKL | DBP |
| 3.6563 | 2.56E-04 | GTExv8.EUR.Whole_Blood | 2.46E-03 | LA16c-312E8.4 | DBP |
| 4.4406 | 8.97E-06 | GTExv8.EUR.Whole_Blood | 1.33E-04 | CCDC154 | DBP |
| -4.2323 | 2.31E-05 | GTExv8.EUR.Whole_Blood | 3.13E-04 | LA16c-390E6.4 | DBP |
| -3.6635 | 2.49E-04 | GTExv8.EUR.Whole_Blood | 2.40E-03 | CLCN7 | DBP |
| -2.7964 | 5.17E-03 | GTExv8.EUR.Whole_Blood | 2.79E-02 | CRAMP1 | DBP |
| -3.8542 | 1.16E-04 | GTExv8.EUR.Whole_Blood | 1.25E-03 | RPS2 | DBP |
| -5.0049 | 5.59E-07 | GTExv8.EUR.Whole_Blood | 1.13E-05 | SYNGR3 | DBP |
| -2.5887 | 9.63E-03 | GTExv8.EUR.Whole_Blood | 4.59E-02 | HCFC1R1 | DBP |
| -2.6775 | 7.42E-03 | GTExv8.EUR.Whole_Blood | 3.71E-02 | IL32 | DBP |
| -4.7953 | 1.62E-06 | GTExv8.EUR.Whole_Blood | 2.91E-05 | ZNF263 | DBP |
| 3.0374 | 2.39E-03 | GTExv8.EUR.Whole_Blood | 1.51E-02 | ZNF75A | DBP |
| -4.5028 | 6.71E-06 | GTExv8.EUR.Whole_Blood | 1.04E-04 | NAA60 | DBP |
| 3.9031 | 9.50E-05 | GTExv8.EUR.Whole_Blood | 1.06E-03 | TRAP1 | DBP |
| -3.7650 | 1.67E-04 | GTExv8.EUR.Whole_Blood | 1.70E-03 | ADCY9 | DBP |
| 4.7608 | 1.93E-06 | GTExv8.EUR.Whole_Blood | 3.41E-05 | MGRN1 | DBP |
| -4.7764 | 1.78E-06 | GTExv8.EUR.Whole_Blood | 3.18E-05 | ZNF500 | DBP |
| -6.9430 | 3.84E-12 | GTExv8.EUR.Whole_Blood | 2.67E-10 | SEPT12 | DBP |
| -6.1725 | 6.72E-10 | GTExv8.EUR.Whole_Blood | 2.76E-08 | SMIM22 | DBP |
| -7.1465 | 8.90E-13 | GTExv8.EUR.Whole_Blood | 7.27E-11 | RP11-127I20.5 | DBP |
| -3.5732 | 3.53E-04 | GTExv8.EUR.Whole_Blood | 3.17E-03 | ROGDI | DBP |
| 5.6572 | 1.54E-08 | GTExv8.EUR.Whole_Blood | 4.54E-07 | GLYR1 | DBP |
| -3.2648 | 1.10E-03 | GTExv8.EUR.Whole_Blood | 8.04E-03 | RP11-876N24.1 | DBP |
| 3.1039 | 1.91E-03 | GTExv8.EUR.Whole_Blood | 1.26E-02 | LITAF | DBP |
| 3.3989 | 6.77E-04 | GTExv8.EUR.Whole_Blood | 5.44E-03 | NPIPB2 | DBP |
| -4.4155 | 1.01E-05 | GTExv8.EUR.Whole_Blood | 1.48E-04 | BFAR | DBP |
| 3.5996 | 3.19E-04 | GTExv8.EUR.Whole_Blood | 2.93E-03 | NDE1 | DBP |
| 3.2311 | 1.23E-03 | GTExv8.EUR.Whole_Blood | 8.81E-03 | CTB-193M12.5 | DBP |
| -3.6319 | 2.81E-04 | GTExv8.EUR.Whole_Blood | 2.64E-03 | AF001548.5 | DBP |
| -3.5996 | 3.19E-04 | GTExv8.EUR.Whole_Blood | 2.93E-03 | AF001548.6 | DBP |
| 3.2071 | 1.34E-03 | GTExv8.EUR.Whole_Blood | 9.48E-03 | THUMPD1 | DBP |
| 4.1030 | 4.08E-05 | GTExv8.EUR.Whole_Blood | 5.04E-04 | AC004381.6 | DBP |
| 2.8764 | 4.02E-03 | GTExv8.EUR.Whole_Blood | 2.29E-02 | METTL9 | DBP |
| 2.9568 | 3.11E-03 | GTExv8.EUR.Whole_Blood | 1.87E-02 | CTB-31N19.3 | DBP |
| -4.5851 | 4.54E-06 | GTExv8.EUR.Whole_Blood | 7.31E-05 | SLC5A11 | DBP |
| 2.8937 | 3.81E-03 | GTExv8.EUR.Whole_Blood | 2.20E-02 | RP11-266L9.4 | DBP |
| 3.1891 | 1.43E-03 | GTExv8.EUR.Whole_Blood | 9.99E-03 | GSG1L | DBP |
| 2.7768 | 5.49E-03 | GTExv8.EUR.Whole_Blood | 2.94E-02 | XPO6 | DBP |
| 3.7263 | 1.94E-04 | GTExv8.EUR.Whole_Blood | 1.92E-03 | BOLA2 | DBP |
| -2.7396 | 6.15E-03 | GTExv8.EUR.Whole_Blood | 3.21E-02 | INO80E | DBP |
| -2.6911 | 7.12E-03 | GTExv8.EUR.Whole_Blood | 3.60E-02 | TBX6 | DBP |
| -6.4058 | 1.50E-10 | GTExv8.EUR.Whole_Blood | 7.12E-09 | RP11-455F5.4 | DBP |
| 5.8367 | 5.32E-09 | GTExv8.EUR.Whole_Blood | 1.76E-07 | RP11-455F5.6 | DBP |
| 4.4749 | 7.64E-06 | GTExv8.EUR.Whole_Blood | 1.16E-04 | GDPD3 | DBP |
| 2.7337 | 6.26E-03 | GTExv8.EUR.Whole_Blood | 3.26E-02 | NPIPB13 | DBP |
| 2.7292 | 6.35E-03 | GTExv8.EUR.Whole_Blood | 3.29E-02 | SEPT1 | DBP |
| 4.8134 | 1.48E-06 | GTExv8.EUR.Whole_Blood | 2.70E-05 | RNF40 | DBP |
| -5.3365 | 9.48E-08 | GTExv8.EUR.Whole_Blood | 2.32E-06 | RP11-1072A3.3 | DBP |
| -5.4890 | 4.04E-08 | GTExv8.EUR.Whole_Blood | 1.06E-06 | HSD3B7 | DBP |
| 5.2799 | 1.29E-07 | GTExv8.EUR.Whole_Blood | 3.06E-06 | RP11-196G11.2 | DBP |
| -5.9946 | 2.04E-09 | GTExv8.EUR.Whole_Blood | 7.45E-08 | RP11-196G11.6 | DBP |
| -5.3838 | 7.29E-08 | GTExv8.EUR.Whole_Blood | 1.81E-06 | ZNF668 | DBP |
| -5.2937 | 1.20E-07 | GTExv8.EUR.Whole_Blood | 2.87E-06 | PRSS53 | DBP |
| -6.3448 | 2.23E-10 | GTExv8.EUR.Whole_Blood | 1.01E-08 | VKORC1 | DBP |
| -5.1893 | 2.11E-07 | GTExv8.EUR.Whole_Blood | 4.80E-06 | BCKDK | DBP |
| 6.7585 | 1.39E-11 | GTExv8.EUR.Whole_Blood | 8.67E-10 | KAT8 | DBP |
| 5.5350 | 3.11E-08 | GTExv8.EUR.Whole_Blood | 8.47E-07 | RP11-196G11.4 | DBP |
| -6.7585 | 1.39E-11 | GTExv8.EUR.Whole_Blood | 8.67E-10 | RP11-196G11.5 | DBP |
| -2.8918 | 3.83E-03 | GTExv8.EUR.Whole_Blood | 2.21E-02 | ITGAX | DBP |
| -3.7892 | 1.51E-04 | GTExv8.EUR.Whole_Blood | 1.57E-03 | AKTIP | DBP |
| 2.6946 | 7.05E-03 | GTExv8.EUR.Whole_Blood | 3.58E-02 | AMFR | DBP |
| 3.9963 | 6.44E-05 | GTExv8.EUR.Whole_Blood | 7.59E-04 | BBS2 | DBP |
| -3.3013 | 9.62E-04 | GTExv8.EUR.Whole_Blood | 7.20E-03 | C16orf70 | DBP |
| 3.4065 | 6.58E-04 | GTExv8.EUR.Whole_Blood | 5.31E-03 | B3GNT9 | DBP |
| 4.4473 | 8.70E-06 | GTExv8.EUR.Whole_Blood | 1.30E-04 | KIAA0895L | DBP |
| 4.8394 | 1.30E-06 | GTExv8.EUR.Whole_Blood | 2.41E-05 | FHOD1 | DBP |
| 3.6250 | 2.89E-04 | GTExv8.EUR.Whole_Blood | 2.70E-03 | CTRL | DBP |
| -2.7326 | 6.28E-03 | GTExv8.EUR.Whole_Blood | 3.26E-02 | DPEP3 | DBP |
| -3.0706 | 2.14E-03 | GTExv8.EUR.Whole_Blood | 1.38E-02 | NFATC3 | DBP |
| -3.2710 | 1.07E-03 | GTExv8.EUR.Whole_Blood | 7.89E-03 | SLC7A6 | DBP |
| -3.3284 | 8.74E-04 | GTExv8.EUR.Whole_Blood | 6.66E-03 | RP11-96D1.8 | DBP |
| -2.6059 | 9.16E-03 | GTExv8.EUR.Whole_Blood | 4.41E-02 | TANGO6 | DBP |
| -6.8105 | 9.73E-12 | GTExv8.EUR.Whole_Blood | 6.37E-10 | SNTB2 | DBP |
| 6.1351 | 8.51E-10 | GTExv8.EUR.Whole_Blood | 3.38E-08 | PDF | DBP |
| -6.6340 | 3.27E-11 | GTExv8.EUR.Whole_Blood | 1.88E-09 | COG8 | DBP |
| -2.6825 | 7.31E-03 | GTExv8.EUR.Whole_Blood | 3.68E-02 | TMED6 | DBP |
| 4.5814 | 4.62E-06 | GTExv8.EUR.Whole_Blood | 7.40E-05 | TERF2 | DBP |
| -3.7495 | 1.77E-04 | GTExv8.EUR.Whole_Blood | 1.78E-03 | NFAT5 | DBP |
| -2.8382 | 4.54E-03 | GTExv8.EUR.Whole_Blood | 2.52E-02 | NQO1 | DBP |
| -2.8768 | 4.02E-03 | GTExv8.EUR.Whole_Blood | 2.29E-02 | WWP2 | DBP |
| -2.6397 | 8.30E-03 | GTExv8.EUR.Whole_Blood | 4.10E-02 | PHLPP2 | DBP |
| -3.4079 | 6.55E-04 | GTExv8.EUR.Whole_Blood | 5.30E-03 | PKD1L3 | DBP |
| -5.3422 | 9.18E-08 | GTExv8.EUR.Whole_Blood | 2.25E-06 | RP11-252K23.2 | DBP |
| -5.3043 | 1.13E-07 | GTExv8.EUR.Whole_Blood | 2.71E-06 | CFDP1 | DBP |
| -3.6240 | 2.90E-04 | GTExv8.EUR.Whole_Blood | 2.70E-03 | TMEM170A | DBP |
| 6.9190 | 4.55E-12 | GTExv8.EUR.Whole_Blood | 3.12E-10 | RP11-391L3.5 | DBP |
| -2.7256 | 6.42E-03 | GTExv8.EUR.Whole_Blood | 3.32E-02 | HSDL1 | DBP |
| -2.8482 | 4.40E-03 | GTExv8.EUR.Whole_Blood | 2.45E-02 | DNAAF1 | DBP |
| -3.0906 | 2.00E-03 | GTExv8.EUR.Whole_Blood | 1.31E-02 | ZDHHC7 | DBP |
| 4.8736 | 1.10E-06 | GTExv8.EUR.Whole_Blood | 2.10E-05 | MTHFSD | DBP |
| 3.7830 | 1.55E-04 | GTExv8.EUR.Whole_Blood | 1.61E-03 | MAP1LC3B | DBP |
| 4.0403 | 5.34E-05 | GTExv8.EUR.Whole_Blood | 6.41E-04 | FBXO31 | DBP |
| -3.9741 | 7.06E-05 | GTExv8.EUR.Whole_Blood | 8.24E-04 | ZC3H18 | DBP |
| -2.9101 | 3.61E-03 | GTExv8.EUR.Whole_Blood | 2.11E-02 | CYBA | DBP |
| 3.1760 | 1.49E-03 | GTExv8.EUR.Whole_Blood | 1.04E-02 | GALNS | DBP |
| -2.6545 | 7.94E-03 | GTExv8.EUR.Whole_Blood | 3.94E-02 | ACSF3 | DBP |
| -3.1098 | 1.87E-03 | GTExv8.EUR.Whole_Blood | 1.24E-02 | CTD-2555A7.3 | DBP |
| 3.4114 | 6.46E-04 | GTExv8.EUR.Whole_Blood | 5.24E-03 | AC137932.5 | DBP |
| -2.6183 | 8.84E-03 | GTExv8.EUR.Whole_Blood | 4.29E-02 | AC137932.6 | DBP |
| -2.6135 | 8.96E-03 | GTExv8.EUR.Whole_Blood | 4.33E-02 | SPG7 | DBP |
| -10.6273 | 2.23E-26 | GTExv8.EUR.Whole_Blood | 5.89E-24 | SPATA33 | DBP |
| -6.0952 | 1.09E-09 | GTExv8.EUR.Whole_Blood | 4.13E-08 | CDK10 | DBP |
| 7.2924 | 3.04E-13 | GTExv8.EUR.Whole_Blood | 2.74E-11 | SPATA2L | DBP |
| 4.2439 | 2.20E-05 | GTExv8.EUR.Whole_Blood | 3.01E-04 | VPS9D1-AS1 | DBP |
| 3.2632 | 1.10E-03 | GTExv8.EUR.Whole_Blood | 8.04E-03 | VPS9D1 | DBP |
| -4.2446 | 2.19E-05 | GTExv8.EUR.Whole_Blood | 3.00E-04 | FANCA | DBP |
| 2.8587 | 4.25E-03 | GTExv8.EUR.Whole_Blood | 2.38E-02 | SPIRE2 | DBP |
| 2.9478 | 3.20E-03 | GTExv8.EUR.Whole_Blood | 1.92E-02 | GAS8 | DBP |
| 4.0680 | 4.74E-05 | GTExv8.EUR.Whole_Blood | 5.78E-04 | URAHP | DBP |
| -3.7695 | 1.64E-04 | GTExv8.EUR.Whole_Blood | 1.68E-03 | FAM157C | DBP |
| -5.0333 | 4.82E-07 | GTExv8.EUR.Whole_Blood | 1.00E-05 | RP11-356C4.5 | DBP |
| -3.8886 | 1.01E-04 | GTExv8.EUR.Whole_Blood | 1.11E-03 | RP11-356C4.6 | DBP |
| 3.1189 | 1.82E-03 | GTExv8.EUR.Whole_Blood | 1.21E-02 | RFLNB | DBP |
| -3.5054 | 4.56E-04 | GTExv8.EUR.Whole_Blood | 3.94E-03 | VPS53 | DBP |
| 2.6777 | 7.41E-03 | GTExv8.EUR.Whole_Blood | 3.71E-02 | ABR | DBP |
| 5.3957 | 6.82E-08 | GTExv8.EUR.Whole_Blood | 1.70E-06 | SRR | DBP |
| 2.8859 | 3.90E-03 | GTExv8.EUR.Whole_Blood | 2.24E-02 | RP11-74E22.6 | DBP |
| 2.8744 | 4.05E-03 | GTExv8.EUR.Whole_Blood | 2.30E-02 | RP11-235E17.6 | DBP |
| -4.7479 | 2.06E-06 | GTExv8.EUR.Whole_Blood | 3.62E-05 | ALOX15 | DBP |
| 5.1562 | 2.52E-07 | GTExv8.EUR.Whole_Blood | 5.66E-06 | ARRB2 | DBP |
| -2.8550 | 4.30E-03 | GTExv8.EUR.Whole_Blood | 2.40E-02 | RNF167 | DBP |
| 2.6793 | 7.38E-03 | GTExv8.EUR.Whole_Blood | 3.70E-02 | NUP88 | DBP |
| -3.0920 | 1.99E-03 | GTExv8.EUR.Whole_Blood | 1.31E-02 | MIS12 | DBP |
| -8.7252 | 2.66E-18 | GTExv8.EUR.Whole_Blood | 4.13E-16 | ACADVL | DBP |
| -7.4100 | 1.26E-13 | GTExv8.EUR.Whole_Blood | 1.16E-11 | DVL2 | DBP |
| 7.5224 | 5.38E-14 | GTExv8.EUR.Whole_Blood | 5.20E-12 | CTDNEP1 | DBP |
| -8.2233 | 1.98E-16 | GTExv8.EUR.Whole_Blood | 2.57E-14 | ELP5 | DBP |
| -3.6555 | 2.57E-04 | GTExv8.EUR.Whole_Blood | 2.46E-03 | GPS2 | DBP |
| -3.8623 | 1.12E-04 | GTExv8.EUR.Whole_Blood | 1.21E-03 | TNK1 | DBP |
| 4.9492 | 7.45E-07 | GTExv8.EUR.Whole_Blood | 1.49E-05 | ZBTB4 | DBP |
| 6.8494 | 7.42E-12 | GTExv8.EUR.Whole_Blood | 4.90E-10 | TNFSF12 | DBP |
| -4.9241 | 8.48E-07 | GTExv8.EUR.Whole_Blood | 1.68E-05 | SAT2 | DBP |
| -8.2868 | 1.16E-16 | GTExv8.EUR.Whole_Blood | 1.61E-14 | CHD3 | DBP |
| 4.1036 | 4.07E-05 | GTExv8.EUR.Whole_Blood | 5.03E-04 | VAMP2 | DBP |
| 3.5461 | 3.91E-04 | GTExv8.EUR.Whole_Blood | 3.46E-03 | CTC1 | DBP |
| 2.9463 | 3.22E-03 | GTExv8.EUR.Whole_Blood | 1.93E-02 | PIK3R6 | DBP |
| 3.5226 | 4.27E-04 | GTExv8.EUR.Whole_Blood | 3.73E-03 | CDRT15P1 | DBP |
| 3.9555 | 7.64E-05 | GTExv8.EUR.Whole_Blood | 8.79E-04 | CDRT4 | DBP |
| 3.7051 | 2.11E-04 | GTExv8.EUR.Whole_Blood | 2.07E-03 | TVP23C | DBP |
| 3.0801 | 2.07E-03 | GTExv8.EUR.Whole_Blood | 1.35E-02 | ADORA2B | DBP |
| -4.4743 | 7.67E-06 | GTExv8.EUR.Whole_Blood | 1.16E-04 | TTC19 | DBP |
| -5.1696 | 2.35E-07 | GTExv8.EUR.Whole_Blood | 5.29E-06 | CTC-529I10.1 | DBP |
| 5.2795 | 1.30E-07 | GTExv8.EUR.Whole_Blood | 3.07E-06 | CTC-529I10.2 | DBP |
| 6.0781 | 1.22E-09 | GTExv8.EUR.Whole_Blood | 4.58E-08 | NCOR1 | DBP |
| 5.5776 | 2.44E-08 | GTExv8.EUR.Whole_Blood | 6.81E-07 | PIGL | DBP |
| 6.6937 | 2.18E-11 | GTExv8.EUR.Whole_Blood | 1.30E-09 | CENPV | DBP |
| -3.4054 | 6.61E-04 | GTExv8.EUR.Whole_Blood | 5.33E-03 | LRRC75A | DBP |
| -3.2650 | 1.09E-03 | GTExv8.EUR.Whole_Blood | 7.99E-03 | MPRIP | DBP |
| 2.6768 | 7.43E-03 | GTExv8.EUR.Whole_Blood | 3.71E-02 | FLCN | DBP |
| 3.6466 | 2.66E-04 | GTExv8.EUR.Whole_Blood | 2.53E-03 | COPS3 | DBP |
| -3.6722 | 2.40E-04 | GTExv8.EUR.Whole_Blood | 2.32E-03 | PEMT | DBP |
| -3.7143 | 2.04E-04 | GTExv8.EUR.Whole_Blood | 2.01E-03 | TOM1L2 | DBP |
| 2.7410 | 6.13E-03 | GTExv8.EUR.Whole_Blood | 3.20E-02 | DRG2 | DBP |
| -3.6374 | 2.75E-04 | GTExv8.EUR.Whole_Blood | 2.59E-03 | LLGL1 | DBP |
| 4.8245 | 1.40E-06 | GTExv8.EUR.Whole_Blood | 2.57E-05 | FLII | DBP |
| -4.1570 | 3.23E-05 | GTExv8.EUR.Whole_Blood | 4.16E-04 | TOP3A | DBP |
| 5.2255 | 1.74E-07 | GTExv8.EUR.Whole_Blood | 4.02E-06 | SMCR8 | DBP |
| 6.2701 | 3.61E-10 | GTExv8.EUR.Whole_Blood | 1.56E-08 | SHMT1 | DBP |
| 6.9032 | 5.08E-12 | GTExv8.EUR.Whole_Blood | 3.41E-10 | SLC5A10 | DBP |
| -6.4679 | 9.94E-11 | GTExv8.EUR.Whole_Blood | 5.05E-09 | CTC-457L16.1 | DBP |
| 7.2026 | 5.91E-13 | GTExv8.EUR.Whole_Blood | 4.88E-11 | GRAP | DBP |
| 7.0836 | 1.40E-12 | GTExv8.EUR.Whole_Blood | 1.11E-10 | CTC-457L16.2 | DBP |
| -5.8883 | 3.90E-09 | GTExv8.EUR.Whole_Blood | 1.34E-07 | RNF112 | DBP |
| -3.2736 | 1.06E-03 | GTExv8.EUR.Whole_Blood | 7.84E-03 | ULK2 | DBP |
| 2.7291 | 6.35E-03 | GTExv8.EUR.Whole_Blood | 3.29E-02 | RP11-209D14.4 | DBP |
| -4.7177 | 2.38E-06 | GTExv8.EUR.Whole_Blood | 4.10E-05 | LGALS9B | DBP |
| 2.6251 | 8.66E-03 | GTExv8.EUR.Whole_Blood | 4.22E-02 | LYRM9 | DBP |
| -3.6222 | 2.92E-04 | GTExv8.EUR.Whole_Blood | 2.71E-03 | TMEM97 | DBP |
| -3.7742 | 1.61E-04 | GTExv8.EUR.Whole_Blood | 1.65E-03 | POLDIP2 | DBP |
| -4.0216 | 5.78E-05 | GTExv8.EUR.Whole_Blood | 6.90E-04 | TMEM199 | DBP |
| -3.6320 | 2.81E-04 | GTExv8.EUR.Whole_Blood | 2.64E-03 | SARM1 | DBP |
| -3.6383 | 2.74E-04 | GTExv8.EUR.Whole_Blood | 2.58E-03 | PROCA1 | DBP |
| -3.7348 | 1.88E-04 | GTExv8.EUR.Whole_Blood | 1.87E-03 | RAB34 | DBP |
| 2.8552 | 4.30E-03 | GTExv8.EUR.Whole_Blood | 2.40E-02 | GIT1 | DBP |
| 3.1772 | 1.49E-03 | GTExv8.EUR.Whole_Blood | 1.04E-02 | CORO6 | DBP |
| -2.9774 | 2.91E-03 | GTExv8.EUR.Whole_Blood | 1.77E-02 | SSH2 | DBP |
| 3.2750 | 1.06E-03 | GTExv8.EUR.Whole_Blood | 7.84E-03 | SUZ12P1 | DBP |
| 3.2397 | 1.20E-03 | GTExv8.EUR.Whole_Blood | 8.62E-03 | CRLF3 | DBP |
| -3.4186 | 6.29E-04 | GTExv8.EUR.Whole_Blood | 5.12E-03 | ZNHIT3 | DBP |
| 3.5107 | 4.47E-04 | GTExv8.EUR.Whole_Blood | 3.88E-03 | MYO19 | DBP |
| -3.4606 | 5.39E-04 | GTExv8.EUR.Whole_Blood | 4.54E-03 | GGNBP2 | DBP |
| 3.7277 | 1.93E-04 | GTExv8.EUR.Whole_Blood | 1.91E-03 | DHRS11 | DBP |
| -3.2080 | 1.34E-03 | GTExv8.EUR.Whole_Blood | 9.48E-03 | STARD3 | DBP |
| -2.7577 | 5.82E-03 | GTExv8.EUR.Whole_Blood | 3.07E-02 | CASC3 | DBP |
| -3.4460 | 5.69E-04 | GTExv8.EUR.Whole_Blood | 4.73E-03 | RP11-458J1.1 | DBP |
| -2.6350 | 8.41E-03 | GTExv8.EUR.Whole_Blood | 4.13E-02 | JUP | DBP |
| -3.0441 | 2.33E-03 | GTExv8.EUR.Whole_Blood | 1.48E-02 | HSD17B1 | DBP |
| -2.8650 | 4.17E-03 | GTExv8.EUR.Whole_Blood | 2.36E-02 | CNTNAP1 | DBP |
| 3.8630 | 1.12E-04 | GTExv8.EUR.Whole_Blood | 1.21E-03 | ASB16 | DBP |
| 4.1876 | 2.82E-05 | GTExv8.EUR.Whole_Blood | 3.72E-04 | ASB16-AS1 | DBP |
| -8.2747 | 1.29E-16 | GTExv8.EUR.Whole_Blood | 1.76E-14 | DCAKD | DBP |
| 2.5556 | 1.06E-02 | GTExv8.EUR.Whole_Blood | 4.95E-02 | ACBD4 | DBP |
| 3.6400 | 2.73E-04 | GTExv8.EUR.Whole_Blood | 2.58E-03 | SPATA32 | DBP |
| 5.4866 | 4.10E-08 | GTExv8.EUR.Whole_Blood | 1.07E-06 | PLEKHM1 | DBP |
| 6.3886 | 1.67E-10 | GTExv8.EUR.Whole_Blood | 7.83E-09 | LRRC37A4P | DBP |
| -6.4264 | 1.31E-10 | GTExv8.EUR.Whole_Blood | 6.33E-09 | DND1P1 | DBP |
| -6.4348 | 1.24E-10 | GTExv8.EUR.Whole_Blood | 6.10E-09 | RP11-707O23.1 | DBP |
| -6.4264 | 1.31E-10 | GTExv8.EUR.Whole_Blood | 6.33E-09 | MAPK8IP1P2 | DBP |
| -6.3901 | 1.66E-10 | GTExv8.EUR.Whole_Blood | 7.83E-09 | LINC02210 | DBP |
| -6.4265 | 1.31E-10 | GTExv8.EUR.Whole_Blood | 6.33E-09 | KANSL1-AS1 | DBP |
| -5.9214 | 3.19E-09 | GTExv8.EUR.Whole_Blood | 1.11E-07 | MAPK8IP1P1 | DBP |
| -5.7685 | 8.00E-09 | GTExv8.EUR.Whole_Blood | 2.46E-07 | RP11-259G18.3 | DBP |
| -2.6384 | 8.33E-03 | GTExv8.EUR.Whole_Blood | 4.10E-02 | LRRC37A | DBP |
| -5.9539 | 2.62E-09 | GTExv8.EUR.Whole_Blood | 9.30E-08 | LRRC37A2 | DBP |
| -3.4913 | 4.81E-04 | GTExv8.EUR.Whole_Blood | 4.11E-03 | ARL17A | DBP |
| 5.9377 | 2.89E-09 | GTExv8.EUR.Whole_Blood | 1.02E-07 | GOSR2 | DBP |
| 4.1666 | 3.09E-05 | GTExv8.EUR.Whole_Blood | 3.99E-04 | LRRC37A17P | DBP |
| -3.6825 | 2.31E-04 | GTExv8.EUR.Whole_Blood | 2.25E-03 | SCRN2 | DBP |
| -3.3311 | 8.65E-04 | GTExv8.EUR.Whole_Blood | 6.60E-03 | PNPO | DBP |
| 5.5659 | 2.61E-08 | GTExv8.EUR.Whole_Blood | 7.21E-07 | CDK5RAP3 | DBP |
| -2.7256 | 6.42E-03 | GTExv8.EUR.Whole_Blood | 3.32E-02 | HOXB4 | DBP |
| -5.4365 | 5.43E-08 | GTExv8.EUR.Whole_Blood | 1.39E-06 | HOXB7 | DBP |
| -3.8234 | 1.32E-04 | GTExv8.EUR.Whole_Blood | 1.41E-03 | CALCOCO2 | DBP |
| -6.1885 | 6.07E-10 | GTExv8.EUR.Whole_Blood | 2.54E-08 | ATP5G1 | DBP |
| -6.2578 | 3.90E-10 | GTExv8.EUR.Whole_Blood | 1.67E-08 | UBE2Z | DBP |
| 2.9169 | 3.54E-03 | GTExv8.EUR.Whole_Blood | 2.07E-02 | GNGT2 | DBP |
| -2.6203 | 8.78E-03 | GTExv8.EUR.Whole_Blood | 4.27E-02 | RP11-81K2.1 | DBP |
| 3.9674 | 7.27E-05 | GTExv8.EUR.Whole_Blood | 8.42E-04 | SPATA20 | DBP |
| 3.3739 | 7.41E-04 | GTExv8.EUR.Whole_Blood | 5.85E-03 | ABCC3 | DBP |
| -5.0549 | 4.31E-07 | GTExv8.EUR.Whole_Blood | 9.06E-06 | TSPOAP1-AS1 | DBP |
| 6.5444 | 5.97E-11 | GTExv8.EUR.Whole_Blood | 3.15E-09 | TSPOAP1 | DBP |
| 5.3696 | 7.89E-08 | GTExv8.EUR.Whole_Blood | 1.95E-06 | TEX14 | DBP |
| 2.6575 | 7.87E-03 | GTExv8.EUR.Whole_Blood | 3.92E-02 | AC099850.1 | DBP |
| 5.7037 | 1.17E-08 | GTExv8.EUR.Whole_Blood | 3.49E-07 | SMG8 | DBP |
| 2.9266 | 3.43E-03 | GTExv8.EUR.Whole_Blood | 2.02E-02 | RPS6KB1 | DBP |
| -2.8326 | 4.62E-03 | GTExv8.EUR.Whole_Blood | 2.55E-02 | RP11-51F16.1 | DBP |
| -6.4903 | 8.56E-11 | GTExv8.EUR.Whole_Blood | 4.40E-09 | TEX2 | DBP |
| 5.2899 | 1.22E-07 | GTExv8.EUR.Whole_Blood | 2.90E-06 | PECAM1 | DBP |
| -3.5309 | 4.14E-04 | GTExv8.EUR.Whole_Blood | 3.64E-03 | CEP95 | DBP |
| 3.5309 | 4.14E-04 | GTExv8.EUR.Whole_Blood | 3.64E-03 | DDX5 | DBP |
| 3.3527 | 8.00E-04 | GTExv8.EUR.Whole_Blood | 6.23E-03 | RP11-147L13.8 | DBP |
| 3.5965 | 3.23E-04 | GTExv8.EUR.Whole_Blood | 2.96E-03 | PRKAR1A | DBP |
| 2.7711 | 5.59E-03 | GTExv8.EUR.Whole_Blood | 2.98E-02 | FAM20A | DBP |
| 2.7281 | 6.37E-03 | GTExv8.EUR.Whole_Blood | 3.30E-02 | FAM104A | DBP |
| 5.0603 | 4.19E-07 | GTExv8.EUR.Whole_Blood | 8.83E-06 | GALK1 | DBP |
| 4.8767 | 1.08E-06 | GTExv8.EUR.Whole_Blood | 2.06E-05 | UNK | DBP |
| 6.8013 | 1.04E-11 | GTExv8.EUR.Whole_Blood | 6.65E-10 | UNC13D | DBP |
| -6.4920 | 8.47E-11 | GTExv8.EUR.Whole_Blood | 4.39E-09 | RP11-552F3.9 | DBP |
| 3.3795 | 7.26E-04 | GTExv8.EUR.Whole_Blood | 5.75E-03 | RP11-552F3.10 | DBP |
| 5.0687 | 4.01E-07 | GTExv8.EUR.Whole_Blood | 8.49E-06 | MRPL38 | DBP |
| 4.0193 | 5.84E-05 | GTExv8.EUR.Whole_Blood | 6.96E-04 | CDK3 | DBP |
| -4.1906 | 2.78E-05 | GTExv8.EUR.Whole_Blood | 3.68E-04 | RNF157 | DBP |
| -2.6999 | 6.94E-03 | GTExv8.EUR.Whole_Blood | 3.53E-02 | SPHK1 | DBP |
| 4.0888 | 4.34E-05 | GTExv8.EUR.Whole_Blood | 5.34E-04 | ST6GALNAC1 | DBP |
| -3.8028 | 1.43E-04 | GTExv8.EUR.Whole_Blood | 1.49E-03 | MXRA7 | DBP |
| -4.1035 | 4.07E-05 | GTExv8.EUR.Whole_Blood | 5.03E-04 | JMJD6 | DBP |
| 2.9082 | 3.63E-03 | GTExv8.EUR.Whole_Blood | 2.12E-02 | SEC14L1 | DBP |
| 2.7536 | 5.89E-03 | GTExv8.EUR.Whole_Blood | 3.09E-02 | TMC8 | DBP |
| -4.9028 | 9.45E-07 | GTExv8.EUR.Whole_Blood | 1.85E-05 | RP11-806H10.4 | DBP |
| 4.0835 | 4.44E-05 | GTExv8.EUR.Whole_Blood | 5.45E-04 | DNAH17 | DBP |
| -3.9979 | 6.39E-05 | GTExv8.EUR.Whole_Blood | 7.55E-04 | TIMP2 | DBP |
| 5.7540 | 8.71E-09 | GTExv8.EUR.Whole_Blood | 2.64E-07 | RP11-1055B8.4 | DBP |
| 3.0804 | 2.07E-03 | GTExv8.EUR.Whole_Blood | 1.35E-02 | PDE6G | DBP |
| 3.1207 | 1.80E-03 | GTExv8.EUR.Whole_Blood | 1.20E-02 | OXLD1 | DBP |
| 2.8657 | 4.16E-03 | GTExv8.EUR.Whole_Blood | 2.35E-02 | ARL16 | DBP |
| 2.7681 | 5.64E-03 | GTExv8.EUR.Whole_Blood | 3.00E-02 | SLC25A10 | DBP |
| -2.6289 | 8.57E-03 | GTExv8.EUR.Whole_Blood | 4.19E-02 | WDR45B | DBP |
| -2.7574 | 5.83E-03 | GTExv8.EUR.Whole_Blood | 3.07E-02 | COLEC12 | DBP |
| 3.6844 | 2.29E-04 | GTExv8.EUR.Whole_Blood | 2.23E-03 | ENOSF1 | DBP |
| 5.1147 | 3.14E-07 | GTExv8.EUR.Whole_Blood | 6.84E-06 | RP11-672L10.6 | DBP |
| -3.2608 | 1.11E-03 | GTExv8.EUR.Whole_Blood | 8.10E-03 | RBBP8 | DBP |
| -3.3635 | 7.70E-04 | GTExv8.EUR.Whole_Blood | 6.06E-03 | C18orf8 | DBP |
| -2.6480 | 8.10E-03 | GTExv8.EUR.Whole_Blood | 4.01E-02 | TTC39C-AS1 | DBP |
| 4.4604 | 8.18E-06 | GTExv8.EUR.Whole_Blood | 1.23E-04 | CDH2 | DBP |
| 3.6293 | 2.84E-04 | GTExv8.EUR.Whole_Blood | 2.66E-03 | TPGS2 | DBP |
| 3.0320 | 2.43E-03 | GTExv8.EUR.Whole_Blood | 1.53E-02 | KIAA1328 | DBP |
| -2.8043 | 5.04E-03 | GTExv8.EUR.Whole_Blood | 2.75E-02 | RP11-850A17.1 | DBP |
| -6.5999 | 4.11E-11 | GTExv8.EUR.Whole_Blood | 2.33E-09 | POLI | DBP |
| -2.9846 | 2.84E-03 | GTExv8.EUR.Whole_Blood | 1.74E-02 | ZNF532 | DBP |
| -2.9315 | 3.37E-03 | GTExv8.EUR.Whole_Blood | 2.00E-02 | HSBP1L1 | DBP |
| 4.1712 | 3.03E-05 | GTExv8.EUR.Whole_Blood | 3.92E-04 | STK11 | DBP |
| 5.0222 | 5.11E-07 | GTExv8.EUR.Whole_Blood | 1.05E-05 | DOT1L | DBP |
| 5.2459 | 1.56E-07 | GTExv8.EUR.Whole_Blood | 3.65E-06 | AC004490.1 | DBP |
| 3.2708 | 1.07E-03 | GTExv8.EUR.Whole_Blood | 7.89E-03 | PLEKHJ1 | DBP |
| -5.0351 | 4.78E-07 | GTExv8.EUR.Whole_Blood | 9.94E-06 | SF3A2 | DBP |
| 2.6818 | 7.32E-03 | GTExv8.EUR.Whole_Blood | 3.68E-02 | LSM7 | DBP |
| 3.0139 | 2.58E-03 | GTExv8.EUR.Whole_Blood | 1.61E-02 | ARRDC5 | DBP |
| 3.2420 | 1.19E-03 | GTExv8.EUR.Whole_Blood | 8.59E-03 | CTC-312O10.2 | DBP |
| -5.1387 | 2.77E-07 | GTExv8.EUR.Whole_Blood | 6.10E-06 | CTB-133G6.1 | DBP |
| -5.1387 | 2.77E-07 | GTExv8.EUR.Whole_Blood | 6.10E-06 | ARHGEF18 | DBP |
| 3.0807 | 2.06E-03 | GTExv8.EUR.Whole_Blood | 1.35E-02 | CLEC4G | DBP |
| 2.6983 | 6.97E-03 | GTExv8.EUR.Whole_Blood | 3.54E-02 | CERS4 | DBP |
| 6.1786 | 6.47E-10 | GTExv8.EUR.Whole_Blood | 2.67E-08 | RPS28 | DBP |
| 3.1066 | 1.89E-03 | GTExv8.EUR.Whole_Blood | 1.25E-02 | RAB11B | DBP |
| 3.5499 | 3.85E-04 | GTExv8.EUR.Whole_Blood | 3.42E-03 | MARCH2 | DBP |
| -4.3968 | 1.10E-05 | GTExv8.EUR.Whole_Blood | 1.60E-04 | KANK2 | DBP |
| -5.5631 | 2.65E-08 | GTExv8.EUR.Whole_Blood | 7.29E-07 | CTC-510F12.2 | DBP |
| 2.8994 | 3.74E-03 | GTExv8.EUR.Whole_Blood | 2.17E-02 | TSPAN16 | DBP |
| 2.7802 | 5.43E-03 | GTExv8.EUR.Whole_Blood | 2.92E-02 | EPOR | DBP |
| 6.1681 | 6.91E-10 | GTExv8.EUR.Whole_Blood | 2.82E-08 | RGL3 | DBP |
| -3.9299 | 8.50E-05 | GTExv8.EUR.Whole_Blood | 9.59E-04 | PRKCSH | DBP |
| -3.6921 | 2.22E-04 | GTExv8.EUR.Whole_Blood | 2.17E-03 | ZNF443 | DBP |
| -2.7595 | 5.79E-03 | GTExv8.EUR.Whole_Blood | 3.06E-02 | ZNF564 | DBP |
| -4.2573 | 2.07E-05 | GTExv8.EUR.Whole_Blood | 2.86E-04 | PRDX2 | DBP |
| -3.9174 | 8.95E-05 | GTExv8.EUR.Whole_Blood | 1.01E-03 | LINC01835 | DBP |
| -3.9498 | 7.82E-05 | GTExv8.EUR.Whole_Blood | 8.92E-04 | EPS15L1 | DBP |
| 2.9001 | 3.73E-03 | GTExv8.EUR.Whole_Blood | 2.17E-02 | C19orf44 | DBP |
| -8.8387 | 9.69E-19 | GTExv8.EUR.Whole_Blood | 1.54E-16 | HAUS8 | DBP |
| -5.5161 | 3.47E-08 | GTExv8.EUR.Whole_Blood | 9.26E-07 | MYO9B | DBP |
| 6.2654 | 3.72E-10 | GTExv8.EUR.Whole_Blood | 1.60E-08 | ANKLE1 | DBP |
| -3.8648 | 1.11E-04 | GTExv8.EUR.Whole_Blood | 1.21E-03 | GTPBP3 | DBP |
| 2.5940 | 9.49E-03 | GTExv8.EUR.Whole_Blood | 4.53E-02 | INSL3 | DBP |
| -4.0863 | 4.38E-05 | GTExv8.EUR.Whole_Blood | 5.38E-04 | SSBP4 | DBP |
| -6.4546 | 1.08E-10 | GTExv8.EUR.Whole_Blood | 5.42E-09 | MAU2 | DBP |
| 3.5640 | 3.65E-04 | GTExv8.EUR.Whole_Blood | 3.27E-03 | YJEFN3 | DBP |
| 4.0465 | 5.20E-05 | GTExv8.EUR.Whole_Blood | 6.26E-04 | ATP13A1 | DBP |
| 3.8923 | 9.93E-05 | GTExv8.EUR.Whole_Blood | 1.10E-03 | ZNF101 | DBP |
| 3.0878 | 2.02E-03 | GTExv8.EUR.Whole_Blood | 1.32E-02 | ZNF506 | DBP |
| -4.0081 | 6.12E-05 | GTExv8.EUR.Whole_Blood | 7.27E-04 | ZNF430 | DBP |
| -4.4158 | 1.01E-05 | GTExv8.EUR.Whole_Blood | 1.48E-04 | VN1R83P | DBP |
| -4.0552 | 5.01E-05 | GTExv8.EUR.Whole_Blood | 6.06E-04 | BNIP3P25 | DBP |
| -4.1750 | 2.98E-05 | GTExv8.EUR.Whole_Blood | 3.87E-04 | ZNF708 | DBP |
| -3.7686 | 1.64E-04 | GTExv8.EUR.Whole_Blood | 1.68E-03 | ZNF738 | DBP |
| -3.7839 | 1.54E-04 | GTExv8.EUR.Whole_Blood | 1.60E-03 | ZNF493 | DBP |
| -4.4081 | 1.04E-05 | GTExv8.EUR.Whole_Blood | 1.52E-04 | CTD-2561J22.5 | DBP |
| -3.8296 | 1.28E-04 | GTExv8.EUR.Whole_Blood | 1.37E-03 | LINC00664 | DBP |
| -3.7640 | 1.67E-04 | GTExv8.EUR.Whole_Blood | 1.70E-03 | ZNF429 | DBP |
| -3.8632 | 1.12E-04 | GTExv8.EUR.Whole_Blood | 1.21E-03 | RP11-678G14.2 | DBP |
| -4.2003 | 2.67E-05 | GTExv8.EUR.Whole_Blood | 3.56E-04 | RP11-678G14.3 | DBP |
| -4.2152 | 2.50E-05 | GTExv8.EUR.Whole_Blood | 3.35E-04 | RP11-678G14.4 | DBP |
| -4.2985 | 1.72E-05 | GTExv8.EUR.Whole_Blood | 2.41E-04 | MTDHP3 | DBP |
| -6.0192 | 1.75E-09 | GTExv8.EUR.Whole_Blood | 6.42E-08 | MTDHP4 | DBP |
| -6.6565 | 2.80E-11 | GTExv8.EUR.Whole_Blood | 1.62E-09 | RP11-420K14.6 | DBP |
| -7.2793 | 3.36E-13 | GTExv8.EUR.Whole_Blood | 2.99E-11 | ZNF100 | DBP |
| -4.0452 | 5.23E-05 | GTExv8.EUR.Whole_Blood | 6.29E-04 | ZNF43 | DBP |
| 3.2106 | 1.32E-03 | GTExv8.EUR.Whole_Blood | 9.36E-03 | ZNF257 | DBP |
| 2.9219 | 3.48E-03 | GTExv8.EUR.Whole_Blood | 2.05E-02 | LINC00662 | DBP |
| 3.8200 | 1.33E-04 | GTExv8.EUR.Whole_Blood | 1.41E-03 | CTC-459F4.9 | DBP |
| 3.0758 | 2.10E-03 | GTExv8.EUR.Whole_Blood | 1.37E-02 | CTC-459F4.6 | DBP |
| -3.0112 | 2.60E-03 | GTExv8.EUR.Whole_Blood | 1.62E-02 | TSHZ3 | DBP |
| -2.6339 | 8.44E-03 | GTExv8.EUR.Whole_Blood | 4.14E-02 | ZNF585B | DBP |
| -4.2666 | 1.98E-05 | GTExv8.EUR.Whole_Blood | 2.75E-04 | ZNF793 | DBP |
| 3.5277 | 4.19E-04 | GTExv8.EUR.Whole_Blood | 3.67E-03 | ZNF781 | DBP |
| -3.8413 | 1.22E-04 | GTExv8.EUR.Whole_Blood | 1.31E-03 | ACTN4 | DBP |
| -2.9792 | 2.89E-03 | GTExv8.EUR.Whole_Blood | 1.76E-02 | CAPN12 | DBP |
| -5.7875 | 7.15E-09 | GTExv8.EUR.Whole_Blood | 2.23E-07 | MAP3K10 | DBP |
| 4.4186 | 9.93E-06 | GTExv8.EUR.Whole_Blood | 1.47E-04 | CTC-425O23.5 | DBP |
| -4.0124 | 6.01E-05 | GTExv8.EUR.Whole_Blood | 7.15E-04 | SERTAD3 | DBP |
| -3.1215 | 1.80E-03 | GTExv8.EUR.Whole_Blood | 1.20E-02 | EGLN2 | DBP |
| -3.3519 | 8.03E-04 | GTExv8.EUR.Whole_Blood | 6.25E-03 | CTC-490E21.11 | DBP |
| 5.0150 | 5.30E-07 | GTExv8.EUR.Whole_Blood | 1.09E-05 | HNRNPUL1 | DBP |
| -2.8317 | 4.63E-03 | GTExv8.EUR.Whole_Blood | 2.55E-02 | TMEM91 | DBP |
| -3.4744 | 5.12E-04 | GTExv8.EUR.Whole_Blood | 4.35E-03 | ATP5SL | DBP |
| 3.4606 | 5.39E-04 | GTExv8.EUR.Whole_Blood | 4.54E-03 | SMG9 | DBP |
| 2.8842 | 3.92E-03 | GTExv8.EUR.Whole_Blood | 2.24E-02 | ZNF283 | DBP |
| 2.9371 | 3.31E-03 | GTExv8.EUR.Whole_Blood | 1.97E-02 | PPM1N | DBP |
| 2.6469 | 8.12E-03 | GTExv8.EUR.Whole_Blood | 4.02E-02 | CCDC61 | DBP |
| 2.6180 | 8.85E-03 | GTExv8.EUR.Whole_Blood | 4.29E-02 | AP2S1 | DBP |
| 3.0424 | 2.35E-03 | GTExv8.EUR.Whole_Blood | 1.49E-02 | SAE1 | DBP |
| -3.0213 | 2.52E-03 | GTExv8.EUR.Whole_Blood | 1.58E-02 | CABP5 | DBP |
| 6.9844 | 2.86E-12 | GTExv8.EUR.Whole_Blood | 2.08E-10 | NTN5 | DBP |
| 4.1530 | 3.28E-05 | GTExv8.EUR.Whole_Blood | 4.21E-04 | PRMT1 | DBP |
| -3.4720 | 5.17E-04 | GTExv8.EUR.Whole_Blood | 4.38E-03 | FUZ | DBP |
| -3.2672 | 1.09E-03 | GTExv8.EUR.Whole_Blood | 7.99E-03 | PNKP | DBP |
| -4.2463 | 2.17E-05 | GTExv8.EUR.Whole_Blood | 2.97E-04 | TBC1D17 | DBP |
| -3.0139 | 2.58E-03 | GTExv8.EUR.Whole_Blood | 1.61E-02 | ZNF160 | DBP |
| 2.6039 | 9.22E-03 | GTExv8.EUR.Whole_Blood | 4.43E-02 | LILRB3 | DBP |
| -2.8037 | 5.05E-03 | GTExv8.EUR.Whole_Blood | 2.75E-02 | ZNF582-AS1 | DBP |
| -3.1930 | 1.41E-03 | GTExv8.EUR.Whole_Blood | 9.89E-03 | ZNF304 | DBP |
| -3.1351 | 1.72E-03 | GTExv8.EUR.Whole_Blood | 1.16E-02 | ZNF749 | DBP |
| 3.1351 | 1.72E-03 | GTExv8.EUR.Whole_Blood | 1.16E-02 | AC004076.5 | DBP |
| -2.6432 | 8.21E-03 | GTExv8.EUR.Whole_Blood | 4.06E-02 | CTD-2583A14.11 | DBP |
| -2.7543 | 5.88E-03 | GTExv8.EUR.Whole_Blood | 3.09E-02 | ZNF417 | DBP |
| -2.8143 | 4.89E-03 | GTExv8.EUR.Whole_Blood | 2.67E-02 | ZNF606 | DBP |
| 3.4279 | 6.08E-04 | GTExv8.EUR.Whole_Blood | 4.98E-03 | CENPB | DBP |
| 4.2830 | 1.84E-05 | GTExv8.EUR.Whole_Blood | 2.57E-04 | MKKS | DBP |
| 2.7190 | 6.55E-03 | GTExv8.EUR.Whole_Blood | 3.37E-02 | SLX4IP | DBP |
| -6.1139 | 9.72E-10 | GTExv8.EUR.Whole_Blood | 3.78E-08 | BTBD3 | DBP |
| 3.1618 | 1.57E-03 | GTExv8.EUR.Whole_Blood | 1.08E-02 | SEC23B | DBP |
| 2.9983 | 2.72E-03 | GTExv8.EUR.Whole_Blood | 1.68E-02 | LINC00493 | DBP |
| 2.8013 | 5.09E-03 | GTExv8.EUR.Whole_Blood | 2.77E-02 | CST7 | DBP |
| 2.9235 | 3.46E-03 | GTExv8.EUR.Whole_Blood | 2.04E-02 | APMAP | DBP |
| 3.1217 | 1.80E-03 | GTExv8.EUR.Whole_Blood | 1.20E-02 | RP5-965G21.4 | DBP |
| -2.6263 | 8.63E-03 | GTExv8.EUR.Whole_Blood | 4.22E-02 | PYGB | DBP |
| -7.0656 | 1.60E-12 | GTExv8.EUR.Whole_Blood | 1.23E-10 | HM13 | DBP |
| -7.0712 | 1.54E-12 | GTExv8.EUR.Whole_Blood | 1.20E-10 | MCTS2P | DBP |
| -3.2473 | 1.17E-03 | GTExv8.EUR.Whole_Blood | 8.46E-03 | SNTA1 | DBP |
| 2.5657 | 1.03E-02 | GTExv8.EUR.Whole_Blood | 4.83E-02 | GGT7 | DBP |
| -3.2660 | 1.09E-03 | GTExv8.EUR.Whole_Blood | 7.99E-03 | MYH7B | DBP |
| 3.7554 | 1.73E-04 | GTExv8.EUR.Whole_Blood | 1.75E-03 | TRPC4AP | DBP |
| -2.6517 | 8.01E-03 | GTExv8.EUR.Whole_Blood | 3.97E-02 | FER1L4 | DBP |
| 3.4491 | 5.62E-04 | GTExv8.EUR.Whole_Blood | 4.68E-03 | OSER1-AS1 | DBP |
| 2.6387 | 8.32E-03 | GTExv8.EUR.Whole_Blood | 4.10E-02 | YWHAB | DBP |
| 2.5642 | 1.03E-02 | GTExv8.EUR.Whole_Blood | 4.83E-02 | SNX21 | DBP |
| 6.4667 | 1.00E-10 | GTExv8.EUR.Whole_Blood | 5.05E-09 | PREX1 | DBP |
| 3.8746 | 1.07E-04 | GTExv8.EUR.Whole_Blood | 1.17E-03 | ZNFX1 | DBP |
| 2.9340 | 3.35E-03 | GTExv8.EUR.Whole_Blood | 1.99E-02 | ZFAS1 | DBP |
| 4.9219 | 8.57E-07 | GTExv8.EUR.Whole_Blood | 1.69E-05 | RP4-791K14.2 | DBP |
| -2.7476 | 6.00E-03 | GTExv8.EUR.Whole_Blood | 3.14E-02 | UBE2V1 | DBP |
| 2.7221 | 6.49E-03 | GTExv8.EUR.Whole_Blood | 3.35E-02 | SMIM25 | DBP |
| 6.5008 | 7.99E-11 | GTExv8.EUR.Whole_Blood | 4.17E-09 | NELFCD | DBP |
| -3.3027 | 9.58E-04 | GTExv8.EUR.Whole_Blood | 7.20E-03 | ZNF831 | DBP |
| -3.3027 | 9.58E-04 | GTExv8.EUR.Whole_Blood | 7.20E-03 | EDN3 | DBP |
| -2.8764 | 4.02E-03 | GTExv8.EUR.Whole_Blood | 2.29E-02 | DIDO1 | DBP |
| 3.2255 | 1.26E-03 | GTExv8.EUR.Whole_Blood | 9.00E-03 | SLC17A9 | DBP |
| -3.1685 | 1.53E-03 | GTExv8.EUR.Whole_Blood | 1.06E-02 | GMEB2 | DBP |
| 5.8003 | 6.62E-09 | GTExv8.EUR.Whole_Blood | 2.09E-07 | STMN3 | DBP |
| -6.5841 | 4.58E-11 | GTExv8.EUR.Whole_Blood | 2.52E-09 | ARFRP1 | DBP |
| -3.6693 | 2.43E-04 | GTExv8.EUR.Whole_Blood | 2.35E-03 | LIME1 | DBP |
| -5.6151 | 1.96E-08 | GTExv8.EUR.Whole_Blood | 5.61E-07 | PRPF6 | DBP |
| -5.2383 | 1.62E-07 | GTExv8.EUR.Whole_Blood | 3.78E-06 | TCEA2 | DBP |
| 6.3402 | 2.29E-10 | GTExv8.EUR.Whole_Blood | 1.03E-08 | OPRL1 | DBP |
| -3.5900 | 3.31E-04 | GTExv8.EUR.Whole_Blood | 3.01E-03 | RWDD2B | DBP |
| -3.9670 | 7.27E-05 | GTExv8.EUR.Whole_Blood | 8.42E-04 | CCT8 | DBP |
| 3.2250 | 1.26E-03 | GTExv8.EUR.Whole_Blood | 9.00E-03 | MAP3K7CL | DBP |
| -2.6800 | 7.35E-03 | GTExv8.EUR.Whole_Blood | 3.69E-02 | LINC00189 | DBP |
| -2.9020 | 3.71E-03 | GTExv8.EUR.Whole_Blood | 2.16E-02 | GAPDHP14 | DBP |
| -2.6370 | 8.37E-03 | GTExv8.EUR.Whole_Blood | 4.11E-02 | OLIG1 | DBP |
| -2.9090 | 3.63E-03 | GTExv8.EUR.Whole_Blood | 2.12E-02 | IFNAR2 | DBP |
| 3.5850 | 3.37E-04 | GTExv8.EUR.Whole_Blood | 3.06E-03 | IFNGR2 | DBP |
| -4.8620 | 1.16E-06 | GTExv8.EUR.Whole_Blood | 2.18E-05 | ITSN1 | DBP |
| 3.3110 | 9.30E-04 | GTExv8.EUR.Whole_Blood | 7.03E-03 | MORC3 | DBP |
| 2.8870 | 3.88E-03 | GTExv8.EUR.Whole_Blood | 2.23E-02 | HLCS | DBP |
| -2.8320 | 4.62E-03 | GTExv8.EUR.Whole_Blood | 2.55E-02 | KCNJ15 | DBP |
| 2.9070 | 3.65E-03 | GTExv8.EUR.Whole_Blood | 2.13E-02 | BRWD1-AS2 | DBP |
| 3.3730 | 7.43E-04 | GTExv8.EUR.Whole_Blood | 5.86E-03 | BRWD1 | DBP |
| -3.9610 | 7.45E-05 | GTExv8.EUR.Whole_Blood | 8.58E-04 | UMODL1-AS1 | DBP |
| -2.9200 | 3.50E-03 | GTExv8.EUR.Whole_Blood | 2.06E-02 | U2AF1 | DBP |
| -8.6180 | 6.78E-18 | GTExv8.EUR.Whole_Blood | 1.01E-15 | RRP1B | DBP |
| 2.7760 | 5.51E-03 | GTExv8.EUR.Whole_Blood | 2.95E-02 | COL18A1 | DBP |
| 2.5900 | 9.60E-03 | GTExv8.EUR.Whole_Blood | 4.58E-02 | SLC19A1 | DBP |
| 2.6090 | 9.08E-03 | GTExv8.EUR.Whole_Blood | 4.38E-02 | PCNT | DBP |
| 2.7110 | 6.71E-03 | GTExv8.EUR.Whole_Blood | 3.44E-02 | DSTNP1 | DBP |
| -7.8007 | 6.15E-15 | GTExv8.EUR.Whole_Blood | 6.77E-13 | MICAL3 | DBP |
| 2.5824 | 9.81E-03 | GTExv8.EUR.Whole_Blood | 4.65E-02 | AC006547.13 | DBP |
| -3.1044 | 1.91E-03 | GTExv8.EUR.Whole_Blood | 1.26E-02 | PI4KA | DBP |
| 2.6118 | 9.01E-03 | GTExv8.EUR.Whole_Blood | 4.35E-02 | GNAZ | DBP |
| -3.3243 | 8.87E-04 | GTExv8.EUR.Whole_Blood | 6.75E-03 | MMP11 | DBP |
| -3.0149 | 2.57E-03 | GTExv8.EUR.Whole_Blood | 1.61E-02 | DERL3 | DBP |
| 2.7541 | 5.89E-03 | GTExv8.EUR.Whole_Blood | 3.09E-02 | MIATNB | DBP |
| 4.0796 | 4.51E-05 | GTExv8.EUR.Whole_Blood | 5.52E-04 | PITPNB | DBP |
| 3.0921 | 1.99E-03 | GTExv8.EUR.Whole_Blood | 1.31E-02 | KREMEN1 | DBP |
| 3.1605 | 1.57E-03 | GTExv8.EUR.Whole_Blood | 1.08E-02 | CTA-747E2.10 | DBP |
| 3.6772 | 2.36E-04 | GTExv8.EUR.Whole_Blood | 2.29E-03 | EMID1 | DBP |
| 4.4752 | 7.64E-06 | GTExv8.EUR.Whole_Blood | 1.16E-04 | GAS2L1 | DBP |
| -3.5865 | 3.35E-04 | GTExv8.EUR.Whole_Blood | 3.04E-03 | THOC5 | DBP |
| -2.6262 | 8.64E-03 | GTExv8.EUR.Whole_Blood | 4.22E-02 | NIPSNAP1 | DBP |
| 2.7904 | 5.26E-03 | GTExv8.EUR.Whole_Blood | 2.83E-02 | ZMAT5 | DBP |
| 2.9741 | 2.94E-03 | GTExv8.EUR.Whole_Blood | 1.79E-02 | UQCR10 | DBP |
| -3.0425 | 2.35E-03 | GTExv8.EUR.Whole_Blood | 1.49E-02 | CCDC157 | DBP |
| 4.5903 | 4.43E-06 | GTExv8.EUR.Whole_Blood | 7.15E-05 | MTFP1 | DBP |
| 4.1125 | 3.91E-05 | GTExv8.EUR.Whole_Blood | 4.90E-04 | SELENOM | DBP |
| -4.6984 | 2.62E-06 | GTExv8.EUR.Whole_Blood | 4.48E-05 | RNF185 | DBP |
| 3.6238 | 2.90E-04 | GTExv8.EUR.Whole_Blood | 2.70E-03 | PIK3IP1 | DBP |
| 4.5115 | 6.44E-06 | GTExv8.EUR.Whole_Blood | 1.00E-04 | SFI1 | DBP |
| 6.1305 | 8.76E-10 | GTExv8.EUR.Whole_Blood | 3.44E-08 | EIF4ENIF1 | DBP |
| 4.3327 | 1.47E-05 | GTExv8.EUR.Whole_Blood | 2.09E-04 | PISD | DBP |
| 4.8228 | 1.42E-06 | GTExv8.EUR.Whole_Blood | 2.60E-05 | DEPDC5 | DBP |
| 4.6828 | 2.83E-06 | GTExv8.EUR.Whole_Blood | 4.80E-05 | APOL3 | DBP |
| -3.6946 | 2.20E-04 | GTExv8.EUR.Whole_Blood | 2.15E-03 | TRIOBP | DBP |
| 3.1113 | 1.86E-03 | GTExv8.EUR.Whole_Blood | 1.24E-02 | PICK1 | DBP |
| -3.0383 | 2.38E-03 | GTExv8.EUR.Whole_Blood | 1.51E-02 | SYNGR1 | DBP |
| -2.7045 | 6.84E-03 | GTExv8.EUR.Whole_Blood | 3.50E-02 | EP300 | DBP |
| 4.2247 | 2.39E-05 | GTExv8.EUR.Whole_Blood | 3.22E-04 | L3MBTL2 | DBP |
| 4.2316 | 2.32E-05 | GTExv8.EUR.Whole_Blood | 3.14E-04 | RP4-756G23.5 | DBP |
| -2.8824 | 3.95E-03 | GTExv8.EUR.Whole_Blood | 2.26E-02 | RANGAP1 | DBP |
| 3.3564 | 7.90E-04 | GTExv8.EUR.Whole_Blood | 6.17E-03 | TEF | DBP |
| -2.5841 | 9.76E-03 | GTExv8.EUR.Whole_Blood | 4.63E-02 | SNU13 | DBP |
| -3.2515 | 1.15E-03 | GTExv8.EUR.Whole_Blood | 8.33E-03 | TNFRSF13C | DBP |
| 2.6481 | 8.09E-03 | GTExv8.EUR.Whole_Blood | 4.01E-02 | CENPM | DBP |
| 3.9061 | 9.38E-05 | GTExv8.EUR.Whole_Blood | 1.05E-03 | OGFRP1 | DBP |
| -3.0725 | 2.12E-03 | GTExv8.EUR.Whole_Blood | 1.37E-02 | TCF20 | DBP |
| 2.6416 | 8.25E-03 | GTExv8.EUR.Whole_Blood | 4.07E-02 | LINC00899 | DBP |
| -3.5190 | 4.33E-04 | GTExv8.EUR.Whole_Blood | 3.77E-03 | MIRLET7BHG | DBP |
| -3.4148 | 6.38E-04 | GTExv8.EUR.Whole_Blood | 5.19E-03 | ZBED4 | DBP |
| -2.8368 | 4.56E-03 | GTExv8.EUR.Whole_Blood | 2.53E-02 | CRELD2 | DBP |
| 4.3554 | 1.33E-05 | GTExv8.EUR.Whole_Blood | 1.91E-04 | RP3-402G11.28 | DBP |
| -2.9236 | 3.46E-03 | GTExv8.EUR.Whole_Blood | 2.04E-02 | TUBGCP6 | DBP |
| -2.6878 | 7.19E-03 | GTExv8.EUR.Whole_Blood | 3.63E-02 | DENND6B | DBP |
| 3.1276 | 1.76E-03 | GTExv8.EUR.Whole_Blood | 1.18E-02 | LMF2 | DBP |
| 4.5364 | 5.72E-06 | GTExv8.EUR.Whole_Blood | 1.23E-02 | CYP3A5 | DKD |
| -4.5186 | 6.22E-06 | GTExv8.EUR.Whole_Blood | 1.23E-02 | INTS8 | DKD |
| 4.2350 | 2.29E-05 | GTExv8.EUR.Whole_Blood | 3.64E-02 | TP53INP1 | DKD |
| -5.3197 | 1.04E-07 | GTExv8.EUR.Whole_Blood | 8.25E-04 | PRKD2 | DKD |
| -4.7471 | 2.06E-06 | GTExv8.EUR.Whole_Blood | 8.18E-03 | FKRP | DKD |
| -4.1321 | 3.59E-05 | GTExv8.EUR.Whole_Blood | 4.75E-02 | UQCR10 | DKD |
| 2.9708 | 2.97E-03 | GTExv8.EUR.Whole_Blood | 2.87E-02 | PLEKHN1 | Education |
| -2.9387 | 3.30E-03 | GTExv8.EUR.Whole_Blood | 3.08E-02 | ATAD3A | Education |
| -2.9309 | 3.38E-03 | GTExv8.EUR.Whole_Blood | 3.12E-02 | CDK11B | Education |
| 4.2554 | 2.09E-05 | GTExv8.EUR.Whole_Blood | 6.12E-04 | RP1-140A9.1 | Education |
| -2.8055 | 5.02E-03 | GTExv8.EUR.Whole_Blood | 4.08E-02 | RERE | Education |
| -2.8686 | 4.12E-03 | GTExv8.EUR.Whole_Blood | 3.57E-02 | RP3-477M7.5 | Education |
| 2.8830 | 3.94E-03 | GTExv8.EUR.Whole_Blood | 3.46E-02 | SPSB1 | Education |
| 3.1069 | 1.89E-03 | GTExv8.EUR.Whole_Blood | 2.06E-02 | NBPF1 | Education |
| 3.0449 | 2.33E-03 | GTExv8.EUR.Whole_Blood | 2.42E-02 | CROCCP2 | Education |
| 3.4439 | 5.73E-04 | GTExv8.EUR.Whole_Blood | 8.25E-03 | LINC00339 | Education |
| 3.1330 | 1.73E-03 | GTExv8.EUR.Whole_Blood | 1.92E-02 | CDC42 | Education |
| 2.8915 | 3.83E-03 | GTExv8.EUR.Whole_Blood | 3.40E-02 | SESN2 | Education |
| 5.4433 | 5.23E-08 | GTExv8.EUR.Whole_Blood | 3.70E-06 | PHACTR4 | Education |
| 5.4788 | 4.28E-08 | GTExv8.EUR.Whole_Blood | 3.16E-06 | SNHG12 | Education |
| 4.0467 | 5.19E-05 | GTExv8.EUR.Whole_Blood | 1.24E-03 | YTHDF2 | Education |
| 3.5108 | 4.47E-04 | GTExv8.EUR.Whole_Blood | 6.97E-03 | MECR | Education |
| 2.7566 | 5.84E-03 | GTExv8.EUR.Whole_Blood | 4.57E-02 | SERINC2 | Education |
| -4.4315 | 9.36E-06 | GTExv8.EUR.Whole_Blood | 3.21E-04 | PEF1 | Education |
| 4.4099 | 1.03E-05 | GTExv8.EUR.Whole_Blood | 3.46E-04 | MEAF6 | Education |
| 2.8995 | 3.74E-03 | GTExv8.EUR.Whole_Blood | 3.35E-02 | GNL2 | Education |
| 2.8175 | 4.84E-03 | GTExv8.EUR.Whole_Blood | 3.99E-02 | NDUFS5 | Education |
| -3.5535 | 3.80E-04 | GTExv8.EUR.Whole_Blood | 6.15E-03 | HPCAL4 | Education |
| 3.4226 | 6.20E-04 | GTExv8.EUR.Whole_Blood | 8.72E-03 | EXO5 | Education |
| -3.0069 | 2.64E-03 | GTExv8.EUR.Whole_Blood | 2.62E-02 | NFYC | Education |
| 3.0486 | 2.30E-03 | GTExv8.EUR.Whole_Blood | 2.39E-02 | TIE1 | Education |
| 6.2529 | 4.03E-10 | GTExv8.EUR.Whole_Blood | 4.94E-08 | SZT2 | Education |
| -6.3838 | 1.73E-10 | GTExv8.EUR.Whole_Blood | 2.21E-08 | CCDC24 | Education |
| -3.2918 | 9.95E-04 | GTExv8.EUR.Whole_Blood | 1.25E-02 | PRDX1 | Education |
| 3.5661 | 3.62E-04 | GTExv8.EUR.Whole_Blood | 5.93E-03 | RP11-767N6.2 | Education |
| -2.8924 | 3.82E-03 | GTExv8.EUR.Whole_Blood | 3.39E-02 | MAST2 | Education |
| -2.9785 | 2.90E-03 | GTExv8.EUR.Whole_Blood | 2.83E-02 | RP4-657D16.3 | Education |
| 4.1058 | 4.03E-05 | GTExv8.EUR.Whole_Blood | 1.03E-03 | C1orf123 | Education |
| 3.2485 | 1.16E-03 | GTExv8.EUR.Whole_Blood | 1.40E-02 | HHLA3 | Education |
| 2.9634 | 3.04E-03 | GTExv8.EUR.Whole_Blood | 2.91E-02 | CTH | Education |
| -7.2948 | 2.99E-13 | GTExv8.EUR.Whole_Blood | 5.54E-11 | FPGT | Education |
| -5.0738 | 3.90E-07 | GTExv8.EUR.Whole_Blood | 2.12E-05 | TNNI3K | Education |
| 4.8729 | 1.10E-06 | GTExv8.EUR.Whole_Blood | 5.28E-05 | CRYZ | Education |
| -2.8244 | 4.74E-03 | GTExv8.EUR.Whole_Blood | 3.92E-02 | RP4-612B15.3 | Education |
| 3.4856 | 4.91E-04 | GTExv8.EUR.Whole_Blood | 7.43E-03 | KYAT3 | Education |
| 3.5280 | 4.19E-04 | GTExv8.EUR.Whole_Blood | 6.62E-03 | TMED5 | Education |
| 2.9543 | 3.13E-03 | GTExv8.EUR.Whole_Blood | 2.97E-02 | CCDC18 | Education |
| -3.0825 | 2.05E-03 | GTExv8.EUR.Whole_Blood | 2.19E-02 | DR1 | Education |
| 2.8712 | 4.09E-03 | GTExv8.EUR.Whole_Blood | 3.56E-02 | RP4-713B5.2 | Education |
| 2.8123 | 4.92E-03 | GTExv8.EUR.Whole_Blood | 4.04E-02 | RWDD3 | Education |
| 3.3243 | 8.86E-04 | GTExv8.EUR.Whole_Blood | 1.13E-02 | DPYD | Education |
| -3.3726 | 7.45E-04 | GTExv8.EUR.Whole_Blood | 9.97E-03 | FRRS1 | Education |
| 3.3292 | 8.71E-04 | GTExv8.EUR.Whole_Blood | 1.12E-02 | PRMT6 | Education |
| 2.9578 | 3.10E-03 | GTExv8.EUR.Whole_Blood | 2.95E-02 | SYPL2 | Education |
| -5.2295 | 1.70E-07 | GTExv8.EUR.Whole_Blood | 1.06E-05 | ATXN7L2 | Education |
| 5.1793 | 2.23E-07 | GTExv8.EUR.Whole_Blood | 1.30E-05 | AMIGO1 | Education |
| -4.5522 | 5.31E-06 | GTExv8.EUR.Whole_Blood | 2.01E-04 | STRIP1 | Education |
| 4.2635 | 2.01E-05 | GTExv8.EUR.Whole_Blood | 5.97E-04 | KCNC4 | Education |
| 5.1066 | 3.28E-07 | GTExv8.EUR.Whole_Blood | 1.84E-05 | RAP1A | Education |
| 5.1552 | 2.53E-07 | GTExv8.EUR.Whole_Blood | 1.45E-05 | LINC01160 | Education |
| 3.3658 | 7.63E-04 | GTExv8.EUR.Whole_Blood | 1.01E-02 | NUDT17 | Education |
| -3.5693 | 3.58E-04 | GTExv8.EUR.Whole_Blood | 5.89E-03 | ECM1 | Education |
| -4.6819 | 2.84E-06 | GTExv8.EUR.Whole_Blood | 1.20E-04 | RP11-54A4.2 | Education |
| 2.8283 | 4.68E-03 | GTExv8.EUR.Whole_Blood | 3.89E-02 | LYSMD1 | Education |
| -2.8310 | 4.64E-03 | GTExv8.EUR.Whole_Blood | 3.87E-02 | RFX5 | Education |
| -3.5368 | 4.05E-04 | GTExv8.EUR.Whole_Blood | 6.48E-03 | SLC39A1 | Education |
| -6.1469 | 7.90E-10 | GTExv8.EUR.Whole_Blood | 8.99E-08 | FCER1A | Education |
| 3.4179 | 6.31E-04 | GTExv8.EUR.Whole_Blood | 8.83E-03 | SLAMF9 | Education |
| 4.4661 | 7.96E-06 | GTExv8.EUR.Whole_Blood | 2.82E-04 | NME7 | Education |
| -2.9251 | 3.44E-03 | GTExv8.EUR.Whole_Blood | 3.16E-02 | BLZF1 | Education |
| -2.9764 | 2.92E-03 | GTExv8.EUR.Whole_Blood | 2.84E-02 | SELP | Education |
| 4.5571 | 5.19E-06 | GTExv8.EUR.Whole_Blood | 1.98E-04 | RP11-160H22.5 | Education |
| -3.7360 | 1.87E-04 | GTExv8.EUR.Whole_Blood | 3.51E-03 | CACYBP | Education |
| 2.8321 | 4.62E-03 | GTExv8.EUR.Whole_Blood | 3.86E-02 | SMG7-AS1 | Education |
| 2.8110 | 4.94E-03 | GTExv8.EUR.Whole_Blood | 4.04E-02 | SMG7 | Education |
| 3.9746 | 7.05E-05 | GTExv8.EUR.Whole_Blood | 1.60E-03 | ARPC5 | Education |
| 5.1026 | 3.35E-07 | GTExv8.EUR.Whole_Blood | 1.87E-05 | EDEM3 | Education |
| 3.7079 | 2.09E-04 | GTExv8.EUR.Whole_Blood | 3.81E-03 | RGS2 | Education |
| -4.1630 | 3.14E-05 | GTExv8.EUR.Whole_Blood | 8.48E-04 | C1orf106 | Education |
| -5.0729 | 3.92E-07 | GTExv8.EUR.Whole_Blood | 2.12E-05 | MDM4 | Education |
| 2.8785 | 4.00E-03 | GTExv8.EUR.Whole_Blood | 3.50E-02 | RBBP5 | Education |
| -4.3200 | 1.56E-05 | GTExv8.EUR.Whole_Blood | 4.95E-04 | TRAF5 | Education |
| -4.3075 | 1.65E-05 | GTExv8.EUR.Whole_Blood | 5.15E-04 | LPGAT1 | Education |
| 5.2063 | 1.93E-07 | GTExv8.EUR.Whole_Blood | 1.14E-05 | TMEM206 | Education |
| 3.8600 | 1.13E-04 | GTExv8.EUR.Whole_Blood | 2.35E-03 | NENF | Education |
| 3.9126 | 9.13E-05 | GTExv8.EUR.Whole_Blood | 2.00E-03 | RPS6KC1 | Education |
| -3.4141 | 6.40E-04 | GTExv8.EUR.Whole_Blood | 8.90E-03 | RAB3GAP2 | Education |
| 3.0355 | 2.40E-03 | GTExv8.EUR.Whole_Blood | 2.46E-02 | NVL | Education |
| -5.9934 | 2.05E-09 | GTExv8.EUR.Whole_Blood | 2.07E-07 | TBCE | Education |
| -3.7608 | 1.69E-04 | GTExv8.EUR.Whole_Blood | 3.25E-03 | B3GALNT2 | Education |
| 2.9236 | 3.46E-03 | GTExv8.EUR.Whole_Blood | 3.17E-02 | RP11-385F5.4 | Education |
| 3.0234 | 2.50E-03 | GTExv8.EUR.Whole_Blood | 2.53E-02 | CHRM3-AS2 | Education |
| -3.7569 | 1.72E-04 | GTExv8.EUR.Whole_Blood | 3.30E-03 | OPN3 | Education |
| 7.2048 | 5.82E-13 | GTExv8.EUR.Whole_Blood | 1.05E-10 | LINC01347 | Education |
| -8.1506 | 3.62E-16 | GTExv8.EUR.Whole_Blood | 9.30E-14 | CEP170 | Education |
| -8.2134 | 2.15E-16 | GTExv8.EUR.Whole_Blood | 5.87E-14 | SDCCAG8 | Education |
| 3.0103 | 2.61E-03 | GTExv8.EUR.Whole_Blood | 2.60E-02 | RNASEH1-AS1 | Education |
| 3.3732 | 7.43E-04 | GTExv8.EUR.Whole_Blood | 9.96E-03 | RP11-791G15.2 | Education |
| 2.9809 | 2.87E-03 | GTExv8.EUR.Whole_Blood | 2.82E-02 | TRIB2 | Education |
| -4.4310 | 9.38E-06 | GTExv8.EUR.Whole_Blood | 3.21E-04 | NBAS | Education |
| -2.8868 | 3.89E-03 | GTExv8.EUR.Whole_Blood | 3.43E-02 | FAM228B | Education |
| 2.7245 | 6.44E-03 | GTExv8.EUR.Whole_Blood | 4.94E-02 | DTNB | Education |
| -2.8018 | 5.08E-03 | GTExv8.EUR.Whole_Blood | 4.10E-02 | SELENOI | Education |
| -3.8515 | 1.17E-04 | GTExv8.EUR.Whole_Blood | 2.41E-03 | PPP1CB | Education |
| 4.3995 | 1.09E-05 | GTExv8.EUR.Whole_Blood | 3.63E-04 | TRMT61B | Education |
| 2.7489 | 5.98E-03 | GTExv8.EUR.Whole_Blood | 4.65E-02 | TOGARAM2 | Education |
| 2.8046 | 5.04E-03 | GTExv8.EUR.Whole_Blood | 4.08E-02 | CLIP4 | Education |
| 3.6295 | 2.84E-04 | GTExv8.EUR.Whole_Blood | 4.89E-03 | NLRC4 | Education |
| -3.8532 | 1.17E-04 | GTExv8.EUR.Whole_Blood | 2.41E-03 | PREPL | Education |
| 7.6050 | 2.85E-14 | GTExv8.EUR.Whole_Blood | 5.82E-12 | CAMKMT | Education |
| -3.7754 | 1.60E-04 | GTExv8.EUR.Whole_Blood | 3.09E-03 | FOXN2 | Education |
| -3.3064 | 9.45E-04 | GTExv8.EUR.Whole_Blood | 1.19E-02 | GPR75 | Education |
| -3.7034 | 2.13E-04 | GTExv8.EUR.Whole_Blood | 3.86E-03 | PUS10 | Education |
| -4.1955 | 2.72E-05 | GTExv8.EUR.Whole_Blood | 7.55E-04 | AC016747.3 | Education |
| -5.4637 | 4.66E-08 | GTExv8.EUR.Whole_Blood | 3.40E-06 | AHSA2 | Education |
| 3.7160 | 2.02E-04 | GTExv8.EUR.Whole_Blood | 3.70E-03 | RP11-493E12.2 | Education |
| -2.9132 | 3.58E-03 | GTExv8.EUR.Whole_Blood | 3.25E-02 | AC008074.5 | Education |
| -2.8143 | 4.89E-03 | GTExv8.EUR.Whole_Blood | 4.02E-02 | CEP68 | Education |
| 3.6039 | 3.13E-04 | GTExv8.EUR.Whole_Blood | 5.27E-03 | TEX261 | Education |
| -2.7408 | 6.13E-03 | GTExv8.EUR.Whole_Blood | 4.74E-02 | RAB11FIP5 | Education |
| 3.2411 | 1.19E-03 | GTExv8.EUR.Whole_Blood | 1.43E-02 | FBXO41 | Education |
| -3.6567 | 2.55E-04 | GTExv8.EUR.Whole_Blood | 4.49E-03 | ALMS1 | Education |
| -2.8701 | 4.10E-03 | GTExv8.EUR.Whole_Blood | 3.56E-02 | RP11-434P11.2 | Education |
| -3.4094 | 6.51E-04 | GTExv8.EUR.Whole_Blood | 9.00E-03 | TET3 | Education |
| -3.5987 | 3.20E-04 | GTExv8.EUR.Whole_Blood | 5.38E-03 | CIAO1 | Education |
| -3.8049 | 1.42E-04 | GTExv8.EUR.Whole_Blood | 2.82E-03 | ANKRD36 | Education |
| -3.4417 | 5.78E-04 | GTExv8.EUR.Whole_Blood | 8.29E-03 | ACTR1B | Education |
| -3.5612 | 3.69E-04 | GTExv8.EUR.Whole_Blood | 6.02E-03 | LYG1 | Education |
| -3.2914 | 9.97E-04 | GTExv8.EUR.Whole_Blood | 1.25E-02 | RP11-527J8.1 | Education |
| 4.5075 | 6.56E-06 | GTExv8.EUR.Whole_Blood | 2.41E-04 | REV1 | Education |
| -7.6223 | 2.49E-14 | GTExv8.EUR.Whole_Blood | 5.22E-12 | AFF3 | Education |
| 8.2102 | 2.21E-16 | GTExv8.EUR.Whole_Blood | 5.87E-14 | CHST10 | Education |
| 3.2214 | 1.28E-03 | GTExv8.EUR.Whole_Blood | 1.52E-02 | RGPD8 | Education |
| -3.5280 | 4.19E-04 | GTExv8.EUR.Whole_Blood | 6.62E-03 | AC009303.2 | Education |
| 3.6295 | 2.84E-04 | GTExv8.EUR.Whole_Blood | 4.89E-03 | INSIG2 | Education |
| -3.0560 | 2.24E-03 | GTExv8.EUR.Whole_Blood | 2.34E-02 | STEAP3 | Education |
| -3.8322 | 1.27E-04 | GTExv8.EUR.Whole_Blood | 2.58E-03 | MTATP6P26 | Education |
| -2.7916 | 5.25E-03 | GTExv8.EUR.Whole_Blood | 4.19E-02 | RP11-286H15.1 | Education |
| 4.5366 | 5.72E-06 | GTExv8.EUR.Whole_Blood | 2.15E-04 | GPD2 | Education |
| 9.1511 | 5.63E-20 | GTExv8.EUR.Whole_Blood | 3.20E-17 | LINC01806 | Education |
| -3.2136 | 1.31E-03 | GTExv8.EUR.Whole_Blood | 1.55E-02 | FASTKD1 | Education |
| -5.8397 | 5.23E-09 | GTExv8.EUR.Whole_Blood | 4.84E-07 | HAT1 | Education |
| -5.6233 | 1.87E-08 | GTExv8.EUR.Whole_Blood | 1.54E-06 | SLC25A12 | Education |
| -3.3286 | 8.73E-04 | GTExv8.EUR.Whole_Blood | 1.12E-02 | CIR1 | Education |
| 4.7435 | 2.10E-06 | GTExv8.EUR.Whole_Blood | 9.45E-05 | CWC22 | Education |
| 2.9515 | 3.16E-03 | GTExv8.EUR.Whole_Blood | 3.00E-02 | ITGA4 | Education |
| 2.8448 | 4.44E-03 | GTExv8.EUR.Whole_Blood | 3.76E-02 | CERKL | Education |
[truncated: 234,484 more chars]
